# Discovery of (*R*)-*N*-Benzyl-2-(2,5-dioxopyrrolidin-1-yl)propanamide [**(*R*)-AS-1**], a Novel Orally Bioavailable EAAT2 Modulator with Drug-like Properties and Potent Antiseizure Activity *In Vivo*

**DOI:** 10.1021/acs.jmedchem.2c00534

**Published:** 2022-08-19

**Authors:** Michał Abram, Marcin Jakubiec, Katelyn Reeb, Mary Hongying Cheng, Robin Gedschold, Anna Rapacz, Szczepan Mogilski, Katarzyna Socała, Dorota Nieoczym, Małgorzata Szafarz, Gniewomir Latacz, Bartłomiej Szulczyk, Justyna Kalinowska-Tłuścik, Kinga Gawel, Camila V. Esguerra, Elżbieta Wyska, Christa E. Müller, Ivet Bahar, Andréia C. K. Fontana, Piotr Wlaź, Rafał M. Kamiński, Krzysztof Kamiński

**Affiliations:** † Department of Medicinal Chemistry, Faculty of Pharmacy, Jagiellonian University Medical College, Medyczna 9, 30-688Krakow, Poland; ‡ Department of Pharmacology and Physiology, Drexel University College of Medicine, Philadelphia, Pennsylvania19102, United States; § Department of Computational and Systems Biology, School of Medicine, University of Pittsburgh, Pittsburgh, Pennsylvania15213, United States; ∥ Department of Pharmacodynamics, Faculty of Pharmacy, Jagiellonian University Medical College, Medyczna 9, 30-688Krakow, Poland; ⊥ PharmaCenter Bonn, Pharmaceutical Institute, Pharmaceutical & Medicinal Chemistry, Rheinische Friedrich-Wilhelms-Universität Bonn, An der Immenburg 4, D-53121Bonn, Germany; # Department of Animal Physiology and Pharmacology, Institute of Biological Sciences, Faculty of Biology and Biotechnology, Maria Curie-Skłodowska University, Akademicka 19, 20-033Lublin, Poland; ∇ Department of Pharmacokinetics and Physical Pharmacy, Faculty of Pharmacy, Jagiellonian University Medical College, Medyczna 9, 30-688Krakow, Poland; ○ Department of Technology and Biotechnology of Drugs, Faculty of Pharmacy, Jagiellonian University Medical College, Medyczna 9, 30-688Krakow, Poland; ◆ Department of Pharmacodynamics, Centre for Preclinical Research and Technology, Medical University of Warsaw, Banacha 1B, 02-097Warsaw, Poland; ¶ Department of Crystal Chemistry and Crystal Physics, Faculty of Chemistry, Jagiellonian University, Gronostajowa 2, 30-387Krakow, Poland; †† Department of Experimental and Clinical Pharmacology, Medical University of Lublin, Jaczewskiego 8B, 20-090Lublin, Poland; ‡‡ Chemical Neuroscience Group, Centre for Molecular Medicine Norway, University of Oslo, Gaustadalléen 21, Forskningsparken, 0349Oslo, Norway

## Abstract

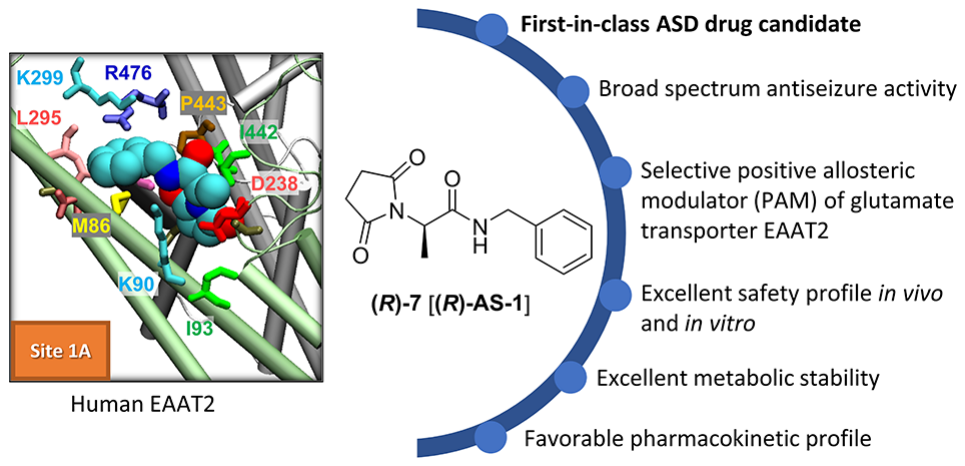

**(*****R*****)-7** [**(*****R*****)-AS-1**] showed broad-spectrum antiseizure activity across *in vivo* mouse seizure models: maximal electroshock (MES), 6 Hz (32/44 mA), acute pentylenetetrazol (PTZ), and PTZ-kindling. A remarkable separation between antiseizure activity and CNS-related adverse effects was also observed. *In vitro* studies with primary glia cultures and COS-7 cells expressing the glutamate transporter EAAT2 showed enhancement of glutamate uptake, revealing a stereoselective positive allosteric modulator (PAM) effect, further supported by molecular docking simulations. **(*****R*****)-7** [**(*****R*****)-AS-1**] was not active in EAAT1 and EAAT3 assays and did not show significant off-target activity, including interactions with targets reported for marketed antiseizure drugs, indicative of a novel and unprecedented mechanism of action. Both *in vivo* pharmacokinetic and *in vitro* absorption, distribution, metabolism, excretion, toxicity (ADME-Tox) profiles confirmed the favorable drug-like potential of the compound. Thus, **(*****R*****)-7** [**(*****R*****)-AS-1**] may be considered as the first-in-class small-molecule PAM of EAAT2 with potential for further preclinical and clinical development in epilepsy and possibly other CNS disorders.

## Introduction

Epilepsy is a common neurological disorder characterized by spontaneous and recurrent seizures, often accompanied by a spectrum of neuropsychiatric symptoms. It is also a very heterogeneous disease with multifactorial and complex etiology. There are many different types of epilepsies, and the disease affects more than 70 million patients globally.^1^ Progress in epilepsy research has led to the approval and marketing authorization of more than 30 antiseizure drugs (ASDs) over recent decades. Yet, despite this unquestionable therapeutic success, approximately one-third of epilepsy patients still experience uncontrolled and debilitating seizures.^2^ These patients are considered as having the so-called drug-resistant epilepsy, which is defined as the failure of adequate trials of two tolerated and appropriately chosen ASD schedules (whether as monotherapies or in combination) to achieve seizure freedom.^3^ The mechanisms underlying drug resistance in epilepsy are complex and still relatively poorly understood, which in combination with the multifactorial etiology and pathophysiology of epilepsy immensely complicate the rational selection of ASDs for optimal therapy. As such, drug-resistant epilepsy creates an urgent unmet medical need and propels ongoing drug discovery and development programs globally.

Currently approved ASDs work through a number of mechanisms to restore the disturbed balance between excitatory and inhibitory neurotransmission responsible for seizure generation.^4^ Such drugs target mainly ion channels (*e.g.*, sodium or potassium channels), excitatory and inhibitory receptors, or presynaptic neurotransmitter release mechanisms. So far, only a few ASDs target neurotransmitter uptake mechanisms, mainly γ-aminobutyric acid (GABA) uptake (*i.e.*, tiagabine). However, glutamate uptake dysregulation, which is not yet specifically targeted by ASDs, emerges as one of the critical drivers of excitotoxicity and seizures.^5^

The excitatory amino acid transporter-2 (EAAT2) is one of the major glutamate transporters expressed in the brain and accounts for approximately 90% of glutamate uptake from the synapses.^5,6^ It is primarily localized on astrocytes in the central nervous system (CNS) and is essential for maintaining a low concentration of glutamate in the synaptic cleft and for avoiding excitotoxicity.^7,8^ Glutamate excitotoxicity plays a key role in the secondary damage following several pathologies such as amyotrophic lateral sclerosis, Alzheimer’s disease, Parkinson’s disease, Huntington’s disease, ischemia, schizophrenia, neuropathic pain, anxiety, depression, and autism.^9−13^ Importantly, numerous studies consistently showed elevated glutamate levels in patients with epilepsy resulting from failure of glutamate transport mechanisms.^14−16^ Furthermore, several research groups have demonstrated dramatic reductions in EAAT2 in brain tissue surgically resected in patients with epilepsy, which can be responsible for triggering seizures.^17−21^ Consistently, transgenic mice with increased expression of EAAT2 displayed protection against seizures and epilepsy development.^22^ Further, a small-molecule compound that enhances EAAT2-mediated glutamate uptake by indirect upregulation of the transporter expression also showed protective effects in an epilepsy model.^23^ However, so far, compounds that directly activate EAAT2 did not show favorable drug-like properties and have not been examined in epilepsy models. Such a class of compounds might offer clinical advantages over drugs that increase EAAT2 expression, such as ceftriaxone,^24^ including lower risk of toxicity and adverse effects after chronic treatment.^25^

**Figure 1:**
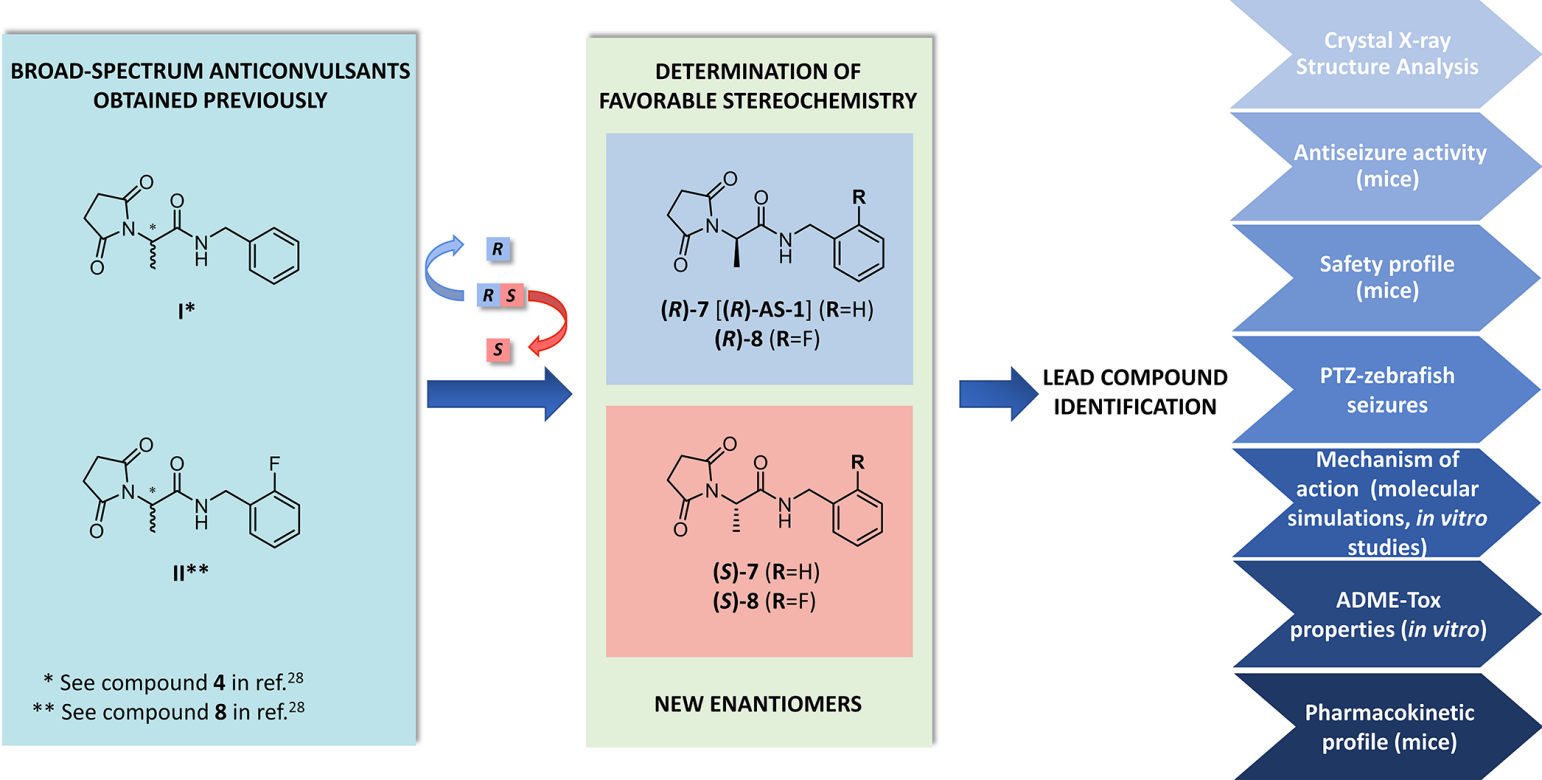
Development strategy leading to the identification of the lead anticonvulsant with a defined stereochemistry.

In recent years, chiral molecules have been actively pursued by the pharmaceutical industry and have played an important role in the development of new drugs. It should be mentioned that enantiomers/diastereoisomers of drug candidates often display marked differences in pharmacodynamic, pharmacokinetic, and toxicological properties.^26^ The chiral-switch approach of the already marketed racemates and/or introduction of drug candidates in a predefined enantiomeric form often results in improved potency and/or decreased toxicity for new compounds. Therefore, there is a need for full characterization and examination of each stereoisomer at the early stages of new drug development. This trend is also strongly visible in the case of novel ASDs, which have been used in pharmacotherapy in recent years as single enantiomers, *i.e.*, lacosamide (LCS), levetiracetam (LEV), cannabidiol (CBD), and cenobamate. The latter was approved by the Food and Drug Administration (FDA) in 2019 for the treatment of patients with focal-onset seizures.^27^ As a consequence, a part of our lead compound optimization process focused on the development of *R*- and *S*-enantiomers of the most promising racemates of recently discovered compounds (**I**, **II**) characterized by a robust and broad-spectrum antiseizure activity.^28,29^ The strategy for development of the lead compound with a defined stereochemistry is shown in Figure 1.

Thus, the main goal of the current study was to synthesize a lead compound with a favorable stereochemistry for antiseizure efficacy, to confirm its drug-like properties, to elucidate the mechanism of action, and to enable future preclinical and possibly clinical investigations. We report here our chemical and pharmacological studies that led to the discovery of the compound **(*****R*****)-7** [**(*****R*****)-AS-1**] (see the enantiomer in Figure 1) as a highly selective and orally bioavailable positive allosteric modulator (PAM) of EAAT2. **(*****R*****)-7** [**(*****R*****)-AS-1**] is shown to have robust antiseizure properties in a broad range of epilepsy models in mice and zebrafish, as well as an excellent tolerability and safety profile in both *in vitro* and *in vivo* studies.

## Results and Discussion

The pyrrolidine-2,5-dione ring is known to be a core fragment for many compounds with diverse therapeutic activities, *i.e.*, anticonvulsant, antipsychotic, antidepressant, anti-inflammatory, antibacterial, antiviral, or anticancer.^30,31^ Our research has been focused for many years on the development of new succinimides acting on the CNS. The most recent studies led to the identification of several groups of pyrrolidine-2,5-dione derivatives, characterized by potent antiseizure and antinociceptive efficacy in preclinical studies.^32−36^ Among these substances, *N*-benzyl-(2,5-dioxopyrrolidin-1-yl)propanamides represented by compounds **I** and **II** shown in Figure 1 revealed especially favorable anticonvulsant and safety profiles.^28,29,37^ Both compounds **I** and **II** showed antiseizure properties in commonly employed screening seizure models such as the maximal electroshock seizure test (MES), the subcutaneous pentylenetetrazol seizure test (*sc*PTZ), as well as the 6 Hz seizure model (32 and 44 mA) in mice (Table 1). They both exhibited a more potent efficacy or/and a wider spectrum of protection compared to several clinically relevant ASDs, such as ethosuximide (ETX), LEV, LCS, and especially valproic acid (VPA). Importantly, VPA is recognized as one of the most effective first-line ASD in the treatment of different types of seizures (*e.g.*, primary generalized tonic-clonic, partial, absence, atypical absence myoclonic, and atonic seizures).^38,39^ It is also noteworthy that compound **I** delayed the progression of kindling induced by repeated injections of PTZ, as well as displayed a supra-additive (synergistic) interaction with VPA against PTZ-induced seizures in mice.^29^ Thus, **I** may be potentially used as an add-on therapy with VPA. The *in vitro* absorption, distribution, metabolism, excretion, toxicity (ADME-Tox) studies showed favorable drug-like properties for **I**, displaying a good permeability in the parallel artificial membrane permeability assay (PAMPA), an excellent metabolic stability in human liver microsomes (HLMs), no influence on CYP3A4/CYP2D6 activity, as well as no hepatotoxic properties in HepG2 cells.^29^ Interestingly, despite the potent and wide-spectrum anticonvulsant activity, the mechanism of action for this molecule has not been elucidated until now.

**Table 1:** ED_50_, TD_50_, and Protective Index (PI) Values in Mice after Intraperitoneal (i.p.) Dosing of the Newly Obtained Compounds and Reference Substances^a^

compd	PT (h)^b^	ED_50_ (MES) (mg/kg)	ED_50_ (6 Hz 32 mA) (mg/kg)	ED_50_ (*sc*PTZ) (mg/kg)	TD_50_ (rotarod) (mg/kg)	PI (TD_50_/ED_50_)
**(*****R*****)-7**	0.5	**66.3** (53.6–82.0)^c^	**15.6** (9.1–26.9)	**36.3** (15.5–73.5)	**>500**	**>7.5 (MES)**
						**>32.0 (6 Hz)**
						**>13.8 (*****sc*****PTZ)**
**(*****S*****)-7**	0.5	87.5 (69.5–110.2)^c^	28.8 (16.9–48.9)	52.7 (37.7–85.0)	>500	>5.7 (MES)
						>17.4 (6 Hz)
						>9.5 (*sc*PTZ)
**(*****R*****)-8**	0.5	33.0 (22.5–48.2)^c^	14.1 (8.4–23.5)	33.2 (29.3–37.5)	224.7 (186.1–71.4)	6.8 (MES)
						16.0 (6 Hz)
						6.8 (*sc*PTZ)
**(*****S*****)-8**	0.5	49.9 (44.7–55.8)^c^	62.9 (45.7–86.6)	78.8 (53.2–95.3)	>300	>6.0 (MES)
						>4.8 (6 Hz)
						>3.8 (*sc*PTZ)
**I^d^**	0.5	67.6 (56.3–81.2)	24.6 (18.1–33.5)	42.8 (24.4–74.9)	347.6 (307.5–392.8)	5.1 (MES)
						14.1 (6 Hz)
						8.1 (*sc*PTZ)
**II**^d^	0.5	54.9 (48.3–62.3)	33.8 (11.0–103.7)	50.3 (34.7–72.6)	300.9 (256.7–352.6)	5.5 (MES)
						8.9 (6 Hz)
						6.0 (*sc*PTZ)
**ETX^e^**	0.25	n.a.	>200	140.4 (115.8–170.2)	318.0 (295.8–341.9)	2.3 (*sc*PTZ)
**LCS**^e^	0.5	9.2 (8.5–10.0)	5.3 (3.5–7.8)	n.a.	46.2 (44.5–48.0)	5.0 (MES)
						8.8 (6 Hz)
**LEV**^e^	1.0	>500	15.7 (10.4–23.7)	n.a.	>500	>31.8 (6 Hz)
**VPA**^e^	0.5	252.7 (220.1–290.2)	130.6 (117.6–145.2)	239.4 (209.2–274.1)	430.7 (407.9–454.9)	1.7 (MES)
						3.3 (6 Hz)
						1.8 (*sc*PTZ)
**CBD^f^**	1.0	80 (65.5–96.0)	144 (102–194)	120 (98.5–146)	272 (241–303)	3.4 (MES)
						1.9 (6 Hz)
						2.3 (*sc*PTZ)

a The data for lead compound **(*****R*****)-7** [**(*****R*****)-AS-1**] are shown in bold for better visualization. Values in parentheses are 95% confidence intervals. n.a.—non-active.

b Pretreatment time.

c No mortality was observed in the MES model for **(*****R*****)-7** and **(*****R*****)-8**, *ca.* 16% mortality was noted for **(*****S*****)-7** and **(*****S*****)-8**, whereas *ca.* 66% mortality was observed in the control group.

d Data for racemates **I** and **II** (compounds **4** and **8**, respectively).^28^

e Reference ASDs: ETX, LCS, LEV, and VPA tested under the same conditions, data taken from own experiments or literature.^40^

f CBD data.^41^

Taking into consideration the aforementioned promising results for racemates (especially **I**) in the current study, we have obtained their separate *R-* and *S*-enantiomers applying an asymmetric synthetic procedure. These stereoisomers were tested *in vivo* for antiseizure activity in several acute seizure models, *i.e.*, MES, *sc*PTZ, and 6 Hz (32 and 44 mA). The lead compound was evaluated in the PTZ kindling model in mice and in the PTZ-induced model of tonic-clonic seizures in zebrafish. Additionally, as a part of the safety *in vivo* profiling for the lead molecule, we determined its influence on coordination and locomotor activity in mice. We also assessed its *in vivo* pharmacokinetic profile and several *in vitro* ADME-Tox parameters, such as hepatotoxicity, neurotoxicity, and influence on the function of crucial cytochrome P-450 isoforms (*i.e.*, CYP3A4, CYP2D6, and CYP2C9), to support the early development process. Finally, we examined the mechanism of action of the lead compound by a combination of *in silico* and *in vitro* studies.

### Synthesis

**Scheme 1:**
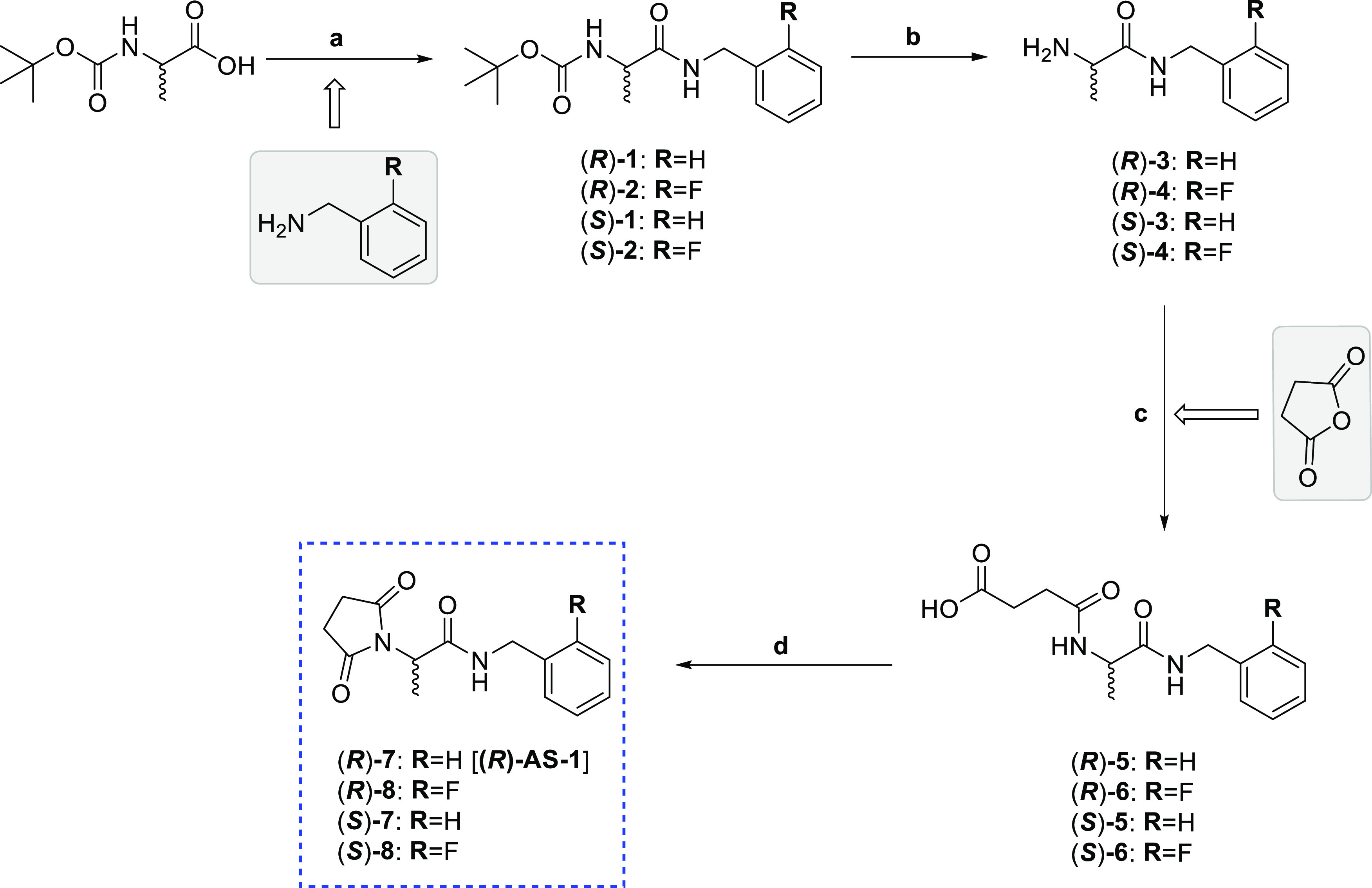
Synthesis of Target Enantiomers **(*****R*****)-7** [**(*****R*****)-AS-1**], **(*R*)-8**, and **(*S*)**-**7**, **(*S*)**-**8** Reaction conditions: (a) DCC, dichloromethane (DCM), room temperature (rt), 1 h; (b), TFA, DCM, rt, 1 h; (c) AcOEt, rt, 0.5 h; (d) HMDS, ZnCl_2_, 1,4-dioxane, reflux, 2 h.

The stereoisomers of the chemical prototypes **I** and **II** were obtained according to the procedure depicted in Scheme 1. First, the coupling reaction of commercially available Boc-d-alanine or Boc-l-alanine with the benzylamine or 2-fluorobenzylamine in the presence of dicyclohexylcarbodiimide (DCC) as the coupling agent yielded the respective amide derivatives (***R***)-**1**, (***R***)-**2**, and (***S***)-**1**, (***S***)-**2**; next, removal of the Boc group in (***R***)-**1**, (***R***)-**2**, and (***S***)-**1**, (***S***)-**2** with trifluoroacetic acid (TFA) followed by neutralization with ammonium hydroxide gave amine derivatives (***R***)-**3**, (***R***)-**4**, and (***S***)-**3**, (***S***)-**4**. These intermediates were converted to corresponding succinamic acids (***R***)-**5**, (***R***)-**6**, and (***S***)-**5**, (***S***)-**6** after reaction with an equimolar amount of succinic anhydride. Next, amido-acids (***R***)-**5**, (***R***)-**6**, and (***S***)-**5**, (***S***)-**6** underwent the hexamethyldisilazane (HMDS)-promoted cyclization reaction to form the desired enantiomers **(*****R*****)-7** [**(*****R*****)-AS-1**], (***R***)-**8**, and (***S***)-**7**, (***S***)-**8** with an enantiomeric purity of >99% as determined applying a chiral high-performance liquid chromatography (HPLC) method.

It should be stressed that asymmetric synthesis was restricted to only four enantiomers of the most promising racemates previously described by our team.^28,29^ This is because we focus here on the structural optimizations aiming at the identification of lead compounds with defined stereochemistry, as well as optimal antiseizure properties and safety profiles, predominantly in the *in vivo* studies in mice.

The target enantiomers **(*****R*****)-7** [**(*****R*****)-AS-1**], (***R***)-**8**, and (***S***)-**7**, (***S***)-**8** were obtained in good yields (>80%). The intermediates and final molecules were fully characterized using ^1^H NMR, ^13^C NMR, and LC-MS spectra. Additionally, for target enantiomers, high-resolution mass spectrometry (HRMS) and elemental (C, H, N) analyses were carried out. The purity of the final compounds determined by ultra-performance liquid chromatography (UPLC) was ≥99%. The enantiomeric excess (% ee) assessed by chiral HPLC was >99%. To confirm the enantiomeric purity, the chiral HPLC resolution was performed also for the respective racemates **(*****R***, ***S*****)-7** and **(*****R***, ***S*****)-8**. For details, see the Experimental Section and the Supporting Information.

### Single-Crystal X-ray Structure Analysis

The compounds **(*****R*****)-7** [**(*****R*****)-AS-1**], **(*****S*****)-7**, **(*****R*****)-8** and **(*****S*****)-8** obtained with the asymmetric synthesis method as separated *R* and *S* enantiomers, were tested to check the samples’ enantiopurity and determine the absolute configuration. Compounds were crystallized by slow evaporation of the solvent (2-propanol) under ambient conditions. Good quality single crystals were selected randomly from the sample. The obtained X-ray diffraction data showed that the investigated crystals represented symmetry of noncentrosymmetric space groups *P*2_1_ [**(*****R*****)-7** [**(*****R*****)-AS-1**] and **(*****S*****)-7**] and *P*2_1_2_1_2_1_ [**(*****R*****)-8** and **(*****S*****)-8**], which confirmed their enantiopurity. Each pair of enantiomers was isostructural with very close unit cell dimensions. Their absolute configuration was confirmed by the anomalous scattering phenomenon. Crystal data and refinement results are shown in Table S1. All of the experimental procedures including the detailed X-ray crystallographic analyses are presented in the Supporting Information.

**Figure 2:**
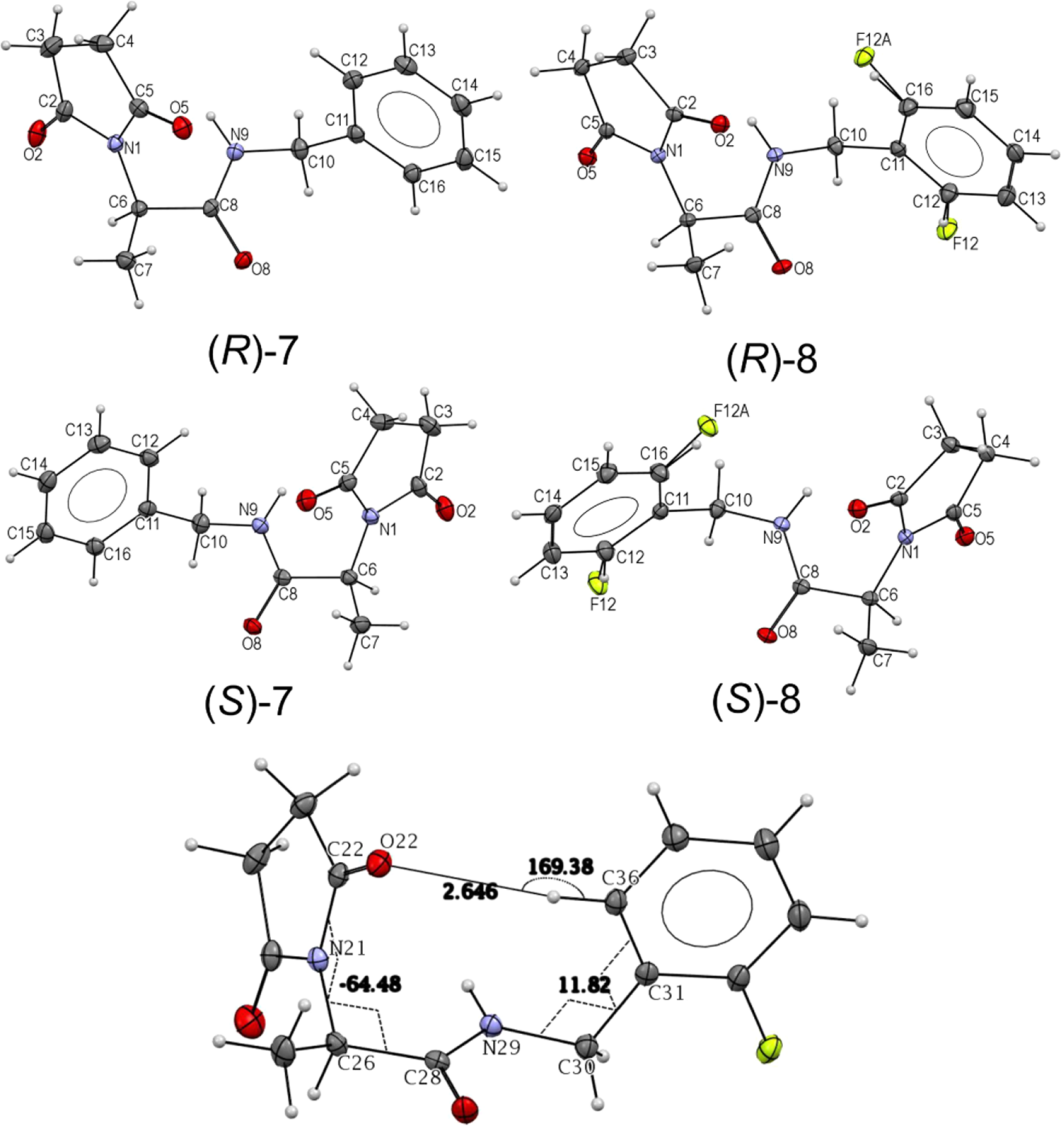
Molecular geometry observed in the crystal structure of enantiomeric pairs **(*****R*****)-7** [**(*****R*****)-AS-1**], **(*****S*****)-7** and **(*****R*****)-8**, **(*****S*****)-8** (only one molecule from the asymmetric unit is shown for figure clarity, presenting the numbering scheme). Below, the distinct molecular geometry observed in structure **(*****R*****)-8** (similar also for **(*****S*****)-8**) that results from the formation of the intramolecular hydrogen bond C36–H···O22 stabilizing the preferential conformation.

The fluorinated derivatives **(*****R*****)-8** and **(*****S*****)-8** exhibit a peculiar molecular geometry (shown in Figure 2 for **(*****R*****)-8** as an example) stabilized by the weak hydrogen bond C36–H···O22 formed between the aromatic C–H as a donor and the carbonyl oxygen of succinimide fragment as acceptor. Such a type of intramolecular weak hydrogen bond is well established in the literature.^42^ This contact can be initiated by increased acidity of C36–H, related to the presence of the electronegative F substituent in the aromatic ring, which is a known strong modulator of the C–H donor properties.^43,44^ This kind of interaction, influencing the molecular geometry, is not observed in **(*****R*****)-7** [**(*****R*****)-AS-1**] and **(*****S*****)-7** structures. Thus, the mentioned weak intramolecular interaction can favor a preferential mutual arrangement of the two ring moieties, with a plausible impact on the bioactivity of this compound.

### *In Vivo* Antiseizure Activity in Acute Seizure Models

Despite the rapid progress in both pharmaceutical and medical sciences, the efficacy and therapeutic potential of new ASD candidates are still determined by *in vivo* screening methods. Among the battery of different rodent seizure models available in preclinical studies, the MES, 6 Hz (32 or 44 mA), and *sc*PTZ remain routine tools to screen for new ASDs, due to their high clinical predictability.^45,46^

Based on the results obtained for the racemates **I** and **II**, herein we tested all enantiomers obtained, namely, **(*****R*****)-7** [**(*****R*****)-AS-1**], (***R***)-**8**, and (***S***)-**7**, (***S***)-**8** after intraperitoneal (i.p.) administration in mice (pretreatment time of 0.5 h): in the MES test, a model of generalized tonic-clonic seizures; the 6 Hz (32 mA) model of focal seizures, the 6 Hz (44 mA) model of pharmacoresistant seizures; and the *sc*PTZ model of generalized absence and myoclonic seizures. Furthermore, their influence on the motor coordination of mice was studied in the standard rotarod test. Based on the aforementioned data, the protective index (PI) which describes the benefit–risk ratio of the therapeutic agent was calculated for each seizure model (PI = TD_50_/ED_50_). The ED_50_, TD_50_, and PI values are summarized in Table 1.

The results revealed that all enantiomers synthesized herein protected mice significantly against seizures in three acute animal models of seizures, *i.e.*, MES, 6 Hz (32 mA), and *sc*PTZ, showing a wide-spectrum of anticonvulsant activity similarly to racemates **I** and **II**. Additionally, the *in vivo* data proved more beneficial antiseizure protection for the *R*-enantiomers (eutomers), **(*****R*****)-7** [**(*****R*****)-AS-1**], and (***R***)-**8** compared to the compounds with *S*-configuration (distomers), (***S***)-**7** and (***S***)-**8**, as well as racemic mixtures **I** and **II**. In the whole series, the most potent protection in each seizure model was observed for (***R***)-**8**. Despite the higher efficacy of (***R***)-**8***vs***(*****R*****)-7** [**(*****R*****)-AS-1**], the 2-fluoro-derivative was characterized by distinctly higher motor impairment in the rotarod test (TD_50_ of 224.7 mg/kg *vs* TD_50_ > 500 mg/kg, respectively), that resulted in lower safety margin expressed as PIs, predominantly in the 6 Hz (32 mA) and *sc*PTZ (*ca.* 2-fold). In the *sc*PTZ test, both **(*****R*****)-7** [**(*****R*****)-AS-1**] and **(*****R*****)**-**8** prolonged the latency time to the first seizure episode in a dose-dependent manner compared to the vehicle-treated group and statistically significant results were obtained for doses 40 mg/kg (*p* < 0.05), 60 mg/kg (*p* < 0.01) for **(*****R*****)-7** [**(*****R*****)-AS-1**], and 30 mg/kg, 40 mg/kg (*p* < 0.001 for both doses) in the case of **(*****R*****)**-**8** (Figure S1).

Notably, the 6 Hz model with 44 mA stimulus intensity has been described as a useful tool in the discovery of novel compounds with potential efficacy against pharmacoresistant seizures.^46,47^ Therefore, selected most potent enantiomers in the 6 Hz (32 mA) seizure model, *i.e.*, **(*****R*****)-7** [**(*****R*****)-AS-1**], (***S***)-**7**, and **(*****R*****)**-**8**, were tested applying stimulation intensity of 44 mA. The resulting data are summarized in Table 2.

**Table 2:** Effect of the Synthesized Compounds and Reference ASDs Administered i.p. in the 6 Hz (44 mA) Seizures in Mice^a^

compd	PT (h)^b^	ED_50_ (6 Hz, 44 mA) (mg/kg)	TD_50_ (rotarod) (mg/kg)	PI (TD_50_/ED_50_)
**(*****R*****)-7**	**0.5**	**41.6** **(32.8–52.7)**	**>500**	**>12.0**
**(*****S*****)-7**	0.5	115.1 (107.9–122.7)	>500	>4.3
**(*****R*****)-8**	0.5	37.3 (23.0–60.4)	224.7 (186.1–71.4)	6.0
**LCS^c^**	0.5	6.9 (5.4–8.6)	46.2 (44.5–48.0)	6.7
**LEV**^c^	1.0	>1000	>500	n.c.
**VPA**^c^	0.5	183.1 (143.5–233.7)	430.7 (407.9–454.9)	2.3
**CBD^d^**	1.0	173 (136–213)	272 (241–303)	1.6

a The data for the lead compound **(*R***)-**7** [**(*R*)-AS-1**] have been bolded for better visualization. Values in parentheses are 95% confidence intervals.

b Pretreatment time.

c Reference ASDs tested in the same conditions. PTs taken from own experiments or literature.^40^

d CBD data.^41^

In the 6 Hz (44 mA) seizure model, the most potent protection was demonstrated by **(*R*)**-**8**, which also revealed the strongest protection in other acute seizure tests (Table 1). Importantly, **(*****R*****)-7** [**(*****R*****)-AS-1**] showed a slightly weaker protection but showed a lack of any motor impairment at high doses like 500 mg/kg (as mentioned above) and this molecule demonstrated a 2-fold better PI compared to **(*R*)**-**8**. It should be emphasized that the 6 Hz (44 mA) model enabled distinct separation of effective doses between both enantiomers, namely, **(*R*)**-**7** [**(*R*)**-**AS-1**] and approximately a 3-fold less effective **(*****S*****)-7**. Taken together, these results confirmed the potential of **(*****R*****)-7** [**(*****R*****)-AS-1**] for the treatment of pharmacoresistant seizures.

**Figure 3:**
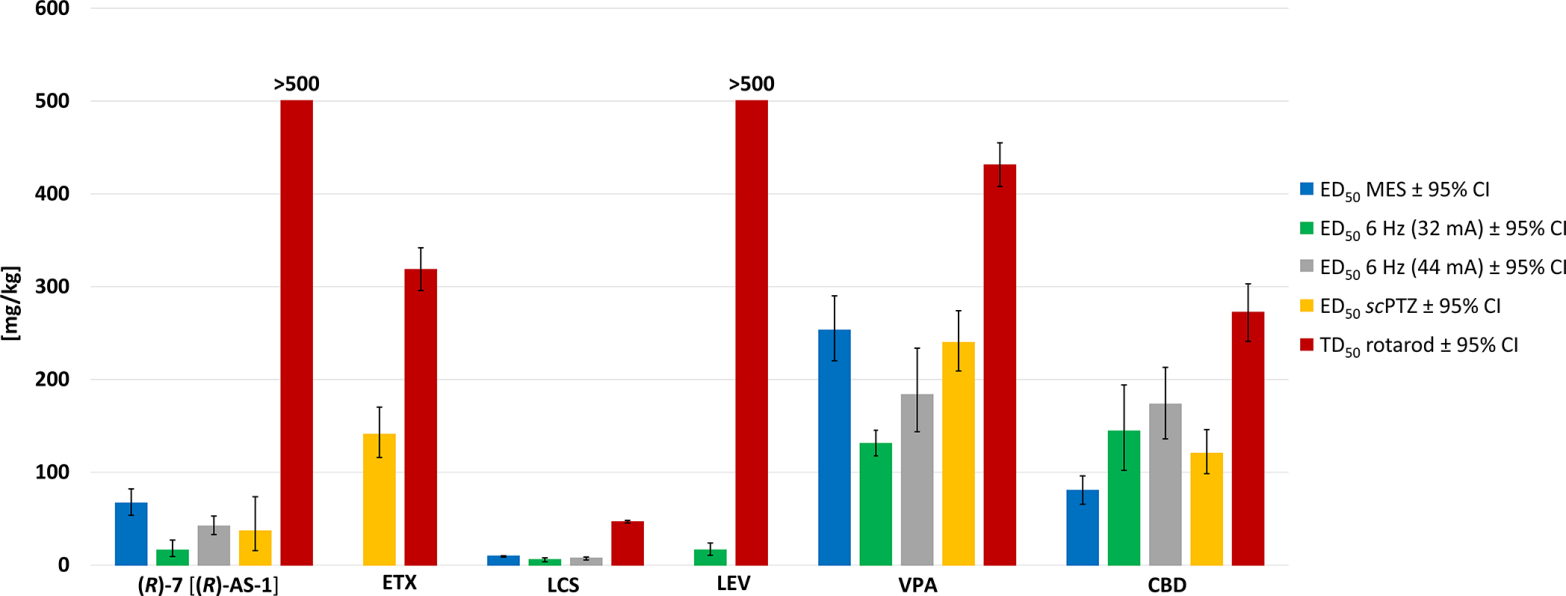
Comparison of the ED_50_ and TD_50_ values ±95% confidence intervals (95% CI) for **(*****R*****)-7** [**(*****R*****)-AS-1**] with those of the reference ASDs (ETX, LCS, LEV, VPA, and CBD), administered in mice i.p. Bars are based on data from Tables 1 and 2.

**Figure 4:**
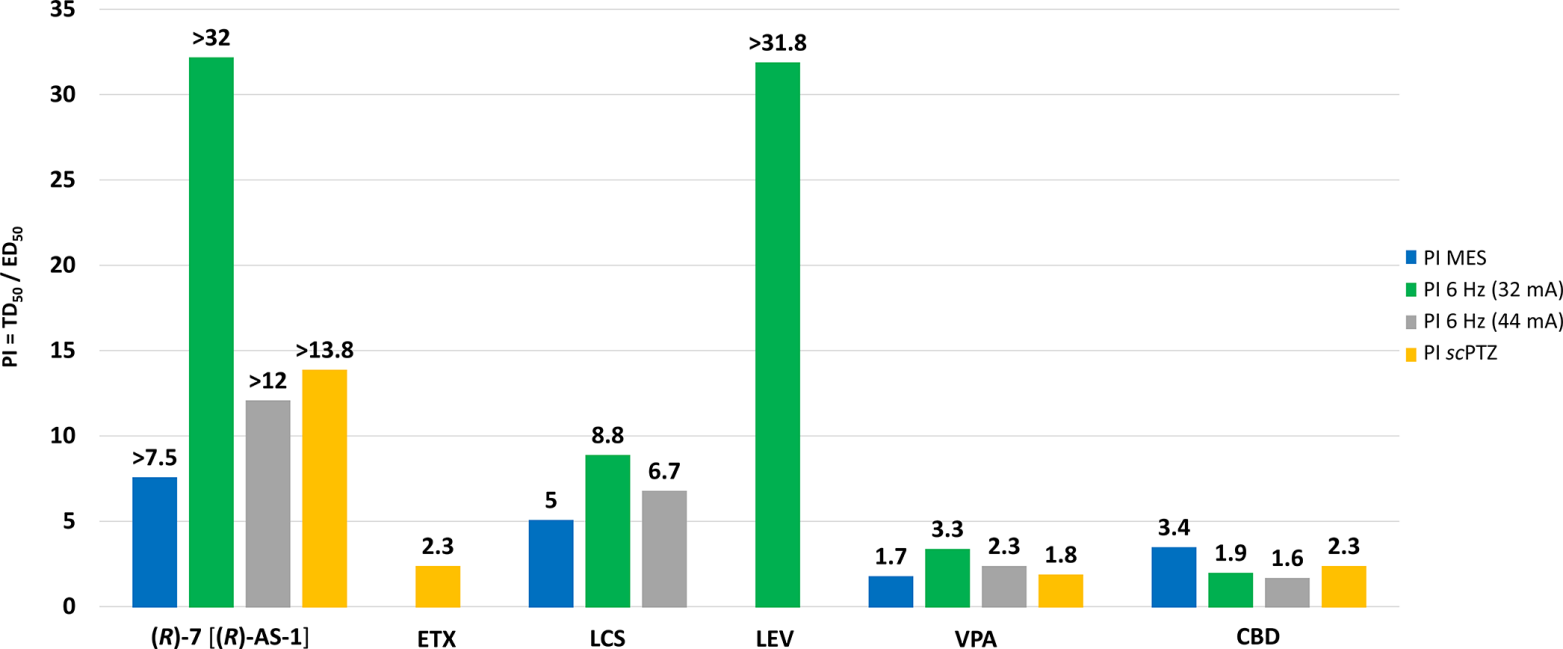
Comparison of PIs (TD_50_/ED_50_) for **(*****R*****)-7** [**(*****R*****)-AS-1**] with those of ASDs (ETX, LCS, LEV, VPA, and CBD) obtained in mice administered i.p. Bars are based on data from Tables 1 and 2.

The data obtained in the acute seizure models enable clear differentiation of the efficacy/safety profile for **(*****R*****)-7** [**(*****R*****)-AS-1**] from the reference ASDs with high therapeutic utility, *i.e.*, ETX, LCS, LEV, VPA, and CBD (Figures 3 and 4). Thus, **(*****R*****)-7** [**(*****R*****)-AS-1**] provides a much wider protection compared to ETX, LCS, and LEV. Additionally, **(*****R*****)-7** [**(*****R*****)-AS-1**] is much more effective in each seizure model compared to both CBD and VPA, which show similar, wide-spectrum protection in preclinical studies. Notably, CBD and VPA are recognized as multitarget ASDs with a high therapeutic utility in different types of epilepsy, including drug-resistant epilepsy in the case of CBD. Furthermore, **(*****R*****)-7** [**(*****R*****)-AS-1**] is characterized by an excellent safety profile in the rotarod test as it does not produce any motor impairment (TD_50_ > 500 mg/kg, similarly to LEV). Consequently, **(*****R*****)-7** [**(*****R*****)-AS-1**] provides a much better differentiation between effective and toxic doses compared to all of the aforementioned ASDs except LEV. Graphical comparisons of ED_50_, TD_50_, and PI values for **(*****R*****)-7** [**(*****R*****)-AS-1**] with those of the reference ASDs are presented in Figures 3 and 4.

Because of these very promising i.p. data, **(*****R*****)-7** [**(*****R*****)-AS-1**] was subsequently tested following oral administration. Mice were given **(*****R*****)-7** [**(*****R*****)-AS-1**] 1 h prior to testing in the same models as before (Table 3).

**Table 3:** Efficacy and Safety Profile of **(*****R*****)-7** [**(*****R*****)-AS-1**] after Oral Administration to Mice at a Pretreatment Time of 1 h^b^

ED_50_ (MES)	ED_50_ (6 Hz, 32 mA)	ED_50_ (6 Hz, 44 mA)	ED_50_ (*sc*PTZ)	TD_50_ (rotarod)	PI (TD_50_/ED_50_)
(mg/kg)	(mg/kg)	(mg/kg)	(mg/kg)	(mg/kg)	
48.6 (42.4–55.8)^a^	40.3 (33.9–47.8)	73.2 (57.4–93.4)	83.5 (65.9–105.7)	473.7 (454.7–493.4)	9.7 (MES)
					11.7 (6 Hz, 32 mA)
					6.5 (6 Hz, 44 mA)
					5.7 (*sc*PTZ)

a No mortality was observed in the MES model in test group *vs* control group (mortality *ca.* 66%).

b Values in parentheses are 95% confidence intervals.

Orally administered **(*****R*****)-7** [**(*****R*****)-AS-1**] was effective in all seizure models, demonstrating potent and broad-spectrum protection. In the *sc*PTZ test, 80 mg/kg (*p* < 0.001) and 100 mg/kg (*p* < 0.0001), **(*****R*****)-7** [**(*****R*****)-AS-1**] significantly prolonged the latency time to the first seizure episode compared to vehicle-treated group (Figure S2). Importantly, **(*****R*****)-7** [**(*****R*****)-AS-1**] produced only a minor motor impairment in the rotarod test, which resulted in very favorable PI values in the range of 5.7–11.7. Consequently, the data presented in Table 3 indicate satisfying oral bioavailability of compound **(*****R*****)-7** [**(*****R*****)-AS-1**], which can achieve an effective concentration in the CNS.

### PTZ-Induced Kindling Model in Mice

**Figure 5:**
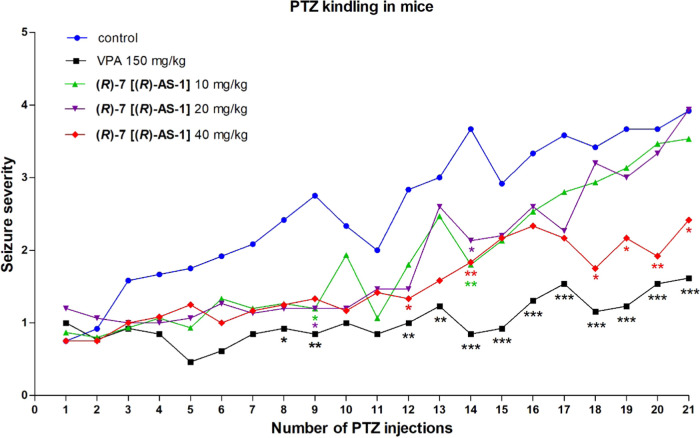
Effect of **(*****R*****)-7** [**(*****R*****)-AS-1**] on the PTZ-induced kindling progression in mice. PTZ (40 mg/kg, i.p.) was injected three times a week for a total of 21 injections. **(*****R*****)-7** [**(*****R*****)-AS-1**] and VPA were injected i.p., 30 min before PTZ injection. Data are shown as mean (*n* = 12–15 animals). The statistical significance was evaluated by repeated measures two-way ANOVA followed by the Bonferroni *post hoc* test: *p < 0.05, **p < 0.01, ***p < 0.001 compared with control group (GraphPad Prism 8.0.1).

To further characterize the antiseizure-like potency of **(*****R*****)-7** [**(*****R*****)-AS-1**], we tested its effect on the progression of epileptic seizures in the PTZ-induced kindling model of epilepsy. Repeated measures two-way analysis of variance (ANOVA) revealed a significant relationship interaction between time and treatment [*F*(80,1240) = 1.86, *p* < 0.0001] with a significant effect of both time (*p* < 0.0001) and treatment alone (*p* < 0.0001). Repetitive PTZ injection increased the seizure severity score in the PTZ control group from 0.75 ± 0.13 after the first PTZ injection to 3.92 ± 0.47 after the last PTZ injection. Bonferroni *post hoc* test showed that 10 and 20 mg/kg of (***R*****)-7** [**(*****R*****)-AS-1**] significantly decreased mean seizure severity score only after 9th and 14th PTZ injection. However, when injected at a dose of 40 mg/kg, it significantly delayed kindling progression, which was demonstrated by a significant reduction in the mean seizure severity score compared to the PTZ-kindled control group after 12th, 14th, and 18th–21st PTZ injection. The average seizure severity score for the group pretreated with **(*****R*****)-7** [**(*****R*****)-AS-1**] at 40 mg/kg was 0.75 ± 0.13 and 2.5 ± 0.5 after the first and the last PTZ injection, respectively (Figure 5). Importantly, the chemical kindling is considered a model of epileptogenesis, in which repetitive administration of PTZ at subthreshold doses gradually increases seizure susceptibility and induces permanent changes in the brain resembling those occurring in human epilepsy.^48^ Thus, the ability of **(*****R*****)-7** [**(*****R*****)-AS-1**] to suppress kindling progression suggests that this compound may produce antiepileptogenic-like effects, which prevent epilepsy development. Since **(*****R*****)-7** [**(*****R*****)-AS-1**] was administered before PTZ injection, the observed effect on kindling development was likely related to its acute antiseizure rather than antiepileptogenic action. It is worth noting that VPA also suppressed kindling development in our study, though this drug is devoid of antiepileptogenic properties in clinical practice. Consequently, a different experimental design (*e.g.*, administration of the compound after each PTZ injection or withdrawal of the drug and observation for the resumption of kindling) or use of other models of epileptogenesis (preferably those resulting in spontaneous recurrent seizures) need to be employed to evaluate the possible antiepileptogenic properties of **(*****R*****)-7** [**(*****R*****)-AS-1**].

After kindling completion, animals were subjected to behavioral tests to evaluate the possible effects of **(*****R*****)-7** [**(*****R*****)-AS-1**] on some psychiatric comorbidities in epilepsy. No significant changes in the spontaneous locomotor activity, anxiety- and depressive-like behavior in the PTZ-kindled control group (compared to the nonkindled control group) were observed. Likewise, repeated injection of **(*****R*****)-7** [**(*****R*****)-AS-1**] or VPA did not produce any significant effects (data shown in Figure S3A–D).

### Antiseizure Activity in Zebrafish

**Figure 6:**
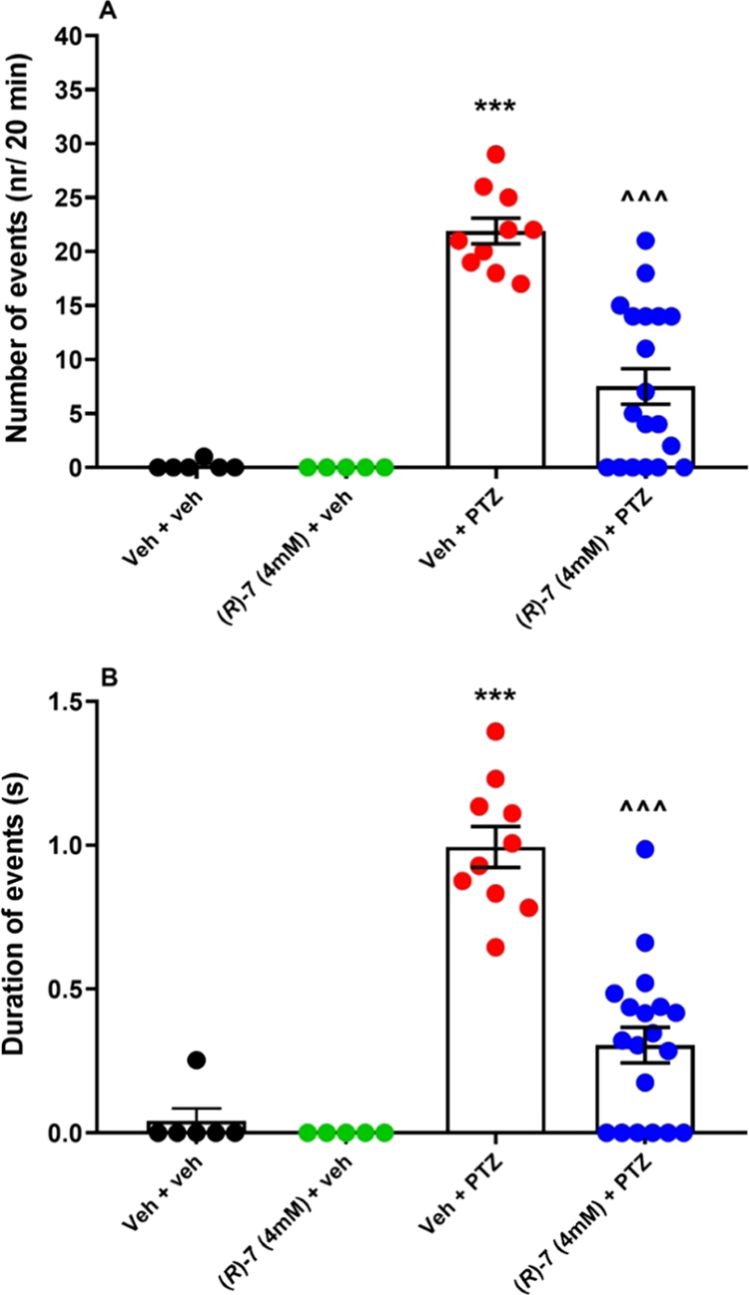
EEG events detected from the optic tectum of 7-day-old zebrafish larvae preexposed for 24 h to **(*****R*****)-7** [**(*****R*****)-AS-1**] (4 mM). Next, each larva was incubated with 20 mM PTZ or Veh for 5 min, before being mounted in agarose for EEG measurements. Data are shown as: (A) number of events (nr/20 min) and (B) mean duration of events (s/20 min). Data are shown as mean ± SEM. Veh + veh (*n* = 6), **(*****R*****)-7** [**(*****R*****)-AS-1**] 4 mM + Veh (*n* = 5), Veh + PTZ (*n* = 10), **(*****R*****)-7** [**(*****R*****)-AS-1**] 4 mM + PTZ (*n* = 19). The statistical significance was evaluated by one-way ANOVA followed by the Tukey’s *post hoc* test: ***p < 0.001 *vs* Veh + veh; ^∧∧∧^*p* < 0.001 *vs* Veh + PTZ (GraphPad Prism 8.0.1).

The PTZ-induced model of tonic-clonic seizures in zebrafish is commonly used for initial screening of potential natural^49−51^ and synthetic anticonvulsant agents.^29,52^ Larval zebrafish, when incubated in PTZ solution, experience seizures that behaviorally manifested as a hyperactivity.^49,50^ Recently, however, it was revealed that relying on the locomotor changes when assessing antiseizure activity of compounds may give false positive results.^53^ Therefore, final confirmation should be done using electroencephalographic (EEG) assay. With this in mind, we first assessed the antiseizure activity of **(*****R*****)-7** [**(*****R*****)-AS-1**] in locomotor activity test, followed by EEG analysis. To do so, larval zebrafish (6 days post-fertilization) were incubated for 24 h in different doses of **(*****R*****)-7 [(*****R*****)-AS-1**] (1, 2, or 4 mM). The PTZ (final concentration 20 mM) was applied 5 min before locomotor tracking took place. Here, we measured distance traveled within 5-min long time bins and total distance within 30 min observation period.^49^ Two-way ANOVA with repeated measures indicated the differences between tested groups of larvae [group: *F*(5,200) = 113.4, *p* < 0.001; time: *F*(5,428) = 248.5, *p* < 0.001; group × time interaction: *F*(25,1000) = 44.91, *p* < 0.001] (Figure S4A). One-way ANOVA of total distance traveled confirmed this observation [*F*(5,200) = 113.4, *p* < 0.001] (Figure S4B). Next, the zebrafish larvae were incubated in the highest concentration of **(*****R*****)-7** [**(*****R*****)-AS-1**] *i.e.*, 4 mM for 24 h and exposed to PTZ for 5 min before EEG assay started. Here, one way ANOVA revealed the differences between groups in terms of number of discharges [*F*(3,37) = 28.69, *p* < 0.001] and duration of events [*F*(3,37) = 43.19, *p* < 0.001] (Figure 6). *Post hoc* analysis indicated that **(*****R*****)-7** [**(*****R*****)-AS-1**] pretreated zebrafish which were subsequently exposed to PTZ exhibited a lower number of events (*p* < 0.001) and a shorter duration of events (*p* < 0.001), compared to only PTZ-treated larvae.

### Spontaneous Locomotor Activity Test

**Figure 7:**
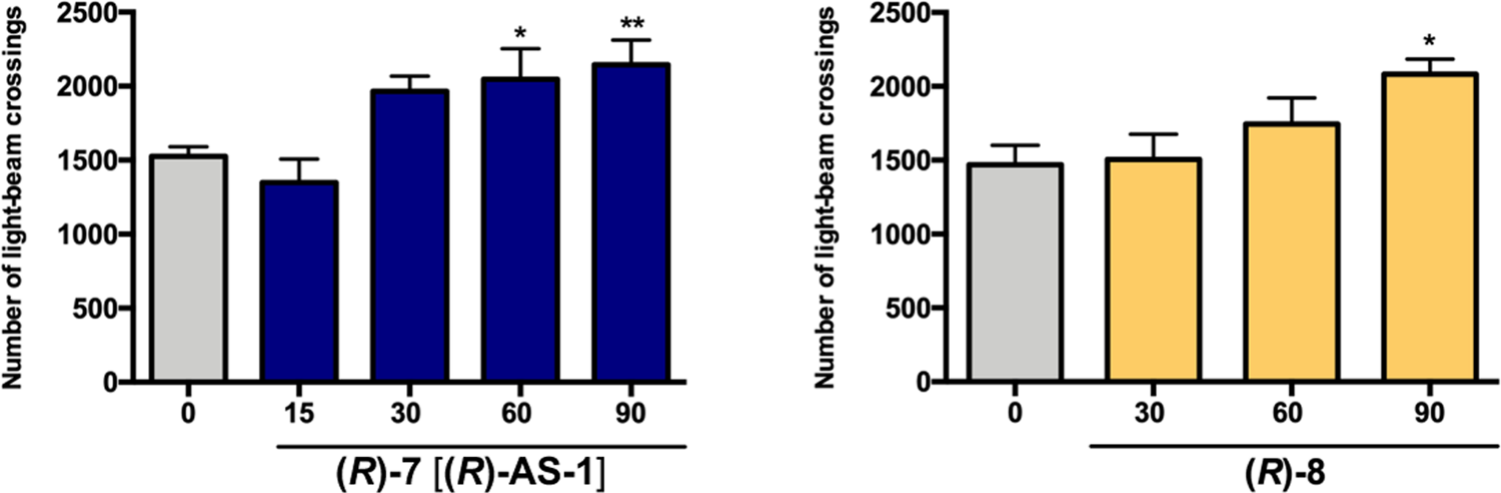
Influence of **(*****R*****)-7** [**(*****R*****)-AS-1**] and **(*****R*****)-8** on spontaneous locomotor activity of mice. Results are shown as the number of light beam crossings during 30 min of observation beginning at 30 min after i.p. administration. The vehicle-treated group received 1% water solution of Tween 80. Data are shown as mean ± SEM (*n* = 8–10 animals). The statistical significance was evaluated using one-way ANOVA with Dunnett’s *post hoc* test: *p < 0.05, **p < 0.01 (GraphPad Prism 8.0.1).

One of the most prevalent CNS adverse effects observed during ASD therapy is sedation. This side effect occurs with most of the commonly used ASDs, including the newest treatment options, *i.e.*, LCS, LEV, *etc.*(4,54−56) Therefore, the potential of **(*****R*****)-7** [**(*****R*****)-AS-1**] to induce sedation was evaluated in the spontaneous locomotor activity test (Figure 7 and Table S2). Only the lowest dose of 15 mg/kg slightly decreased locomotor activity to 88.4% (effect not statistically significant), whereas higher doses, namely, 30, 60, and 90 mg/kg dose-dependently increased the spontaneous locomotor activity to the value of 128.8, 134.1, and 140.5% of the control group, respectively. To confirm these unexpected results, similar studies were carried out for the 2-fluoro analogue **(*****R*****)-8**. Similarly, **(*****R*****)-8** injected at doses of 30, 60, and 90 mg/kg increased mice locomotor activity in a dose-dependent manner reaching statistical significance at 90 mg/kg. Thus, the data reported herein proved that both compounds do not cause any sedative effects. Moreover, the higher doses such as 60 and 90 mg/kg of **(*****R*****)-7** [**(*****R*****)-AS-1**] and 90 mg/kg of **(*****R*****)-8** have a moderate but significant stimulating impact on spontaneous locomotor activity in mice, which contrasts with the similar data for standard and commonly used antiepileptic drugs, *e.g.*, VPA, gabapentin, or pregabalin. This kind of pharmacological profile may be potentially beneficial in clinical practice, as sedation related to ASDs-pharmacotherapy has significant negative effects on the patients’ quality of life.

In summary, these results identify **(*****R*****)-7** [**(*****R*****)-AS-1**] as the lead compound with good oral bioavailability, potent and broad-spectrum antiseizure activity (both in acute and chronic seizure models), a very favorable safety/tolerability profile, not accompanied by sedation (in contrast to majority commonly used ASDs).

### Characterization of the Mechanism of Action

The structures of chemical prototypes **I** and **II** (Figure 1) of the enantiomers reported here were designed as hybrid compounds by a combination of chemical fragments which are similar to ETX (*i.e.*, pyrrolidine-2,5-dione), LCS (*i.e.*, benzylamide), and LEV (*i.e.*, pyrrolidine-2-one incorporated in the succinimide ring and alkylamide fragment).^28^ Both **I** and **II**, but especially **(*****R*****)-7** [**(*****R*****)-AS-1**], revealed a broader spectrum of antiseizure efficacy compared to each of these individual drugs, *i.e.*, ETX (PTZ-protection), LCS [MES, 6 Hz (32/44 mA)-protection], and LEV [6 Hz (32 mA)-protection], indicating a possible multimodal or unique mechanism of action. Because of this, we carried out binding and electrophysiological studies to determine if **(*****R*****)-7** [**(*****R*****)-AS-1**] acted through a molecular target previously reported for those aforementioned ASDs, *i.e.*, low-voltage activated calcium channels Ca_v_3.2 (ETX); TTX-sensitive sodium channels (LCS); and synaptic vesicle glycoprotein 2A (SV2A) (LEV). Surprisingly, the obtained results showed no interaction with calcium and sodium ion channels in a high concentration of 100 μM (Tables S3 and S4), and negligible (*K*_i_ > 250 μM) or no binding to human and mouse SV2A (Figure S5). Additionally, there were no distinct differences in binding to SV2A for both enantiomers, which makes this mechanism of action very unlikely, as other ASDs show clear stereoselective bioactivity, such as LEV (*S*-enantiomer *vsR*-enantiomer^26^). Furthermore, it should be stressed that **I**, which is the racemic mixture of **(*****R*****)-7** [**(*****R*****)-AS-1**] and **(*****S*****)-7**, did not affect sodium currents in rat prefrontal cortex pyramidal neurons by the patch-clamp technique.^29^ Thus, **(*****R*****)-7** [**(*****R*****)-AS-1**] does not seem to share any mechanistic similarities to ETX, LCS, and LEV, despite their shared chemical prototype.

Consequently, as a next step for elucidating the mechanism of action, we tested the interaction of **(*****R*****)-7** [**(*****R*****)-AS-1**] in a broader panel of other ion channels, ionotropic/metabotropic receptors, transporters, or enzymes responsible for the activity of known ASDs or ASD candidates (*i.e.*, Ca_v_2.2, GABA_A_R, AMPAR, NMDAR, sigmaR_1_, GlyR_A1_, caspase 1, GAT-1 and GAT-3, *etc.*),^4^ as well as other molecular targets not involved directly in seizure induction or spread, *i.e.*, monoamine transporters such as serotonin transporter (SERT), dopamine transporter (DAT) and norepinephrine transporter (NET) which have been pointed out to possibly serve as targets for epilepsy treatment^57,58^ (Tables S4–S6). We have found that **(*****R*****)-7** [**(*****R*****)-AS-1**] was inactive in all of the aforementioned *in vitro* assays at 10, 50, and/or 100 μM. Notably, there was no interaction with the hERG channel, which is recognized as a key off-target tested in the early stage of drug development. Furthermore, the patch-clamp recordings in slices of the prefrontal cortex showed that **(*****R*****)-7** [**(*****R*****)-AS-1**] did not influence the tonic NMDA currents mediated by both synaptic and extrasynaptic NMDA receptors at a high concentration of 100 μM. For details, see Figure S6.

The structural investigation has revealed several structural similarities of **(*****R*****)-7** [**(*****R*****)-AS-1**] to (*R*)-3-[(4-cyclohexyl-1-piperazinyl)[1-(2-phenylethyl)-1*H*-tetrazol-5-yl]methyl]-6-methoxy-2(1*H*)-quinolinone [(*R*)-GT949], which is a potent and selective PAM of the EAAT2 glutamate transporter.^59,60^ These similarities include bioisosteric replacement of *i.e.*, 5,6-dihydropyridin-2(1H)-one ring ([(*R*)-GT949]) into pyrrolidine-2,5-dione in **(*****R*****)-7** [**(*****R*****)-AS-1**]; tetrazole moiety ([(*R*)-GT949]) into amide fragment of **(*****R*****)-7** [**(*****R*****)-AS-1**], as well as exchange of phenylethyl substituent ([(*R*)-GT949]) to benzyl in **(*****R*****)-7** [**(*****R*****)-AS-1**]. Thus, to examine the hypothesis that the enantiomers **(*****R*****)-7** [**(*****R*****)-AS-1**] and **(*****R*****)-8** could potentially act as PAMs of glutamate transporters, we performed molecular docking and glutamate transport studies for both molecules.

### Structural Modeling of EAAT2 and Prediction of **(*R*)-7** [**(*R*)-AS-1**] and **(*R*)-8** Binding Sites onto EAAT2

Previous studies of the three-dimensional structure of EAAT family members revealed that the transporter is a trimer, each monomer being composed of two domains: a transport domain containing the substrate binding sites with structural elements essential to substrate uptake and release, and a peripheral rigid scaffold, designated as the trimerization domain.^61,62^ The trimerization domain provides the interaction interface between the three monomers, while the transport domain undergoes a concerted elevator-like movement along with local gating events when the transporter reconfigures between its outward-facing (OF) and inward-facing (IF) states.^63−66^ We generated structural models for human EAAT2 (residues K43 to D505; UniProt ID P43004) trimer in the OF and IF states using SWISS-MODEL^67^ based on the structures resolved for the symmetric OF EAAT1^64^ (PDB: 5LLU) and IF EAAT3^68^ (PDB: 6X2L). In addition, an asymmetric hEAAT2 trimer model (containing one subunit in OF and two in IF conformations) was constructed based on the asymmetric EAAT3^68^ trimer (PDB: 6X3E).

**Figure 8:**
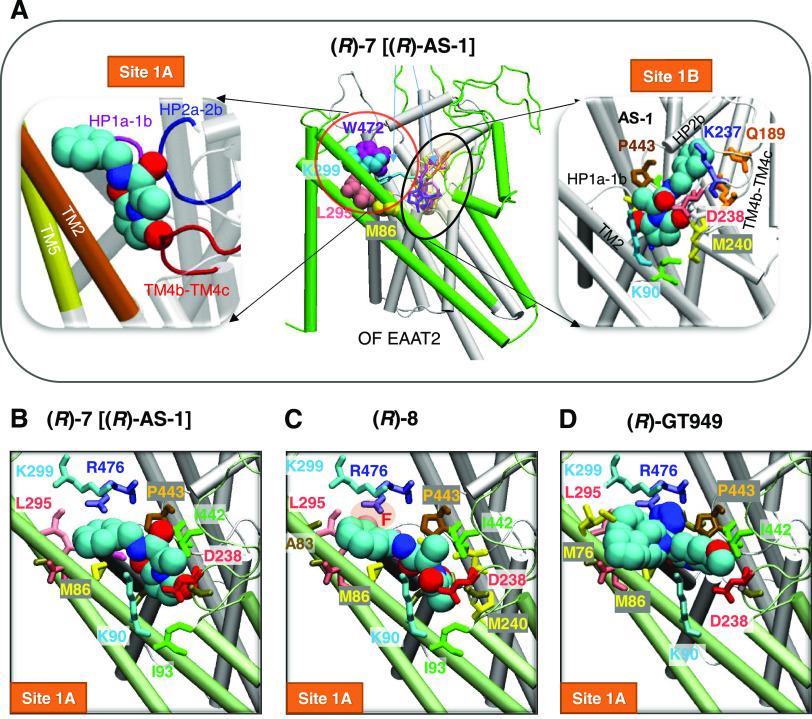
**(*****R*****)-7** [(***R*)-AS-1**] and **(*R*)-8** bind at the interface between the scaffold domain (green) and the transport domain (white) of human EAAT2 in the OF state. (A) Binding poses of **(*R*)-7** [**(*R*)-AS-1**] to the two most favorable sites site 1A and site 1B. The diagram in the center shows the multiple binding poses of **(*****R*****)-7[(*****R*****)-AS-1**] onto OF EAAT2, shown in sticks of different colors. Residues reported earlier to coordinate the binding of PAM (*R*)-GT949^60^ are displayed in van der Waals (VDW) representation in different colors (middle). Site 1A pocket is lined by TM2 (orange) and TM5 (yellow), and the three loops TM4b-4c (red), HP1a-1b (purple), and HP2a-2b (blue) (left) and site 1B pocket is lined by residues from TM2, TM4b-4c, HP1a-1b and HP2 (right). Site 1A and site 1B, shown on the left and right panels, are predicted to show equal affinity (−7.3 kcal/mol). (B–D) Comparison of the predicted binding poses of **(*****R*****)-7** [**(*****R*****)-AS-1**] (B), **(*****R*****)-8** (C), and (*R*)-GT949 (D) onto site 1A. AutoDock predicts the respective binding affinities of the three compounds to site 1A as −7.3, −7.6, and −8.0 kcal/mol. The EAAT2 protein was modeled after the EAAT1 OF conformer (PDB: 5LLU).

We performed molecular docking simulations to explore whether **(*****R*****)-7** [**(*****R*****)-AS-1**] or **(*****R*****)-8** could bind onto EAAT2, and if so to determine their binding site(s) and affinities, as well as the dependency on the conformational state of EAAT2. Our simulations revealed that both compounds could bind to EAAT2, and the OF conformer was found to lead to most favorable intermolecular interactions in general. The highest affinity binding site for either ligand was an extracellularly exposed interfacial region between the scaffold and transport domains of each monomer. Figure 8A illustrates the top-ranking binding poses and sites for **(*****R*****)-7** [**(*****R*****)-AS-1**] observed in our simulations. Mainly, **(*****R*****)-7** [**(*****R*****)-AS-1**] is predicted to preferentially occupy two binding sites, sites 1A and 1B.

Residues coordinating the binding of **(*****R*****)-7** [**(*****R*****)-AS-1**] onto site 1A include G82, D83, M86, K90 and I93 on TM2, D238–G239 on TM4b-4c loop, L295 and K299 on TM5, G360–G366 (HP1 loop), I442–P443 (HP2 loop) and R476 (TM8) (see Figure 8A,B). The corresponding binding affinity is −7.3 ± 0.3 kcal/mol. Molecular interactions stabilizing the bound form include a cation−π interaction between K299/R476 and the benzene ring of the compounds, strong hydrophobic interactions involving M83, I93, L295, I442, and P443, and hydrogen bonds between the pyrrolidine-2,5-dione ring and K90 and D238.

Notably, site 1A coincides with the binding site for (*R*)-GT949, a PAM of EAAT2 (see Figure 8D) reported in a previous study.^60^ Residues K299, M86, and L295, reported therein to participate in the allosteric modulation of EAAT2 by (*R*)-GT949,^60^ are found here to coordinate **(*****R*****)-7** [**(*****R*****)-AS-1**] and **(*****R*****)-8** (Figure 8B,C).

In addition to site 1A, **(*****R*****)-7** [**(*****R*****)-AS-1**] could bind to an additional site (site 1B) (Figure 8A, right) with comparable binding affinity. Residues coordinating the binding of **(*****R*****)-7** [**(*****R*****)-AS-1**] to site 1B include K90 and I93 from TM2, Q187–Q189, and K237–V242 from TM4b-4c loop, T361–G366 from HP1, and I442–V448 from HP2. Notably, in both poses, the pyrrolidine-2,5-dione ring from **(*****R*****)-7** [**(*****R*****)-AS-1**] binds to the same region of the transporter. We suggest that the **(*****R*****)-7** [**(*****R*****)-AS-1**] pyrrolidine-2,5-dione ring may determine its binding affinity and selectivity to EAAT2 over other EAATs, since K90, as well as several residues from the TM4b-4c loop (*e.g.*, K237, D238, and M240) are specific to EAAT2.

We also performed docking simulations for binding **(*****R*****)-8** and PAM (*R*)-GT949 onto EAAT2. The computations revealed both ligands selected the same binding sites as **(*****R*****)-7** [**(*****R*****)-AS-1**] (not shown). AutoDock yielded a slightly higher affinity (−7.6 ± 0.3 kcal/mol) for binding **(*****R*****)-8** to site 1A, compared **(*****R*****)-7** [**(*****R*****)-AS-1**] (−7.3 ± 0.3 kcal/mol). In addition to the favorable cation-π interactions, hydrophobic interactions, and hydrogen bonds, which were observed for binding **(*****R*****)-7** [**(*****R*****)-AS-1**], we noted that additional interactions could promote the binding of **(*****R*****)-8**. In particular, the fluorine atom in the benzene ring of **(*****R*****)-8** may increase the electrostatic interaction with the positively charged R476 due to the electronegativity of fluorine (yellow circle in Figure 8C), and the larger size of **(*****R*****)-8** promoted closer hydrophobic contacts with A83 and M240 according to the model.

Finally, to study the effects of stereochemistry, we performed additional docking simulations of **(*****S*****)-7** and **(*****S*****)-8** onto EAAT2. Of note, both **(*****S*****)-(7)** and **(*****S*****)-8** exhibit reduced binding affinities (by at least 1 kcal/mol) to the OF EAAT2, compared with their respective *R*-enantiomers (see Figure S7). Furthermore, *S*-enantiomers do not target those residues, *e.g.*, M86 and L295, reported earlier to coordinate the binding of PAM (*R*)-GT949.^60^ Interestingly, mutations of the homologous counterparts of M86 and L295 in EAAT1 were found to shift the transporter function and substrate uptake rate.^69^ Collectively, we suggest that the *S*-enantiomers have lower potency for targeting EAAT2 than their *R*-enantiomers.

### Glutamate Uptake Studies in COS-7 Cell Lines Mediated by EAATs and in Primary Glia Cells

**Figure 9:**
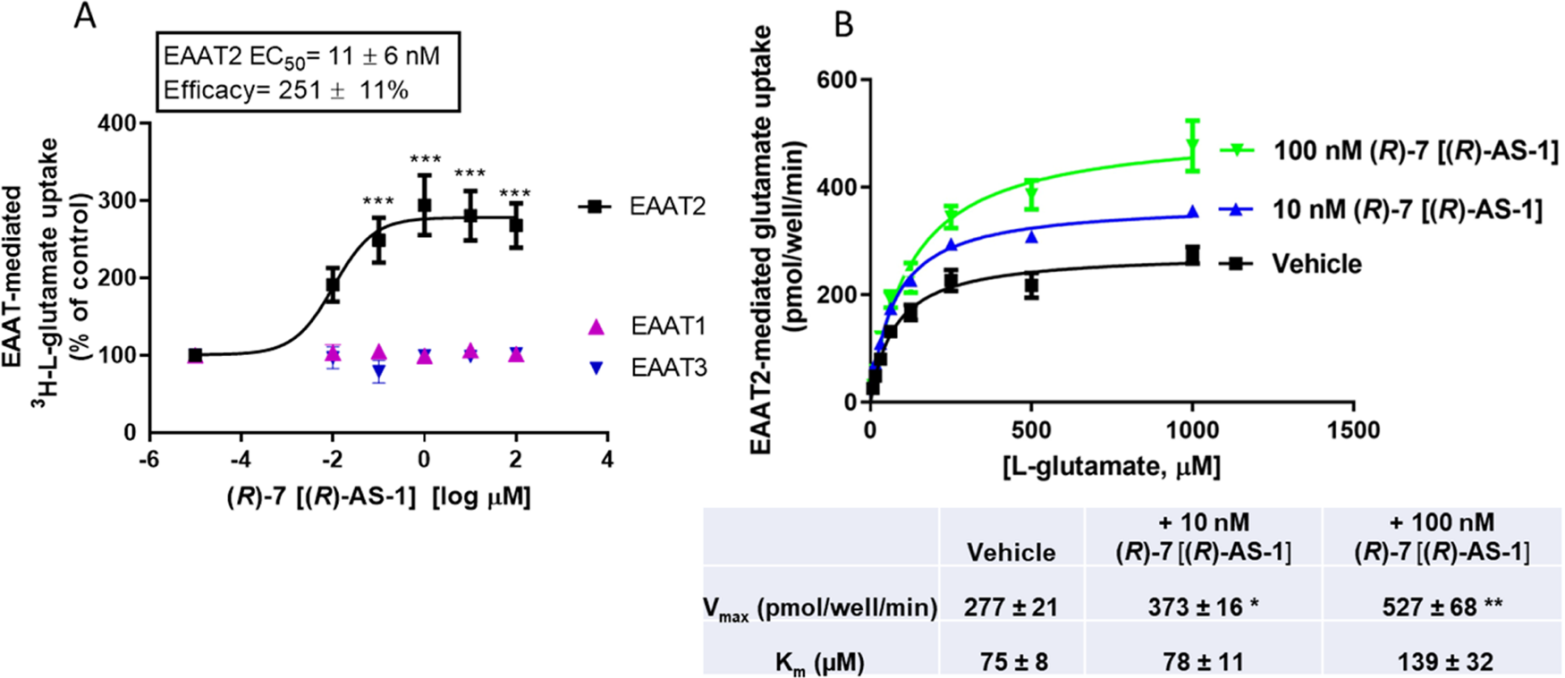
(A) **(*****R*****)-7** [**(*****R*****)-AS-1**] augments glutamate uptake mediated by EAAT2 (but not EAAT1 or 3) in transfected COS-7 cells (***p < 0.001 compared to vehicle). (B) Kinetic analysis of glutamate uptake in the presence of 10 (blue) or 100 nM (green) **(*****R*****)-7** [**(*****R*****)-AS-1**]. The table underneath shows *V*_max_ and *K*_m_ values obtained from four independent experiments performed in triplicate (*p < 0.05 and **p < 0.01 for *V*_max_ of compound compared to vehicle). ANOVA followed by Dunnet’s *post hoc* test, averages of triplicate determinations of four independent experiments ± SEM. *K*_m_ was not statistically different among groups (GraphPad Prism 9).

Our results indicate that **(*****R*****)-7** [**(*****R*****)-AS-1**] stimulated l-glutamate uptake through EAAT2 in COS-7 cell lines with an EC_50_ of 11 ± 6 nM and an efficacy of augmentation of 251 ± 11% (Figure 9A), with no effects on EAAT1 and EAAT3-mediated uptake. A kinetic analysis of glutamate uptake in the presence of 10 nM (blue) or 100 nM (green) **(*****R*****)-7** [**(*****R*****)-AS-1**] revealed significant increases in the *V*_max_ values, by ∼135 and ∼190%, respectively, while *K*_m_ values remained relatively stable among groups, *i.e.*, without a statistically significant difference (Figure 9B).

**Figure 10:**
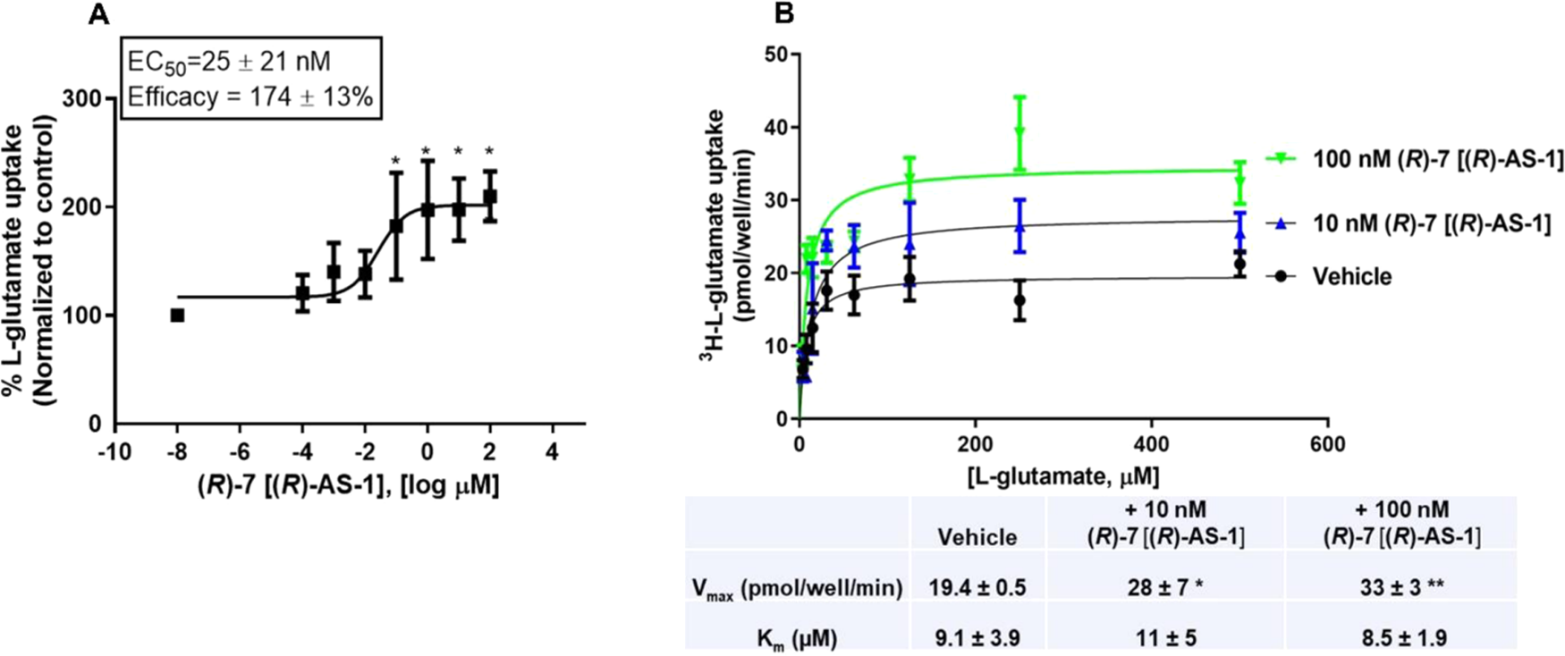
(A) **(*****R*****)-7** [**(*****R*****)-AS-1**] augments glutamate uptake in cultured glia cells (*p < 0.05 compared to vehicle). (B) Kinetic analysis of glutamate uptake in the presence of 10 nM (blue) or 100 nM (green) **(*****R*****)-7** [**(*****R*****)-AS-1**]. The table underneath shows *V*_max_ and *K*_m_ values obtained from four independent experiments performed in triplicate; (*p < 0.05 and **p < 0.01 for *V*_max_ of compound compared to vehicle). ANOVA followed by Dunnet’s *post hoc* test, averages of triplicate determinations of four independent experiments ± SEM. *K*_m_ was not statistically different among groups (GraphPad Prism 9).

To validate the effect of **(*****R*****)-7** [**(*****R*****)-AS-1**] in a more physiologically relevant assay, we performed dose–response assays in cultured glia (Figure 10A). The compound again showed a potency of 25 ± 21 nM and efficacy of 174 ± 13% for l-glutamate uptake augmentation, which was comparable to the values obtained in the heterologous expression system (COS-7 cells). Kinetic analysis of l-glutamate uptake in the presence of different concentrations of the compound is shown in Figure 10B. *V*_max_ values significantly increased by ∼147 and ∼174% in the presence of 10 and 100 nM compound, respectively. Similarly to the results obtained in the transfected COS-7 cells, the *K*_m_ values did not exhibit significant differences under different conditions.

These results demonstrate that **(*****R*****)-7** [**(*****R*****)-AS-1**] acts as a selective enhancer of the activity of EAAT2. The affinity for the substrate (*K*_m_) was not statistically different when measured in the presence of the compound, suggesting that **(*****R*****)-7** [**(*****R*****)-AS-1**] enhances the glutamate translocation rate, with no effect on substrate interaction, suggesting an allosteric mechanism.

We have also investigated the effects of **(*****R*****)-8** on transfected COS-7 cells and found that this compound also acts as an EAAT2 PAM, however, with a slightly lower potency (EC_50_ = 20 ± 11 nM) and efficacy (166 ± 13%) of augmentation of EAAT2-mediated glutamate transport, compared to **(*****R*****)-7** [**(*****R*****)-AS-1**] (Figure S8A). Notably, studies in cultured glia cells **(*****R*****)-8** revealed a more potent effect (with an EC_50_ of 0.1 ± 0.3 nM, Figure S8B) than what was observed for **(*****R*****)-7** [**(*****R*****)-AS-1**] (∼20 nM) with a similar efficacy (156 ± 28%) of glutamate transport augmentation. This higher potency correlates with the more potent protection of **(*****R*****)-8***vs***(*****R*****)-7** [**(*****R*****)-AS-1**] observed in the *in vivo* seizure models (Tables 1 and 2) since the glia cultures are a more physiological approach than transfected cells. Another potential explanation is that both aforementioned compounds could have also different pharmacokinetic properties/exposure or additional mechanisms and targets that have not been investigated thus far.

Our previous work suggested that residues at the interface between the transport and the trimerization domains are critical for transport stimulation,^70^ as well as coordination of substrate transport and chloride channeling.^69^ Our proposed mechanism of interaction between compound **(*****R*****)-7** [**(*****R*****)-AS-1**] and EAAT2 included the hypothesis that the compound might be binding to the site previously identified to bind allosteric modulators.^70^ Current simulations confirmed that both **(*****R*****)-7** [**(*****R*****)-AS-1**] and **(*****R*****)-8** bind to a high-affinity site, site 1A, at the interface between the transport and trimerization domains of the monomers. The binding of small molecules at this interfacial region may possibly accelerate the rate of transport by facilitating the movement of the transport domain.^59,60^

With the aim of assessing the influence of stereochemistry on glutamate uptake mediated by EAAT2, we carried out similar studies in COS-7 cell lines for both *S*-enantiomers, namely, **(*****S*****)-7** and **(*****S*****)-8** (Figure S9). Our results illustrate that the *S*-enantiomers displayed an enhancing effect on glutamate uptake in EAAT2-transfected cells only at a very high concentration of 1 mM, as opposed to the *R*-enantiomers that showed a much more potent effect (EC_50_ for **(*****R*****)-7** [**(*****R*****)-AS-1**] is 11 ± 6 nM, as shown in Figure 9A and for **(*****R*****)-8** EC_50_ is 20 ± 11 nM, as shown in Figure S8A). These data suggest that the *S*-enantiomers have low potency, with EC_50_ higher than 100 μM. Data shown in Figure S9 indicate that the *R*-enantiomers display an inverted dose-effect curve. Other allosteric compounds have been shown to behave in this manner, as exemplified by PAMs of dopamine D1 receptors^71^ (a mechanism suggested as a potential treatment of neuropsychiatric disorders) and PAMs of mGluR5^72^ (suggested as potential cognitive enhancers).

In summary, *in vitro* glutamate transport studies suggest that **(*****R*****)-7** [**(*****R*****)-AS-1**] and **(*****R*****)-8** act as selective PAMs of EAAT2, and *in silico* studies reveal the specific mechanism of binding at the interface between the transport and trimerization domains of each monomer, which shows a particularly high avidity when EAAT2 is in its OF conformation. Until now, such an allosteric mechanism of action has not been identified for small-molecule amino acid derivatives with potent antiseizure activity *in vivo*. Furthermore, until now, to the best of our knowledge, other PAM compounds selectively acting on EAAT2 have not been tested as a potential epilepsy therapy.^4^

### *In Vitro* ADME-Tox Assays

Poor ADME-Tox parameters can preclude each compound from becoming a clinical candidate. Therefore, early identification of potential limitations identified in ADME-Tox studies is an essential step in drug development. For this purpose, selected *in vitro* assays were performed for **(*****R*****)-7** [**(*****R*****)-AS-1**]. The obtained results are summarized and shown in Table 4.

**Table 4:** ADME-Tox Parameters Determined *In Vitro* for **(*****R*****)-7** [**(*****R*****)-AS-1**]^a^

permeability/plasma protein binding	
PAMPA—Pe (10^–6^ cm/s) ± SD	1.98 ± 0.6
plasma protein binding—fraction bound *f*_b_ (% ± SD)	23.8 ± 3.7
plasma protein binding—*K*_D_ (μM)	1970.0

metabolic pathways in human	
phase I	no metabolites found
phase II (glucuronidation)	no metabolites found

metabolic pathways in mouse	
phase I	no metabolites found
phase II (glucuronidation)	not tested

CYPs safety profile	
CYP3A4 activity (% of control ± SD at 10 μM)	100.4 ± 4.2
CYP2D6 activity (% of control ± SD at 10 μM)	105.3 ± 1.0
CYP2C9 activity (% of control ± SD at 10 μM)	67.9 ± 3.6

cytotoxicity assay	
HepG2 viability (% of control ± SD at 100 μM)	94.3 ± 7.1
HEK-293 viability (% of control ± SD at 100 μM)	96.8 ± 21.9
SH-SY5Y viability (% of control ± SD at 10 μM)	127.33 ± 7.3

a Abbreviations: Pe—permeability coefficient.

PAMPA results showed satisfactory ability for passive diffusion with a permeability (Pe) value higher than the breakpoint for permeable compounds according to the manufacturer’s guideline (Pe ≥ 1.5 × 10^–6^ cm/s).^73^

Regarding the distribution parameter, **(*****R*****)-7** [**(*****R*****)-AS-1**] showed low plasma protein binding compared to the positive, highly bound reference warfarin^74^ (23.8 and 98.5% fraction bound, respectively).

Previously, racemate **I** was shown to be very stable metabolically in human liver microsomes (HLMs).^29^ Therefore, it was not surprising that **(*****R*****)-7** [**(*****R*****)-AS-1**] is also a highly metabolically stable compound. No metabolites were found in the presence of HLMs or mouse liver microsomes (MLMs) (Table 4 and Figure S10A,B). The additional investigation of glucuronide phase II metabolism of **(*****R*****)-7** [**(*****R*****)-AS-1**] by HLMs was performed here. The obtained UPLC chromatogram did not confirm the presence of any glucuronide metabolite of **(*****R*****)-7** [**(*****R*****)-AS-1**] (Table 4 and Figure S11A).

No significant differences between **(*****R*****)-7** [**(*****R*****)-AS-1**] and racemate **I** were found in drug–drug interaction studies. **(*****R*****)-7** [**(*****R*****)-AS-1**] showed slight inhibition of CYP3A4 and slight activation of CYP2D6 only at the highest concentration of 25 μM. A moderate inhibitory effect, comparable to that of the racemate, was shown in the CYP2C9 assay (Table 4 and Figure S12A–C).

In our previous studies,^29,37^ the slight effect of racemate **I** on the viability of hepatoma HepG2 cells was observed at a high concentration of 100 μM. In the present study, a similar protocol was applied for **(*****R*****)-7** [**(*****R*****)-AS-1**] as well as the reference ASDs (LEV, LCS, ETX, and VPA) for 72 h. None of the compounds, **(*****R*****)-7** [**(*****R*****)-AS-1**], and ASDs, showed any effect on HepG2 cells, suggesting they may be safe for long-term use (Table 4 and Figure S13A,B). Moreover, the safety of **(*****R*****)-7** [**(*****R*****)-AS-1**] was also confirmed after incubation with human embryonic kidney (HEK-293) cells for 72 h (Table 4 and Figure S14).

In the neurotoxicity assay, an interesting neurogenic effect of compound **(*****R*****)-7** [**(*****R*****)-AS-1**] was observed after 72 h of incubation of the neuroblastoma SH-SY5Y cells. The statistically significant induction (up to 130% of control) of the cells’ viability was observed in all tested concentrations from 0.0001 to 10 μM (Table 4 and Figure S15A). Consistently, an apparent and comparable neurogenesis effect has already been described for other pyrrolidine-2,5-dione derivatives synthesized by our research group.^36,75^ A similar effect on SH-SY5Y cells was observed only for LEV at the highest dose of 100 μM (Table 4 and Figure S15B). Additionally, the *in vitro*/*in vivo* stimulation of neuronal cell viability by this ASD has been reported in several recent articles.^76−78^

### Pharmacokinetic Studies

**Figure 11:**
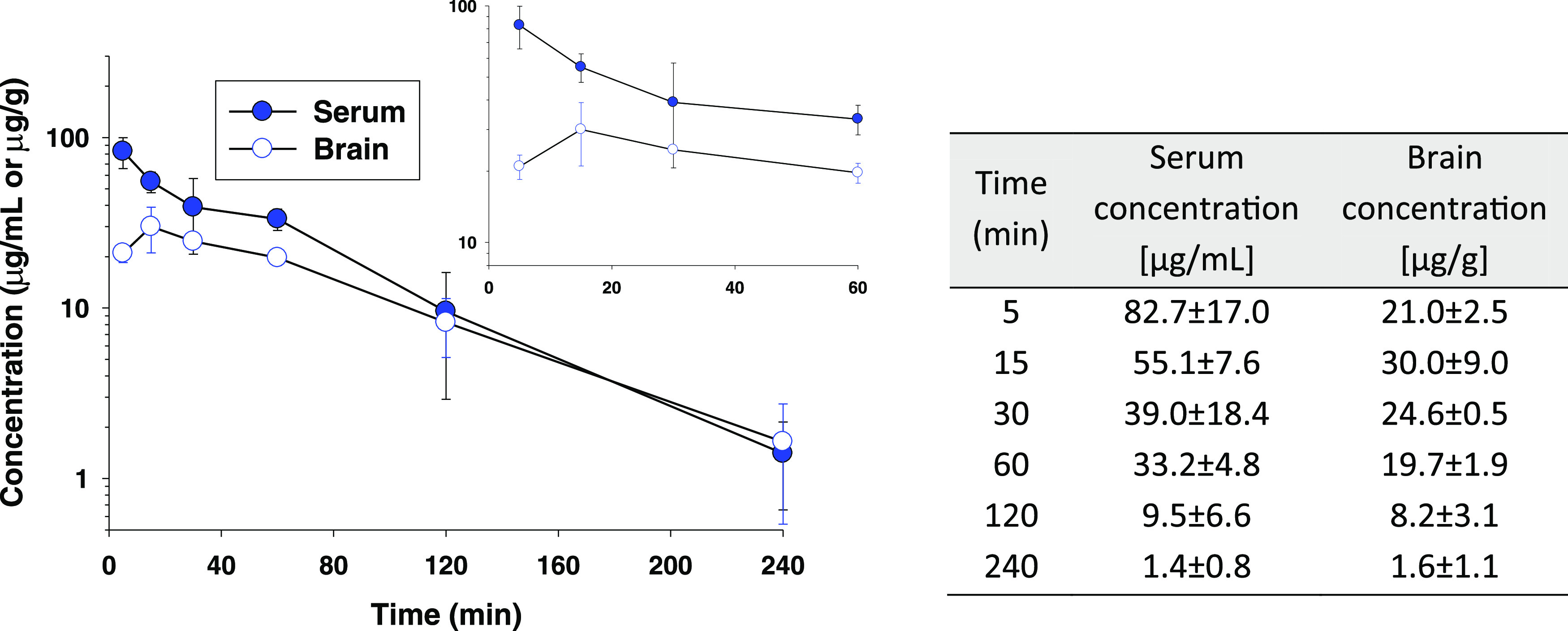
Serum and brain **(*****R*****)-7** [**(*****R*****)-AS-1**] concentrations (±SD) as a function of time following i.p. administration of a dose of 100 mg/kg to CD-1 mice (*n* = 3–4). The inset shows the concentration–time curves close to the peak (0–60 min).

The pharmacokinetic profile of **(*****R*****)-7** [**(*****R*****)-AS-1**] was assessed after its i.p. administration in mice at a screening dose of 100 mg/kg. The amount of the test compound in serum and brain was determined by a liquid chromatography–tandem mass spectrometry (LC-MS/MS) system (Figure 11).

Pharmacokinetic parameters estimated based on these data by the noncompartmental analysis are presented in Table 5.

**Table 5:** Pharmacokinetic Parameters of **(*****R*****)-7** [**(*****R*****)-AS-1**] in Serum and Brain Tissue Following i.p. Administration of This Compound at a Dose of 100 mg/kg to Mice

parameter^a^	serum	brain
*t*_max_ (min)	5	15
*C*_max_ (μg/mL(g))	82.7	30.0
λ*_z_* (min^–1^)	0.017	0.014
*t*_0.5λ*z*_ (min)	42.0	50.1
*V*_z_/*F* (L/kg)	1.3	
CL/*F* (L/min/kg)	0.02	
AUC_0–∞_ (μg·min/mL(g))	4625.8	2813.0
MRT (min)	59.1	77.0

a Pharmacokinetic parameters: *C*_max_—maximum serum/brain concentration; *t*_max_—time to reach *C*_max_; λ*_z_*—terminal slope; *t*_.5λ*z*_—terminal half-life; *V*_z_/*F*—volume of distribution; CL/*F*—clearance; AUC_0–∞_—area under the curve, MRT—mean residence time.

As presented in this table, the peak concentration of **(*****R*****)-7** [**(*****R*****)-AS-1**] in serum was attained at the first observation time (*i.e.*, at 5 min) indicating that the compound is very quickly absorbed from the peritoneal cavity. The maximum concentration in murine brain was observed slightly later than in serum, namely, 15 min after dosing. **(*****R*****)-7** [**(*****R*****)-AS-1**] was able to penetrate the blood–brain barrier as the brain-to-serum AUC ratio was 0.6. Its elimination from both serum and brain was rather slow as the terminal half-lives assessed in these matrices were longer than 40 and 50 min, respectively. The mean residence time (MRT) values were also somehow higher in brain than in serum (77 *vs* 59 min), which may limit off-target actions. As the percentage of water in fat-free wet weight of mice is about 80%,^79^ the volume of distribution (1.3 L/kg) estimated in this study only slightly exceeded mice total body water, confirming a moderate distribution of **(*****R*****)-7** [**(*****R*****)-AS-1**] to organs and tissues and a limited degree of tissues binding. The total clearance calculated using the noncompartmental approach (approx. 0.6 mL/min) was lower than the mouse liver blood flow (2.25 mL/min).^80^ This may suggest that the compound is not extensively metabolized in the liver. However, the values of both *V_z_* and CL calculated after i.p. dosing are dependent on the fraction of dose absorbed (*F*). If this fraction is lower than 1, the calculated values of these parameters following extravascular administration are higher than the true values, *i.e.*, estimated following intravenous administration, where *F* = 1. Thus, given these considerations, especially in relation to *V_z_*, the results obtained should be taken with caution.

## Conclusions

The present chemical and pharmacological studies confirmed favorable absolute configuration for the optimal antiseizure activity of racemates identified in a group of *N*-benzyl-2-(2,5-dioxopyrrolidin-1-yl)propanamides reported previously. Consequently, *R*-enantiomers were identified as the eutomers, and revealed wide-spectrum and potent protection in several of the most crucial acute seizure models in mice (i.p.). These models include MES, 6 Hz (32 mA), *sc*PTZ, and the 6 Hz (44 mA), which is recognized as a model of drug-resistant epilepsy. The compound with the most beneficial antiseizure properties and the best safety profile in the rotarod performance test was **(*****R*****)-7** [**(*****R*****)-AS-1**], which then was identified as the lead compound. Additionally, this molecule displayed potent protection in the chronic PTZ-kindling model in mice i.p., as well as in the PTZ-zebrafish seizures. **(*****R*****)-7** [**(*****R*****)-AS-1**] was effective when given orally to mice, showing satisfactory bioavailability from the gastrointestinal tract. Notably, **(*****R*****)-7** [**(*****R*****)-AS-1**] revealed a unique and novel mechanism of action, not yet observed in other ASDs, as it is a selective PAM of glutamate transport by EAAT2. This mechanism of action was tested in COS-7 and glia cells and supported by molecular docking studies. Thus, the lead compound **(*****R*****)-7** [**(*****R*****)-AS-1**] may be classified as the first-in-class small-molecule and amino-acid (d-alanine)-based ASD candidate, that acts as a selective EAAT2 PAM. This unique mechanism of action together with potent antiseizure activity, an excellent *in vivo*/*in vitro* safety profile, and satisfying pharmacokinetic properties make **(*****R*****)-7** [**(*****R*****)-AS-1**] a very promising candidate for more detailed preclinical and clinical development in epilepsy, as well as other neurological and psychiatric diseases related to increased glutamate excitotoxicity (*i.e.*, amyotrophic lateral sclerosis, Alzheimer’s disease, Parkinson’s disease, Huntington’s disease, ischemia, schizophrenia, neuropathic pain, anxiety, depression, and autism spectrum disorders). It should be noted that PAMs of EAAT2 might have an advantage over enhancers of EAAT2 expression,^81,82^ which are mostly limited to β-lactam antibiotics such as ceftriaxone. These β-lactam antibiotics have several pharmacokinetic and practical issues, including the necessity of parenteral administration, poor brain penetrability, limitations related to chronic dosing, and adverse effect profile.^83^ Therefore, **(*****R*****)-7** [**(*****R*****)-AS-1**] that is devoid of these drawbacks is a promising drug candidate with a novel treatment strategy worthy of more detailed evaluation.

In the next steps, we will determine the effect of **(*****R*****)-7** [**(*****R*****)-AS-1**] in more advanced seizure models (*i.e.*, the lamotrigine-resistant amygdala kindling model or the Dravet syndrome model, *etc.*), as well as other *in vivo* models of neuropathic pain, depression, or anxiety, which are all models that involve the dysregulation of the glutamatergic system.^84,85^ Finally, detailed safety and tolerability assays after acute and subchronic treatment in rodents will be carried out. In summary, we assume that the data described herein and the additional studies we plan to perform in the near future will confirm the potential of **(*****R*****)-7** [**(*****R*****)-AS-1**] as a promising, first-in-class ASD candidate.

## Experimental Section

### Chemistry

#### General Information

All chemicals and solvents were purchased from commercial suppliers and were used without further purification. Melting points (mp.) were determined in open capillaries on a Büchi 353 melting point apparatus (Büchi Labortechnik, Flawil, Switzerland) and are uncorrected. The purity and homogeneity of the compounds were assessed by thin-layer chromatography (TLC) and the gradient UPLC chromatography. Thin-layer TLC was performed in silica gel 60 F_254_ precoated aluminum sheets (Macherey-Nagel, Düren, Germany), using a developing system that consisted of the following: S_1_-DCM/MeOH (9:0.3; v/v), S_2_-DCM/MeOH (9:0.5; v/v). Spots were detected by their absorption under UV light (λ = 254 nm). The UPLC analyses and mass spectra (LC-MS) were obtained on the Waters ACQUITY TQD system (Waters, Milford, CT) with the MS-TQ detector and UV-Vis-DAD eλ detector. The ACQUITY UPLC BEH C18, 1.7 μm (2.1 × 100 mm^2^) column was used with the VanGuard Acquity UPLC BEH C18, 1.7 μm (2.1 × 5 mm^2^) (Waters, Milford, CT). Standard solutions (1 mg/mL) of each compound were prepared in analytical grade acetonitrile (MeCN)/water mixture (1:1; v/v). Conditions applied were as follows: eluent A (water/0.1% HCOOH), eluent B (MeCN/0.1% HCOOH), a flow rate of 0.3 mL/min, a gradient of 5–100% B over 10 min, and an injection volume of 10 μL. The UPLC retention times (*t*_R_) are given in minutes. The purity of target compounds determined by the use of the UPLC method was >99%. Preparative column chromatography was performed using silica gel 60 (particle size 0.063–0.200; 70–230 mesh ATM) purchased from Merck (Darmstadt, Germany). Elemental analyses (C, H, and N) for final compounds were carried out by a micro method using the elemental Vario EI III Elemental analyzer (Hanau, Germany). The results of elemental analyses were within ±0.4% of the theoretical values. ^1^H NMR and ^13^C NMR spectra were obtained in a JEOL-500 spectrometer (JEOL USA, Inc. MA), in CDCl_3_ operating at 500 MHz (^1^H NMR) 126 MHz (^13^C NMR). Chemical shifts are reported in δ values (ppm) relative to tetramethylsilane (TMS) δ = 0 (^1^H), as internal standard (IS). The *J* values are expressed in hertz (Hz). Signal multiplicities are represented by the following abbreviations: singlet (s), broad singlet (br s), doublet (d), double double doublet (ddd), triplet (t), triple doublet (td), quartet (q), multiplet (m). The optical activity and the specific optical rotation ([α]_D_^20^) of chiral imides were determined on Jasco polarimeter p-2000 (Jasco Inc. Easton, MD). For these purposes, 0.1% solutions of compounds in DCM were prepared. Chiral HPLC assays were conducted on an HPLC system (Shimadzu Prominence i LC-2030C SD Plus, Shimadzu Corporation, Kyoto, Japan) equipped with Chiralart Amylose-C column (250 × 4.6 mm^2^). The conditions applied were as follows: hexane/*i*-PrOH = 85/15 (v/v), flow rate: 0.7 mL/min, detection at λ = 209 nm. HRMS was performed commercially on Bruker Impact II spectrometer using electrospray ionization quadrupole time-of-flight tandem mass spectrometry (ESI-QTOF) by the Jagiellonian Centre of Innovation (Krakow, Poland).

### Method for the Preparation of **(*R*)-1**, **(*R*)-2** and **(*S*)-1**, **(*S*)-2**

To an anhydrous DCM (100 mL) solution of Boc-d-alanine or Boc-l-alanine (5.0 g, 27 mmol, 1 equiv) was successively added DCC (6.81 g, 1.2 equiv) dissolved in 10 mL of anhydrous DCM. After stirring (15 min), the benzylamine or 2-fluorobenzylamine (1 equiv) dissolved in 5 mL of anhydrous DCM was added dropwise and the reaction was stirred at room temperature for 1 h. The DCM was evaporated in vacuum, and the product was purified by column chromatography using a DCM/MeOH—9:0.3 (*v/v*) (S_1_) mixture as a solvent system.

#### (*R*)-*tert*-Butyl-(1-(benzylamino)-1-oxopropan-2-yl)carbamate **(*R*)-1**

Light oil. Yield: 91% (6.95 g); TLC: *R_f_***=** 0.43 (S_1_); UPLC (purity >99%): *t*_R_ = 5.45 min. C_15_H_22_N_2_O_3_ (278.35). LC-MS (ESI): *m*/*z* calcd for C_15_H_22_N_2_O_3_ (M + H)^+^ 279.16, found 279.18. ^1^H NMR (500 MHz, CDCl_3_) δ 1.26–1.48 (m, 12H), 4.19 (br s, 1H), 4.40 (br s, 2H), 5.13 (br s, 1H), 6.69–6.78 (m, 1H), 7.20–7.25 (m, 3H), 7.28 (d, *J* = 7.2 Hz, 2H). ^13^C NMR (126 MHz, CDCl_3_) δ 18.4, 28.4, 43.4, 50.2, 80.2, 127.6, 127.6, 128.7, 138.2, 155.7, 172.8.

#### (*R*)-*tert*-Butyl-(1-((2-fluorobenzyl)amino)-1-oxopropan-2-yl)carbamate **(*R*)-2**

Light oil. Yield: 92% (7.20 g); TLC: *R_f_***=** 0.43 (S_1_); UPLC (purity >99%): *t*_R_ = 5.59 min. C_15_H_21_FN_2_O_3_ (296.34). LC-MS (ESI): *m*/*z* calcd for C_15_H_21_FN_2_O_3_ (M + H)^+^ 297.15, found 279.26. ^1^H NMR (500 MHz, CDCl_3_) δ 1.26–1.41 (m, 12H), 4.18 (br s, 1H), 4.46 (d, *J* = 4.6 Hz, 2H), 5.06 (br s, 1H), 6.72 (br s, 1H), 7.00 (ddd, *J* = 9.9, 8.5, 1.0 Hz, 1H), 7.06 (td, *J* = 7.5, 1.2 Hz, 1H), 7.20–7.25 (m, 1H), 7.28 (t, *J* = 7.6 Hz, 1H). ^13^C NMR (126 MHz, CDCl_3_) δ 18.2, 28.3, 37.5 (d, *J* = 3.6 Hz), 50.1, 80.3, 115.3, 115.5, 124.3 (d, *J* = 3.0 Hz), 125.1, 129.3, 130.0, 160.0, 172.8.

#### (*S*)-*tert*-Butyl-(1-(benzylamino)-1-oxopropan-2-yl)carbamate **(*S*)-1**

Light oil. Yield: 89% (6.80 g); TLC: *R_f_***=** 0.43 (S_1_); UPLC (purity >99%): *t*_R_ = 5.41 min. C_15_H_22_N_2_O_3_ (278.35). LC-MS (ESI): *m*/*z* calcd for C_15_H_22_N_2_O_3_ (M + H)^+^ 279.16, found 279.28. ^1^H NMR (500 MHz CDCl_3_) δ 1.33–1.44 (m, 12H), 4.19 (br s, 1H), 4.40 (br s, 2H), 5.13 (br s, 1H), 6.74 (br s, 1H), 7.23 (t, *J =* 8.2 Hz, 3H), 7.28 (d, *J =* 7.2 Hz, 2H). ^13^C NMR (126 MHz, CDCl_3_) δ 18.4, 28.4, 43.4, 50.2, 80.2, 127.5, 127.6, 128.7, 138.2, 155.7, 172.8.

#### (*S*)-*tert*-Butyl-(1-((2-fluorobenzyl)amino)-1-oxopropan-2-yl)carbamate **(*S*)-2**

Light oil. Yield: 90% (7.04 g); TLC: *R_f_***=** 0.43 (S_1_); UPLC (purity >99%): *t*_R_ = 5.54 min. C_15_H_21_FN_2_O_3_ (296.34). LC-MS (ESI): *m*/*z* calcd for C_15_H_21_FN_2_O_3_ (M + H)^+^ 297.15, found 279.49. ^1^H NMR (500 MHz, CDCl_3_) δ 1.32–1.43 (m, 12H), 4.18 (br s, 1H), 4.46 (d, *J =* 4.6 Hz, 2H), 5.06 (br s, 1H), 6.72 (br s, 1H), 7.00 (ddd, *J =* 9.9, 8.5, 1.0 Hz, 1H), 7.06 (td, *J* = 7.5, 1.2 Hz, 1H), 7.19–7.24 (m, 1H), 7.28 (t, *J =* 7.6 Hz, 1H). ^13^C NMR (126 MHz, CDCl_3_) δ 18.2, 28.3, 37.5 (d, *J =* 3.6 Hz), 50.1, 80.3, 115.3, 115.5, 124.3(d, *J =* 3.0 Hz), 125.1 (d, *J =* 14.5 Hz), 129.3 (d, *J* = 7.8 Hz), 130.0, 160.0, 172.8.

### Procedure for the Preparation of **(*R*)-3**, **(*R*)-4** and **(*S*)-3**, **(*S*)-4**

The DCM (100 mL) solution of **(*R*)-1**, **(*R*)-2**, **(*S*)-1** or **(*S*)-2** (20 mmol, 1 equiv) was treated with TFA (6.84 g, 4.56 mL, 60 mmol, 3 equiv) and stirred at room temperature for 1 h. Afterward, the organic solvents were evaporated in vacuum. The resulting oil residue was dissolved in water (20 mL) and then 25% ammonium hydroxide was carefully added to pH = 8. The aqueous layer was extracted with DCM (3 × 50 mL), dried over Na_2_SO_4_, and concentrated in vacuum to give the **(*R*)-3**, **(*R*)-4** and **(*S*)-3**, **(*S*)-4** as yellow oils, and were used to further reaction without purification.

#### (*R*)-2-Amino-*N*-benzylpropanamide **(*R*)-3**

Yellow oil. Yield: 89% (3.17 g); TLC: *R_f_***=** 0.21 (S_2_) UPLC (purity 99%): *t*_R_ = 2.07 min. C_10_H_14_N_2_O (178.24). LC-MS (ESI): *m*/*z* calcd for C_10_H_14_N_2_O (M + H)^+^ 179.11, found 179.24. ^1^H NMR (500 MHz, CDCl_3_) δ 1.33 (d, *J* = 6.9 Hz, 3H), 1.69 (br s, 2H), 3.50 (q, *J* = 7.0 Hz, 1H), 4.40 (d, *J* = 6.0 Hz, 2H), 7.22–7.26 (m, 3H), 7.28–7.32 (m, 2H), 7.64 (br s, 1H). ^13^C NMR (126 MHz, CDCl_3_) δ 21.9, 43.1, 50.8, 127.5, 127.7, 128.7, 138.5, 175.8.

#### (*R*)-2-Amino-*N*-(2-fluorobenzyl)propanamide **(*R*)-4**

Yellow oil. Yield: 91% (3.57 g); TLC: *R_f_***=** 0.21 (S_2_) UPLC (purity >99%): *t*_R_ = 2.27 min. C_10_H_13_FN_2_O (196.23). LC-MS (ESI): *m*/*z* calcd for C_10_H_13_FN_2_O (M + H)^+^ 197.10, found 197.23. ^1^H NMR (500 MHz, CDCl_3_) δ 1.32 (d, *J =* 7.2 Hz, 3H), 1.65 (br s, 2H), 3.50 (q, *J =* 6.9 Hz, 1H), 4.46 (d, *J =* 6.0 Hz, 2H), 7.01 (ddd, *J =* 9.9, 8.5, 1.2 Hz, 1H), 7.07 (td, *J =* 7.5, 1.2 Hz, 1H), 7.20–7.25 (m, 1H), 7.29 (td, *J =* 7.6, 1.7 Hz, 1H), 7.67 (br s, 1H). ^13^C NMR (126 MHz, CDCl_3_) δ 21.8, 37.1 (d, *J =* 3.6 Hz), 50.8, 115.4 (d, *J =* 21.7 Hz), 124.3 (d, *J =* 3.6 Hz), 125.5 (d, *J =* 15.1 Hz), 129.3 (d, *J =* 8.4 Hz), 130.1 (d, *J =* 4.2 Hz), 161.1 (d, *J =* 246.3 Hz), 175.8.

#### (*S*)-2-Amino-*N*-benzylpropanamide **(*S*)-3**

Yellow oil. Yield: 87% (3.10 g); TLC: *R_f_***=** 0.21 (S_2_) UPLC (purity >99%): *t*_R_ = 2.14 min. C_10_H_14_N_2_O (178.24). LC-MS (ESI): *m*/*z* calcd for C_10_H_14_N_2_O (M + H)^+^ 179.11, found 179.17. ^1^H NMR (500 MHz, CDCl_3_) δ 1.33 (d, *J =* 6.9 Hz, 3H), 1.69 (br s, 2H), 3.50 (q, *J =* 7.0 Hz, 1H), 4.40 (d, *J =* 6.0 Hz, 2H), 7.21–7.26 (m, 3H), 7.28–7.32 (m, 2H), 7.64 (br s, 1H). ^13^C NMR (126 MHz, CDCl_3_) δ 21.9, 43.1, 50.8, 127.4, 127.7, 128.8, 138.6, 175.8.

#### (*S*)-2-Amino-*N*-(2-fluorobenzyl)propanamide **(*S*)-4**

Yellow oil. Yield: 88% (3.45 g); TLC: *R_f_***=** 0.21 (S_2_) UPLC (purity >99%): *t*_R_ = 2.23 min. C_10_H_13_FN_2_O (196.23). LC-MS (ESI): *m*/*z* calcd for C_10_H_13_FN_2_O (M + H)^+^ 197.10, found 197.18. ^1^H NMR (500 MHz, CDCl_3_) δ 1.32 (d, *J =* 7.2 Hz, 3H), 1.65 (br s, 2H), 3.50 (q, *J =* 6.9 Hz, 1H), 4.46 (d, *J =* 6.0 Hz, 2H), 7.01 (ddd, *J =* 9.9, 8.5, 1.2 Hz, 1H), 7.07 (td, *J =* 7.5, 1.2 Hz, 1H), 7.20–7.24 (m, 1H), 7.29 (td, *J =* 7.6, 1.7 Hz, 1H), 7.67 (br s, 1H). ^13^C NMR (126 MHz, CDCl_3_) δ 21.8, 37.1 (d, *J =* 3.6 Hz), 50.8, 115.4 (d, *J =* 21.7 Hz), 124.3 (d, *J =* 3.6 Hz), 125.5 (d, *J =* 15.1 Hz), 129.3 (d, *J =* 8.4 Hz), 130.1 (d, *J =* 4.2 Hz), 161.1 (d, *J =* 246.3 Hz), 175.8.

### Procedure for the Preparation of **(*R*)-5**, **(*R*)-6** and **(*S*)-5**, **(*S*)-6**

To a solution of succinic anhydride (1.56 g, 15 mmol, 1 equiv) in ethyl acetate (10 mL) was added a solution of **(*R*)-3**, **(*R*)-4**, **(*S*)-3**, or **(*S*)-4** (15 mmol, 1 equiv) in ethyl acetate (40 mL). The reaction mixture was stirred for 0.5 h, after this time, ethyl acetate was evaporated to dryness. The succinamic acid (***R***)-**5**, (***R***)-**6** and (***S***)-**5**, (***S***)-**6** were obtained as solid substances after washing with diethyl ether.

#### (*R*)-4-((1-(Benzylamino)-1-oxopropan-2-yl)amino)-4-oxobutanoic Acid **(*R*)-5**

White solid. Yield: 95% (3.96 g); TLC: *R_f_***=** 0.34 (S_2_) UPLC (purity 99%): *t*_R_ = 3.23 min. C_14_H_18_N_2_O_4_ (278.31). LC-MS (ESI): *m*/*z* calcd for C_14_H_18_N_2_O_4_ (M + H)^+^ 279.13, found 279.18. ^1^H NMR (500 MHz, CDCl_3_) δ 1.24–1.29 (m, 3H), 2.36–2.41 (m, 2H), 2.49–2.56 (m, 2H), 3.72–3.86 (m, 2H), 4.29 (d, *J =* 7.5 Hz, 2H), 7.12–7.28 (m, 5H), 7.36 (br s, 1H), 7.64–7.73 (m, 1H). ^13^C NMR (126 MHz, CDCl_3_) δ 17.8, 28.8, 28.9, 29.3, 30.6, 43.2, 49.2, 127.3, 127.4, 128.6, 138.0, 172.5, 172.9, 175.2.

#### (*R*)-4-((1-((2-Fluorobenzyl)amino)-1-oxopropan-2-yl)amino)-4-oxobutanoic Acid **(*R*)-6**

White solid. Yield: 93% (4.13 g); TLC: *R_f_***=** 0.34 (S_2_) UPLC (purity >99%): *t*_R_ = 3.23 min. C_14_H_17_FN_2_O_4_ (296.30). LC-MS (ESI): *m*/*z* calcd for C_14_H_17_FN_2_O_4_ (M + H)^+^ 297.12, found 279.13. ^1^H NMR (500 MHz, CDCl_3_) δ 1.29 (d, *J =* 7.2 Hz, 3H), 2.40–2.44 (m, 2H), 2.55–2.58 (m, 2H), 3.64 (s, 1H), 4.33–4.35 (m, 1H), 4.36–4.41 (m, 2H), 6.94–6.99 (m, 1H), 7.02–7.06 (m, 1H), 7.14–7.26 (m, 2H), 7.57 (br s, 1H). ^13^C NMR (126 MHz, CDCl_3_) δ 17.9, 24.9, 25.6, 29.4, 48.9, 115.2, 115.4, 124.3 (d, *J =* 3.6 Hz), 124.9 (d, *J =* 15.1 Hz), 129.2 (d, *J =* 7.8 Hz), 129.8, 160.8 (d, *J =* 246.3 Hz), 172.4, 173.0, 175.4.

#### (*S*)-4-((1-(Benzylamino)-1-oxopropan-2-yl)amino)-4-oxobutanoic Acid **(*S*)-5**

White solid. Yield: 96% (4.00 g); TLC: *R_f_***=** 0.34 (S_2_) UPLC (purity >99%): *t*_R_ = 3.23 min. C_14_H_18_N_2_O_4_ (278.31). LC-MS (ESI): *m*/*z* calcd for C_14_H_18_N_2_O_4_ (M + H)^+^ 279.13, found 279.26. ^1^H NMR (500 MHz, CDCl_3_) δ 1.22–1.30 (m, 3H), 2.35–2.42 (m, 2H), 2.53 (dt, *J =* 4.8, 2.3 Hz, 2H), 3.74–3.85 (m, 2H), 4.27–4.36 (m, 2H), 7.12–7.27 (m, 5H), 7.36 (br s, 1H), 7.66–7.70 (m, 1H). ^13^C NMR (126 MHz, CDCl_3_) δ 17.8, 28.8, 28.9, 29.3, 30.6, 43.2, 49.2, 127.3, 127.4, 128.6, 138.0, 172.5, 172.9, 175.2.

#### (*S*)-4-((1-((2-Fluorobenzyl)amino)-1-oxopropan-2-yl)amino)-4-oxobutanoic Acid **(*S*)-6**

White solid. Yield: 96% (4.26 g); TLC: *R_f_***=** 0.34 (S_2_) UPLC (purity >99%): *t*_R_ = 3.32 min. C_14_H_17_FN_2_O_4_ (296.30). LC-MS (ESI): *m*/*z* calcd for C_14_H_17_FN_2_O_4_ (M + H)^+^ 297.12, found 297.27. ^1^H NMR (500 MHz, CDCl_3_) δ 1.29 (d, *J =* 7.2 Hz, 3H), 2.40–2.44 (m, 2H), 2.54–2.58 (m, 2H), 3.64 (s, 1H), 4.33–4.35 (m, 1H), 4.36–4.43 (m, 2H), 6.93–6.99 (m, 1H), 7.04 (td, *J =* 7.5, 1.2 Hz, 1H), 7.15–7.27 (m, 2H), 7.57 (br s, 1H). ^13^C NMR (126 MHz, CDCl_3_) δ 17.9, 24.9, 25.6, 29.4, 48.9, 115.2, 115.4, 124.3 (d, *J =* 3.6 Hz), 124.9 (d, *J =* 15.1 Hz), 129.2 (d, *J =* 7.8 Hz), 129.8, 160.8 (d, *J =* 246.3 Hz), 172.4, 173.0, 175.4.

### Procedure for the Preparation of **(*R*)-7** [**(*R*)-AS-1**], **(*R*)-8** and **(*S*)-7**, **(*S*)-8**

To a suspension of the succinamic acids **(*R*)-5**, **(*R*)-6**, **(*S*)-5**, or **(*S*)-6** (10 mmol, 1 equiv) in dry 1,4-dioxane (50 mL) was added ZnCl_2_ (1.35 g, 10 mmol, 1 equiv), and the mixture was heated to 100 °C. Afterward, a solution of HMDS (1.62 g, 2.1 mL, 10 mmol, 1.5 equiv) in dry 1,4-dioxane (5 mL) was added dropwise over 30 min. The reaction mixture was refluxed for an additional 2 h and concentrated under reduced pressure. The resulting oil residue was dissolved in DCM (50 mL) and then 10% hydrochloric acid was added. The aqueous layer was extracted with DCM (3 × 50 mL), dried over Na_2_SO_4_, and concentrated in vacuum. The final compound was obtained as solid substances followed by the concentration of organic solvents under reduced pressure and crystallized in 2-propanol.

#### (*R*)-*N*-Benzyl-2-(2,5-dioxopyrrolidin-1-yl)propanamide **(*R*)-7** [**(*R*)-AS-1**]

White solid. Yield: 90% (2.34); melting point: 138.2–138.9 °C; TLC: *R_f_* = 0.39 (S_1_); UPLC (purity >99%): *t*_R_ = 3.81 min. C_14_H_16_N_2_O_3_ (260.29); LC-MS (ESI): *m*/*z* calcd for C_14_H_16_N_2_O_3_ (M + H)^+^ 261.12, found 261.25; HRMS (ESI/Q-TOF): *m*/*z* calcd for C_14_H_16_N_2_O_3_Na [M + Na]^+^ 283.1059, found 283.1046. ^1^H NMR (500 MHz, CDCl_3_) δ 1.56 (d, *J =* 7.5 Hz, 3H), 2.66 (s, 4H), 4.38 (d, *J =* 5.7 Hz, 2H), 4.76 (q, *J =* 7.3 Hz, 1H), 6.43 (br s, 1H), 7.21–7.26 (m, 3H), 7.29–7.32 (m, 2H). ^13^C NMR (126 MHz, CDCI_3_) δ 14.5, 28.2, 43.8, 49.8, 127.6, 127.7, 128.8, 137.9, 168.6, 177.0. Chiral HPLC >99% ee (*t*_R_ = 22.649 min). [α]_D_^20^ +51.49° (*c* 0.1, DCM). Anal. calcd for C_14_H_16_N_2_O_3_: C, 64.58; H, 6.22; N, 10.79; found: C, 64.53; H, 6.39; N, 10.76.

#### (*R*)-2-(2,5-Dioxopyrrolidin-1-yl)-*N*-(2-fluorobenzyl)propanamide **(*R*)-8**

White solid. Yield: 89% (4.47); melting point: 115.1–118.8 °C; TLC: *R_f_* = 0.43 (S_1_); UPLC (purity >99%): *t*_R_ = 4.03 min. C_14_H_15_FN_2_O_3_ (278.28), LC-MS (ESI): *m*/*z* calcd for C_14_H_15_FN_2_O_3_ (M + H)^+^ 279.11, found 279.06; HRMS (ESI/Q-TOF): *m*/*z* calcd for C_14_H_15_FN_2_O_3_Na [M + Na]^+^ 301.0964, found 301.0948. ^1^H NMR (500 MHz, CDCl_3_) δ 1.57 (d, *J =* 7.2 Hz, 3H), 2.69 (s, 4H), 4.39–4.51 (m, 2H), 4.76 (q, *J =* 7.2 Hz, 1H), 6.50 (br s, 1H), 7.00 (t, *J =* 9.0 Hz, 1H), 7.08 (td, *J =* 7.5, 1.2 Hz, 1H), 7.21–7.24 (m, 1H), 7.30 (td, *J =* 7.6, 1.7 Hz, 1H). ^13^C NMR (126 MHz, CDCl_3_) δ 14.5, 28.2, 37.9 (d, *J =* 4.2 Hz), 49.8, 115.4 (d, *J =* 21.7 Hz), 124.5 (d, *J =* 3.6 Hz), 124.8 (d, *J =* 14.5 Hz), 129.4 (d, *J =* 8.5 Hz), 130.2 (d, *J =* 4.2 Hz), 161.0 (d, *J =* 246.3 Hz), 168.8, 176.9. Chiral HPLC >99% ee (*t*_R_ = 24.859 min). [α]_D_^20^ +27.90° (*c* 0.1, DCM). Anal. calcd for C_14_H_15_FN_2_O_3_: C, 60.43; H, 5.43; N, 10.07; found: C, 60.55; H, 5.69; N, 10.42.

#### (*S*)-*N*-Benzyl-2-(2,5-dioxopyrrolidin-1-yl)propanamide **(*S*)-7**

White solid. Yield: 87% (2.26); melting point: 138.4–139.2 °C; TLC: *R_f_* = 0.39 (S_1_); UPLC (purity >99%): *t*_R_ = 3.85 min. C_14_H_16_N_2_O_3_ (260.29), LC-MS (ESI): *m*/*z* calcd for C_14_H_16_N_2_O_3_ (M + H)^+^ 261.12, found 261.22; HRMS (ESI/Q-TOF): *m*/*z* calcd for C_14_H_16_N_2_O_3_Na [M + Na]^+^ 283.1059, found 283.1042. ^1^H NMR (500 MHz, CDCl_3_) δ 1.56 (d, *J =* 7.5 Hz, 3H), 2.66 (s, 4H), 4.38 (d, *J =* 5.7 Hz, 2H), 4.76 (q, *J =* 7.3 Hz, 1H), 6.43 (br s, 1H), 7.21–7.26 (m, 3H), 7.29–7.32 (m, 2H). ^13^C NMR (126 MHz, CDCI_3_) δ 14.5, 28.2, 43.8, 49.8, 127.6, 127.7, 128.8, 137.9, 168.6, 177.0. Chiral HPLC >99% ee (*t*_R_ = 24.008 min). [α]_D_^20^ −49.52° (*c* 0.1, DCM). Anal. calcd for C_14_H_16_N_2_O_3_: C, 64.58; H, 6.22; N, 10.79; found: C, 64.64; H, 6.15; N, 10.87.

#### (*S*)-2-(2,5-Dioxopyrrolidin-1-yl)-*N*-(2-fluorobenzyl)propanamide **(*S*)-8**

White solid. Yield: 91% (2.53); melting point: 115.2–118.7 °C; TLC: *R_f_* = 0.43 (S_1_); UPLC (purity >99%): *t*_R_ = 4.03 min. C_14_H_15_FN_2_O_3_ (278.28), LC-MS (ESI): *m*/*z* calcd for C_14_H_15_FN_2_O_3_ (M + H)^+^ 279.11, found 279.19; HRMS (ESI/Q-TOF): *m*/*z* calcd for C_14_H_15_FN_2_O_3_Na [M + Na]^+^ 301.0964, found 301.0945. ^1^H NMR (500 MHz, CDCl_3_) δ 1.57 (d, *J =* 7.2 Hz, 3H), 2.69 (s, 4H), 4.39–4.51 (m, 2H), 4.74–4.77 (m, 1H), 6.50 (br s, 1H), 7.00 (t, *J =* 9.0 Hz, 1H), 7.08 (td, *J =* 7.5, 1.2 Hz, 1H), 7.21–7.24 (m, 1H), 7.30 (td, *J =* 7.6, 1.7 Hz, 1H). ^13^C NMR (126 MHz, CDCl_3_) δ 14.5, 28.2, 37.9 (d, *J =* 4.2 Hz), 49.8, 115.4 (d, *J =* 21.7 Hz), 124.5 (d, *J =* 3.6 Hz), 124.8 (d, *J =* 14.5 Hz), 129.4 (d, *J =* 8.5 Hz), 130.2 (d, *J =* 4.2 Hz), 161.0 (d, *J =* 246.3 Hz), 168.8, 176.9. Chiral HPLC >99% ee (*t*_R_ = 19.291 min). [α]_D_^20^ −27.60° (*c* 0.1, DCM). Anal. calcd for C_14_H_15_FN_2_O_3_: C, 60.43; H, 5.43; N, 10.07; found: C, 64.58; H, 5.21; N, 10.32.

### Acute Antiseizure Models, Rotarod Test, and Locomotor Activity Test

#### General Information

Male adult CD-1 mice from the accredited animal facility Jagiellonian University Medical College (Krakow, Poland) that weighed between 22 and 26 g were used. The animals were kept in environmentally controlled rooms at a constant ambient temperature of 22 ± 2 °C, 12/12 light/dark cycle, with food and water available *ad libitum*. Experiments were carried out under EU Directive 2010/63/EU and approved by the Local Ethics Committee for Experiments on Animals of the Jagiellonian University in Krakow, Poland (50/2015, 111/2016, 149/2018, 289A/2019). Compounds were dissolved in 1% Tween 80 and administered i.p. 30 min or orally 60 min before the given test at a constant volume of 10 mL/kg. On each day of experimentation, fresh solutions were prepared. The ED_50_ or TD_50_ parameters were estimated based on the results obtained in three to five groups of animals consisting of six mice.

The maximal electroshock seizure (MES) test was performed according to the procedure by Löscher et al.^86^ Briefly, the mice received an electrical stimulus of sufficient intensity (25 mA, maximum output voltage 500 V, 50 Hz, 0.2 s) delivered *via* auricular electrodes by the electroshock generator (Rodent Shocker, type 221, Hugo Sachs, Germany) to induce maximal seizures. The endpoint was the tonic extension of the hind limbs. In vehicle-treated mice, the procedure caused immediate hindlimb tonic extension. Mice not displaying hindlimb tonic extension were considered to be protected from seizures.

The psychomotor seizure (6 Hz) test was performed according to the procedure by Barton et al.^47^ and Kaminski et al.^87^ In this test, seizures were induced by an electric stimulus of 32 mA and/or 44 mA (which represent 1.5× and 2× the convulsive current for inducing seizures in 97% of mice (CC_97_)) and a frequency of 6 pulses/s using an electric shock generator (ECT Unit 57800; Ugo Basile, Gemonio, Italy) and the corneal electrodes. Before the test, the eye surface was gently moistened with a solution of local anesthetic (1% lidocaine solution). An electrical pulse was delivered continuously for 3 s, followed by observation of the animal for 10 s. During this time, immobility or stun associated with rearing, forelimb clonus, twitching of the vibrissae, and Straub’s tail were observed. These symptoms persist throughout the observation period, indicating the occurrence of psychomotor seizures in mice. Mice resuming normal behavior within 10 s after stimulation were considered as protected.

The PTZ test was performed according to Łuszczki et al.^88^ with some minor modification to the procedure by Łączkowski et al.^89^ In this test, seizures were induced by subcutaneous administration of pentylenetetrazole (PTZ) at a dose of 100 mg/kg. After PTZ administration, the animals were placed individually and observed in the next 30 min for the occurrence of clonic seizures (clonus of the whole body lasting at least 3 s with loss of the righting reflex). The absence of clonic convulsions within the observed time period was interpreted as the compound’s ability to protect against PTZ-induced seizures. Moreover, the latency time to first seizure episode was noted and compared with the control group using one-way analysis of variance (ANOVA) and Dunnett’s *post hoc* test (multiple comparison test). The value at the significance level *p* < 0.05 was considered statistically significant.

The influence of tested compounds on motor coordination was assessed in the rotarod test (May Commat, RR 0711 Rota Rod, Turkey). Mice were trained the day before the actual experiment. They were placed individually on a 2 cm diameter rod rotating at 10 revolutions per minute (rpm). During each training session, the animals remained on the rod for 3 min. The experiment was carried out 30 min after administration of the compounds. Motor coordination was tested at the speed of the rotating bar: 10 rpm for 60 seconds. Motor impairments were defined as the inability to remain on the rotating rod for 1 min.

#### Data Analysis

ED_50_ and TD_50_ values with 95% confidence limits were calculated by probit analysis.^90^ The protective indexes for the compounds investigated and reference ASDs were calculated by dividing the TD_50_ value, as determined in the rotarod test, by the respective ED_50_ value, as determined in the MES, *sc*PTZ, or 6 Hz (32 or 44 mA) tests. The protective index is considered as an index of the margin of safety and tolerability between antiseizure doses and doses of the compounds exerting acute adverse effects such as motor coordination impairment, ataxia, or other neurotoxic manifestations.

#### Influence on Spontaneous Locomotor Activity

This test was carried out according to the procedure described elsewhere.^91^ The animals were injected i.p. with **(*****R*****)-7** [**(*****R*****)-AS-1**] or (***R***)**-8** at doses of 15 (only **(*****R*****)-7** [**(*****R*****)-AS-1**]), 30, 60, and 90 mg/kg and placed in the activity cages (Multiple Activity Cage; Ugo Basile, Gemonio VA, Italy) individually (30 min before experiment). The number of light-beam crossings was counted in each group during the next 30 min in 10 min intervals.

### PTZ-Induced Kindling Model in Mice

#### General Information

Naïve male albino Swiss mice obtained from the Experimental Medicine Centre (Lublin, Poland) were used for all experiments. The experimental procedures and protocols were approved by the Local Ethics Committee in Lublin (license no. 149/2018).

#### PTZ Kindling

To induce PTZ kindling, mice were injected i.p. with a subconvulsive dose of PTZ (40 mg/kg), three times a week for a total of 21 injections. **(*****R*****)-7** [**(*****R*****)-AS-1**] and VPA (positive control) were administered i.p. 30 min before each PTZ injection. **(*****R*****)-7** [**(*****R*****)-AS-1**] was suspended in 1% Tween 80, while VPA and PTZ were dissolved in normal saline. Immediately following injection, mice were placed individually into a transparent box for 30 min for behavioral observation. The seizure severity of each subject was scored using the modified Racine’s scale as follows: stage 0, no response; stage 1, immobility, ear and facial twitching; stage 2, myoclonic jerks; stage 3, forelimb clonus, stage 4, clonic seizure with rearing and falling; stage 5, generalized clonic seizure with loss of righting reflex; stage 6, tonic fore- and hindlimb extension. Experimental grouping was as follows: (a) 1% Tween + saline (nonkindled control), (b) 1% Tween + PTZ (PTZ control); (c) VPA at 150 mg/kg + PTZ (positive control); (d)–(f) PTZ + **(*****R*****)-7** [**(*****R*****)-AS-1**] at 10, 20 and 40 mg/kg. After kindling completion (24 h after the last PTZ injection), animals were subjected to the behavioral tests (the locomotor activity test, the elevated plus maze test, and the forced swim test) according to the methods described in detail elsewhere.^92^

#### Statistics

The mean seizure severity scores were calculated for all experimental groups after each PTZ injection. Repeated measures two-way ANOVA followed by the Bonferonni *post hoc* test was used to study the effect of treatment and time factors on seizure severity. Data from the behavioral tests were analyzed using one-way ANOVA.

### Antiseizure Activity in Zebrafish

Wild type (WT) adult zebrafish (AB strain) were obtained from Zebrafish International Resource Center (Eugene, Oregon). They were raised and kept under standard conditions (at 28.5 °C on a 14-h light/10-h dark cycle). Embryos resulting from natural spawning were maintained in E3 embryo water (1.5 mM *N*-(2-hydroxyethyl)piperazine-*N*′-ethanesulfonic acid (HEPES), pH 7.6, 17.4 mM NaCl, 0.21 mM KCl, 0.12 mM MgSO_4_ and 0.18 mM Ca(NO_3_)_2_), at 28.5 °C and on a 14-h/10-h light/dark cycle. All animal experiments got approval through the Norwegian Food Safety Authority experimental animal administration’s supervisory and application system (“Forsøksdyrforvatningentilsyns- og søknadssystem”, FOTS-ID 15469 and 23935). Compliance with the ARRIVE and the National Institute of Health Guidelines for the Care and Use of Laboratory Animals, the European Community Council Directive of November 2010 for Care and Use of Laboratory Animals (Directive 2010/63/EU) guidelines were applied to all experiments. The maximal tolerated dose of **(*****R*****)-7** [**(*****R*****)-AS-1**] was chosen as described previously.^49^ For the locomotor activity assessment, 6 dpf larvae were incubated for 24 h at 28.5 °C. Next, vehicle or PTZ (final concentration 20 mM) was added to each well. After 5 min, larval activity was tracked (ZebraBox, Viewpoint, Lyon, France) for 30 min with 2 min time bins. The distance covered by each larva in millimeters (mm) was assessed.^49^ The measurements were two or three times replicated and the data were pooled together. For the EEG measurements, each larva was mounted on a glass slide in a thin layer of 2% low-melting-point agarose. Then, the glass electrode (resistance 1–5 MΩ) filled with artificial cerebrospinal fluid (124 mM NaCl, 2 mM KCl, 2 mM MgSO_4_, 2 mM CaCl_2_, 1.25 mM KH_2_PO_4_, 26 mM NaHCO_3_, 10 mM glucose) was inserted into the optic tectum (MultiClamp 700B amplifier, Digidata 1550 digitizer, Axon instruments). The recordings for each larva were performed within 20 min. Clampfit 10.2 software (Molecular Devices Corporation) and custom-written R script for Windows were used for analysis purposes.^49^

### Computational Modeling and Simulations of Binding to Human EAAT2

The ligand-binding sites and binding poses of **(*****R*****)-7** [**(*****R*****)-AS-1**] and **(*****R*****)-8** on EAAT2 were determined using AutoDock Vina,^93^ based on the modeled OF, IF, and asymmetric EAAT2 conformers. Ligand docking simulations were first performed on EAAT2 trimers and then refined on EAAT2 protomers. Simulations were carried out using grids with dimensions set to encapsulate the entire structure of the protein. The total number of runs (exhaustiveness parameter in Autodock Vina) was set to 50 and the algorithm returned 20 binding modes of interest for each conformer based on these runs. Binding sites were rank-ordered based on binding affinities calculated using Vina. To compare with the binding sites of a known EAAT2 PAM, (*R*)-GT949^60^ (ZINC ID: 1382165), we docked (*R*)-GT949 onto EAAT2 conformers following the same protocol.

### Neurotransmitter Transport Studies in COS-7 Cell Lines

Cell Culture and DNA Transfection. COS-7 cells (ATCC, Manassas, AV) were maintained in Dulbecco’s modified Eagle’s medium (DMEM) containing 10% fetal bovine serum (FBS), 100 units/mL of penicillin, and 100 μg/mL streptomycin in a humidified incubator with 5% CO_2_ at 37 °C. Subconfluent COS-7 cells were transiently transfected with 0.5 μg of plasmid DNA (EAAT1, EAAT2, EAAT3, GAT-1, GAT-3) per well using TransIT-LT1 transfection reagent (Mirus Bio LLC, Madison, WI), and plated at a density of 50,000 cells/well and uptake experiments were performed 2 days after plating. Transfection with empty vector pCMV-5 was used to control for the level of endogenous uptake of radiolabeled substrate in each experimental condition.

#### Dose–Response Assays

Neurotransmitter uptake assays were performed as previously reported.^59,94^ Briefly, cells were washed with room temperature phosphate buffer PBS-CM (2.7 mM KCl; 1.2 mM KH_2_PO_4_, 138 mM NaCl; 8.1 mM Na_2_HPO_4_, added 0.1 mM CaCl_2_ and 1 mM MgCl_2_, pH 7.4) and incubated for 10 min at 37 °C with compounds **(*****R*****)-7** [**(*****R*****)-AS-1**] and (***R*****)-8** [**(*****R*****)-AS-7**] at 0.01–100 μM final concentration. Uptake reactions were initiated by the addition of an appropriate radiolabeled substrate ([^3^H]-l-glutamate or [^3^H]-GABA) at a final concentration of 50 nM. After 10 min, reactions were terminated in two washes with buffer and lysis with 1% sodium dodecyl sulfate (SDS)/0.1 M NaOH. The lysate was transferred to scintillation vials containing 3 mL of scintillation fluid (ScintiVerse, Fisher Scientific, Pittsburgh, PA) and radioactivity was quantified in a scintillation counter LS 6500 (Beckman Coulter, Brea, CA).

#### Kinetic Assays

For kinetic assays, COS-7 cells were transfected with wild-type (WT) EAAT2 or empty vector, as described above. After 2 days, the plates were preincubated with compound **(*****R*****)-7** [**(*****R*****)-AS-1**] for 10 min at the indicated concentrations. Uptake reactions were initiated by the addition of unlabeled l-glutamate and [^3^H]-l-glutamate (1–1000 μM, final concentration, 99% unlabeled and 1% labeled). After 10 min, uptake was terminated and radioactivity counted as above.

### Glutamate Transporter Studies in Astrocytes

#### Astrocyte Preparation

Glia was prepared and cultured according to a previous study,^95^ with modifications. Briefly, cerebral cortices from 2- to 4-day-old Sprague-Dawley rat pups were dissected under sterile conditions and placed in 60 mm dishes containing dissection medium (in mM: glucose 16, sucrose 22, NaCl 135, KCl 5, Na_2_HPO_4_ 1, KH_2_PO_4_ 0.22, HEPES 10, pH 7.4, osmolarity 310+10 mOsm). Tissue was minced with curved scissors, digested for 15 min in 0.25% trypsin, and dissociated by passing through a serological plastic pipette several times in the presence of 60 μg/mL DNAse. The cells were pelleted by centrifugation for 15 min at 280*g*, resuspended in glia plating medium (90% DMEM, 10% FBS, and 50 μg/mL gentamicin), and incubated in culture flasks at 37 °C (5–10% CO_2_). After growth for 10 days *in vitro* (DIV), the cells were detached with 0.05% trypsin, centrifuged, and plated at the density of 10,000 cells/well in polylysine-coated 96-well plates. Plates are grown for 14 DIV before uptake assays.

#### Uptake Assays

Assays were performed as described.^95^ Briefly, the cells were washed in PBS-CM buffer using an Elx50 Biotek plate washer (Winooski, VT). For dose–response assays, vehicle and several concentrations of compounds **(*****R*****)-7** [**(*****R*****)-AS-1**] and **(*****R*****)-8** [**(*****R*****)-AS-7**] were added and incubated for 10 min at 37 °C. Uptake assays were initiated by the addition of 50 nM [^3^H]-l-glutamate, and incubation was carried on for 10 min at room temperature. Nonspecific uptake was obtained in the presence of 10 μM DL-TBOA.

#### Kinetic Assays

For kinetic assays, the cells are washed in PBS-CM buffer and preincubated in the presence of either vehicle or several concentrations of **(*****R*****)-7** [**(*****R*****)-AS-1**], uptake reactions are initiated by the addition of unlabeled l-glutamate and [^3^H]-l-glutamate (1–1000 μM, final concentration, 99% unlabeled and 1% labeled). Incubation was carried on for 10 min at room temperature. Nonspecific uptake was also obtained in the presence of DL-TBOA.

Reactions were finished by washing the plates twice with PBS-CM and the addition of 100 μL of scintillation fluid to each well. Radioactivity was counted in a Microplate Scintillation and Luminescence Counter (Wallac, Shelton, CT).

### Pharmacokinetic Studies

#### General Information

Male CD-1 mice weighing 28–32 g were used in this study. The studies were approved by the Local Ethical Committee for Experiments on Animals of the Jagiellonian University in Krakow (Poland), No 270/2019. The animals were fasted overnight prior to drug administration but had free access to water. **(*****R*****)-7** [**(*****R*****)-AS-1**] was suspended in a 1% Tween 80 solution in sterile water for injection and given i.p. at a dose of 100 mg/kg (*n* = 3-4 per time point). Blood samples were collected at different time points following compound administration and allowed to clot at room temperature for 20 min. Moreover, brains were removed from skulls and washed with 0.9% NaCl. The blood samples were centrifuged for 10 min at the speed of 8000 rpm (Eppendorf miniSpin centrifuge). The obtained serum and brains were stored at −80 °C until analysis.

#### Chemicals and Reagents

Oxcarbazepine (an internal standard, IS) was purchased from Tocris Bioscience (Bristol, U.K.). MeCN, MeOH, and DCM of HPLC grade were from Sigma-Aldrich (Steinheim, Germany). Ultrapure deionized water (0.1 μS/cm) was prepared in-house using a Hydrolab water purification system (Poland) with the 0.2 μm microfiltration capsule.

#### Instrumentation and Chromatographic Condition

The analysis of **(*****R*****)-7** [**(*****R*****)-AS-1**] was performed using a Merck-Hitachi HPLC system (Japan) consisting of an autosampler (model L-2200), an UV–vis detector operating at 205 nm (model L-2420), and a computer (HP 6200 PRO) with EZChrom Elite Client/Server v. 3.2 software for data collection and analysis. The pump (Iso Chrom, SpectraPhysics) was used under isocratic conditions on a manual mode. The chromatographic separation of **(*****R*****)-7** [**(*****R*****)-AS-1**] and IS was achieved at ambient temperature (22 ± 1 °C) on the Supelcosil PCN cyanopropyl bonded-phase column 250 × 4.6 mm^2^ with 5 μm particles protected with the SUPELCOSIL LC-PCN guard column packed with the same packing material as the analytical column (both from Sigma-Aldrich, Steinheim, Germany). The mobile phase consisted of acetonitrile and water mixed in a 30:70 (v/v) ratio. The mobile phase was degassed in the ultrasonic bath (Polsonic, Poland) before use. The flow rate of 0.7 mL/min was used throughout the analytical run.

### Preparation of Standard Solutions

Stock solutions of **(*****R*****)-7** [**(*****R*****)-AS-1**] (5 mg/mL) and IS (1 mg/mL) were prepared in MeOH and kept at 4 °C. The stock solution of **(*****R*****)-7** [**(*****R*****)-AS-1**] was subsequently diluted in MeCN to prepare working standard solutions in the concentration ranges of 2–200 μg/mL for serum and 0.25–25 μg/g for brain tissue. IS solutions (IS_1_ = 500 μg/mL and IS_2_ = 250 μg/mL) were prepared by diluting the stock solution with MeCN.

### Sample Preparation

Frozen serum samples were thawed at room temperature and vortex-mixed briefly (Reax top, Heidolph, Germany). Then, 50 μL of the serum samples was transferred to Eppendorf tubes and an IS_1_ working solution (5 μL) was added. The samples were vortexed again to ensure homogeneous distribution of the IS. Then, 150 μL of MeCN with 0.1% formic acid was added for protein precipitation to each tube. The mixtures were vortexed and then centrifuged at 10,000 rpm for 10 min (Minispin Centrifuge, Eppendorf, Germany). Finally, 40 μL aliquots were injected into the HPLC system. Frozen mouse brains were thawed at room temperature, weighed, and then homogenized (4 mL/g) in distilled water with a tissue homogenizer TH220 (Omni International, Inc.). The brain homogenates (1 mL) were transferred to the glass tubes. Then, 5 μL of IS_2_ working solution was added. After vortex mixing for 15 s, samples were extracted with 5 mL of dichloromethane for 15 min on a shaker (VXR Vibrax, IKA, Germany). After centrifugation (Universal 32, Hettich, Germany) at 3000 rpm for 20 min, the organic layers were transferred into conical glass tubes and evaporated to dryness at 37 °C under a gentle stream of nitrogen. The residues were reconstituted in 100 μL of MeCN, vortexed for 30 s and 40 μL of this solution was injected into the HPLC system. The calibration curve constructed by plotting the ratio of the peak area of **(*****R*****)-7** [**(*****R*****)-AS-1**] to IS versus **(*****R*****)-7** [**(*****R*****)-AS-1**] concentrations was linear in the tested concentration ranges. The limit of quantification of the analytical method was 2 μg/mL (0.25 μg/g tissue). There were no interfering peaks observed in the chromatograms at the retention times of the analyte and IS. The assay was reproducible as indicated by coefficients of variation less than 10% for both intra- and interday assessments. The extraction efficiencies of **(*****R*****)-7** [**(*****R*****)-AS-1**] and IS were higher than 85%.

### Pharmacokinetic Data Analysis

Serum and brain *vs* time profiles were analyzed by the noncompartmental approach. The maximum concentration (*C*_max_) and the time to reach maximum concentration (*t*_max_) were obtained directly from individual concentration versus time profiles. The linear trapezoidal rule was employed to calculate the area under the concentration *vs* time curve (AUC) from the time of dosing to infinity (AUC_0–∞_). The terminal slope (λ*_z_*) was estimated by the linear regression and the terminal half-life (*t*_0.5λ*z*_) was calculated as ln 2/λ*_z_*. The volume of distribution based on the terminal phase (*V_z_*/*F*) was calculated as: dose/(λ*_z_* AUC_0–∞_) and clearance (CL/*F*) was obtained from the equation: Dose/AUC_0–∞_, where *F* is the fraction of dose absorbed. The mean residence time (MRT) was calculated as: AUMC_0–∞_/AUC_0–∞_, where AUMC is the area under the first moment curve.

### Radioligand Binding/Functional Assays

Binding/functional studies were carried out commercially in Cerep Laboratories (Poitiers, France) using testing procedures reported previously. The general information is listed in the Supporting Information.

#### SV2A Radioligand Binding Assay

The competitive radioligand-binding assay was essentially performed as previously described.^96,97^ The SV2A-containing preparations, either cell lysate of human SV2A-expressing CHO cells, or membrane preparations of mouse brain cortex, were incubated with the radioligand [^3^H]brivaracetam (3 nM) in the absence or presence of test compound for 4 h. Incubation was performed in 500 μL of Tris-buffer (50 mM Tris–HCl, pH 7.4) containing 2 mM MgCl_2_ at 4 °C on a rocking shaker. Nonspecific binding was determined in the presence of 1 mM levetiracetam. The test compounds were dissolved in dimethyl sulfoxide (DMSO) and further diluted in DMSO. The DMSO concentration in all vials was adjusted to 2%. The separation of bound and unbound radioligand was performed *via* rapid vacuum filtration using GF/C glass fiber filters that had been presoaked in Tris-buffer containing 0.1% polyethyleneimine for 30 min. The filters were rapidly washed three times with about 1 mL of Tris-buffer each and subsequently placed in a drying oven at 50 °C for 90 min. The filters were then transferred to scintillation vials and 2.5 mL of scintillation cocktail (ProSafe FC+, Meridian Biotechnologies Ltd.) was added. After an incubation period of at least 9 h, radioactivity was measured in a scintillation counter. Results were analyzed by GraphPad Prism 7.0.

### *In Vitro* ADME-Tox Studies

#### Permeability

Precoated PAMPA Plate System Gentest was provided by Corning, (Tewksbury, MA). Compound **(*****R*****)**-**7** [**(*****R*****)-AS-1**] was tested in a similar way to racemate **I**.^29^ The detailed procedure and proper formulas were described previously.^73^

#### Protein Binding Analyses

Protein binding analyses were performed according to a previously published protocol.^74^

#### Metabolic Stability

These assays were performed on human liver microsomes (HLMs) and mouse liver microsomes (MLMs), purchased from Sigma-Aldrich (St. Louis, MO), in a similar way to that described previously for racemate **I**.^29^

#### Glucuronidation

The reference compound 7-hydroxy-4-trifluoromethylcoumarin (HFC), UDP-glucuronic acid (UDPGA), and HLMs were obtained from Sigma-Aldrich (St. Louis, MO). **(*****R*****)**-**7** [**(*****R*****)-AS-1**] and HFC (both at final concentration 50 μM) were tested in the Tris–HCl buffer (100 mM, pH 7.4) containing HLMs (1 mg/mL), MgCl_2_ (2.5 mM) and UDPGA (2 mM). The reaction mixtures were incubated for 60 min. To stop the reaction the cold acetonitrile was added. The reaction mixtures were centrifuged, and the supernatants were analyzed next by UPLC-MS. The reference reactions were performed under the same conditions but without the addition of HLMs.

#### Influence on Recombinant Human CYP3A4, CYP2D6, and CYP2C9 P450 Cytochromes

The luminescent CYP3A4 P450-Glo and CYP2D6 P450-Glo assays and protocols were provided by Promega (Madison, WI).^98^ The procedures were provided by the manufacturer and were similar as previously reported for the racemate I.^29^

#### Antiproliferative/Hepatotoxic Assays on Human Embryonic Kidney (HEK-293) and/or Hepatoma HepG2 Cells

The test with the use of the HEK-293 cell line was performed as described previously.^99^ The procedures with the use of HepG2 cells for the estimation of hepatotoxicity were similar to that previously reported for the racemate I.^29^

#### Neurotoxicity/Neurogenesis Assay

The studies with the use of neuroblastoma SH-SY5Y cell line were performed in the same way as described previously for another pyrrolidine-2,5-dione derivative obtained in our research group.^36^

## Supporting Information Available

X-ray analysis (crystal structure determination, crystal structure analysis), additional glutamate transporter results in COS-7 cell lines and astrocytes, hCav3.2 QPatch assay, GlyRA1 IonFlux protocol, patch-clamp recordings of tonic NMDA currents, binding/functional/transporter assay data/information, spontaneous locomotor activity results in mice (i.p.), effect of repeated pretreatment on anxiety, depressive-like behavior, and spontaneous locomotor activity in mice subjected to the PTZ kindling, influence on latency time to first clonus in the *sc*PTZ test (i.p. and p.o. data), influence on the seizure-like behavior in the PTZ-induced zebrafish hyperlocomotion assay, ADME-Tox experimental data, chiral HPLC chromatograms, UPLC/MS and HRMS traces, ^1^H NMR spectra, and ^13^C NMR spectra (PDF)
**(*****R*****)-7** [**(*****R*****)-AS-1**] docked in site 1B onto EAAT2 (Figure 8A) (PDB)
**(*****R*****)-7** [**(*****R*****)-AS-1**] docked in site 1A onto EAAT2 (Figure 8B) (PDB)
**(*****R*****)-8** docked in site 1A onto EAAT2 (Figure 8C) (PDB)
(*R*)-GT949 docked in site 1A onto EAAT2 (Figure 8D) (PDB)
Comparison of the predicted binding poses of **(*****R*****)-7** [**(*****R*****)-AS-1**] *vs***(*****S*****)-7** (Figure S7C) (PDB)
Comparison of the predicted binding poses of **(*****R*****)-8***vs***(*****S*****)-8** (Figure S7D) (PDB)
Molecular formula strings of the final compounds together with biological data (XLSX)

## Supplementary Material

jm2c00534_si_001.pdf

jm2c00534_si_002.pdb

jm2c00534_si_003.pdb

jm2c00534_si_004.pdb

jm2c00534_si_005.pdb

jm2c00534_si_006.pdb

jm2c00534_si_007.pdb

## Abbreviations Used

ADME-Tox: absorption, distribution, metabolism, excretion, toxicity
ASDs: antiseizure drugs
CBD: cannabidiol
CNS: central nervous system
DCC: dicyclohexylcarbodiimide
DCM: dichloromethane
EAAT2: excitatory amino acid transporter 2
EEG: electroencephalography
ETX: ethosuximide
FDA: Food and Drug Administration
GABA: γ-aminobutyric acid
HLMs: human liver microsomes
HRMS: high-resolution mass spectroscopy
6 Hz: six-Hertz seizure test
LCS: lacosamide
LEV: levetiracetam
MeCN: acetonitrile
MES: maximal electroshock seizure test
MeOH: methanol
mGluR: metabotropic glutamate receptor
MLMs: mouse liver microsomes
PAM: positive allosteric modulator
PAMPA: parallel artificial membrane permeability assay
PI: protective index (TD_50_/ED_50_)
PT: pretreatment time
PTZ: pentylenetetrazole
*sc*PTZ: subcutaneous pentylenetetrazole seizure test
SV2A: synaptic vesicle glycoprotein 2A
TTX: tetrodotoxin
VPA: valproic acid

## References

[ref1] Ngugi A. K.; Bottomley C.; Kleinschmidt I.; Sander J. W.; Newton C. R. Estimation of the Burden of Active and Life-Time Epilepsy: A Meta-Analytic Approach. Epilepsia 2010, 51, 883–890. 10.1111/j.1528-1167.2009.02481.x. 20067507 PMC3410521

[ref2] Chen Z.; Brodie M. J.; Liew D.; Kwan P. Treatment Outcomes in Patients With Newly Diagnosed Epilepsy Treated With Established and New Antiepileptic Drugs: A 30-Year Longitudinal Cohort Study. JAMA Neurol. 2018, 75, 279–286. 10.1001/jamaneurol.2017.3949. 29279892 PMC5885858

[ref3] Kwan P.; Arzimanoglou A.; Berg A. T.; Brodie M. J.; Allen Hauser W.; Mathern G.; Moshé S. L.; Perucca E.; Wiebe S.; French J. Definition of Drug Resistant Epilepsy: Consensus Proposal by the Ad Hoc Task Force of the ILAE Commission on Therapeutic Strategies. Epilepsia 2009, 51, 1069–1077. 10.1111/j.1528-1167.2009.02397.x. 19889013

[ref4] Löscher W.; Klein P. The Pharmacology and Clinical Efficacy of Antiseizure Medications: From Bromide Salts to Cenobamate and Beyond. CNS Drugs 2021, 35, 935–963. 10.1007/s40263-021-00827-8. 34145528 PMC8408078

[ref5] Green J. L.; Dos Santos W. F.; Fontana A. C. K. Role of Glutamate Excitotoxicity and Glutamate Transporter EAAT2 in Epilepsy: Opportunities for Novel Therapeutics Development. Biochem. Pharmacol. 2021, 193, 114786 10.1016/j.bcp.2021.114786. 34571003 PMC8605998

[ref6] Suchak S. K.; Baloyianni N. V.; Perkinton M. S.; Williams R. J.; Meldrum B. S.; Rattray M. The “glial” Glutamate Transporter, EAAT2 (Glt-1) Accounts for High Affinity Glutamate Uptake into Adult Rodent Nerve Endings. J. Neurochem. 2003, 84, 522–532. 10.1046/j.1471-4159.2003.01553.x. 12558972

[ref7] Sheldon A. L.; Robinson M. B. The Role of Glutamate Transporters in Neurodegenerative Diseases and Potential Opportunities for Intervention. Neurochem. Int. 2007, 51, 333–355. 10.1016/j.neuint.2007.03.012. 17517448 PMC2075474

[ref8] Kim K.; Lee S.-G.; Kegelman T. P.; Su Z.-Z.; Das S. K.; Dash R.; Dasgupta S.; Barral P. M.; Hedvat M.; Diaz P.; Reed J. C.; Stebbins J. L.; Pellecchia M.; Sarkar D.; Fisher P. B. Role of Excitatory Amino Acid Transporter-2 (EAAT2) and Glutamate in Neurodegeneration: Opportunities for Developing Novel Therapeutics. J. Cell. Physiol. 2011, 226, 2484–2493. 10.1002/jcp.22609. 21792905 PMC3130100

[ref9] Fontana A. C. K. Current Approaches to Enhance Glutamate Transporter Function and Expression. J. Neurochem. 2015, 134, 982–1007. 10.1111/jnc.13200. 26096891

[ref10] Pajarillo E.; Rizor A.; Lee J.; Aschner M.; Lee E. The Role of Astrocytic Glutamate Transporters GLT-1 and GLAST in Neurological Disorders: Potential Targets for Neurotherapeutics. Neuropharmacology 2019, 161, 107559 10.1016/j.neuropharm.2019.03.002. 30851309 PMC6731169

[ref11] Rosenblum L. T.; Trotti D. EAAT2 and the Molecular Signature of Amyotrophic Lateral Sclerosis. Adv. Neurobiol. 2017, 16, 117–136. 10.1007/978-3-319-55769-4_6. 28828608 PMC6668619

[ref12] John C. S.; Sypek E. I.; Carlezon W. A.; Cohen B. M.; Öngür D.; Bechtholt A. J. Blockade of the GLT-1 Transporter in the Central Nucleus of the Amygdala Induces Both Anxiety and Depressive-Like Symptoms. Neuropsychopharmacology 2015, 40, 1700–1708. 10.1038/npp.2015.16. 25586634 PMC4915252

[ref13] Zaitsev A. V.; Smolensky I. V.; Jorratt P.; Ovsepian S. V. Neurobiology, Functions, and Relevance of Excitatory Amino Acid Transporters (EAATs) to Treatment of Refractory Epilepsy. CNS Drugs 2020, 34, 1089–1103. 10.1007/s40263-020-00764-y. 32926322

[ref14] During M. J.; Spencer D. D. Extracellular Hippocampal Glutamate and Spontaneous Seizure in the Conscious Human Brain. Lancet 1993, 341, 1607–1610. 10.1016/0140-6736(93)90754-5. 8099987

[ref15] Cavus I.; Kasoff W. S.; Cassaday M. P.; Jacob R.; Gueorguieva R.; Sherwin R. S.; Krystal J. H.; Spencer D. D.; Abi-Saab W. M. Extracellular Metabolites in the Cortex and Hippocampus of Epileptic Patients. Ann. Neurol. 2005, 57, 226–235. 10.1002/ana.20380. 15668975

[ref16] Thomas P. M.; Phillips J. P.; O’Connor W. T. Hippocampal Microdialysis during Spontaneous Intraoperative Epileptiform Activity. Acta Neurochir. 2004, 146, 143–151. 10.1007/s00701-003-0189-9. 14963746

[ref17] Mathern G. W.; Mendoza D.; Lozada A.; Pretorius J. K.; Dehnes Y.; Danbolt N. C.; Nelson N.; Leite J. P.; Chimelli L.; Born D. E.; Sakamoto A. C.; Assirati J. A.; Fried I.; Peacock W. J.; Ojemann G. A.; Adelson P. D. Hippocampal GABA and Glutamate Transporter Immunoreactivity in Patients with Temporal Lobe Epilepsy. Neurology 1999, 52, 453–472. 10.1212/wnl.52.3.453. 10025773

[ref18] Proper E. A.; Hoogland G.; Kappen S. M.; Jansen G. H.; Rensen M. G. A.; Schrama L. H.; van Veelen C. W. M.; van Rijen P. C.; van Nieuwenhuizen O.; Gispen W. H.; de Graan P. N. E. Distribution of Glutamate Transporters in the Hippocampus of Patients with Pharmaco-resistant Temporal Lobe Epilepsy. Brain 2002, 125, 32–43. 10.1093/brain/awf001. 11834591

[ref19] Hoogland G.; van Oort R. J.; Proper E. A.; Jansen G. H.; van Rijen P. C.; van Veelen C. W. M.; van Nieuwenhuizen O.; Troost D.; de Graan P. N. E. Alternative Splicing of Glutamate Transporter EAAT2 RNA in Neocortex and Hippocampus of Temporal Lobe Epilepsy Patients. Epilepsy Res. 2004, 59, 75–82. 10.1016/j.eplepsyres.2004.03.003. 15246112

[ref20] Sarac S.; Afzal S.; Broholm H.; Madsen F. F.; Ploug T.; Laursen H. Excitatory Amino Acid Transporters EAAT-1 and EAAT-2 in Temporal Lobe and Hippocampus in Intractable Temporal Lobe Epilepsy. APMIS 2009, 117, 291–301. 10.1111/j.1600-0463.2009.02443.x. 19338517

[ref21] Bjørnsen L. P.; Eid T.; Holmseth S.; Danbolt N. C.; Spencer D. D.; de Lanerolle N. C. Changes in Glial Glutamate Transporters in Human Epileptogenic Hippocampus: Inadequate Explanation for High Extracellular Glutamate during Seizures. Neurobiol. Dis. 2007, 25, 319–330. 10.1016/j.nbd.2006.09.014. 17112731

[ref22] Kong Q.; Takahashi K.; Schulte D.; Stouffer N.; Lin Y.; Lin C.-L. G. Increased Glial Glutamate Transporter EAAT2 Expression Reduces Epileptogenic Processes Following Pilocarpine-Induced Status Epilepticus. Neurobiol. Dis. 2012, 47, 145–154. 10.1016/j.nbd.2012.03.032. 22513140 PMC3572547

[ref23] Kong Q.; Chang L.-C.; Takahashi K.; Liu Q.; Schulte D. A.; Lai L.; Ibabao B.; Lin Y.; Stouffer N.; Mukhopadhyay C. D.; Xing X.; Seyb K. I.; Cuny G. D.; Glicksman M. A.; Lin C.-L. G. Small-Molecule Activator of Glutamate Transporter EAAT2 Translation Provides Neuroprotection. J. Clin. Invest. 2014, 124, 1255–1267. 10.1172/JCI66163. 24569372 PMC3938250

[ref24] Jelenkovic A. V.; Jovanovic M. D.; Stanimirovic D. D.; Bokonjic D. D.; Ocic G. G.; Boskovic B. S. Beneficial Effects of Ceftriaxone against Pentylenetetrazole-Evoked Convulsions. Exp. Biol. Med. 2008, 233, 1389–1394. 10.3181/0803-RM-83. 18703755

[ref25] Han B.; Salituro F. G.; Blanco M.-J. Impact of Allosteric Modulation in Drug Discovery: Innovation in Emerging Chemical Modalities. ACS Med. Chem. Lett. 2020, 11, 1810–1819. 10.1021/acsmedchemlett.9b00655. 33062158 PMC7549105

[ref26] Abram M.; Jakubiec M.; Kamiński K. Chirality as an Important Factor for the Development of New Antiepileptic Drugs. ChemMedChem 2019, 14, 1744–1761. 10.1002/cmdc.201900367. 31476107

[ref27] Latimer D. R.; Edinoff A. N.; Ruff R. D.; Rooney K. C.; Penny K. M.; Patel S. B.; Sabbenahalli S.; Kaye A. M.; Cornett E. M.; Viswanath O.; Urits I.; Kaye A. D. Cenobamate, a Sodium Channel Inhibitor and Positive Allosteric Modulator of GABAA Ion Channels, for Partial Onset Seizures in Adults: A Comprehensive Review and Clinical Implications. Neurol. Int. 2021, 13, 252–265. 10.3390/neurolint13020026. 34207493 PMC8293325

[ref28] Kamiński K.; Rapacz A.; Łuszczki J. J.; Latacz G.; Obniska J.; Kieć-Kononowicz K.; Filipek B. Design, Synthesis and Biological Evaluation of New Hybrid Anticonvulsants Derived from N-Benzyl-2-(2,5-Dioxopyrrolidin-1-Yl)Propanamide and 2-(2,5-Dioxopyrrolidin-1-Yl)Butanamide Derivatives. Bioorg. Med. Chem. 2015, 23, 2548–2561. 10.1016/j.bmc.2015.03.038. 25868743

[ref29] Kamiński K.; Socała K.; Zagaja M.; Andres-Mach M.; Abram M.; Jakubiec M.; Pieróg M.; Nieoczym D.; Rapacz A.; Gawel K.; Esguerra C. V.; Latacz G.; Lubelska A.; Szulczyk B.; Szewczyk A.; Łuszczki J. J.; Wlaź P. N-Benzyl-(2,5-Dioxopyrrolidin-1-Yl)Propanamide (AS-1) with Hybrid Structure as a Candidate for a Broad-Spectrum Antiepileptic Drug. Neurotherapeutics 2020, 17, 309–328. 10.1007/s13311-019-00773-w. 31486023 PMC7007424

[ref30] Parsons W. H.; Rutland N. T.; Crainic J. A.; Cardozo J. M.; Chow A. S.; Andrews C. L.; Sheehan B. K. Development of Succinimide-Based Inhibitors for the Mitochondrial Rhomboid Protease PARL. Bioorg. Med. Chem. Lett. 2021, 49, 128290 10.1016/j.bmcl.2021.128290. 34311087

[ref31] Zhao Z.; Yue J.; Ji X.; Nian M.; Kang K.; Qiao H.; Zheng X. Research Progress in Biological Activities of Succinimide Derivatives. Bioorg. Chem. 2021, 108, 104557 10.1016/j.bioorg.2020.104557. 33376010

[ref32] Abram M.; Zagaja M.; Mogilski S.; Andres-Mach M.; Latacz G.; Baś S.; Łuszczki J. J.; Kieć-Kononowicz K.; Kamiński K. Multifunctional Hybrid Compounds Derived from 2-(2,5-Dioxopyrrolidin-1-Yl)-3-Methoxypropanamides with Anticonvulsant and Antinociceptive Properties. J. Med. Chem. 2017, 60, 8565–8579. 10.1021/acs.jmedchem.7b01114. 28934547

[ref33] Abram M.; Rapacz A.; Mogilski S.; Latacz G.; Lubelska A.; Kamiński R. M.; Kamiński K. Multitargeted Compounds Derived from (2,5-Dioxopyrrolidin-1-Yl)(Phenyl)-Acetamides as Candidates for Effective Anticonvulsant and Antinociceptive Agents. ACS Chem. Neurosci. 2020, 11, 1996–2008. 10.1021/acschemneuro.0c00257. 32479058

[ref34] Kamiński K.; Mogilski S.; Abram M.; Rapacz A.; Latacz G.; Szulczyk B.; Walczak M.; Kuś K.; Matyjaszczyk K.; Kamiński R. M. KA-104, a New Multitargeted Anticonvulsant with Potent Antinociceptive Activity in Preclinical Models. Epilepsia 2020, 61, 2119–2128. 10.1111/epi.16669. 32929733

[ref35] Abram M.; Rapacz A.; Latacz G.; Szulczyk B.; Kalinowska-Tłuścik J.; Otto-Ślusarczyk D.; Struga M.; Kamiński R. M.; Kamiński K. Asymmetric Synthesis and in Vivo/in Vitro Characterization of New Hybrid Anticonvulsants Derived from (2,5-Dioxopyrrolidin-1-Yl)Phenylacetamides. Bioorg. Chem. 2021, 109, 104751 10.1016/j.bioorg.2021.104751. 33647745

[ref36] Abram M.; Jakubiec M.; Rapacz A.; Mogilski S.; Latacz G.; Szulczyk B.; Szafarz M.; Socała K.; Nieoczym D.; Wyska E.; Wlaź P.; Kamiński R. M.; Kamiński K. Identification of New Compounds with Anticonvulsant and Antinociceptive Properties in a Group of 3-Substituted (2,5-Dioxo-Pyrrolidin-1-Yl)(Phenyl)-Acetamides. Int. J. Mol. Sci. 2021, 22, 13092 10.3390/ijms222313092. 34884898 PMC8658016

[ref37] Rapacz A.; Kamiński K.; Obniska J.; Koczurkiewicz P.; Pękala E.; Filipek B. Analgesic, Antiallodynic, and Anticonvulsant Activity of Novel Hybrid Molecules Derived from N-Benzyl-2-(2,5-Dioxopyrrolidin-1-Yl)Propanamide and 2-(2,5-Dioxopyrrolidin-1-Yl)Butanamide in Animal Models of Pain and Epilepsy. Naunyn-Schmiedeberg’s Arch. Pharmacol. 2017, 390, 567–579. 10.1007/s00210-017-1358-3. 28188357 PMC5411412

[ref38] Löscher W. Single-Target Versus Multi-Target Drugs Versus Combinations of Drugs With Multiple Targets: Preclinical and Clinical Evidence for the Treatment or Prevention of Epilepsy. Front. Pharmacol. 2021, 12, 730257 10.3389/fphar.2021.730257. 34776956 PMC8580162

[ref39] Margineanu D. G. Systems Biology, Complexity, and the Impact on Antiepileptic Drug Discovery. Epilepsy Behav. 2014, 38, 131–142. 10.1016/j.yebeh.2013.08.029. 24090772

[ref40] Smith M.; Wilcox K. S.; White H. S. Discovery of Antiepileptic Drugs. Neurotherapeutics 2007, 4, 12–17. 10.1016/j.nurt.2006.11.009. 17199014 PMC7479710

[ref41] Patra P. H.; Barker-Haliski M.; White H. S.; Whalley B. J.; Glyn S.; Sandhu H.; Jones N.; Bazelot M.; Williams C. M.; McNeish A. J. Cannabidiol Reduces Seizures and Associated Behavioral Comorbidities in a Range of Animal Seizure and Epilepsy Models. Epilepsia 2019, 60, 303–314. 10.1111/epi.14629. 30588604 PMC6378611

[ref42] Desiraju G.; Steiner T. The Weak Hydrogen Bond: In Structural Chemistry and Biology. In International Union of Crystallography, Monographs on Crystallography, 9; Oxford University Press: Oxford, 2001.

[ref43] Pietruś W.; Kurczab R.; Kafel R.; Machalska E.; Kalinowska-Tłuścik J.; Hogendorf A.; Żylewski M.; Baranska M.; Bojarski A. J. How Can Fluorine Directly and Indirectly Affect the Hydrogen Bonding in Molecular Systems? – A Case Study for Monofluoroanilines. Spectrochim. Acta, Part A 2021, 252, 119536 10.1016/j.saa.2021.119536. 33588362

[ref44] Pietruś W.; Kurczab R.; Kalinowska-Tłuścik J.; Machalska E.; Golonka D.; Barańska M.; Bojarski A. J. Influence of Fluorine Substitution on Nonbonding Interactions in Selected Para-Halogeno Anilines. ChemPhysChem 2021, 22, 2115–2127. 10.1002/cphc.202100383. 34310822

[ref45] Löscher W. Critical Review of Current Animal Models of Seizures and Epilepsy Used in the Discovery and Development of New Antiepileptic Drugs. Seizure 2011, 20, 359–368. 10.1016/j.seizure.2011.01.003. 21292505

[ref46] Metcalf C. S.; West P. J.; Thomson K.; Edwards S.; Smith M. D.; White H. S.; Wilcox K. S. Development and Pharmacological Characterization of the Rat 6 Hz Model of Partial Seizures. Epilepsia 2017, 58, 1073–1084. 10.1111/epi.13764. 28449218 PMC5469205

[ref47] Barton M. E.; Klein B. D.; Wolf H. H.; White H. S. Pharmacological Characterization of the 6 Hz Psychomotor Seizure Model of Partial Epilepsy. Epilepsy Res. 2001, 47, 217–227. 10.1016/s0920-1211(01)00302-3. 11738929

[ref48] Klitgaard H.; Matagne A.; Schachter S. C.; White H. S. Animal and Translational Models of the Epilepsies. Animal and Translational Models for CNS Drug Discovery; Academic Press, 2008; pp 311–335.

[ref49] Gawel K.; Kukula-Koch W.; Banono N. S.; Nieoczym D.; Targowska-Duda K. M.; Czernicka L.; Parada-Turska J.; Esguerra C. V. 6-Gingerol, a Major Constituent of Zingiber Officinale Rhizoma, Exerts Anticonvulsant Activity in the Pentylenetetrazole-Induced Seizure Model in Larval Zebrafish. Int. J. Mol. Sci. 2021, 22, 7745 10.3390/ijms22147745. 34299361 PMC8305044

[ref50] Gawel K.; Kukula-Koch W.; Nieoczym D.; Stepnik K.; van der Ent W.; Banono N. S.; Tarabasz D.; Turski W. A.; Esguerra C. V. The Influence of Palmatine Isolated from Berberis Sibirica Radix on Pentylenetetrazole-Induced Seizures in Zebrafish. Cells 2020, 9, 1233 10.3390/cells9051233. PMC7290958 32429356

[ref51] Bertoncello K. T.; Bonan C. D. Zebrafish as a Tool for the Discovery of Anticonvulsant Compounds from Botanical Constituents. Eur. J. Pharmacol. 2021, 908, 174342 10.1016/j.ejphar.2021.174342. 34265297

[ref52] Kim S. S.; Kan H.; Hwang K.-S.; Yang J. Y.; Son Y.; Shin D.-S.; Lee B. H.; Ahn S. H.; Ahn J. H.; Cho S.-H.; Bae M. A. Neurochemical Effects of 4-(2Chloro-4-Fluorobenzyl)-3-(2-Thienyl)-1,2,4-Oxadiazol-5(4H)-One in the Pentylenetetrazole (PTZ)-Induced Epileptic Seizure Zebrafish Model. Int. J. Mol. Sci. 2021, 22, 1285 10.3390/ijms22031285. 33525453 PMC7865321

[ref53] Yaksi E.; Jamali A.; Diaz Verdugo C.; Jurisch-Yaksi N. Past, Present and Future of Zebrafish in Epilepsy Research. FEBS J. 2021, 288, 7243–7255. 10.1111/febs.15694. 33394550

[ref54] Flores L.; Kemp S.; Colbeck K.; Moran N.; Quirk J.; Ramkolea P.; von Oertzen T. J.; Nashef L.; Richardson M. P.; Goulding P.; Elwes R. Clinical Experience with Oral Lacosamide as Adjunctive Therapy in Adult Patients with Uncontrolled Epilepsy: A Multicentre Study in Epilepsy Clinics in the United Kingdom (UK). Seizure 2012, 21, 512–517. 10.1016/j.seizure.2012.05.005. 22698379

[ref55] Kennedy G. M.; Lhatoo S. D. CNS Adverse Events Associated with Antiepileptic Drugs. CNS Drugs 2008, 22, 739–760. 10.2165/00023210-200822090-00003. 18698874

[ref56] Brodtkorb E.; Klees T. M.; Nakken K. O.; Lossius R.; Johannessen S. I. Levetiracetam in Adult Patients with and without Learning Disability: Focus on Behavioral Adverse Effects. Epilepsy Behav. 2004, 5, 231–235. 10.1016/j.yebeh.2003.12.005. 15123025

[ref57] Svob Strac D.; Pivac N.; Smolders I. J.; Fogel W. A.; De Deurwaerdere P.; Di Giovanni G. Monoaminergic Mechanisms in Epilepsy May Offer Innovative Therapeutic Opportunity for Monoaminergic Multi-Target Drugs. Front. Neurosci. 2016, 10, 492 10.3389/fnins.2016.00492. 27891070 PMC5102907

[ref58] Cheng M. H.; Bahar I. Monoamine Transporters: Structure, Intrinsic Dynamics and Allosteric Regulation. Nat. Struct. Mol. Biol. 2019, 26, 545–556. 10.1038/s41594-019-0253-7. 31270469 PMC6712983

[ref59] Kortagere S.; Mortensen O. V.; Xia J.; Lester W.; Fang Y.; Srikanth Y.; Salvino J. M.; Fontana A. C. K. Identification of Novel Allosteric Modulators of Glutamate Transporter EAAT2. ACS Chem. Neurosci. 2018, 9, 522–534. 10.1021/acschemneuro.7b00308. 29140675

[ref60] Falcucci R. M.; Wertz R.; Green J. L.; Meucci O.; Salvino J.; Fontana A. C. K. Novel Positive Allosteric Modulators of Glutamate Transport Have Neuroprotective Properties in an in Vitro Excitotoxic Model. ACS Chem. Neurosci. 2019, 10, 3437–3453. 10.1021/acschemneuro.9b00061. 31257852 PMC7099866

[ref61] Akyuz N.; Altman R. B.; Blanchard S. C.; Boudker O. Transport Dynamics in a Glutamate Transporter Homologue. Nature 2013, 502, 114–118. 10.1038/nature12265. 23792560 PMC3829612

[ref62] Akyuz N.; Georgieva E. R.; Zhou Z.; Stolzenberg S.; Cuendet M. A.; Khelashvili G.; Altman R. B.; Terry D. S.; Freed J. H.; Weinstein H.; Boudker O.; Blanchard S. C. Transport Domain Unlocking Sets the Uptake Rate of an Aspartate Transporter. Nature 2015, 518, 68–73. 10.1038/nature14158. 25652997 PMC4351760

[ref63] Reyes N.; Ginter C.; Boudker O. Transport Mechanism of a Bacterial Homologue of Glutamate Transporters. Nature 2009, 462, 880–885. 10.1038/nature08616. 19924125 PMC2934767

[ref64] Canul-Tec J. C.; Assal R.; Cirri E.; Legrand P.; Brier S.; Chamot-Rooke J.; Reyes N. Structure and Allosteric Inhibition of Excitatory Amino Acid Transporter 1. Nature 2017, 544, 446–451. 10.1038/nature22064. 28424515 PMC5410168

[ref65] Jiang J.; Shrivastava I. H.; Watts S. D.; Bahar I.; Amara S. G. Large Collective Motions Regulate the Functional Properties of Glutamate Transporter Trimers. Proc. Natl. Acad. Sci. U.S.A. 2011, 108, 15141–15146. 10.1073/pnas.1112216108. 21876140 PMC3174670

[ref66] Shrivastava I. H.; Jiang J.; Amara S. G.; Bahar I. Time-Resolved Mechanism of Extracellular Gate Opening and Substrate Binding in a Glutamate Transporter. J. Biol. Chem. 2008, 283, 28680–28690. 10.1074/jbc.M800889200. 18678877 PMC2568915

[ref67] Waterhouse A.; Bertoni M.; Bienert S.; Studer G.; Tauriello G.; Gumienny R.; Heer F. T.; de Beer T. A. P.; Rempfer C.; Bordoli L.; Lepore R.; Schwede T. SWISS-MODEL: Homology Modelling of Protein Structures and Complexes. Nucleic Acids Res. 2018, 46, W296–W303. 10.1093/nar/gky427. 29788355 PMC6030848

[ref68] Qiu B.; Matthies D.; Fortea E.; Yu Z.; Boudker O. Cryo-EM Structures of Excitatory Amino Acid Transporter 3 Visualize Coupled Substrate, Sodium, and Proton Binding and Transport. Sci. Adv. 2021 7 (), eabf5814. 10.1126/sciadv.abf5814. 33658209 PMC7929514

[ref69] Cheng M. H.; Torres-Salazar D.; Gonzalez-Suarez A. D.; Amara S. G.; Bahar I. Substrate Transport and Anion Permeation Proceed through Distinct Pathways in Glutamate Transporters. eLife 2017, 6, e25850 10.7554/eLife.25850. 28569666 PMC5472439

[ref70] Mortensen O. V.; Liberato J. L.; Coutinho-Netto J.; Dos Santos W. F.; Fontana A. C. K. Molecular Determinants of Transport Stimulation of EAAT2 Are Located at Interface between the Trimerization and Substrate Transport Domains. J. Neurochem. 2015, 133, 199–210. 10.1111/jnc.13047. 25626691

[ref71] Svensson K. A.; Hao J.; Bruns R. F. Positive Allosteric Modulators of the Dopamine D1 Receptor: A New Mechanism for the Treatment of Neuropsychiatric Disorders. In Neuropsychotherapeutics; Witkin J. M., Ed.; Advances in Pharmacology; Academic Press, 2019; Chapter 9, Vol. 86, pp 273–305. 10.1016/bs.apha.2019.06.001 31378255

[ref72] Argyrousi E. K.; Heckman P. R. A.; Prickaerts J. Glutamate Signalling in Object Novelty Recognition Memory Tests. In Handbook of Object Novelty Recognition; Ennaceur A.; de Souza Silva M. A., Eds.; Handbook of Behavioral Neuroscience; Elsevier, 2018; Chapter 35, Vol. 27, pp 541–551.

[ref73] Chen X.; Murawski A.; Patel K.; Crespi C. L.; Balimane P. V. A Novel Design of Artificial Membrane for Improving the PAMPA Model. Pharm. Res. 2008, 25, 1511–1520. 10.1007/s11095-007-9517-8. 18185985

[ref74] Lubelska A.; Latacz G.; Jastrzębska-Więsek M.; Kotańska M.; Kurczab R.; Partyka A.; Marć M. A.; Wilczyńska D.; Doroz-Płonka A.; Łażewska D.; Wesołowska A.; Kieć-Kononowicz K.; Handzlik J. Are the Hydantoin-1,3,5-Triazine 5-HT6R Ligands a Hope to a Find New Procognitive and Anti-Obesity Drug? Considerations Based on Primary In Vivo Assays and ADME-Tox Profile In Vitro. Molecules 2019, 24, 4472 10.3390/molecules24244472. PMC6943527 31817628

[ref75] Andres-Mach M.; Szewczyk A.; Zagaja M.; Szala-Rycaj J.; Lemieszek M. K.; Maj M.; Abram M.; Kaminski K. Preclinical Assessment of a New Hybrid Compound C11 Efficacy on Neurogenesis and Cognitive Functions after Pilocarpine Induced Status Epilepticus in Mice. Int. J. Mol. Sci. 2021, 22, 3240 10.3390/ijms22063240. 33810180 PMC8004689

[ref76] Alavi M. S.; Negah S. S.; Ghorbani A.; Hosseini A.; Sadeghnia H. R. Levetiracetam Promoted Rat Embryonic Neurogenesis via NMDA Receptor-Mediated Mechanism in Vitro. Life Sci. 2021, 284, 119923 10.1016/j.lfs.2021.119923. 34481865

[ref77] Zagaja M.; Haratym-Maj A.; Szewczyk A.; Rola R.; Maj M.; Łuszczki J. J.; Andres-Mach M. Levetiracetam Combined with ACEA, Highly Selective Cannabinoid CB1 Receptor Agonist Changes Neurogenesis in Mouse Brain. Neurosci. Lett. 2019, 696, 79–86. 10.1016/j.neulet.2018.12.016. 30552944

[ref78] Yan B. C.; Shen H.; Zhang Y.; Zhu X.; Wang J.; Xu P.; Jiang D.; Yu X. The Antiepileptic Drug Levetiracetam Promotes Neuroblast Differentiation and Expression of Superoxide Dismutase in the Mouse Hippocampal Dentate Gyrus via PI3K/Akt Signalling. Neurosci. Lett. 2018, 662, 84–90. 10.1016/j.neulet.2017.10.010. 29024726

[ref79] Sheng H. P.; Huggins R. A. A Review of Body Composition Studies with Emphasis on Total Body Water and Fat. Am. J. Clin. Nutr. 1979, 32, 630–647. 10.1093/ajcn/32.3.630. 420154

[ref80] Brown R. P.; Delp M. D.; Lindstedt S. L.; Rhomberg L. R.; Beliles R. P. Physiological Parameter Values for Physiologically Based Pharmacokinetic Models. Toxicol. Ind. Health 1997, 13, 407–484. 10.1177/074823379701300401. 9249929

[ref81] Nussinov R.; Tsai C.-J. Allostery in Disease and in Drug Discovery. Cell 2013, 153, 293–305. 10.1016/j.cell.2013.03.034. 23582321

[ref82] Lu S.; Li S.; Zhang J. Harnessing Allostery: A Novel Approach to Drug Discovery. Med. Res. Rev. 2014, 34, 1242–1285. 10.1002/med.21317. 24827416

[ref83] Ghanizadeh A.; Berk M. Beta-Lactam Antibiotics as a Possible Novel Therapy for Managing Epilepsy and Autism, a Case Report and Review of Literature. Iran. J. Child Neurol. 2015, 9, 99–102. 25767546 PMC4322506

[ref84] Henter I. D.; Park L. T.; Zarate C. A. Novel Glutamatergic Modulators for the Treatment of Mood Disorders: Current Status. CNS Drugs 2021, 35, 527–543. 10.1007/s40263-021-00816-x. 33904154 PMC8201267

[ref85] Yousuf M. S.; Kerr B. J. The Role of Regulatory Transporters in Neuropathic Pain. Pharmacological Mechanisms and the Modulation of Pain, Advances in Pharmacology; Elsevier Inc., 2016; Vol. 75, pp 245–271. 10.1016/bs.apha.2015.12.003 26920015

[ref86] Löscher W.; Fiedler M. The Role of Technical, Biological, and Pharmacological Factors in the Laboratory Evaluation of Anticonvulsant Drugs. VII. Seasonal Influences on Anticonvulsant Drug Actions in Mouse Models of Generalized Seizures. Epilepsy Res. 2000, 38, 231–248. 10.1016/S0920-1211(99)00095-9. 10642049

[ref87] Kaminski R. M.; Livingood M. R.; Rogawski M. A. Allopregnanolone Analogs That Positively Modulate GABAA Receptors Protect against Partial Seizures Induced by 6-Hz Electrical Stimulation in Mice. Epilepsia 2004, 45, 864–867. 10.1111/j.0013-9580.2004.04504.x. 15230714

[ref88] Łuszczki J. J. Dose-Response Relationship Analysis of Pregabalin Doses and Their Antinociceptive Effects in Hot-Plate Test in Mice. Pharmacol. Rep. 2010, 62, 942–948. 10.1016/s1734-1140(10)70355-8. 21098878

[ref89] Łączkowski K. Z.; Sałat K.; Misiura K.; Podkowa A.; Malikowska N. Synthesis and Anticonvulsant Activities of Novel 2-(Cyclopentylmethylene)Hydrazinyl-1,3-Thiazoles in Mouse Models of Seizures. J. Enzyme Inhib. Med. Chem. 2016, 31, 1576–1582. 10.3109/14756366.2016.1158172. 27052195

[ref90] Litchfield J. T.; Wilcoxon F. A Simplified Method of Evaluating Dose-Effect Experiments. J. Pharmacol. Exp. Ther. 1949, 96, 99–113. 18152921

[ref91] Mogilski S.; Kubacka M.; Łażewska D.; Więcek M.; Głuch-Lutwin M.; Tyszka-Czochara M.; Bukowska-Strakova K.; Filipek B.; Kieć-Kononowicz K. Aryl-1,3,5-Triazine Ligands of Histamine H4 Receptor Attenuate Inflammatory and Nociceptive Response to Carrageen, Zymosan and Lipopolysaccharide. Inflamm. Res. 2017, 66, 79–95. 10.1007/s00011-016-0997-z. 27766379 PMC5209447

[ref92] Socała K.; Mogilski S.; Pieróg M.; Nieoczym D.; Abram M.; Szulczyk B.; Lubelska A.; Latacz G.; Doboszewska U.; Wlaź P.; Kamiński K. KA-11, a Novel Pyrrolidine-2,5-Dione Derived Broad-Spectrum Anticonvulsant: Its Antiepileptogenic, Antinociceptive Properties and in Vitro Characterization. ACS Chem. Neurosci. 2019, 10, 636–648. 10.1021/acschemneuro.8b00476. 30247871

[ref93] Trott O.; Olson A. J. AutoDock Vina: Improving the Speed and Accuracy of Docking with a New Scoring Function, Efficient Optimization, and Multithreading. J. Comput. Chem. 2010, 31, 455–461. 10.1002/jcc.21334. 19499576 PMC3041641

[ref94] Timple J. M. V.; Magalhães L. G.; Souza Rezende K. C.; Pereira A. C.; Cunha W. R.; Andrade e Silva M. L.; Mortensen O. V.; Fontana A. C. K. The Lignan (-)-Hinokinin Displays Modulatory Effects on Human Monoamine and GABA Transporter Activities. J. Nat. Prod. 2013, 76, 1889–1895. 10.1021/np400452n. 24112084

[ref95] Forster Y. M.; Green J. L.; Khatiwada A.; Liberato J. L.; Narayana Reddy P. A.; Salvino J. M.; Bienz S.; Bigler L.; Dos Santos W. F.; Karklin Fontana A. C. Elucidation of the Structure and Synthesis of Neuroprotective Low Molecular Mass Components of the Parawixia Bistriata Spider Venom. ACS Chem. Neurosci. 2020, 11, 1573–1596. 10.1021/acschemneuro.0c00007. 32343555

[ref96] Danish A.; Namasivayam V.; Schiedel A. C.; Müller C. E. Interaction of Approved Drugs with Synaptic Vesicle Protein 2A. Arch. Pharm. 2017, 350, 1700003 10.1002/ardp.201700003. 28220535

[ref97] Hildenbrand S.; Schoch S.; von Lehe M.; Surges R.; Müller C. E. Tritium-Labeled Brivaracetam with High Specific Activity: Preparation, Characterization and Application in Human Brain Samples. ChemMedChem 2012, 7, 1369–1374. 10.1002/cmdc.201200183. 22740425

[ref98] Cali J. J.; Ma D.; Sobol M.; Simpson D. J.; Frackman S.; Good T. D.; Daily W. J.; Liu D. Luminogenic Cytochrome P450 Assays. Expert Opin. Drug Metab. Toxicol. 2006, 2, 629–645. 10.1517/17425255.2.4.629. 16859410

[ref99] Latacz G.; Lubelska A.; Jastrzębska-Więsek M.; Partyka A.; Marć M. A.; Satała G.; Wilczyńska D.; Kotańska M.; Więcek M.; Kamińska K.; Wesołowska A.; Kieć-Kononowicz K.; Handzlik J. The 1,3,5-Triazine Derivatives as Innovative Chemical Family of 5-HT6 Serotonin Receptor Agents with Therapeutic Perspectives for Cognitive Impairment. Int. J. Mol. Sci. 2019, 20, 3420 10.3390/ijms20143420. PMC6678253 31336820

