# Supplementary material for: Discovery of (R)-N-Benzyl-2-(2,5-dioxopyrrolidin-1-yl)propanamide [(R)-AS-1], a Novel Orally Bioavailable EAAT2 Modulator with Drug-like Properties and Potent Antiseizure Activity In Vivo
Source: J Med Chem. 2022 Aug 19;65(17):11703–25. doi: 10.1021/acs.jmedchem.2c00534 (PMC9469208; doi:10.1021/acs.jmedchem.2c00534)
Supplement: Supplementary file 1 — jm2c00534_si_001.pdf [file jm2c00534_si_001.pdf]

Supporting Information for

Discovery of (*R*)-*N*-benzyl-2-(2,5-dioxopyrrolidin-1-yl)propanamide [(*R*)-AS-1], a novel orally bioavailable EAAT2 modulator with drug-like properties and potent antiseizure activity *in vivo*

Michał Abram[†], Marcin Jakubiec[†], Katelyn Reeb[‡], Mary Hongying Cheng[⁑], Robin Gedschold[▲], Anna Rapacz[#], Szczepan Mogilski[#], Katarzyna Socała[⊥], Dorota Nieoczym[⊥], Małgorzata Szafarz[◊], Gniewomir Latacz[♦], Bartłomiej Szulczyk[±], Justyna Kalinowska-Tłuścik[§], Kinga Gawel[◻], Camila V. Esguerra[⁂], Elżbieta Wyska[◊], Christa E. Müller[▲], Ivet Bahar[⁑], Andréia C. K. Fontana[‡], Piotr Wlaź[⊥], Rafał M. Kamiński[†], and Krzysztof Kamiński[†]

[†] Department of Medicinal Chemistry, Faculty of Pharmacy, Jagiellonian University Medical College, Medyczna 9, 30-688 Krakow, Poland

[‡] Department of Pharmacology and Physiology, Drexel University College of Medicine, Philadelphia, PA, 19102, United States

[⁑] Department of Computational and Systems Biology, School of Medicine, University of Pittsburgh, Pittsburgh, PA 15213, United States

[#] Department of Pharmacodynamics, Faculty of Pharmacy, Jagiellonian University Medical College, Medyczna 9, 30-688 Krakow, Poland

[▲] PharmaCenter Bonn, Pharmaceutical Institute, Pharmaceutical & Medicinal Chemistry, Rheinische Friedrich-Wilhelms-Universität Bonn, An der Immenburg 4, D-53121 Bonn, Germany

[⊥] Department of Animal Physiology and Pharmacology, Institute of Biological Sciences, Faculty of Biology and Biotechnology, Maria Curie-Skłodowska University, Akademicka 19, 20-033 Lublin, Poland

[◊] Department of Pharmacokinetics and Physical Pharmacy, Faculty of Pharmacy, Jagiellonian University Medical College, Medyczna 9, 30-688 Krakow, Poland

[♦] Department of Technology and Biotechnology of Drugs, Faculty of Pharmacy, Jagiellonian University Medical College, Medyczna 9, 30-688 Krakow, Poland

[±] Department of Pharmacodynamics, Centre for Preclinical Research and Technology, Medical University of Warsaw, Banacha 1B, 02-097 Warsaw, Poland

[§] Department of Crystal Chemistry and Crystal Physics, Faculty of Chemistry, Jagiellonian University, Gronostajowa 2, 30-387 Krakow, Poland

[◻] Department of Experimental and Clinical Pharmacology, Medical University of Lublin, Jaczewskiego 8B, 20-090 Lublin, Poland

[⁂] Chemical Neuroscience Group, Centre for Molecular Medicine Norway, University of Oslo, Gaustadalléen 21, Forskningsparken, 0349 Oslo, Norway

Corresponding Author

*K.K.: Phone, +48 12 620 54 59; fax, +48 12 620 54 58; e-mail, k.kaminski@uj.edu.pl.

Table of contents

X-ray analysis 3
Crystal structure determination 3
Crystal structure analysis 3
hCa_v3.2 QPatch assay 4
$GlyR_{A1}$ Ion Flux protocol 5
Recordings of tonic NMDA currents in slices of the medial prefrontal cortex 5
Table S1. Crystal data and structure refinement results. 6
Table S2. Influence of **(*R*)-7** [**(*R*)-AS-1**] and (***R***)-**8** on spontaneous locomotor activity of mice 7
Table S3. Results of hCa_v3.2 QPatch assay 7
Table S4. Radioligand binding assays for **(*R*)-7** [**(*R*)-AS-1**] 8
Table S5. Functional and transporter assays for **(*R*)-7** [**(*R*)-AS-1**]. 10
Table S6. Results of GlyRA1 agonist ion flux assay. 11
Figure S1. Influence of the test compounds **(*R*)-7** [**(*R*)-AS-1**] and **(*R*)-8** on latency time to first clonus in the *sc*PTZ test (mice, *i.p.*) 12
Figure S2. Influence of **(*R*)-7** [**(*R*)-AS-1**] on latency time to first clonus in the *sc*PTZ test (mice, *p.o.*) 12
Figure S3. Effect of repeated pretreatment with **(*R*)-7** [**(*R*)-AS-1**] on anxiety, depressive-like behavior, and spontaneous locomotor activity in mice subjected to the PTZ kindling 13
Figure S4. Influence of **(*R*)-7** [**(*R*)-AS-1**] on the seizure-like behavior in the PTZ-induced zebrafish hyperlocomotion assay. 14
Figure S5. Results of SV2A radioligand binding assay 15
Figure S6. Patch-clamp recordings of the tonic NMDA currents 16
Figure S7. Comparison of top binding poses and sites of **(*R*)-7** [**(*R*)-AS-1**] vs. **(*S*)-7** and **(*R*)-8** vs. **(*S*)-8** bind to the human EAAT2 in the OF state. 17
Figure S8. Influence of **(*R*)-8** on glutamate uptake in transfected COS-7 cells and cultured glia cells 18
Figure S9. Effects of compounds **(*R*)-7** [**(*R*)-AS-1**], **(*S*)-7**, **(*R*)-8**, and **(*S*)-8** on EAAT2 mediated glutamate uptake in transfected COS-7 cells 18
Figure S10. The UPLC chromatograms of **(*R*)-7** [**(*R*)-AS-1**] obtained after 120 min reaction with HLMs (**A**) and with MLMs (**B**) 19
Figure S11. The UPLC chromatogram of **(*R*)-7** [**(*R*)-AS-1**] glucuronidation by human liver microsomes 20
Figure S12. The influence of **(*R*)-7** [**(*R*)-AS-1**] on CYPs activity 21
Figure S13. The viability of hepatoma HepG2 cell line after incubation with **(*R*)-7** [**(*R*)-AS-1**] for 72 h 22
Figure S14. The viability of human embryonic kidney HEK-293 cell line after incubation with **(*R*)-7** [**(*R*)-AS-1**] .. 22
Figure S15. The viability of neuroblastoma SH-SY5Y cell line after incubation with **(*R*)-7** [**(*R*)-AS-1**] 23
References 24
UPLC/MS traces for intermediate and final compounds 27
HMRS traces for final compounds 43
^{1}H NMR, ^{13}C NMR spectra for intermediates and final compounds 51
Chiral HPLC chromatograms 83

X-ray analysis

Crystal structure determination

Compounds **(*R*)-7** [**(*R*)-AS-1**], **(*S*)-7** and their fluorinated derivatives **(*R*)-8** and **(*S*)-8** were crystallized by the slow-evaporation method from solution at ambient conditions. X-ray diffraction data for single, plate-shaped crystals were collected using XtalLAB Synergy-S four circle diffractometer with a mirror monochromator and a microfocus CuKα radiation source ($\lambda = 1.5418$ Å) to ensure observation of anomalous diffraction effect for absolute structure determination. Additionally, the diffractometer was equipped with the CryoStream cryostat system allowing low-temperature experiments, performed at 100(2) K. The obtained data sets were processed with CrysAlisPro software.[1] The phase problem was solved with direct methods using SIR2014.[2] Method parameters for the obtained models were refined by full-matrix least-squares on F^2 using SHELXL-2014/6.[3] Calculations were performed using WinGX integrated system (ver. 2014.1).[4] Figures were prepared with Mercury 4.0 software.[5]

All non-hydrogen atoms were refined anisotropically. All hydrogen atoms attached to carbon atoms were positioned with the idealized geometry and refined using the riding model with the isotropic displacement parameter $U_{iso}[H] = 1.2\ U_{eq}[C]$ for all but the methyl group, for which $U_{iso}[H] = 1.5\ U_{eq}[C]$ was applied. Hydrogen atoms bound to nitrogen atoms of the amide moiety were located on the Fourier difference map and refined with no restraints. The absolute configuration for the analyzed compounds has been confirmed by the anomalous dispersion phenomenon. Crystal data and refinement results are shown in **Table S1**.

Crystallographic data have been deposited with the Cambridge Crystallographic Data Centre as supplementary publication nos.: CCDC 2120703 (**(*R*)-7** [**(*R*)-AS-1**]), CCDC 2120704 (**(*S*)-7**), CCDC 2120705 (**(*R*)-8**) and CCDC 2120706 (**(*S*)-8**). Copies of the data can be obtained, free of charge, on application to CCDC, 12 Union Road, Cambridge CB2 1EZ, UK, (e-mail: deposit@ccdc.cam.ac.uk).

Crystal structure analysis

The separated enantiomers **(*R*)-7** [**(*R*)-AS-1**] and **(*S*)-7** and their fluorinated derivatives **(*R*)-8** and **(*S*)-8** crystallize in the monoclinic (non-centrosymmetric space group $P2_1$) and orthorhombic (non-centrosymmetric space group $P2_12_12_1$) crystal systems, respectively. Each pair of enantiomers is isostructural with very close unit cell dimensions.

The asymmetric unit of **(*R*)-7** [**(*R*)-AS-1**] and **(*S*)-7** consist of three molecules of the investigated compound. The chiral centers are located at C6, C26 and C46 in each molecule, respectively. On the contrary, in the orthorhombic structures of fluorinated derivatives **(*R*)-8** and **(*S*)-8** there are two molecules in the asymmetric unit (molecule 1 with the atom numbering scheme and the asymmetric units for all studied crystal structures are shown in **Figure 2** in MS). Surprisingly, the three molecules in the asymmetric unit of enantiomers' pair **(*R*)-7** [**(*R*)-AS-1**] and **(*S*)-7** adopt very similar conformation (RMSD calculated for atoms superposition for molecules 1 & 2, 2 & 3 and 1 & 3 are respectively: 0.3161 Å, 0.1112 Å and 0.2341 Å for structure **(*R*)-7** [**(*R*)-AS-1**] and 0.3175 Å, 0.1111 Å and 0.2364 Å for structure **(*S*)-7**). The main differentiation region is the orientation of the phenyl ring, suggesting possible free rotation of this aromatic fragment. This small difference in the molecular geometry is also evident while comparing the corresponding torsion angles N9-C10 C11-C12 (molecule 1), N29-C30 C31-C32 (molecule 2) and N49-C50 C51-C52 (molecule 1) with respective values -52.16°, -42.45°, -36.91° for structure **(*R*)-7** [**(*R*)-AS-1**] and 52.59°, 42.77°,36.93° for structure **(*S*)-7**.

On the contrary, the two molecules in the asymmetric unit of fluorinated derivatives **(*R*)-8** and **(*S*)-8** adopt more distinct conformations. The strongest conformational differentiation is observed in

the *o*-F-phenyl ring orientation and also within succinimide moiety positioning (RMSD calculated for atoms superposition for molecules 1 & 2, is 1.3973 Å and 1.3970 Å for structures **(*R*)-8** and **(*S*)-8**, respectively). In structures of both fluorinated enantiomers, a positional disorder is observed for one of the molecules of the asymmetric unit. It is defined as an alternative, rotational spatial positioning of F substituent, with site occupancies similar for both structures, equal 55% and 45% for F12A and F12, respectively (**Figure 2** in MS). Interestingly, the second molecule of the asymmetric unit does not exhibit disorder. This may be caused by a conformational stabilization related to the intramolecular hydrogen bond C36-H...O22, formed between the aromatic ring as a donor and the carbonyl oxygen of succinimide fragment as an acceptor (**Figure 2** in MS, structure **(*R*)-8** as an example). The mentioned weak intramolecular interaction can stabilize a preferential mutual arrangement of the two ring moieties, with a putative impact on the bioactivity of this compound. This contact can be initiated by increased acidity of C36-H related to the location of an electronegative F substituent in the aromatic ring. Additionally, the stabilized molecule 2 adopts the most distinct geometry, expressed by torsion angles N29-C30-C31-C36 (11.82° and -11.99° for structures **(*R*)-8** and **(*S*)-8**, respectively) and C28-C26-N21-C22 (-64.48° and 64.49° for structures **(*R*)-8** and **(*S*)-8**, respectively). The corresponding angles N9-C10-C11-C16 and C8-C6-N1-C2 for molecule 1 are -56.79°, -82.97° and 56.67°, 83.30° for **(*R*)-8** and **(*S*)-8**.

The studied crystal structures **(*R*)-7** [**(*R*)-AS-1**]/**(*S*)-7** and **(*R*)-8**/**(*S*)-8** are stabilized mainly by N-H...O hydrogen bond system, formed between peptide fragments of neighboring molecules (**Figure 2** in MS). This interaction creates chain motifs, propagating in [001] direction. Additionally, several weak intermolecular interactions are observed.

The congruous crystal packing and interactions scheme observed for both enantiomer pairs suggest that the observed difference in biological activity for enantiomer *R* and *S* is based on the chirality phenomenon, whereas comparing the activity observed for compound **(*R*)-7** [**(*R*)-AS-1**] and its fluorinated derivative **(*R*)-8**, the observed difference can be a result of conformational preference in the molecule of compound **(*R*)-8**.

hCa$_v$3.2 QPatch assay

Electrophysiological assays were conducted to profile **(*R*)-7** [**(*R*)-AS-1**] for activities on the ion channel hCav3.2 using the QPatch electrophysiological platform.
Assays were performed under described conditions. Cells were held at -100 mV for 100 ms, stepped to -110 mV for 50 ms, and stepped back to -120 mV for 100 ms. In order to activate the calcium channels, the cells were pulsed to -30 mV for 50 ms. The voltage protocol was repeated every 10s. Each concentration of compound was applied for 5 minutes.

The parameters measured were the maximum inward current evoked on stepping to -30 mV from the -100 mV holding potential. All data were filtered for seal quality, seal drop, and current amplitude. The peak current amplitude was calculated before and after compound addition and the amount of activation was assessed by dividing the Test compound current amplitude by the Control current amplitude. Control is the mean hCav3.2 current amplitude collected at the end of the control period; Test Compound is the mean hCav3.2 current amplitude collected in the presence of test compound at each concentration. Results of test compounds are listed in the following **Table S3**.

GlyR$_{A1}$ Ion Flux protocol

Electrophysiological assays were conducted to profile the **(*R*)-7** [**(*R*)-AS-1**] for agonist activities on the ion channel GlyRA 1 using the IonFlex HT electrophysiological platform. Studies were performed commercially in Eurofins Panlabs Inc, (MO, USA) under described conditions. All recordings were obtained from a holding potential of -60 mV.The compound addition sequence that was used for all additions was the same for all assays. One addition of the EC_{100} concentration of Glycine was added to establish baseline response. Each test concentration of compound was applied for 2 seconds followed by 60 seconds wash. The process was repeated with the next ascending concentration of test compound up to a maximum of six concentrations per experimental pattern.

Peak inward currents in response to the additions of compound were measured. All compound data have been normalized to the baseline peak current induced by addition of EC100 Glycine for 2 seconds:

$$Normalized\ Peak\ Current = (\ I^{Compound}/I^{\ Glycine})$$

Where I (*Compound*) is the peak current induced by addition of test compound, I Glycine is the baseline peak current induced by addition of EC_{100} Glycine. Results of test compounds are listed in the following **Table S6**.

Recordings of tonic NMDA currents in slices of the medial prefrontal cortex

The experimental procedures used in this study adhered to the institutional and international guidelines on the ethical use of animals. Three-week-old rats were anaesthetized and decapitated. Slices (300 µM) were cut (Vibrotome Leica VT 1200S) from the medial prefrontal cortex in an ice-cold cutting solution. After being prepared, the slices were incubated in standard artificial cerebrospinal fluid (ACSF). This solution was heated to 32°C for the first 20 minutes of incubation. After that the slices were incubated in the same solution at room temperature for several hours. The compositions of cutting solution and standard ACSF are presented in our previous study.[40]

Recordings were made from layer V pyramidal neurons of the medial prefrontal cortex. We used intracellular solution of the following composition (in mM): potassium-gluconate (105), KCl (20), HEPES-Na+ (10), EGTA (0,1), MgATP (4), Na_2GTP (0,5), (pH=7,4). After gigaseal formation the patch membrane was ruptured. Patch-pipettes had resistances between 4 and 5 MΩ.

Tonic NMDA currents were recorded in voltage-clamp configuration in resting current recording mode in zero magnesium extracellular solution of the following composition (in mM): NaCl (130), KCl (2.5), glucose (10), $NaHCO_3$ (25), NaH_2PO_4 (1.25) and $CaCl_2$ (2), (pH=7.4, bubbled with carbogen). Magnesium ions were omitted to facilitate NMDA receptors. Additionally, the following compounds were added to the recording solution: glycine 50 µM (to facilitate NMDA receptors), TTX 0.25 µM (to block action potential dependent synaptic transmission), DNQX 10 µM and picrotoxin 50 µM (to block AMPA and GABA receptors, respectively).

Stable tonic NMDA currents were evoked by application of NMDA 2 µM for eight or ten minutes. After that NMDA 2 µM and **(*R*)-7** [**(*R*)-AS-1**] 100 µM were coapplied for ten minutes in the same recording.

Selective NMDA receptor inhibitor AP-5 100 µM was coapplied with NMDA 2 µM after three minutes application of NMDA 2 µM. All compounds were applied to the whole bath. The results obtained are shown in **Figure S6**.

Table S1. Crystal data and structure refinement results.

	**(*S*)-7**	**(*R*)-7** [**(*R*)-AS-1**]	**(*S*)-8**	**(*R*)-8**
**Crystal data**				
Chemical formula	$C_{14}H_{16}N_2O_3$	$C_{14}H_{16}N_2O_3$	$C_{14}H_{15}FN_2O_3$	$C_{14}H_{15}FN_2O_3$
M_r	260.29	260.29	278.28	278.28
Crystal system, space group	Monoclinic, $P\ 2_1$	Monoclinic, $P\ 2_1$	Orthorhombic, $P\ 2_12_12_1$	Orthorhombic, $P\ 2_12_12_1$
Temperature (K)	100(2)	100(2)	100(2)	100(2)
a, *b*, *c* (Å)	9.2919 (1), 16.4906 (2), 13.1075 (2)	9.2963 (1), 16.4931 (1), 13.0997 (1)	9.6420 (1), 15.9663 (1), 17.6798 (1)	9.6441 (1), 15.9695 (1), 17.6802 (1)
α, β, γ (°)	90, 96.690 (1), 90	90, 96.611 (1), 90	90, 90, 90	90, 90, 90
V (Å^3)	1994.77 (4)	1994.77 (4)	2721.75 (4)	2722.95 (4)
Z	6 (Z'=3)	6 (Z'=3)	8	8
Radiation type	Cu *K*α	Cu *K*α	Cu *K*α	Cu *K*α
μ (mm^{-1})	0.76	0.76	0.889	0.889
Crystal size (mm)	0.4 × 0.1 × 0.05	0.1 × 0.1 × 0.03	0.30 × 0.10 × 0.05	0.25 × 0.03 × 0.02
**Data collection**				
Diffractometer	XtaLAB Synergy, Dualflex, HyPix			
Absorption correction	Multi-scan *CrysAlis PRO* 1.171.40.37a (Rigaku Oxford Diffraction, 2019) Empirical absorption correction using spherical harmonics, implemented in SCALE3 ABSPACK scaling algorithm.			
T_{min}, T_{max}	0.651, 1	0.884, 1	0.540, 1	0.860, 1
No. of measured, independent and observed [$I > 2\sigma(I)$] reflections	52023, 8189, 7727	60449, 8025, 7729	82943, 5576, 5448	34659, 5883, 5671
R_{int}	0.0955	0.0505	0.0503	0.0450
Theta range for data collection	3.395 to 76.529°	3.396 to 76.683°	3.730 to 75.298°	3.730 to 80.514°
**Refinement results**				
$R[F^2 > 2\sigma(F^2)]$, $wR(F^2)$, S	0.0382, 0.1031, 1.084	0.0288, 0.0697, 1.066	0.0267, 0.0690, 1.059	0.0294, 0.0727, 1.026
No. of reflections	8189	8025	5576	5883
No. of parameters	529	529	381	381
No. of restraints	1	1	0	0
H-atom treatment	Mixed: independent and constrained	Mixed: independent and constrained	Mixed: independent and constrained	Mixed: independent and constrained
$\Delta\rho_{max}$, $\Delta\rho_{min}$ (e Å^{-3})	0.144, −0.225	0.121, −0.171	0.114, −0.185	0.130, −0.162
Absolute structure	Flack x determined using 3427 quotients [(I+)-(I-)]/[(I+)+(I-)] [6]	Flack x determined using 3467 quotients [(I+)-(I-)]/[(I+)+(I-) [6]	Flack x determined using 2342 quotients [(I+)-(I-)]/[(I+)+(I-)] [6]	Flack x determined using 2376 quotients [(I+)-(I-)]/[(I+)+(I-)] [6]
Absolute structure parameter	-0.05 (10)<br>Flack x determined by classical fit to all intensities<br>−0.06 (17)	-0.13 (6)<br>Flack x determined by classical fit to all intensities<br>−0.13 (13)	−0.05 (3)<br>Flack x determined by classical fit to all intensities<br>−0.04 (12)	−0.11 (5)<br>Flack x determined by classical fit to all intensities<br>−0.07 (12)

Table S2. Influence of **(*R*)-7** [**(*R*)-AS-1**] and (***R***)**-8** on spontaneous locomotor activity of mice.

	Dose [mg]	Number of light beam crossings± SEM[a]	[%] inhibition/activation vs vehicle- treated group
Vehicle - treated group[b]	-	1.52×10^3	100
**(*R*)-7** [**(*R*)-AS-1**]	15	1.35×10^3	88.4
	30	1.96×10^3	128.8
	60	2.05×10^3 *	134.1*
	90	2.15×10^3 **	140.5**
Vehicle - treated group[b]	-	1.47×10^3	100
**(*R*)-8**	30	1.50×10^3	102.3
	60	1.74×10^3	118.6
	90	2.08×10^3 *	141.6*

[a] Results are shown as number of light beam crossings during 30 min of observation beginning at 30 min after *i.p.* administration. [b] The vehicle-treated group received 1% water solution of Tween 80. Each experimental group consisted of 8–10 animals. The statistical significance was evaluated using one-way ANOVA with Dunnett's *post hoc* test *p < 0.05, **p < 0.01.

Table S3. Results of $hCa_v3.2$ QPatch assay.

Compound	Concentration (µM)	$hCa_v3.2$ % Inhibition[a]		
		n1	n2	Mean[a]
Vehicle	0.3% DMSO	-6.43	-8.72	-7.57
**(*R*)-7** [**(*R*)-AS-1**]	100	5.25	-6.84	-0.79
Mibefradil[b]	30	96.13	94.78	95.46

[a] Results showing potentiation higher than 50% are considered to represent significant effects of test compound. Studies were performed commercially in Eurofins Panlabs Inc, (MO, USA) using testing procedures described elsewhere.[6] [b] Positive control

Table S4. Radioligand binding assays for ***(R)*-7** [**(*R*)-AS-1**].

Specific binding					
**Assay name**	**Source**	**Concentration (μM)**	**Measurement**	**Mean[a,*]**	**Ref.**
Alpha 2 (non-selective) (Antagonist radioligand)	Rat brain	100	% Inhibition	14.69	8
Ca^{2+} channel (L, dihydropyridine site) (Antagonist radioligand)	Rat brain	100	% Inhibition	1.54	9
Ca^{2+} channel (L, diltiazem site) (Antagonist radioligand)	Rat brain	100	% Inhibition	9.50	10
Ca^{2+} channel (L, verapamil site) (Antagonist radioligand)	Rat brain	100	% Inhibition	-1.01	11
GABAA Ion Channel (non-selective) [^{3}H] Muscimol Binding (Agonist Radioligand)	Rat brain	100	% Inhibition	11.00	12
GABAA Ion Channel (non-selective) [^{3}H] Ro-15-1788 Binding (Hippocampus)	Rat hippocampus	100	% Inhibition	0	13
Glutamate (AMPA, Non-Selective) Ion Channel [^{3}H] AMPA Binding	Rat cerebral cortex	100	% Inhibition	-4.00	14
Glutamate (Kainate, Non-Selective) Ion Channel [^{3}H] Kainic acid Binding	Rat cerebral cortex	10	% Inhibition	-11.00	16, 17
Glutamate (NMDA, Non-Selective) Ion Channel [^{3}H] MK-801 Binding	Rat brain	100	% Inhibition	8.00	17
Glutamate, NMDA, Glycine [^{3}H] MDL 105,519 Binding	Rat cerebral cortex	10	% Inhibition	-3.00	18
Glycine Ion Channel (non-selective) [^{3}H] Strychnine Binding	Rat spinal cord	10	% Inhibition	-4.00	19
GLYT1 Glycine Transporter Binding (Antagonist Radioligand)	Rat brain	10	% Inhibition	-2.00	20
		100		-1.00	
mGlu2 (*h*) (Antagonist Radioligand)	Human recombinant (Chem-1 cells)	50	% Inhibition	-8.00	21
mGluR1 (Agonist radioligand)	Rat brain	10	% Inhibition	-6.72	22
mGluR5 (*h*) (Agonist radioligand)	Human recombinant	10	% Inhibition	-7.78	22
Na^+ channel (site 2) (Antagonist radioligand)	Rat brain	100	% Inhibition	0.49	23
NMDA (Antagonist radioligand)	Rat brain	100	% Inhibition	16.12	24
Potassium Channel hERG (*h*) - [^{3}H] Dofetilide	Human recombinant	100	% Inhibition	-8.02	25

Sigma (Non-Selective) Binding (Agonist Radioligand)	Guinea Pig brain	10	% Inhibition	-9.00	26

[a] Results showing activity higher than 50% are considered to represent significant effects of the test compounds; results showing an inhibition between 25% and 50% are indicative of moderate effect; results showing an inhibition lower than 25% are not considered significant and mostly attributable to variability of the signal around the control level. * Assays performed in duplicate. Binding studies were performed commercially in Eurofins Cerep SA (Celle l'Evescault, France) or in Panlabs Discovery Services Taiwan, Ltd. (New Taipei City, Taiwan).

Table S5. Functional and transporter assays for **(*R*)-7** [**(*R*)-AS-1**].

Functional assays					
**Assay name**	**Source**	**Concentration(μM)**	**Measurement**	**Mean[a]**	**Ref.**
Acetylcholinesterase (*h*) (Enzymatic activity)*	Human recombinant (CHO-K1 cells)	10	% Inhibition	1.59[#]	27
Alpha 1A (*h*) (Agonist effect)*	Human recombinant (CHO-K1 cells)	100	% Effect	-2.05[#]	28
Alpha 1A (*h*) (Antagonist effect)*	Human recombinant (CHO-K1 cells)	100	% Inhibition	1.28[#]	28
CB_1 (*h*) (Agonist effect)*	Human recombinant (CHO-K1 cells)	100	% Effect	-6.83[#]	29
CB_1 (*h*) (Antagonist effect)*	Human recombinant (CHO-K1 cells)	100	% Inhibition	5.59[#]	29
D_1 (*h*) (Agonist effect)*	Human recombinant (CHO-K1 cells)	100	% Effect	1.70[#]	30
D_1 (*h*) (Antagonist effect)*	Human recombinant (CHO-K1 cells)	100	% Inhibition	-2.57[#]	30
D_2L (*h*) (Agonist effect)*	Human recombinant (CHO-K1 cells)	100	% Effect	0.22[#]	31
D_2L (*h*) (Antagonist effect)*	Human recombinant (CHO-K1 cells)	100	% Inhibition	3.27[#]	31
sst2 (*h*) (Agonist effect)*	Human recombinant (Chem-1 cells)	10	% Effect	0.50[#]	32
sst2 (*h*) (Antagonist effect)*	Human recombinant (Chem-1 cells)	50	% Inhibition	5.25[#]	32
sst4 (*h*) (Agonist effect)*	Human recombinant (CHO-K1 cells)	10	% Effect	-7.88[#]	33
sst4 (*h*) (Antagonist effect)*	Human recombinant (CHO-K1 cells)	50	% Inhibition	8.25[#]	33
5-HT_6 (*h*) (Agonist effect)*	Human recombinant (CHO-K1 cells)	100	% Effect	-0.26[#]	34
5-HT_6 (*h*) (Antagonist effect)*	Human recombinant	100	% Inhibition	-6.42[#]	34

	(CHO-K1 cells)				
5-HT_7 (*h*) (Agonist effect)*	Human recombinant (CHO-K1 cells)	10	% Effect	-4.26[#]	35
5-HT_7 (*h*) (Antagonist effect)*	Human recombinant (CHO-K1 cells)	10	% Inhibition	-25.0[#]	35
**Transporter assays**					
Dopamine transporter (*h*) (Antagonist radioligand)*	Human recombinant	100	% Inhibition	-2.69[#]	36
Norepinephrine transporter (*h*) (Antagonist radioligand)*	Human recombinant	100	% Inhibition	3.01[#]	37
Serotonin (5-HT) transporter (*h*) (Antagonist radioligand)*	Human recombinant	100	% Inhibition	-7.24[#]	38
GABA transporter GAT-1 (Substrate radioligand)**	Transfection in COS-7 cells	4 independent assays in 24 well plates (concentration: 0.01–100 μM)	% Effect	0	39
GABA transporter GAT-3 (Substrate radioligand)**	Transfection in COS-7 cells	4 independent assays in 24 well plates (concentration: 0.01–100 μM)	% Effect	0	39

[a] Results showing activity higher than 50% are considered to represent significant effects of the test compounds; results showing an inhibition between 25% and 50% are indicative of moderate effect; results showing an inhibition lower than 25% are not considered significant and mostly attributable to variability of the signal around the control level. [#] Assays performed in duplicate.*Functional/transporter studies performed commercially in Eurofins Cerep SA (Celle l'Evescault, France) or in Panlabs Discovery Services Taiwan, Ltd. (New Taipei City, Taiwan). **Transporter studies performed in Department of Pharmacology and Physiology, Drexel University College of Medicine, Philadelphia, PA, 19102, United States.

Table S6. Results of GlyRA1 agonist ion flux assay.

**GlyRA1 Agonist IonFlux Assay**				
**Compound**	**Concentration (μM)**	**% Control[a]**		
		n1	n2	Mean[a]
Vehicle	0.3% DMSO	1.66	2.44	2.05
**(*R*)-7** [**(*R*)-AS-1**]	100	1.83	2.05	1.94
Glycine[b]	100	92.59	87.38	89.99

[a] Results showing potentiation higher than 25% are considered to represent significant effects of test compounds. Studies were performed commercially in Eurofins Panlabs Inc, (MO, USA) using testing procedures described elsewhere.[7] [b] Positive control.

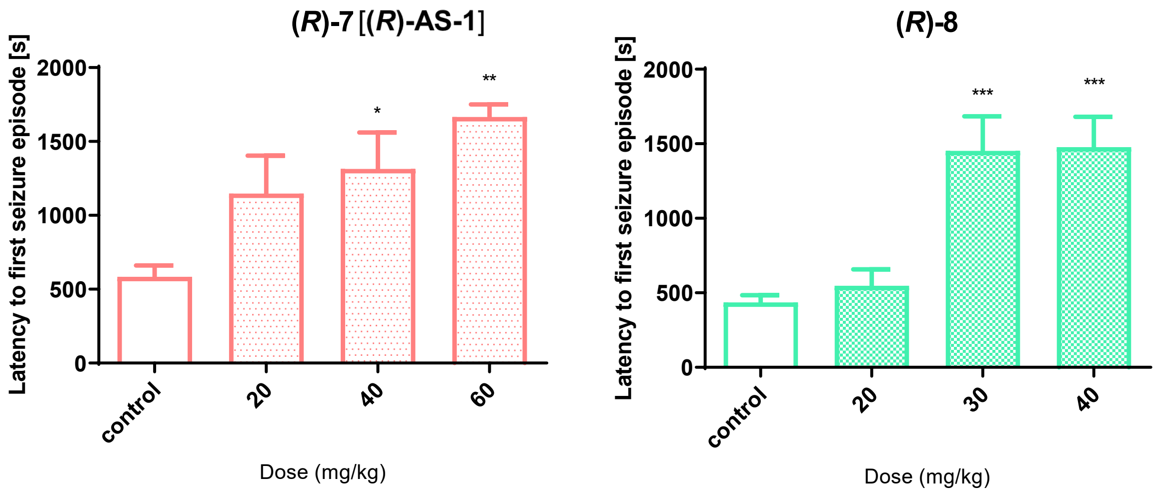

Figure S1. Influence of the test compounds **(*R*)-7** [**(*R*)-AS-1**] and **(*R*)-8** on latency time to first clonus in the *sc*PTZ test (mice, *i.p.*). Data are shown as mean ± SEM (n=6 animals). The statistical significance was evaluated by a one-way ANOVA, followed by *Dunnet's post hoc* test: *p < 0.05, **p < 0.01, ***p < 0.001 (GraphPad Prism 8.0.1).

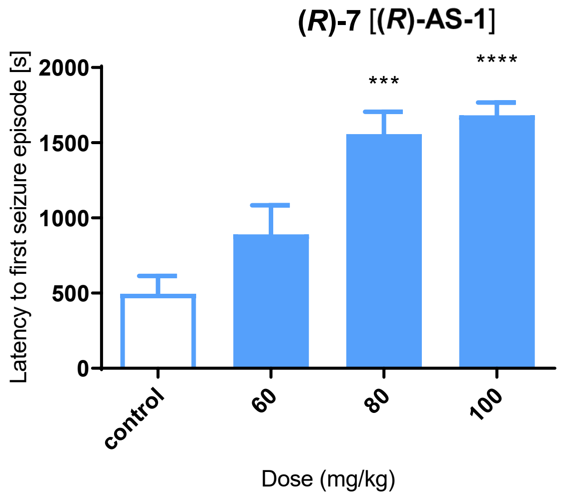

Figure S2. Influence of **(*R*)-7** [**(*R*)-AS-1**] on latency time to first clonus in the *sc*PTZ test (mice, *p.o.*). Data are shown as mean ± SEM. (n=6 animals). The statistical significance was evaluated by a one-way ANOVA, followed by *Dunnet's post hoc* test ***p < 0.001, ****p < 0.0001 (GraphPad Prism 8.0.1).

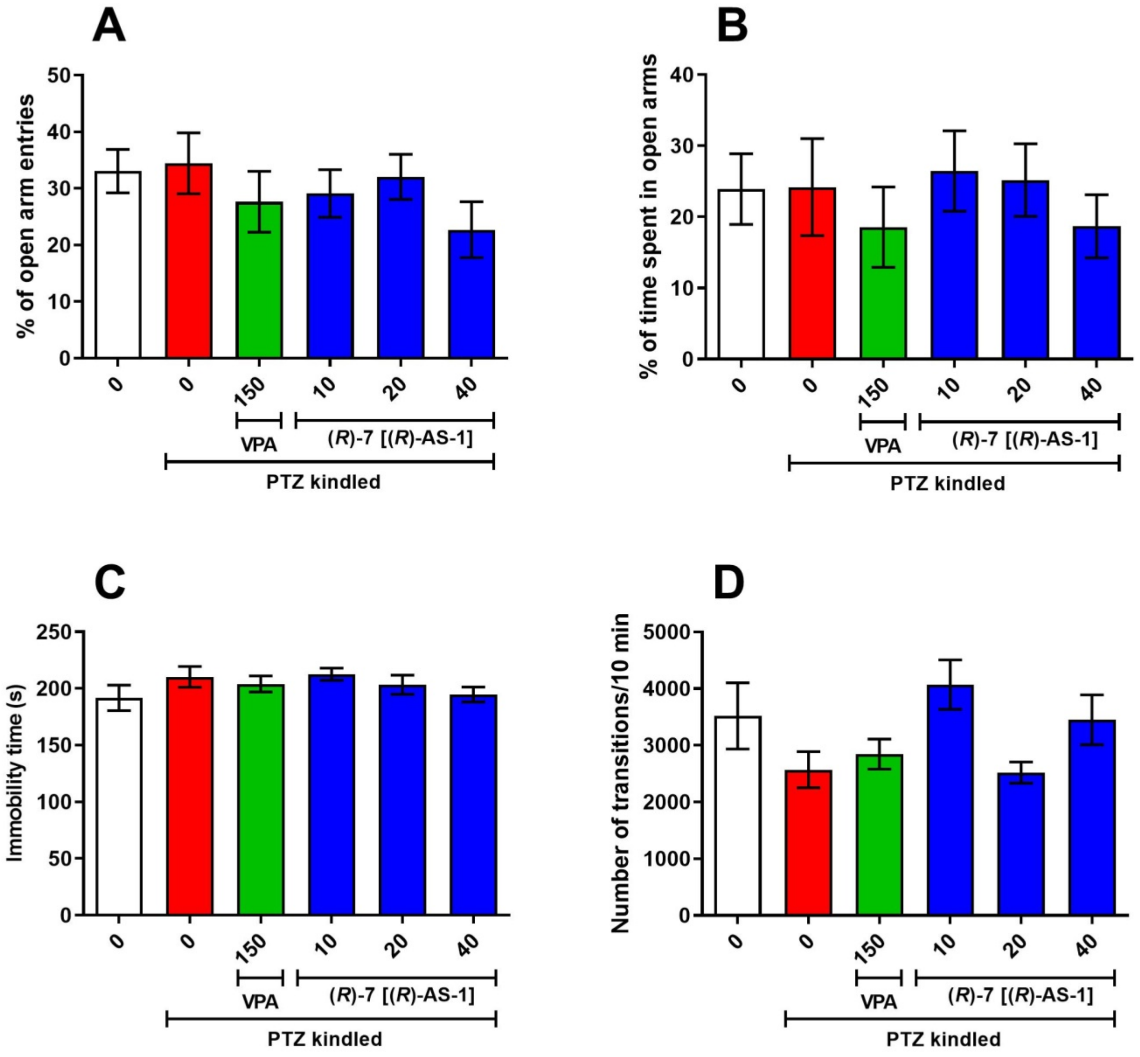

Figure S3. Effect of repeated pretreatment with **(*R*)-7** [**(*R*)-AS-1**] on anxiety, depressive-like behavior, and spontaneous locomotor activity in mice subjected to the PTZ kindling. (**A**) Influence of **(*R*)-7** [**(*R*)-AS-1**] on the percentage of the open arms entries in the elevated plus maze test. (**B**) Influence of **(*R*)-7** [**(*R*)-AS-1**] on the percentage of the time spent in the open arms in the elevated plus maze test. (**C**) Influence of **(*R*)-7** [**(*R*)-AS-1**] on the total immobility duration in the forced swim test. (**D**) Influence of **(*R*)-7** [**(*R*)-AS-1**] on locomotor activity. PTZ (40 mg/kg, *i.p.*) was injected every 48 h for a total of 21 injections. **(*R*)-7** [**(*R*)-AS-1**] and VPA were injected *i.p.*, 30 min before PTZ injection. Data are shown as mean (n=10–15 animals). The statistical significance was evaluated by one-way ANOVA (GraphPad Prism 8.0.1).

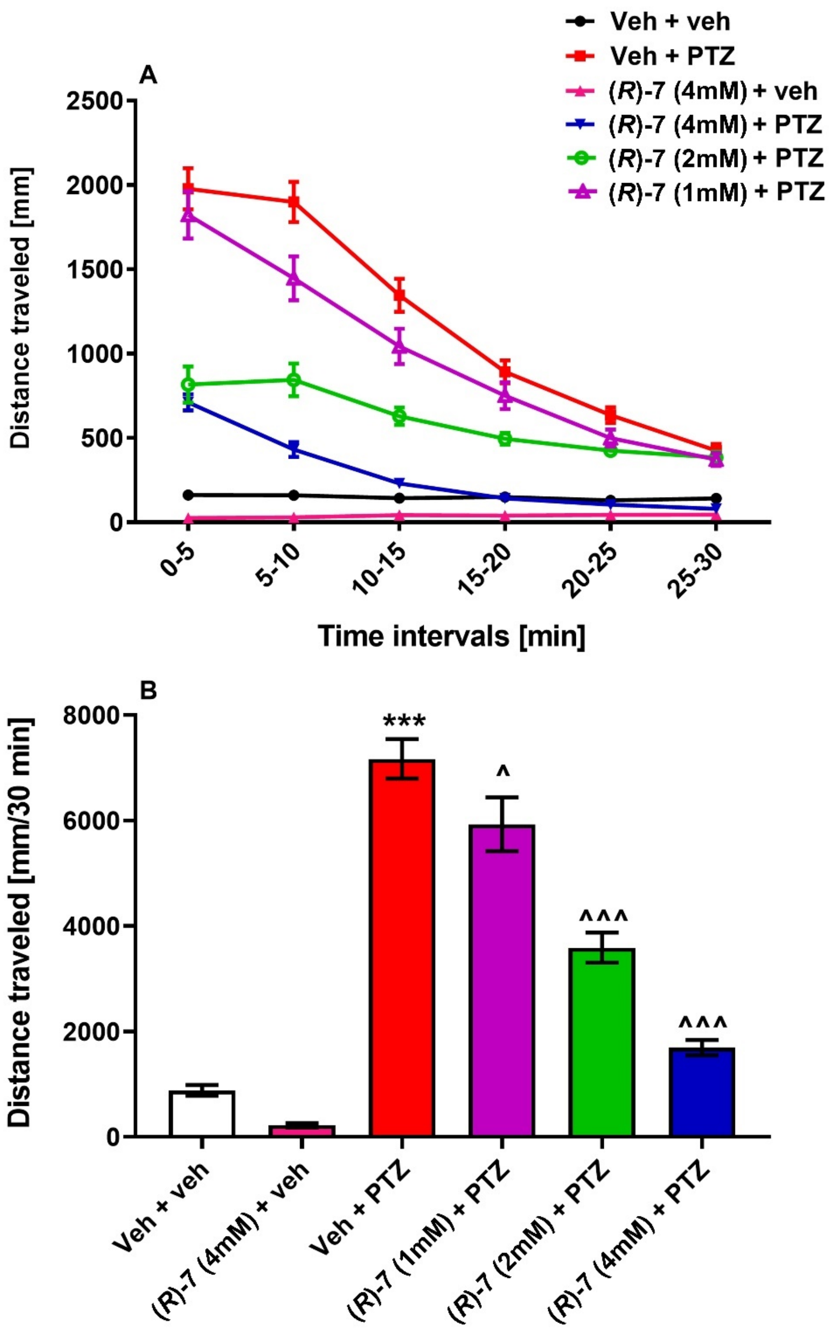

Figure S4. Influence of **(*R*)-7** [**(*R*)-AS-1**] on the seizure-like behavior in the PTZ-induced zebrafish hyperlocomotion assay. After a 24-h incubation in different doses of **(*R*)-7** [**(*R*)-AS-1**] (1, 2 or 4 mM), 7 days old zebrafish larvae were exposed to PTZ (20 mM). Larval behavior was assessed 5 min after PTZ administration. Results of the assay are depicted as: (**A**) distance covered by larvae in 5 min long time bins, and (**B**) total distance covered by larvae during 30 min of assay. Data are shown as mean ± SEM. Veh + veh (n = 48), **(*R*)-7** [**(*R*)-AS-1**] (4 mM) + Veh (n = 48), Veh + PTZ (n = 36), **(*R*)-7** [**(*R*)-AS-1**] (1 mM) + PTZ (n = 24), **(*R*)-7** [**(*R*)-AS-1**] (2 mM) + PTZ (n = 38), **(*R*)-7** [**(*R*)-AS-1**] (4 mM) + PTZ (n = 34). The statistical significance was evaluated by one-way or two-way ANOVA with repeated measures followed by the Tukey's or Bonferroni's *post hoc* test: ***$p < 0.001$ vs. Veh + veh; ^^^$p < 0.001$, ^$p < 0.05$ vs. Veh + PTZ. PTZ – pentylenetetrazol, Veh – vehicle (GraphPad Prism 8.0.1).

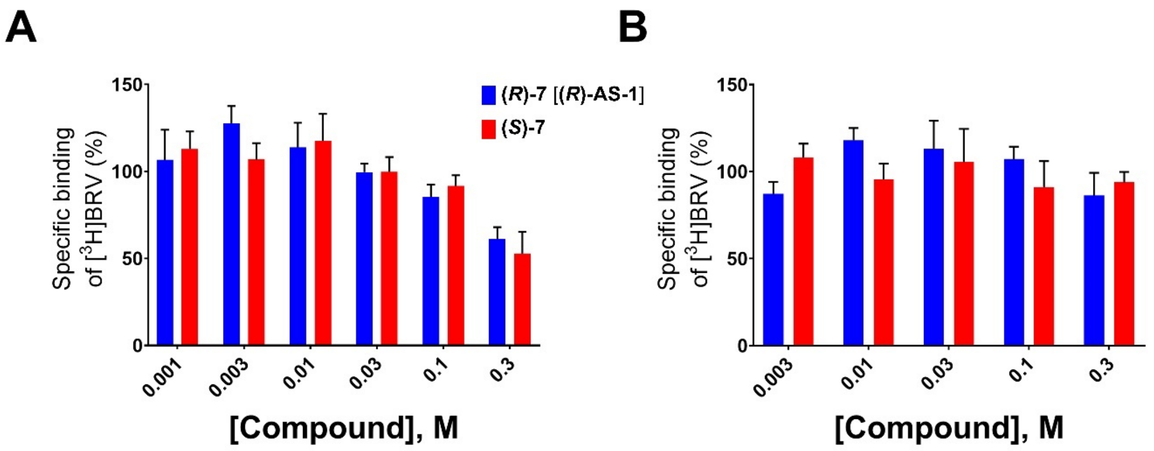

Figure S5. Results of SV2A radioligand binding assay; 3 separate experiments were performed, each in duplicates; non-specific binding was determined in the presence of 1 mM levetiracetam, final DMSO concentration was 2%. (**A**) Binding to human SV2A, **(*R*)-7** [**(*R*)-AS-1**]: K_i 275 ± 53 µM, **(*S*)-7**: K_i 255 ± 69 µM. (**B**) Binding to mouse SV2A: no binding observed. (Results were analyzed by GraphPad Prism 7.0).

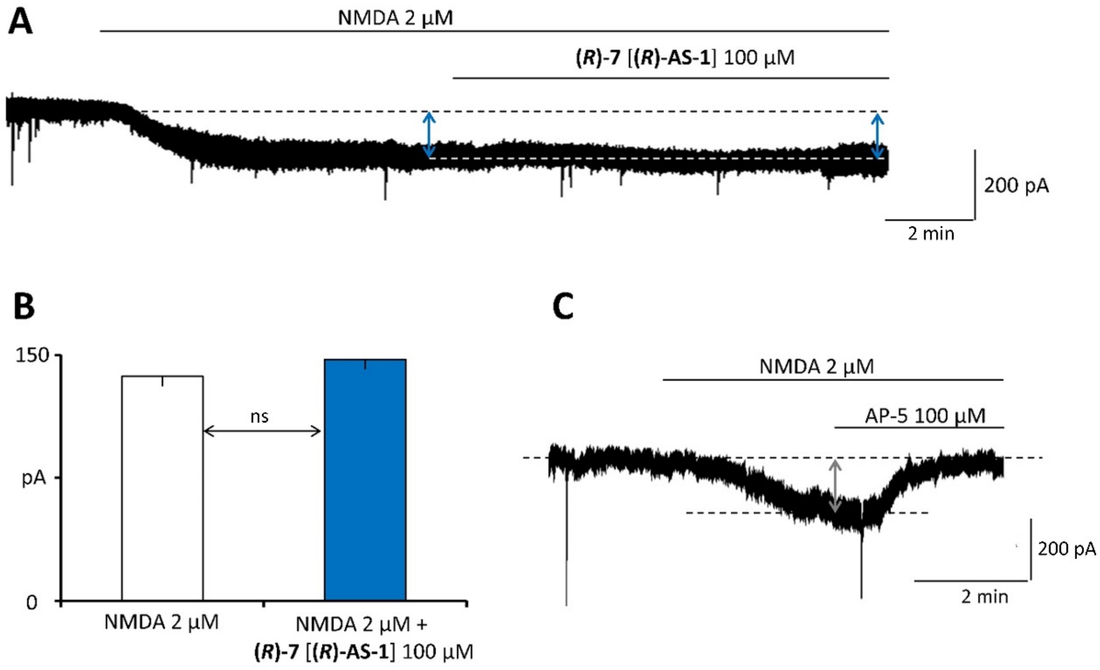

Figure S6. Patch-clamp recordings of the tonic NMDA currents. NMDA 2 µM was applied for 8 or 10 minutes to evoke stable NMDA currents. The amplitude of these control NMDA currents was 137±6.2 pA as shown by left blue arrow in panel (**A**) After evoking stable NMDA currents, NMDA 2 µM and **(*R*)-7** [**(*R*)-AS-1**] 100 µM were coapplied for ten minutes. NMDA currents after ten minutes application of **(*R*)-7** [**(*R*)-AS-1**] 100 µM was 147.3±5.9 pA (as shown by right vertical blue arrow in panel **A**), which was not significantly different from control NMDA currents (see panel **B**, n=3, Wilcoxon matched pairs test, $p > 0.05$, GraphPad Prism 8.0.1). The selective NMDA inhibitor AP-5 at concentration of 100 µM fully inhibited tonic NMDA currents as shown on an example recording in panel **C** (control NMDA current is indicated by vertical grey arrow).

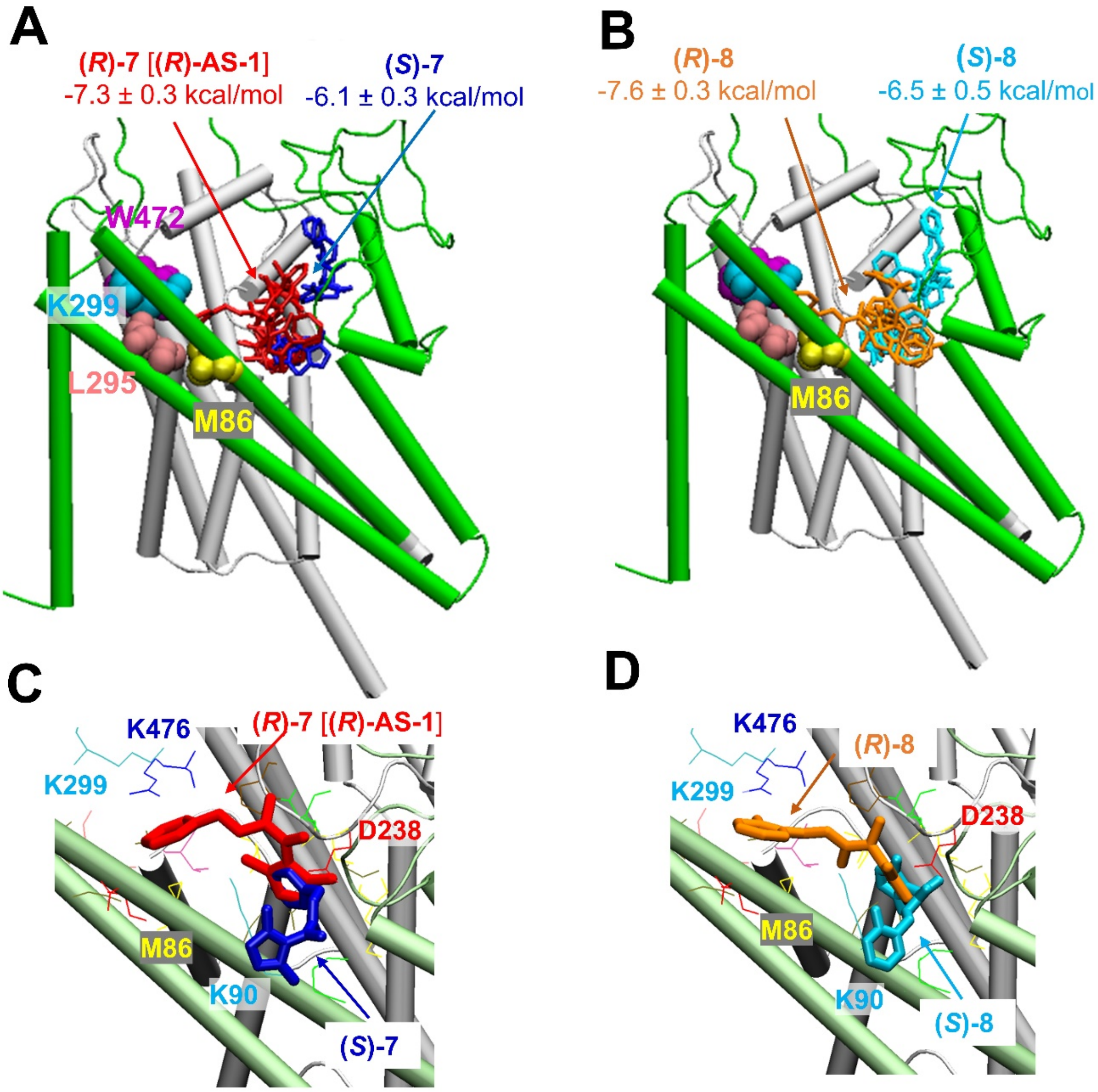

Figure S7. Comparison of top binding poses and sites of (**A**) **(*R*)-7** [**(*R*)-AS-1**] vs. **(*S*)-7** and (**B**) **(*R*)-8** vs. **(*S*)-8** bind to the human EAAT2 in the OF state. The diagrams show the multiple binding poses of the compounds onto OF EAAT2. **(*R*)-7** [**(*R*)-AS-1**] and **(*S*)-7** in panel **A** are shown in *red* and *blue sticks*, respectively. AutoDock predicts the respective binding affinities of **(*R*)-7** [**(*R*)-AS-1**] and **(*S*)-7** as -7.3±0.3 kcal/mol and -6.1±0.3 kcal/mol. **(*R*)-8** and **(*S*)-8** in panel **B** are shown in *orange* and *cyan sticks*. Their binding affinities are predicted to be -7.6±0.3 kcal/mol and -6.5±0.5 kcal/mol, respectively. Residues reported earlier to coordinate the binding of PAM (*R*)-GT949 are displayed in van der Waals (vDW) representation in different colors. Binding affinities are averaged over all poses displayed in the diagrams. Binding poses of **(*R*)-7** [**(*R*)-AS-1**] are also shown in **Figure 8A**. (**C**–**D**) Detailed comparison of the predicted binding poses of **(*R*)-7** [**(*R*)-AS-1**] versus **(*S*)-7** (**C**), and **(*R*)-8** versus **(S)-8** (**D**) onto Site 1A. Binding poses of **(*R*)-7** [**(*R*)-AS-1**] and **(*R*)-8** are also shown in **Figure 8B**–**C**. Note that residues reported earlier to coordinate the binding of PAM (*R*)-GT949, e.g. M86 and L295, are not in direct contacts with *S*-enantiomers.

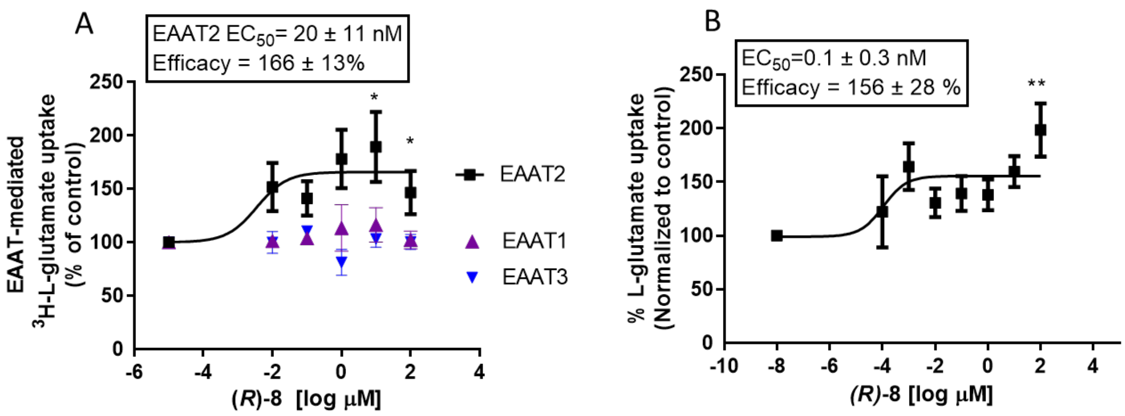

Figure S8. Influence of **(*R*)-8** on glutamate uptake in transfected COS-7 cells and cultured glia cells. (**A**) **(*R*)-8** augments glutamate uptake mediated by EAAT2 in transfected COS-7 cells (but not EAAT1 and EAAT3) (*p < 0.05 compared to vehicle). (**B**) **(*R*)-8** augments glutamate uptake in cultured glia cells (**p < 0.01 compared to vehicle). Data is average of triplicate determinations of four independent experiments ± SEM, normalized to percentage of control (vehicle). ANOVA followed by Dunnet's *post hoc* test, comparing different concentrations to vehicle (GraphPad Prism 9).

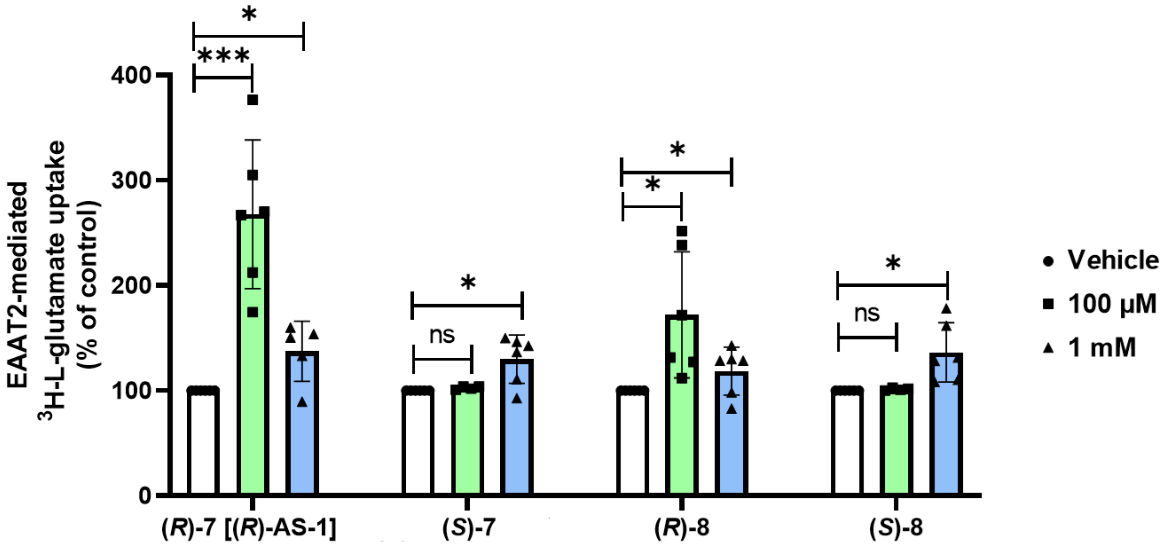

Figure S9. Effects of compounds **(*R*)-7** [**(*R*)-AS-1**], **(*S*)-7**, **(*R*)-8**, and **(*S*)-8** on EAAT2 mediated glutamate uptake in transfected COS-7 cells, at 100 µM and 1 mM concentrations (*p < 0.05 and *** p < 0.001, compared to vehicle). *R*-enantiomers affect EAAT2-mediated glutamate uptake at much higher potency than *S*-enantiomers. Data is average of triplicate determinations of three to four independent experiments ± SEM, normalized to percentage of control (vehicle). ANOVA followed by Dunnet's *post hoc* test, comparing different concentrations to vehicle (GraphPad Prism 9).

Phase I

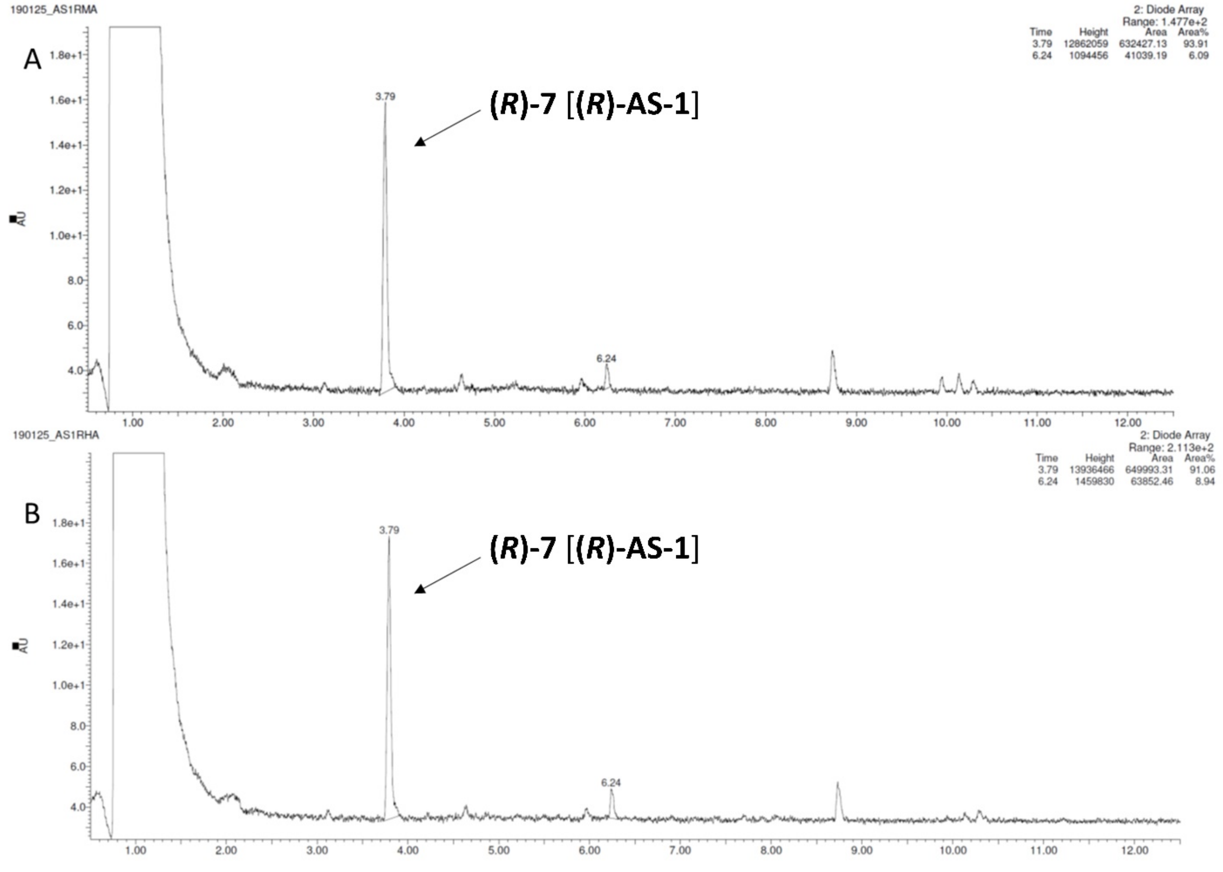

Figure S10. The UPLC chromatograms of **(*R*)-7** [**(*R*)-AS-1**] obtained after 120 min reaction with HLMs (**A**) and with MLMs (**B**). No metabolites found. Peaks at 6.24 were identified as a contamination.

Phase II (glucuronidation)

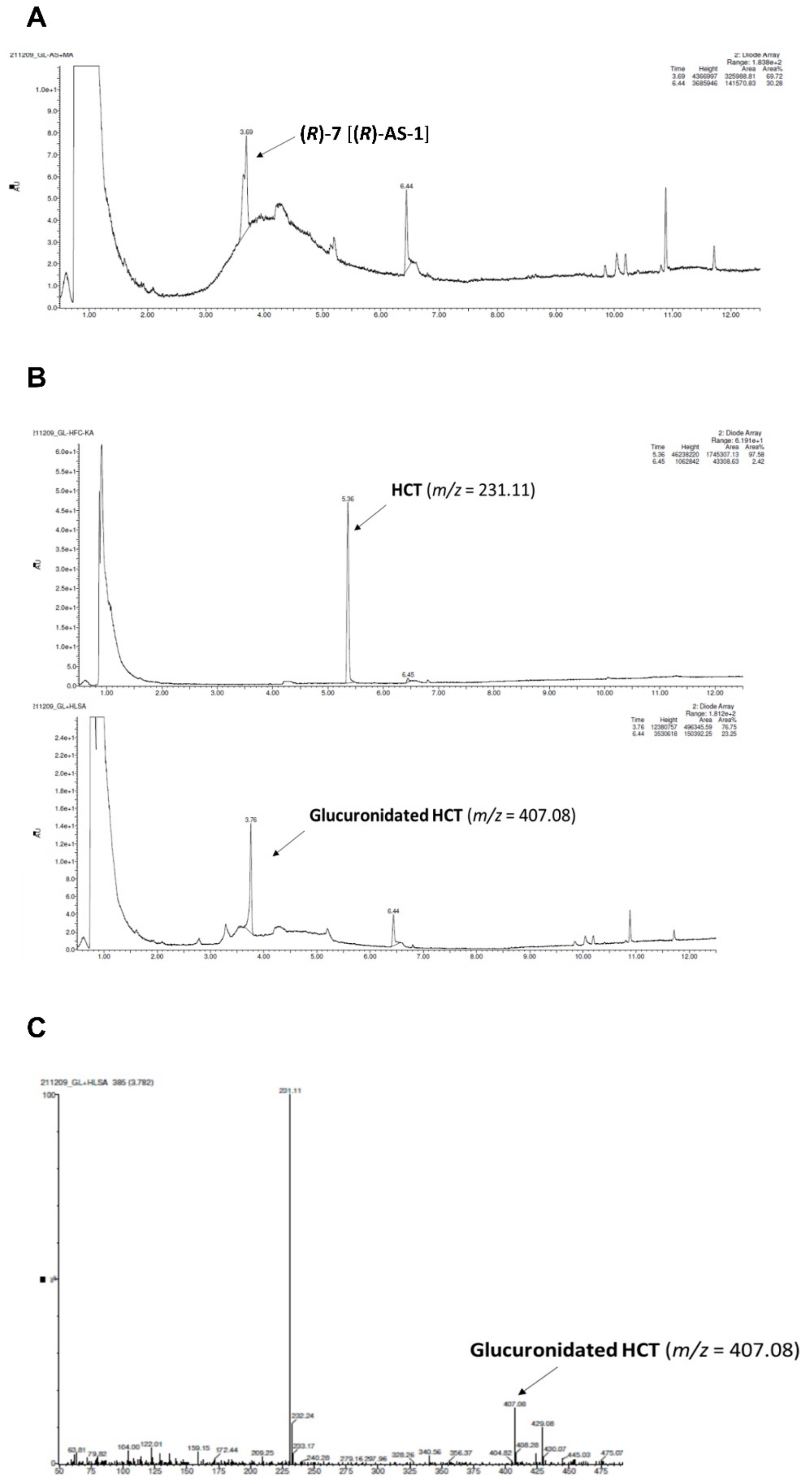

Figure S11. The UPLC chromatogram of **(*R*)-7** [**(*R*)-AS-1**] glucuronidation by human liver microsomes (**A**). No metabolites found. Peak at 6.44 was identified as a contamination (**B**) The glucuronidation of 7-hydroxy-4-trifluoromethylcoumarin (HCT) by HLMs (the reference reaction). Peaks at 6.44-6.45 were identified as a contamination. (**C**) The mass spectra of glucuronidated 7-hydroxy-4-trifluoromethylcoumarin (HCT) by HLMs.

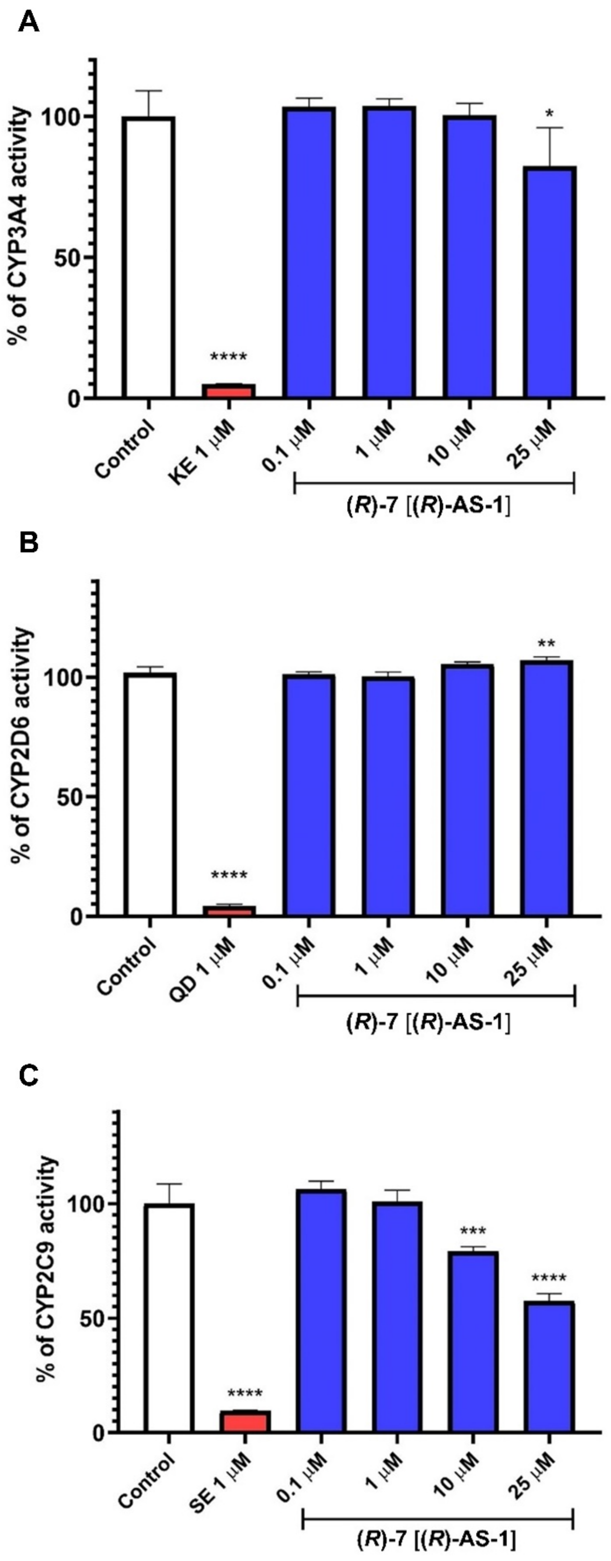

Figure S12. The influence of **(*R*)-7** [**(*R*)-AS-1**] on CYPs activity: (**A**) Influence on CYP3A4, ketoconazole (KE, 1 µM) was used as reference inhibitor. (**B**) Influence on CYP2D6 activity, quinidine (QD, 1 µM) was used as reference inhibitor. (**C**) Influence on CYP2C9 activity. Sulfaphenazole (SE, 1 µM) was used as reference inhibitor. The statistical significance was evaluated by a one-way ANOVA, followed by Bonferroni's Comparison Test (*p < 0.05, **p < 0.01, ***p < 0.001, ****p < 0.0001 compared with control activity).

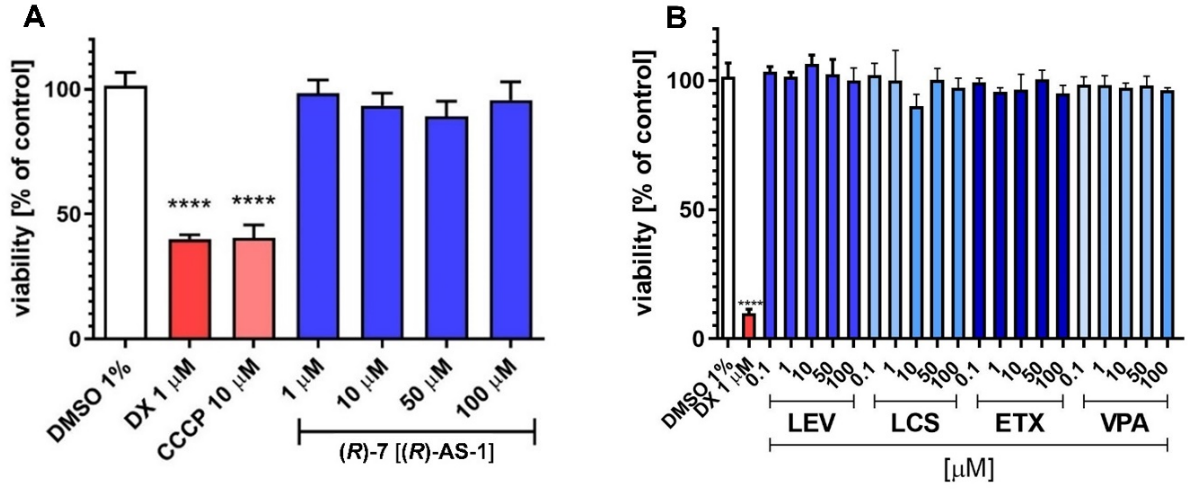

Figure S13. (**A**) The viability of hepatoma HepG2 cell line after incubation with **(*R*)-7** [**(*R*)-AS-1**] for 72 h. Two toxins: doxorubicin (DX, 1 µM) and carbonyl cyanide 3-chlorophenyl-hydrazone (CCCP,10 µM) were used as references. (**B**) The viability of hepatoma HepG2 cell line after incubation with reference AEDs: Levetiracetam (LEV), Lacosamide (LCS), ethosuximide (ETX), valproic acid (VPA) for 72 h. Doxorubicin (DX, 1 µM) was used as reference. The statistical significance was evaluated by a one-way ANOVA, followed by Bonferroni's Comparison Test (****$p < 0.0001$ compared with negative control - medium with 1% DMSO).

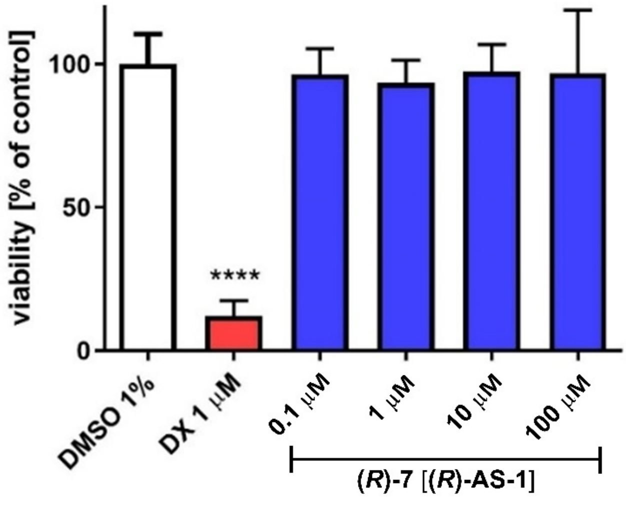

Figure S14. The viability of human embryonic kidney HEK-293 cell line after incubation with **(*R*)-7** [**(*R*)-AS-1**] for 72 h. Doxorubicin (DX, 1 µM) was used as the reference toxin. The statistical significance was evaluated by a one-way ANOVA, followed by Bonferroni's Comparison Test (****$p < 0.0001$ compared with negative control - medium with 1% DMSO).

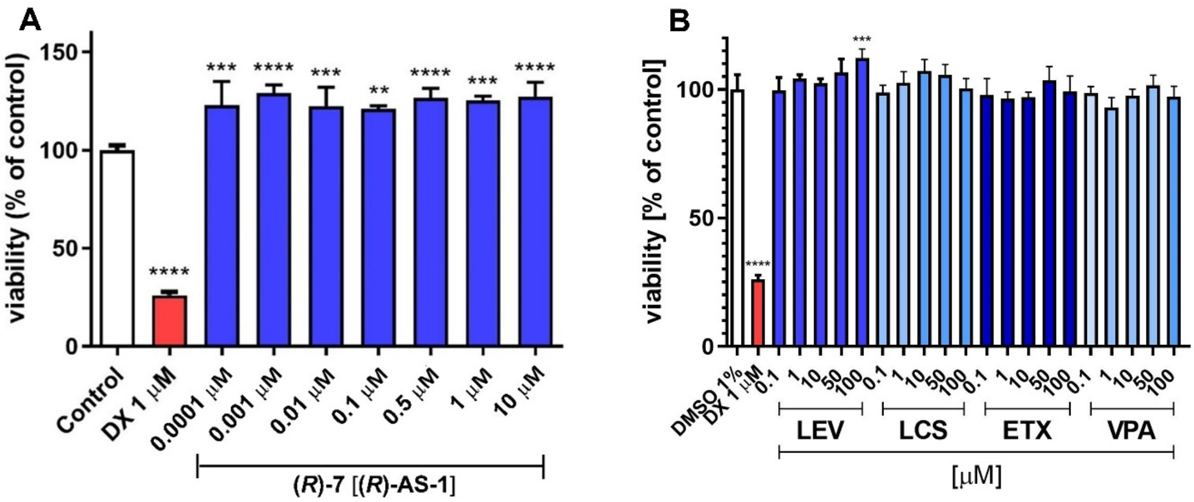

Figure S15. (**A**) The viability of neuroblastoma SH-SY5Y cell line after incubation with **(*R*)-7** [**(*R*)-AS-1**] for 72 h. Doxorubicin (DX, 1 μM) was used as reference. (**B**) The viability of neuroblastoma SH-SY5Y cell line after incubation with reference AEDs: levetiracetam (LEV), lacosamide (LCS), ethosuximide (ETX), valproic acid (VPA) for 72 h. Doxorubicin (DX, 1 μM) was used as reference. The statistical significance was evaluated by a one-way ANOVA, followed by Bonferroni's Comparison Test (**$p < 0.01$, ***$p < 0.001$, ****$p < 0.0001$ compared with negative control - medium with 1% DMSO).

References:

(1) Rigaku-Oxford Diffraction; CrysAlisPro Oxford Diffraction Ltd, Abingdon, England V 1. 171. 36. 2. (Release 27-06-2012 CN) 2006.

(2) Burla, M. C.; Caliandro, R.; Carrozzini, B.; Cascarano, G. L.; Cuocci, C.; Giacovazzo, C.; Mallamo, M.; Mazzone, A.; Polidori, G. Crystal Structure Determination and Refinement via SIR2014. *J. Appl. Crystallogr.* **2015**, *48* (1), 306–309. https://doi.org/10.1107/S1600576715001132.

(3) Sheldrick, G. M. A Short History of SHELX. *Acta Crystallogr. A* **2008**, *64* (1), 112–122. https://doi.org/10.1107/S0108767307043930.

(4) Farrugia, L. J. WinGX Suite for Small-Molecule Single-Crystal Crystallography. *J. Appl. Crystallogr.* **1999**, *32* (4), 837–838. https://doi.org/10.1107/S0021889899006020.

(5) Macrae, C. F.; Sovago, I.; Cottrell, S. J.; Galek, P. T. A.; McCabe, P.; Pidcock, E.; Platings, M.; Shields, G. P.; Stevens, J. S.; Towler, M.; Wood, P. A. Mercury 4.0: From Visualization to Analysis, Design and Prediction. *J. Appl. Crystallogr.* **2020**, *53* (1), 226–235. https://doi.org/10.1107/S1600576719014092.

(6) Eurofins Discovery https://www.eurofinsdiscoveryservices.com/catalogmanagement/viewItem/Cav3.2-%28T-type%29-Human-Calcium-Ion-Channel-Cell-Based-Antagonist-Automated-Patch-Clamp-Assay-%283-Conc.%29/CY8075QP2 (accessed 2021 -12 -15).

(7) Eurofins Discovery https://www.eurofinsdiscoveryservices.com/catalogmanagement/viewItem/GlyRA1-Human-Glycine-Ion-Channel-Cell-Based-Agonist-IonFlux-Assay/CYL8056IF (accessed 2021 -12 -15).

(8) Uhlén, S.; Wikberg, J. E. Rat Spinal Cord Alpha 2-Adrenoceptors Are of the Alpha 2A-Subtype: Comparison with Alpha 2A- and Alpha 2B-Adrenoceptors in Rat Spleen, Cerebral Cortex and Kidney Using 3H-RX821002 Ligand Binding. *Pharmacol. Toxicol.* **1991**, *69* (5), 341–350. https://doi.org/10.1111/j.1600-0773.1991.tb01308.x.

(9) Gould, R. J.; Murphy, K. M.; Snyder, S. H. [3H]Nitrendipine-Labeled Calcium Channels Discriminate Inorganic Calcium Agonists and Antagonists. *Proc. Natl. Acad. Sci.* **1982**, *79* (11), 3656–3660. https://doi.org/10.1073/pnas.79.11.3656.

(10) Schoemaker, H.; Langer, S. Z. [3H]Diltiazem Binding to Calcium Channel Antagonists Recognition Sites in Rat Cerebral Cortex. *Eur. J. Pharmacol.* **1985**, *111* (2), 273–277. https://doi.org/10.1016/0014-2999(85)90768-x.

(11) Reynolds, I. J.; Snowman, A. M.; Snyder, S. H. (-)-[3H] Desmethoxyverapamil Labels Multiple Calcium Channel Modulator Receptors in Brain and Skeletal Muscle Membranes: Differentiation by Temperature and Dihydropyridines. *J. Pharmacol. Exp. Ther.* **1986**, *237* (3), 731–738.

(12) Eurofins Discovery https://www.eurofinsdiscoveryservices.com/catalogmanagement/viewItem/Non-Selective-Rat-GABAA-Ion-Channel-3H-Muscimol-Binding-Agonist-Radioligand-Assay-Panlabs/226500 (accessed 2021 -12 -15).

(13) Eurofins Discovery https://www.eurofinsdiscoveryservices.com/catalogmanagement/viewItem/Non-Selective-Rat-GABAA-Ion-Channel-3H-Ro-15-1788-Binding-Hippocampus-Assay-Panlabs/226630 (accessed 2021 -12 -15).

(14) Eurofins Discovery https://www.eurofinsdiscoveryservices.com/catalogmanagement/viewItem/Glutamate-AMPA-Non-Selective-Rat-Ion-Channel-3H-AMPA-Binding-Assay-Panlabs/232600 (accessed 2021 -12 -15).

(15) Smith, A. L.; McIlhinney, R. A. Effects of Acromelic Acid A on the Binding of [3H]-Kainic Acid and [3H]-AMPA to Rat Brain Synaptic Plasma Membranes. *Br. J. Pharmacol.* **1992**, *105* (1), 83–86. https://doi.org/10.1111/j.1476-5381.1992.tb14214.x.

(16) Zhou, L. M.; Gu, Z. Q.; Costa, A. M.; Yamada, K. A.; Mansson, P. E.; Giordano, T.; Skolnick, P.; Jones, K. A. (2S,4R)-4-Methylglutamic Acid (SYM 2081): A Selective, High-Affinity Ligand for Kainate Receptors. *J. Pharmacol. Exp. Ther.* **1997**, *280* (1), 422–427.
(17) Eurofins Discovery https://www.eurofinsdiscoveryservices.com/catalogmanagement/viewItem/Glutamate-NMDA-Non-Selective-Rat-Ion-Channel-3H-MK-801-Binding-Assay-Panlabs/233010 (accessed 2021 -12 -15).
(18) Eurofins Discovery https://www.eurofinsdiscoveryservices.com/catalogmanagement/viewItem/Glutamate-NMDA-Glycine-3H-MDL-105-519-Binding-Assay-Panlabs/232910 (accessed 2021 -12 -15).
(19) Eurofins Discovery https://www.eurofinsdiscoveryservices.com/catalogmanagement/viewItem/Non-Selective-Rat-Glycine-Ion-Channel-3H-Strychnine-Binding-Assay-Panlabs/239000 (accessed 2021 -12 -15).
(20) Eurofins Discovery https://www.eurofinsdiscoveryservices.com/catalogmanagement/viewItem/GLYT1-Rat-Glycine-Transporter-Binding-Antagonist-Radioligand-Assay-Panlabs/239100 (accessed 2021 -12 -15).
(21) Eurofins Discovery https://www.eurofinsdiscoveryservices.com/catalogmanagement/viewItem/mGlu2-Human-Glutamate-Metabotropic-GPCR-Binding-Antagonist-Radioligand-Assay-Panlabs/236400 (accessed 2021 -12 -15).
(22) Mutel, V.; Ellis, G. J.; Adam, G.; Chaboz, S.; Nilly, A.; Messer, J.; Bleuel, Z.; Metzler, V.; Malherbe, P.; Schlaeger, E. J.; Roughley, B. S.; Faull, R. L.; Richards, J. G. Characterization of [(3)H]Quisqualate Binding to Recombinant Rat Metabotropic Glutamate 1a and 5a Receptors and to Rat and Human Brain Sections. *J. Neurochem.* **2000**, *75* (6), 2590–2601. https://doi.org/10.1046/j.1471-4159.2000.0752590.x.
(23) Brown, G. B. 3H-Batrachotoxinin-A Benzoate Binding to Voltage-Sensitive Sodium Channels: Inhibition by the Channel Blockers Tetrodotoxin and Saxitoxin. *J. Neurosci. Off. J. Soc. Neurosci.* **1986**, *6* (7), 2064–2070.
(24) Sills, M. A.; Fagg, G.; Pozza, M.; Angst, C.; Brundish, D. E.; Hurt, S. D.; Wilusz, E. J.; Williams, M. [3H]CGP 39653: A New N-Methyl-D-Aspartate Antagonist Radioligand with Low Nanomolar Affinity in Rat Brain. *Eur. J. Pharmacol.* **1991**, *192* (1), 19–24. https://doi.org/10.1016/0014-2999(91)90063-v.
(25) Huang, X.-P.; Mangano, T.; Hufeisen, S.; Setola, V.; Roth, B. L. Identification of Human Ether-à-Go-Go Related Gene Modulators by Three Screening Platforms in an Academic Drug-Discovery Setting. *Assay Drug Dev. Technol.* **2010**, *8* (6), 727–742. https://doi.org/10.1089/adt.2010.0331.
(26) Eurofins Discovery https://www.eurofinsdiscoveryservices.com/catalogmanagement/viewItem/sigma-Non-Selective-Guinea-Pig-Binding-Agonist-Radioligand-Assay-Panlabs/278300 (accessed 2021 -12 -15).
(27) Ellman, G. L.; Courtney, K. D.; Andres, V.; Feather-Stone, R. M. A New and Rapid Colorimetric Determination of Acetylcholinesterase Activity. *Biochem. Pharmacol.* **1961**, *7*, 88–95. https://doi.org/10.1016/0006-2952(61)90145-9.
(28) Vicentic, A.; Robeva, A.; Rogge, G.; Uberti, M.; Minneman, K. P. Biochemistry and Pharmacology of Epitope-Tagged Alpha(1)-Adrenergic Receptor Subtypes. *J. Pharmacol. Exp. Ther.* **2002**, *302* (1), 58–65. https://doi.org/10.1124/jpet.302.1.58.
(29) Felder, C. C.; Joyce, K. E.; Briley, E. M.; Mansouri, J.; Mackie, K.; Blond, O.; Lai, Y.; Ma, A. L.; Mitchell, R. L. Comparison of the Pharmacology and Signal Transduction of the Human Cannabinoid CB1 and CB2 Receptors. *Mol. Pharmacol.* **1995**, *48* (3), 443–450.
(30) Zhou, Q. Y.; Grandy, D. K.; Thambi, L.; Kushner, J. A.; Van Tol, H. H.; Cone, R.; Pribnow, D.; Salon, J.; Bunzow, J. R.; Civelli, O. Cloning and Expression of Human and Rat D1 Dopamine Receptors. *Nature* **1990**, *347* (6288), 76–80. https://doi.org/10.1038/347076a0.

(31) Grandy, D. K.; Marchionni, M. A.; Makam, H.; Stofko, R. E.; Alfano, M.; Frothingham, L.; Fischer, J. B.; Burke-Howie, K. J.; Bunzow, J. R.; Server, A. C. Cloning of the CDNA and Gene for a Human D2 Dopamine Receptor. *Proc. Natl. Acad. Sci. U. S. A.* **1989**, *86* (24), 9762–9766. https://doi.org/10.1073/pnas.86.24.9762.
(32) Gillies, G. Somatostatin: The Neuroendocrine Story. *Trends Pharmacol. Sci.* **1997**, *18* (3), 87–95. https://doi.org/10.1016/s0165-6147(96)01032-2.
(33) Engström, M.; Tomperi, J.; El-Darwish, K.; Ahman, M.; Savola, J.-M.; Wurster, S. Superagonism at the Human Somatostatin Receptor Subtype 4. *J. Pharmacol. Exp. Ther.* **2005**, *312* (1), 332–338. https://doi.org/10.1124/jpet.104.075531.
(34) Kohen, R.; Metcalf, M. A.; Khan, N.; Druck, T.; Huebner, K.; Lachowicz, J. E.; Meltzer, H. Y.; Sibley, D. R.; Roth, B. L.; Hamblin, M. W. Cloning, Characterization, and Chromosomal Localization of a Human 5-HT6 Serotonin Receptor. *J. Neurochem.* **1996**, *66* (1), 47–56. https://doi.org/10.1046/j.1471-4159.1996.66010047.x.
(35) Adham, N.; Zgombick, J. M.; Bard, J.; Branchek, T. A. Functional Characterization of the Recombinant Human 5-Hydroxytryptamine7(a) Receptor Isoform Coupled to Adenylate Cyclase Stimulation. *J. Pharmacol. Exp. Ther.* **1998**, *287* (2), 508–514.
(36) Pristupa, Z. B.; Wilson, J. M.; Hoffman, B. J.; Kish, S. J.; Niznik, H. B. Pharmacological Heterogeneity of the Cloned and Native Human Dopamine Transporter: Disassociation of [3H]WIN 35,428 and [3H]GBR 12,935 Binding. *Mol. Pharmacol.* **1994**, *45* (1), 125–135.
(37) Pacholczyk, T.; Blakely, R. D.; Amara, S. G. Expression Cloning of a Cocaine- and Antidepressant-Sensitive Human Noradrenaline Transporter. *Nature* **1991**, *350* (6316), 350–354. https://doi.org/10.1038/350350a0.
(38) Tatsumi, M.; Jansen, K.; Blakely, R. D.; Richelson, E. Pharmacological Profile of Neuroleptics at Human Monoamine Transporters. *Eur. J. Pharmacol.* **1999**, *368* (2–3), 277–283. https://doi.org/10.1016/s0014-2999(99)00005-9.
(39) Forster, Y. M.; Green, J. L.; Khatiwada, A.; Liberato, J. L.; Narayana Reddy, P. A.; Salvino, J. M.; Bienz, S.; Bigler, L.; Dos Santos, W. F.; Karklin Fontana, A. C. Elucidation of the Structure and Synthesis of Neuroprotective Low Molecular Mass Components of the Parawixia Bistriata Spider Venom. *ACS Chem. Neurosci.* **2020**, *11* (11), 1573–1596. https://doi.org/10.1021/acschemneuro.0c00007.
(40) Pasierski, M.; Szulczyk, B. Capsaicin Inhibits Sodium Currents and Epileptiform Activity in Prefrontal Cortex Pyramidal Neurons. *Neurochem. Int.* **2020**, *135*, 104709. https://doi.org/10.1016/j.neuint.2020.104709.

UPLC/MS traces for intermediate and final compounds

The purity of all obtained compounds determined by use of chromatographic UPLC method was ≥99%.

(*R*)-*tert*-butyl-(1-(benzylamino)-1-oxopropan-2-yl)carbamate ***(R)*-1**

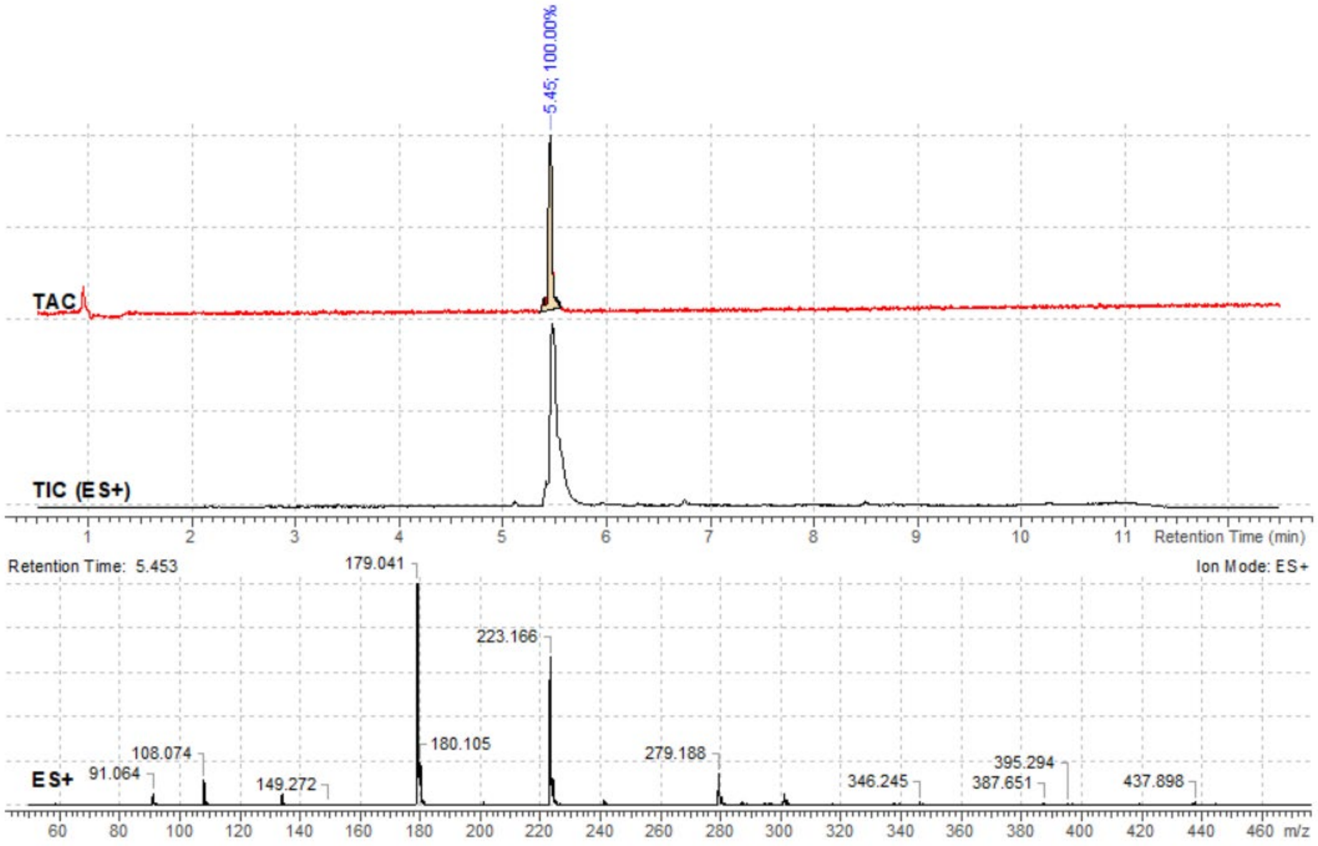

(*R*)-tert-butyl-(1-((2-fluorobenzyl)amino)-1-oxopropan-2-yl)carbamate **(*R*)-2**

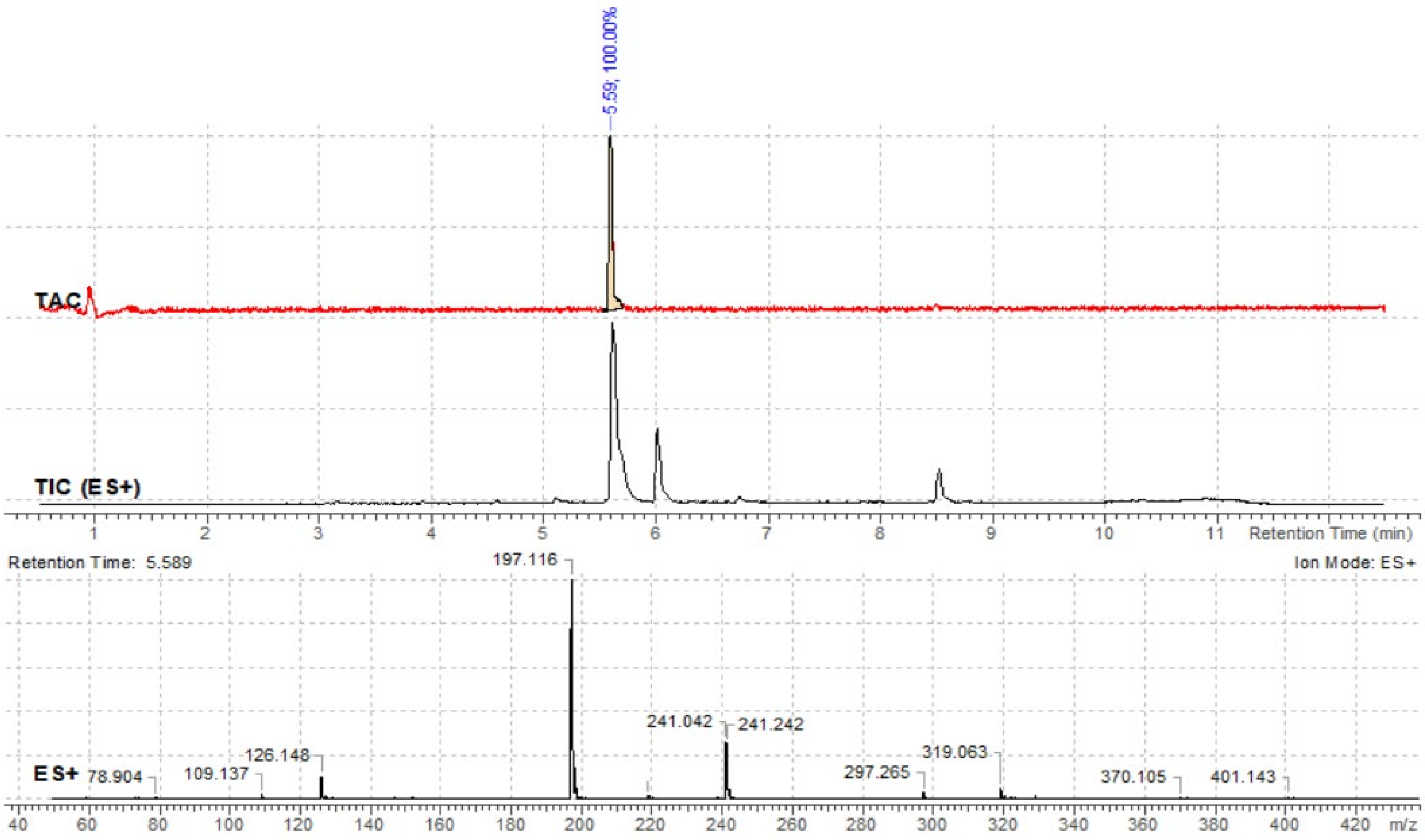

(*S*)-*tert*-butyl-(1-(benzylamino)-1-oxopropan-2-yl)carbamate **(*S*)-1**

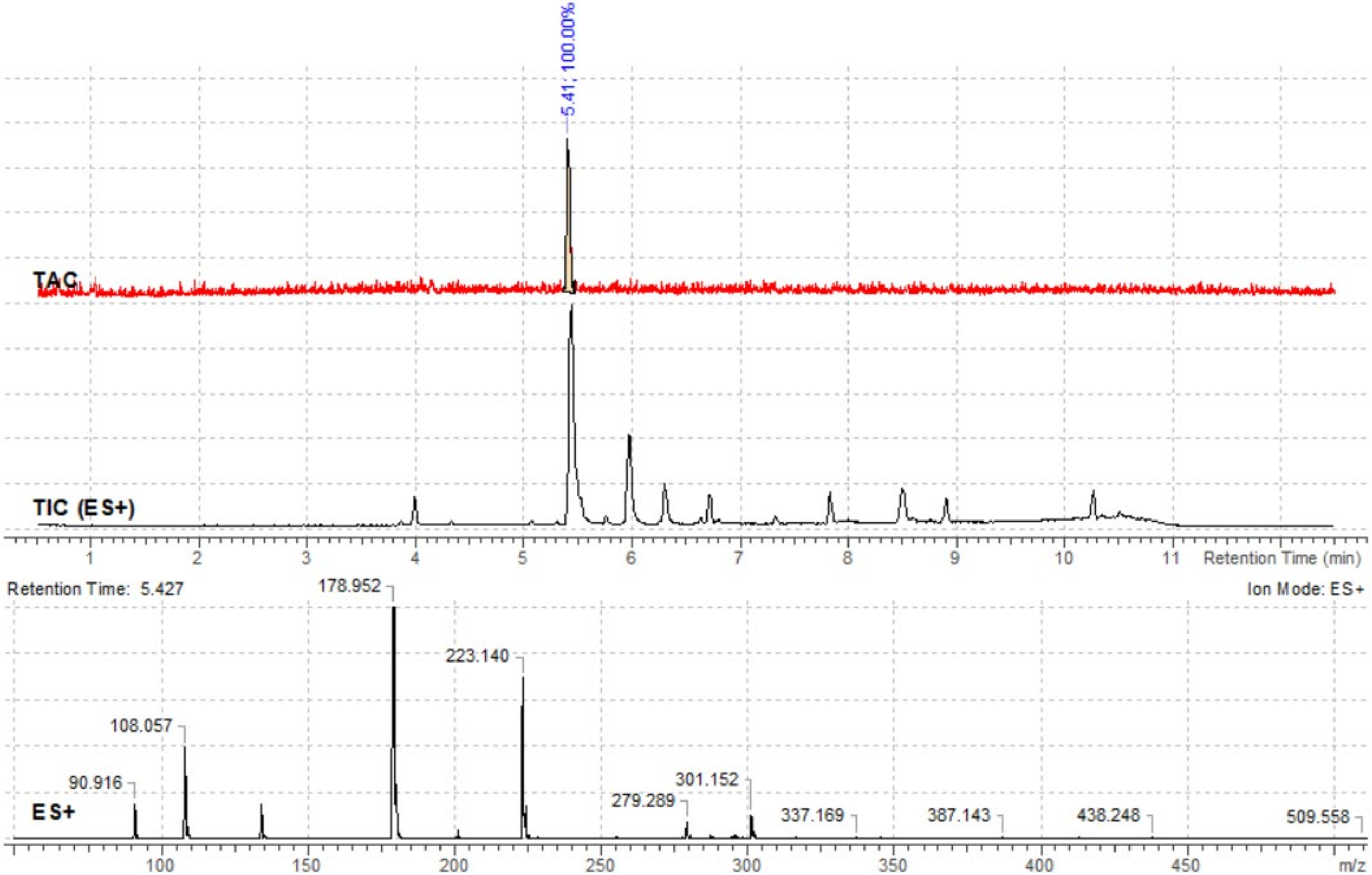

(*S*)-tert-butyl-(1-((2-fluorobenzyl)amino)-1-oxopropan-2-yl)carbamate **(*S*)-2**

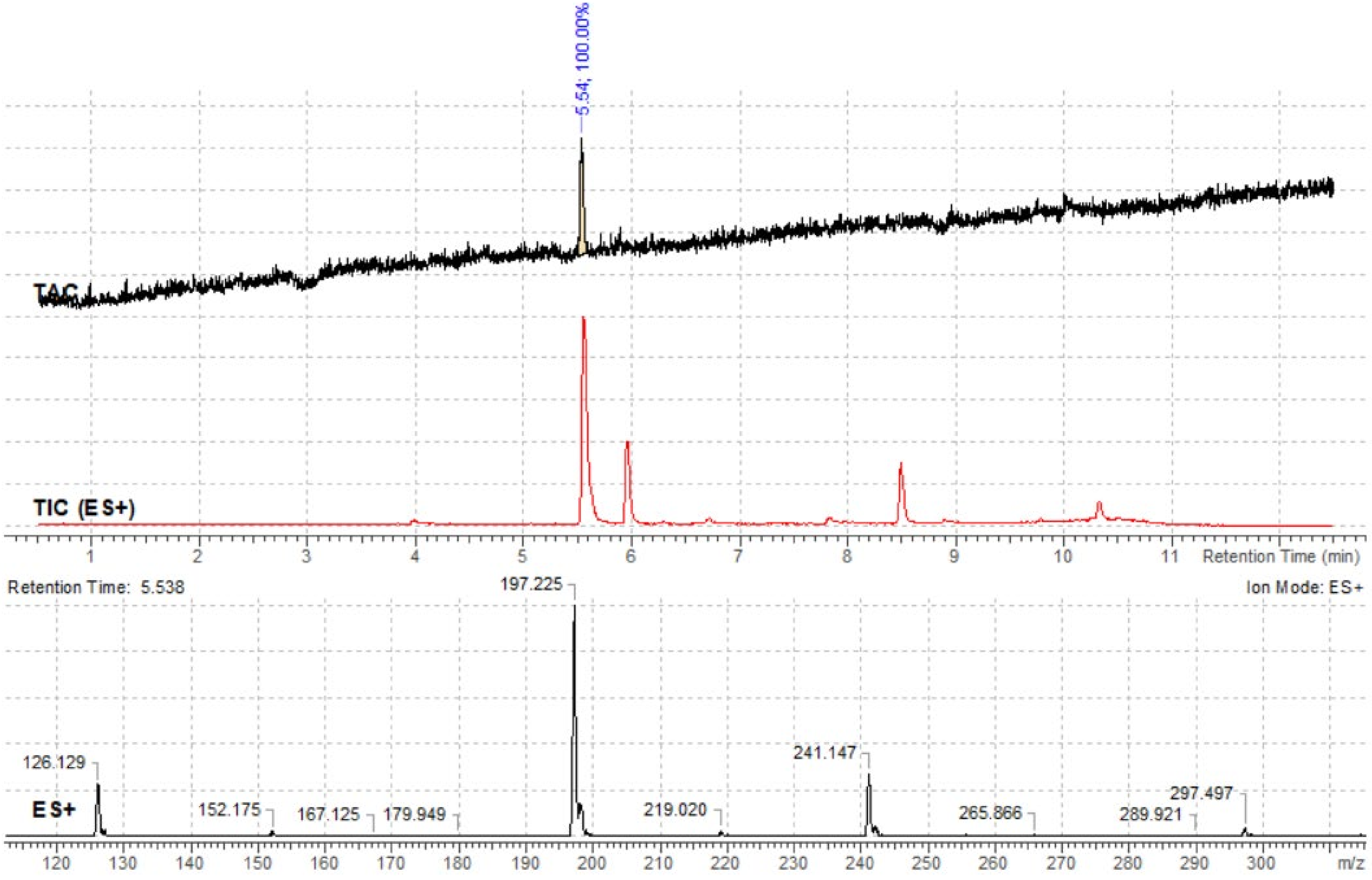

(*R*)-2-amino-*N*-benzylpropanamide ***(R)*-3**

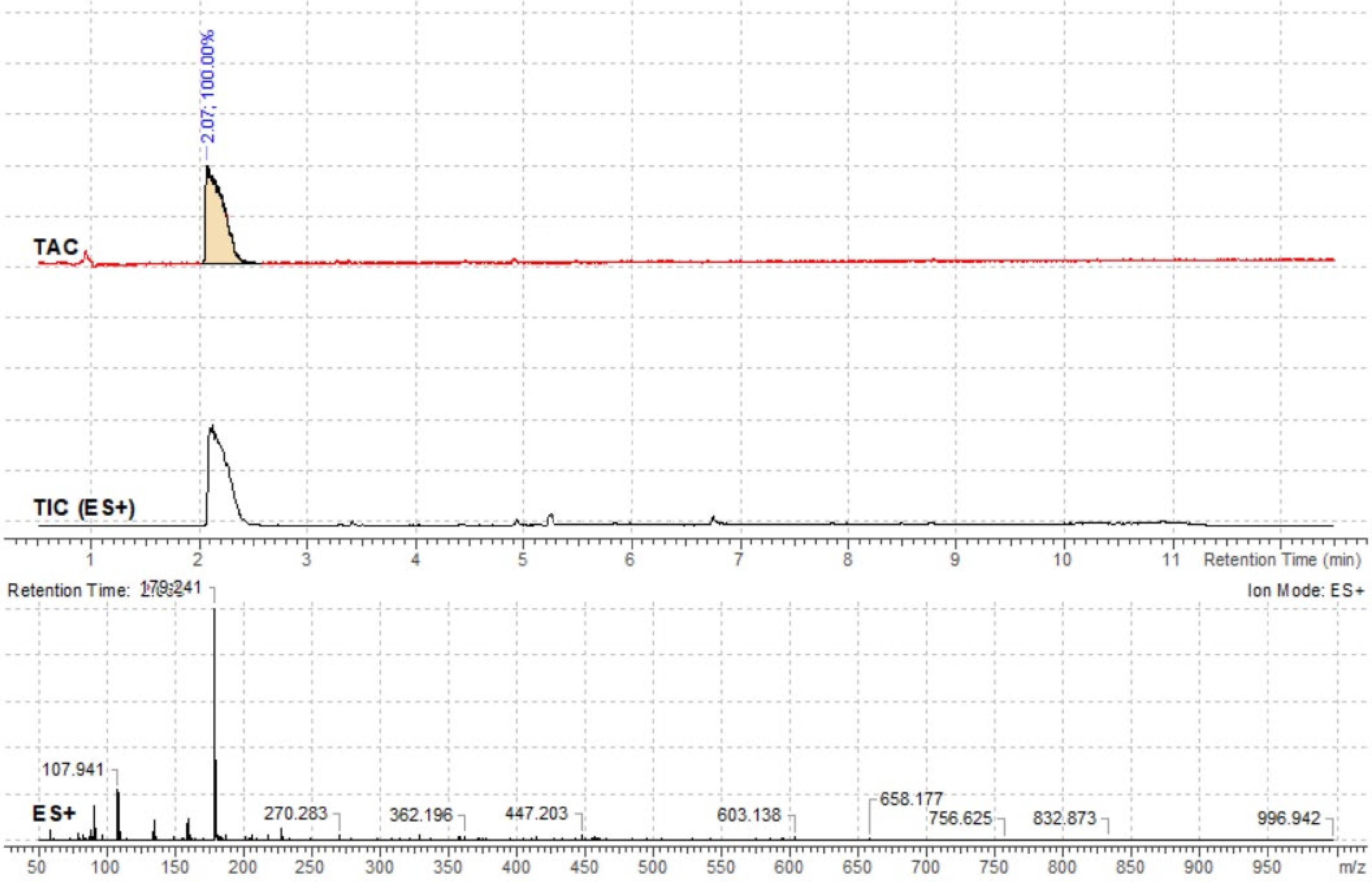

(*R*)-2-amino-*N*-(2-fluorobenzyl)propanamide **(*R*)-4**

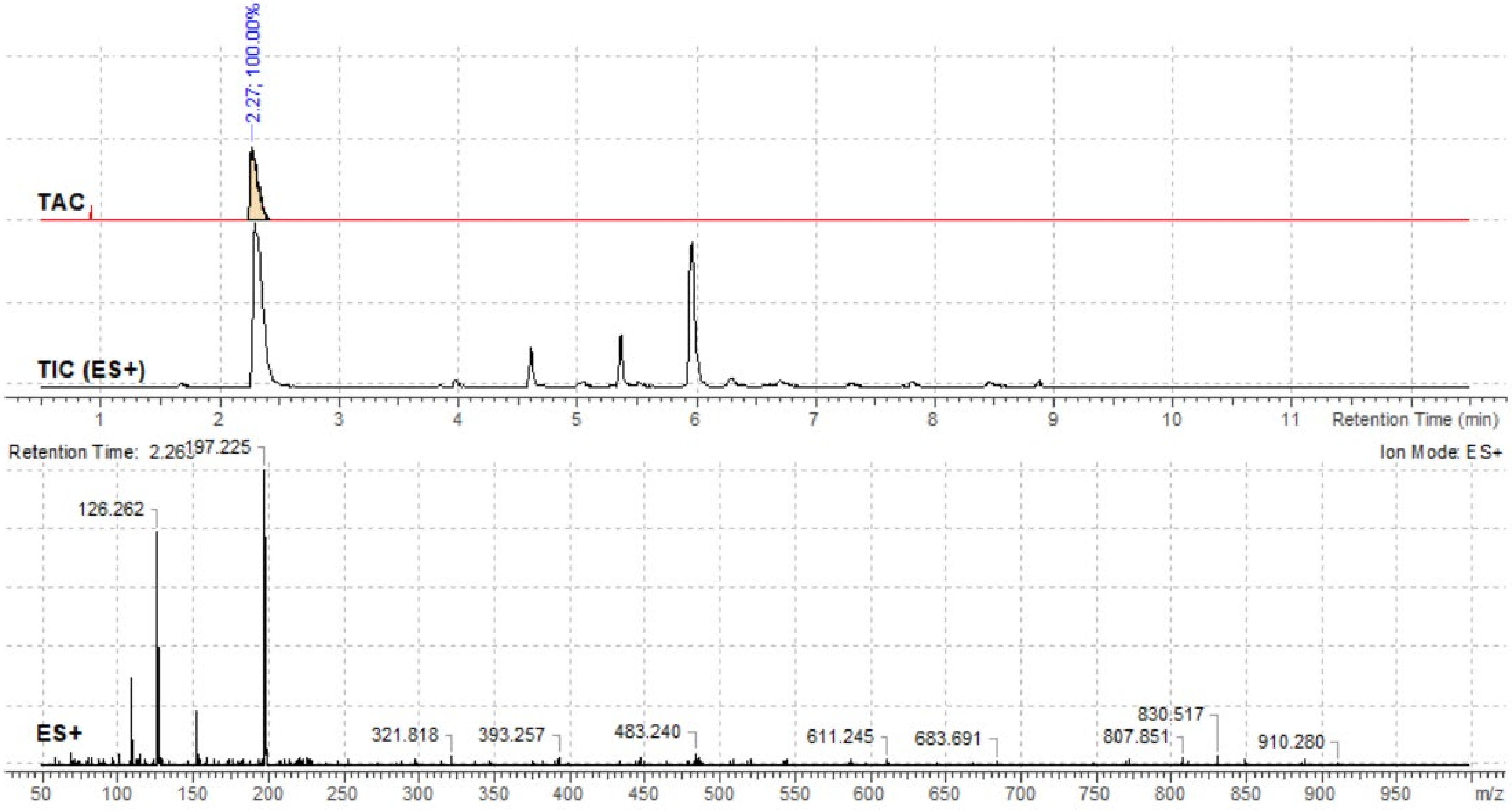

(*S*)-2-amino-*N*-benzylpropanamide **(*S*)-3**

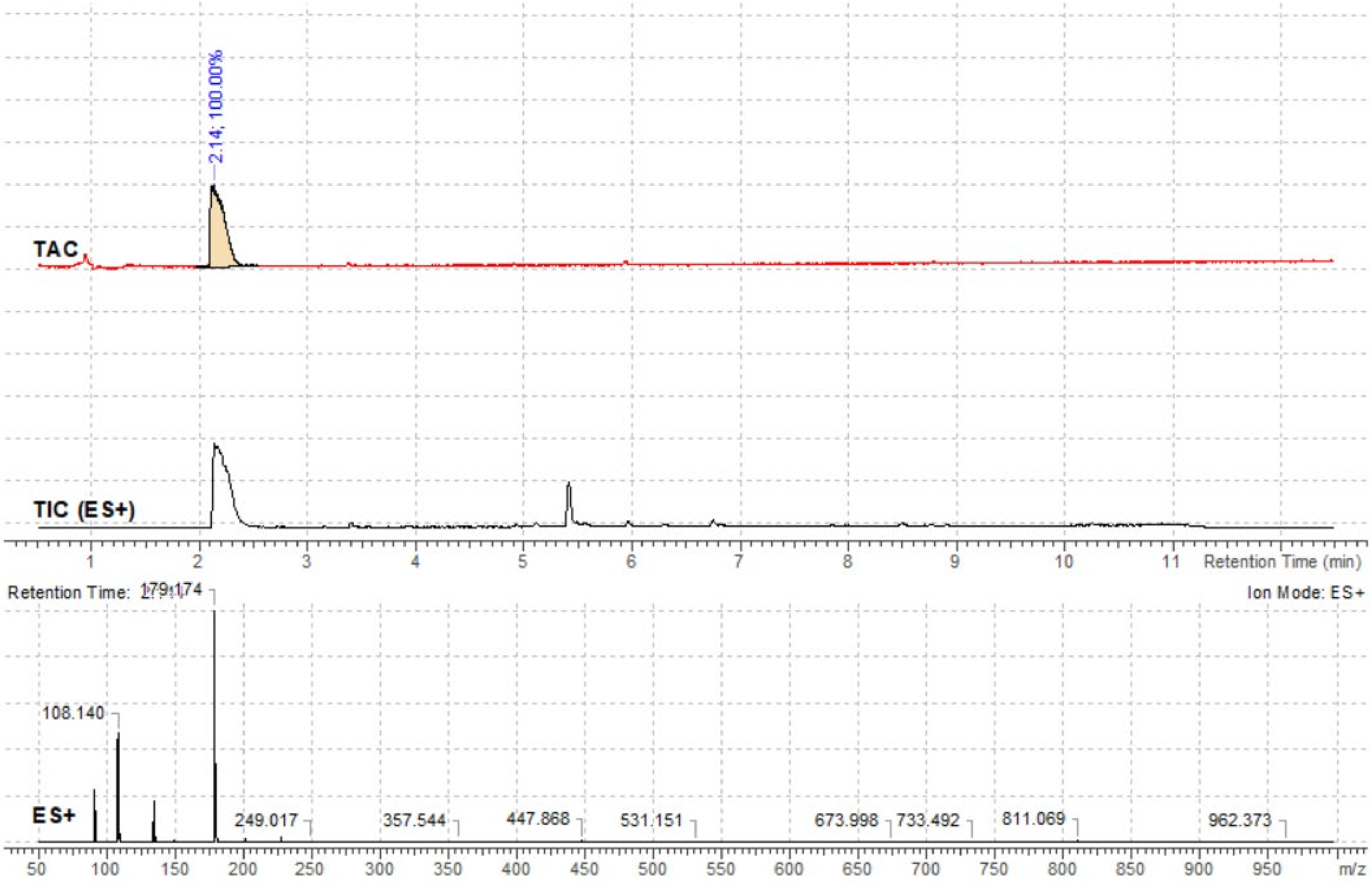

(*S*)-2-amino-*N*-(2-fluorobenzyl)propanamide **(*S*)-4**

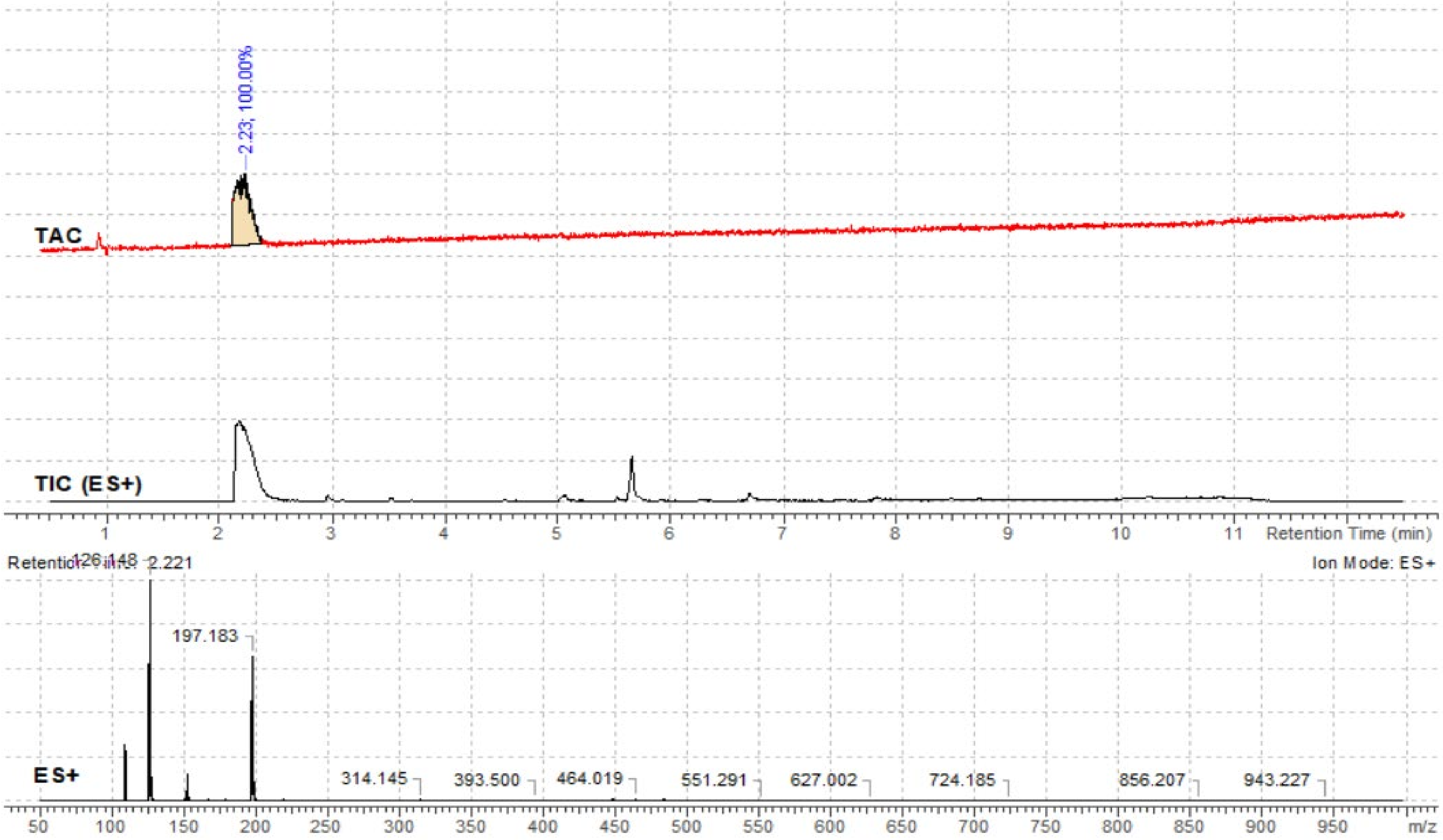

(*R*)-4-((1-(benzylamino)-1-oxopropan-2-yl)amino)-4-oxobutanoic acid **(*R*)-5**

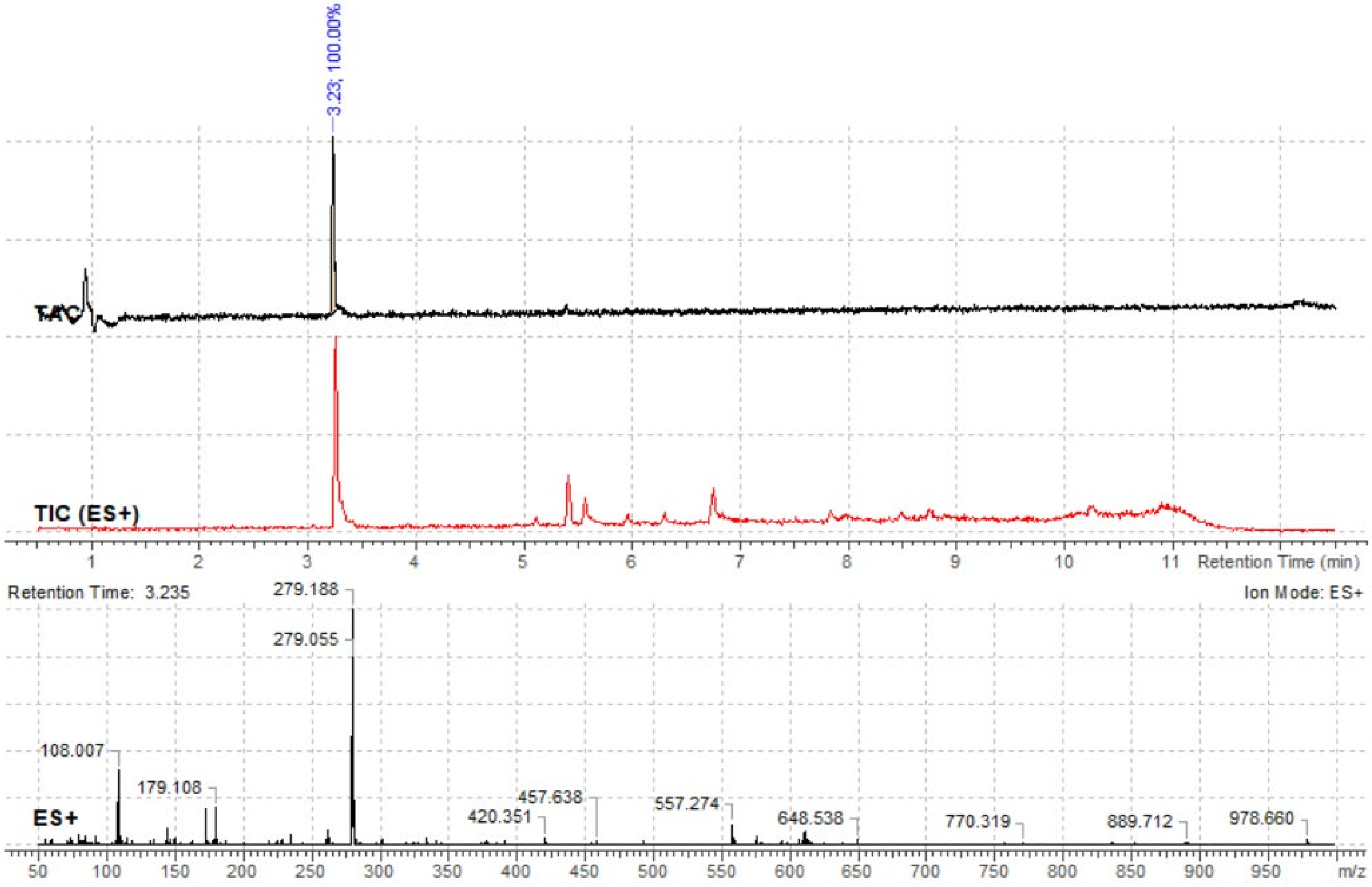

(*R*)-4-((1-((2-fluorobenzyl)amino)-1-oxopropan-2-yl)amino)-4-oxobutanoic acid **(*R*)-6**

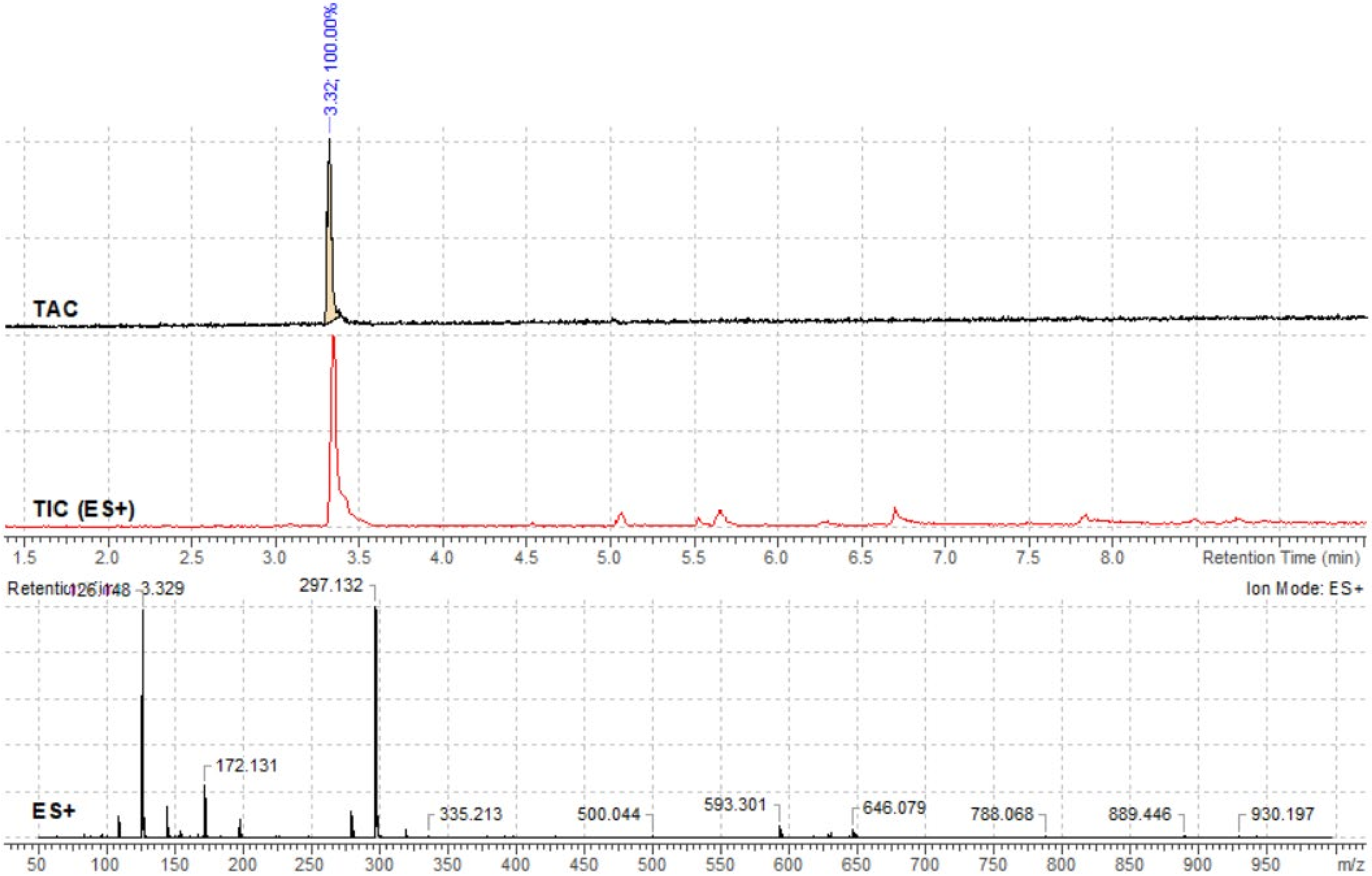

(*S*)-4-((1-(benzylamino)-1-oxopropan-2-yl)amino)-4-oxobutanoic acid **(*S*)-5**

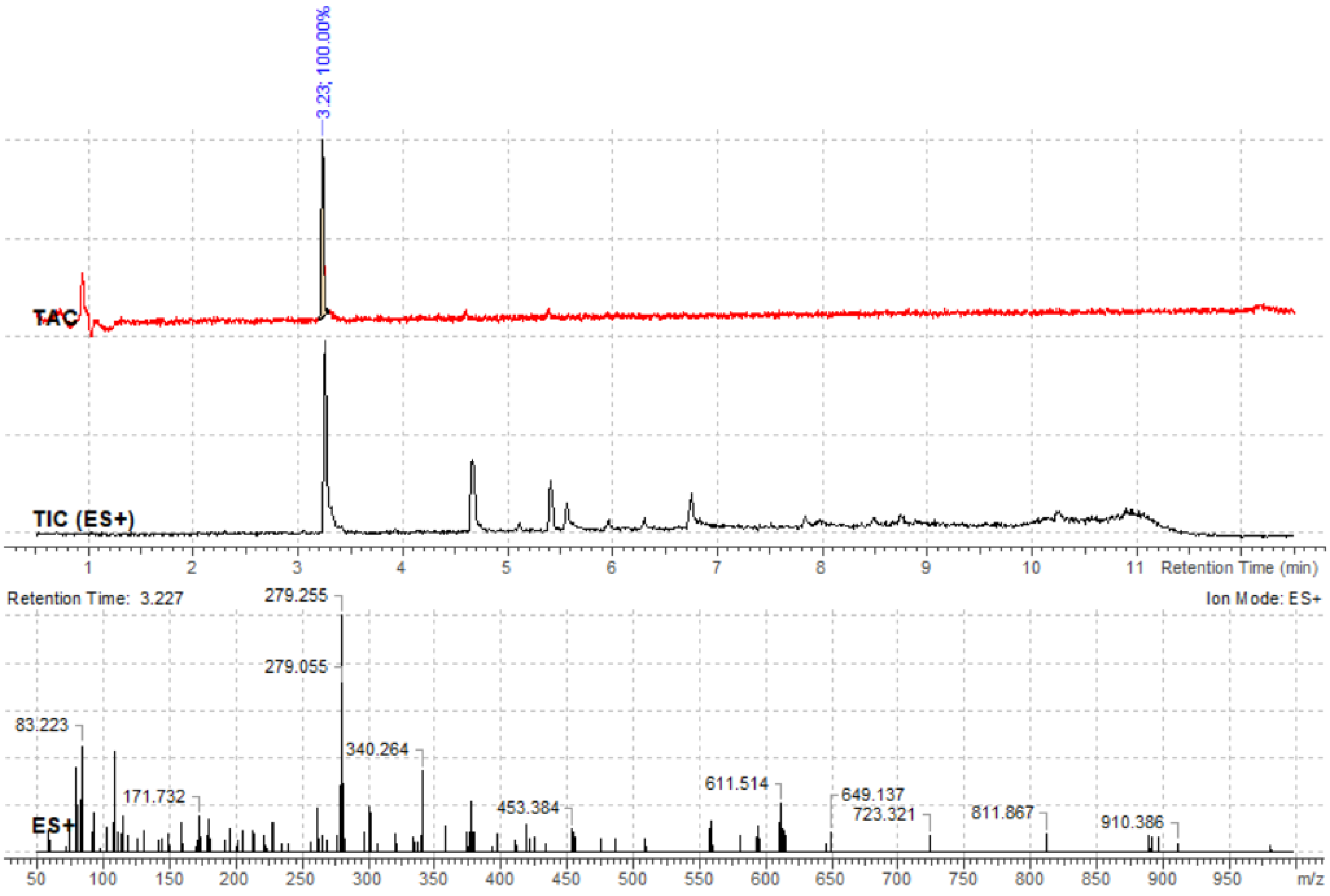

(*S*)-4-((1-((2-fluorobenzyl)amino)-1-oxopropan-2-yl)amino)-4-oxobutanoic acid **(*S*)-6**

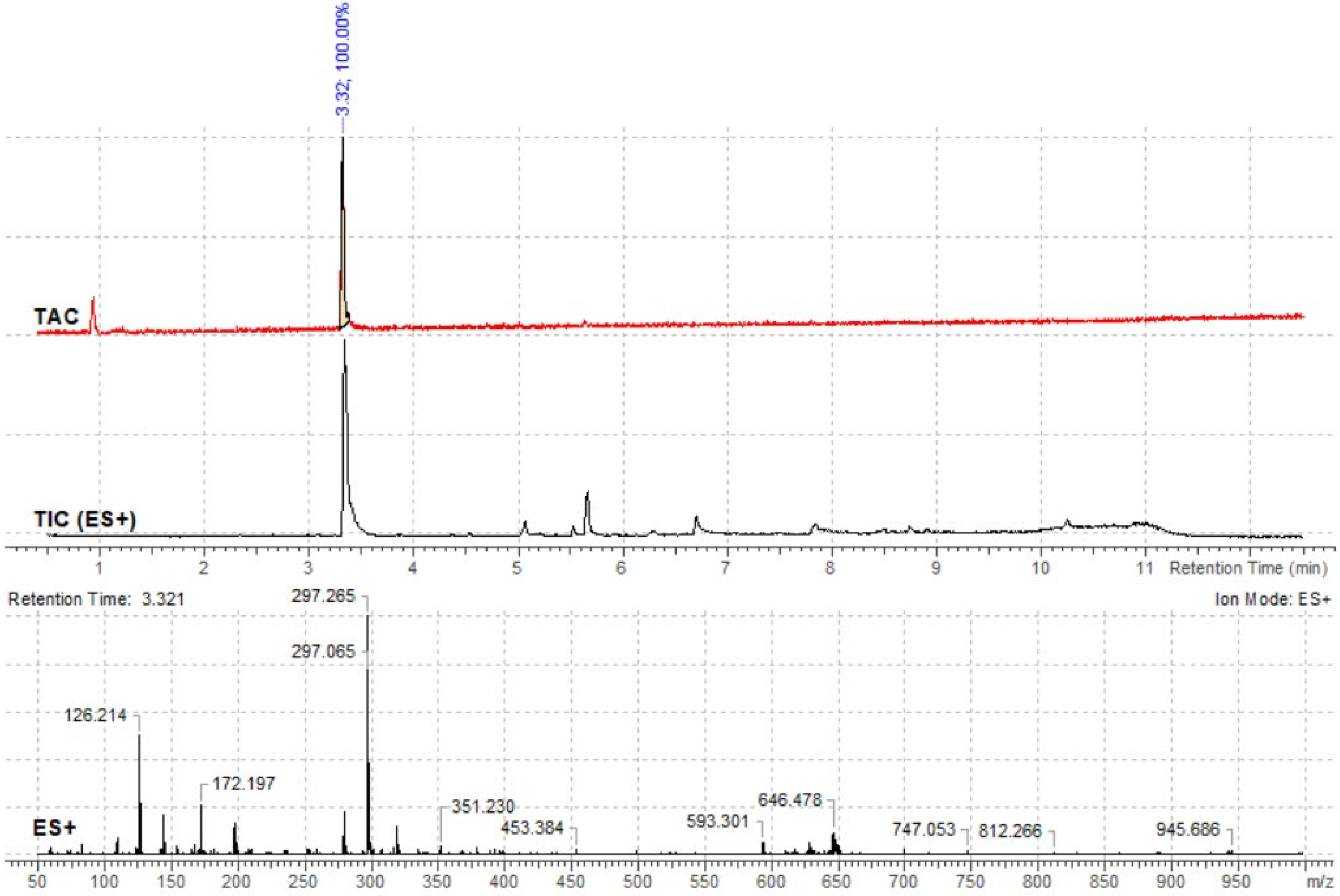

(*R*)-*N*-benzyl-2-(2,5-dioxopyrrolidin-1-yl)propanamide **(*R*)-7**

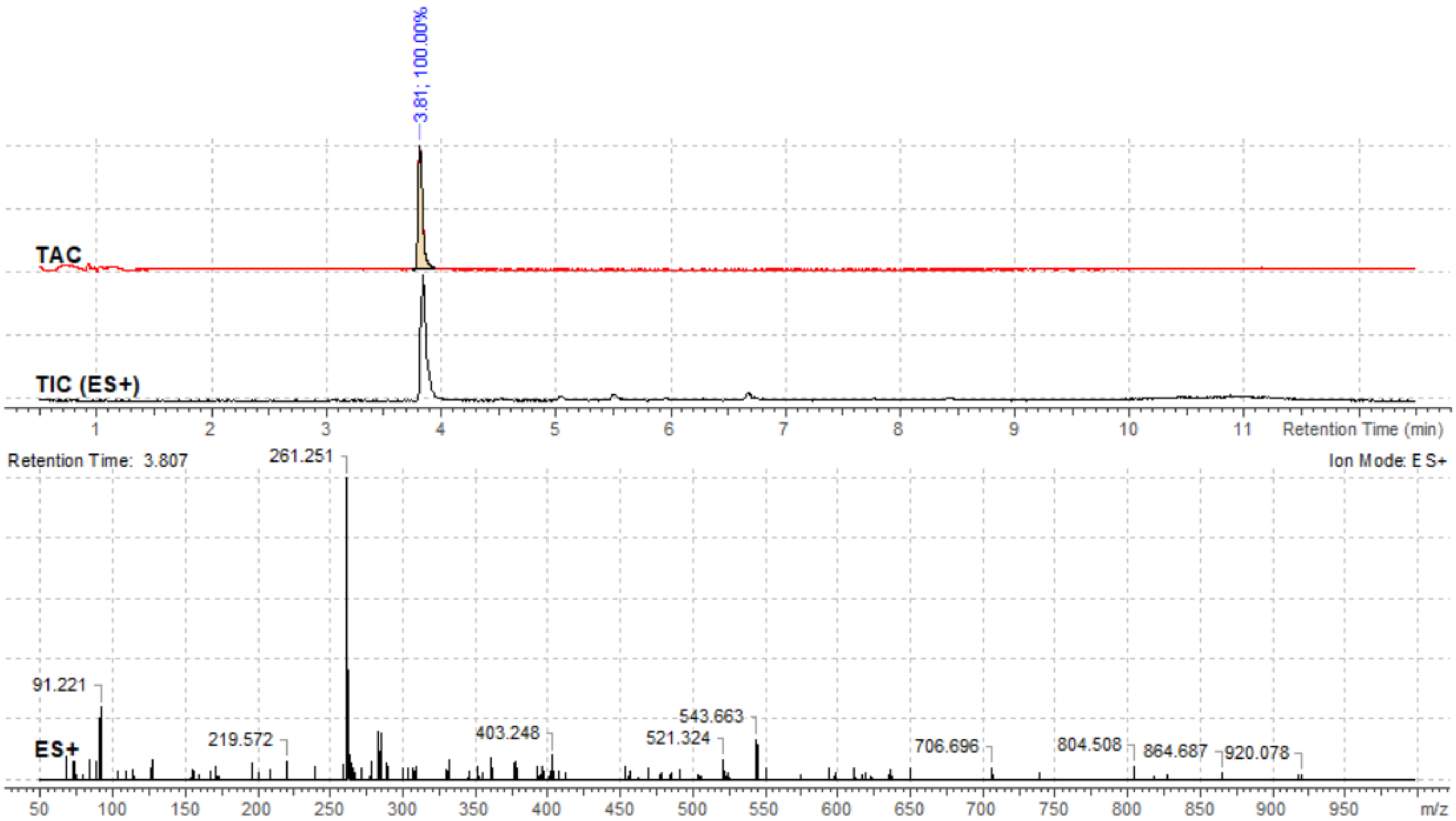

(*S*)-*N*-benzyl-2-(2,5-dioxopyrrolidin-1-yl)propanamide **(*S*)-7**

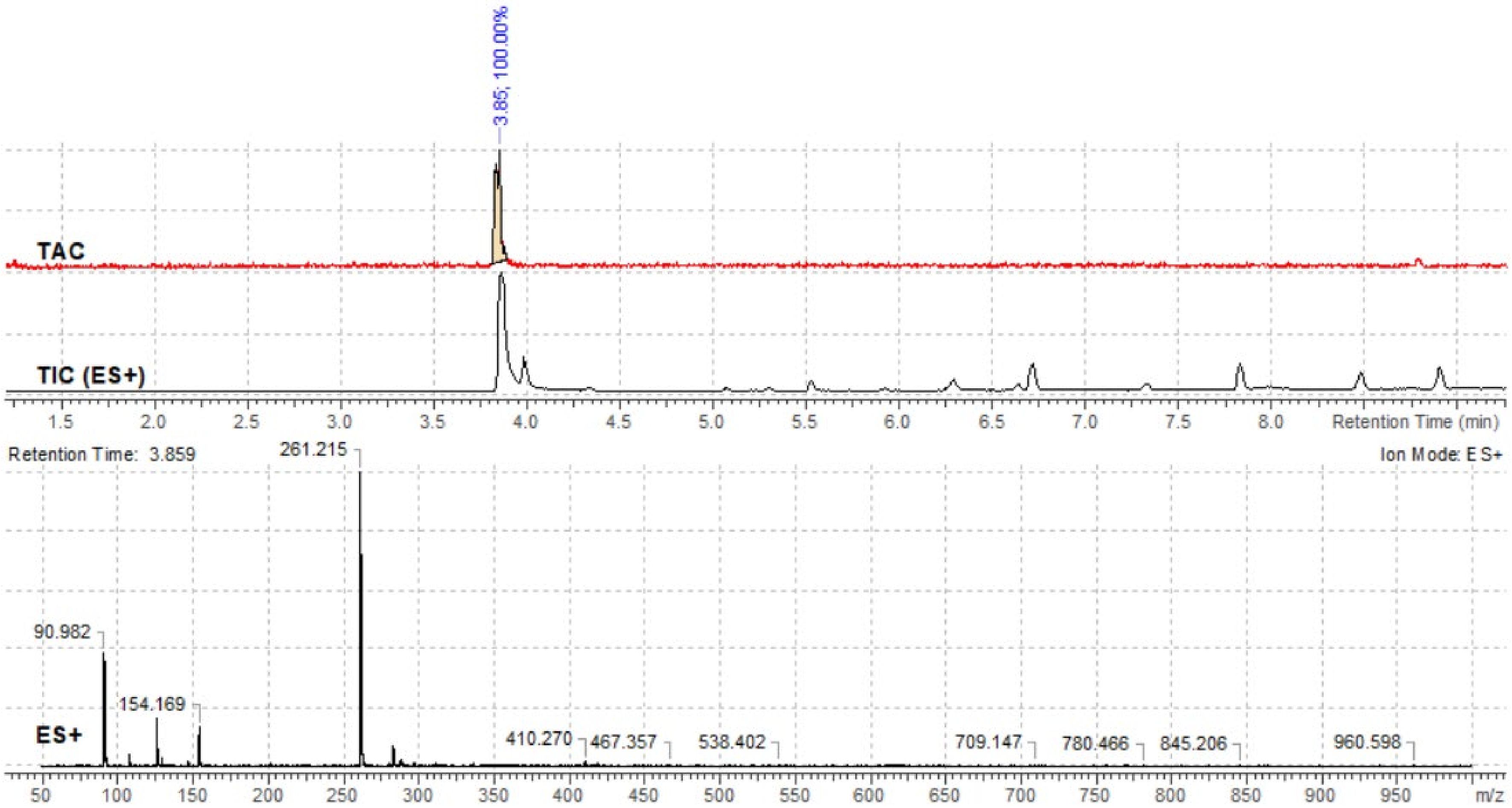

(*R*)-2-(2,5-dioxopyrrolidin-1-yl)-*N*-(2-fluorobenzyl)propanamide **(*R*)-8**

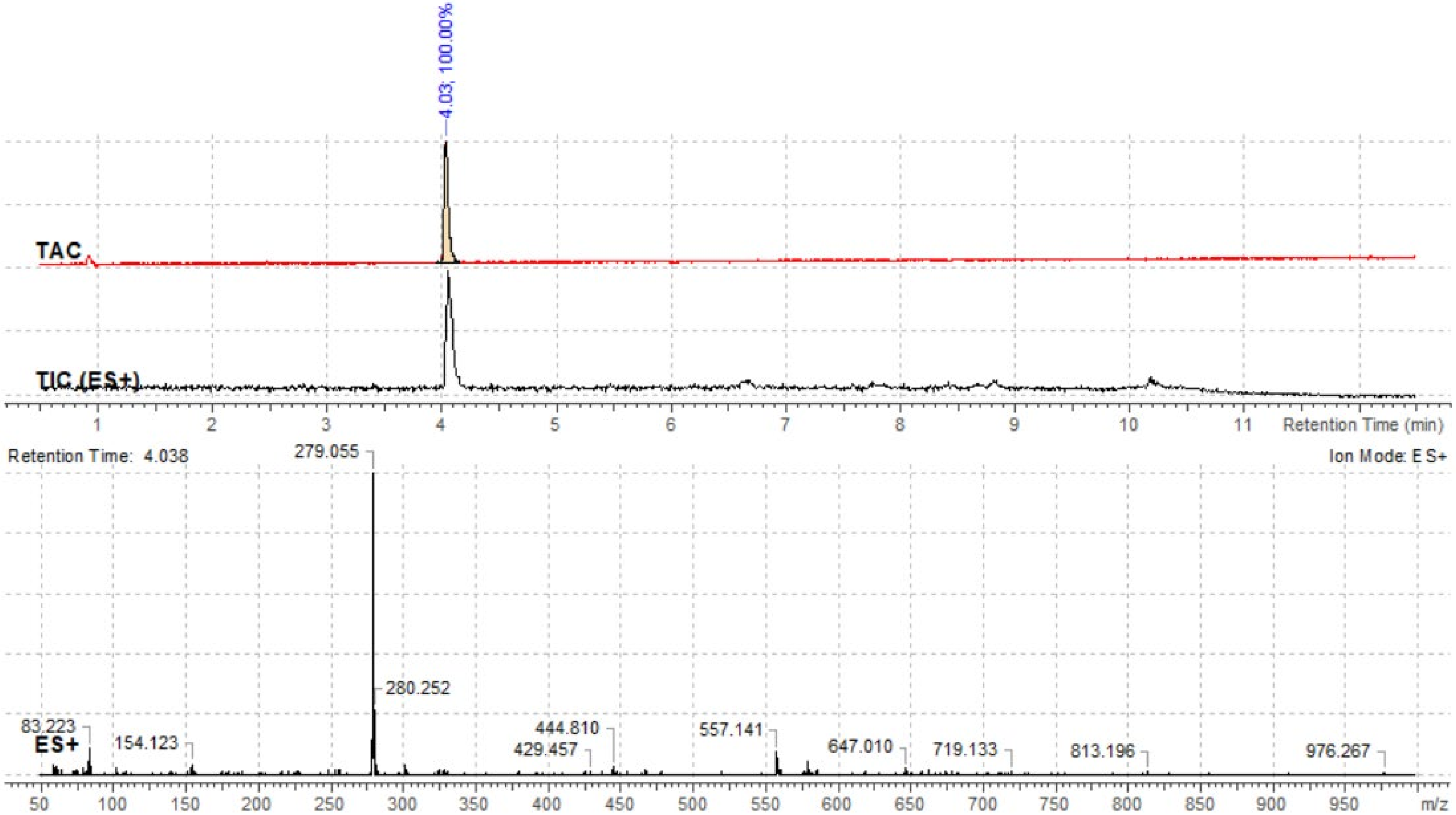

(*S*)-2-(2,5-dioxopyrrolidin-1-yl)-*N*-(2-fluorobenzyl)propanamide **(*S*)-8**

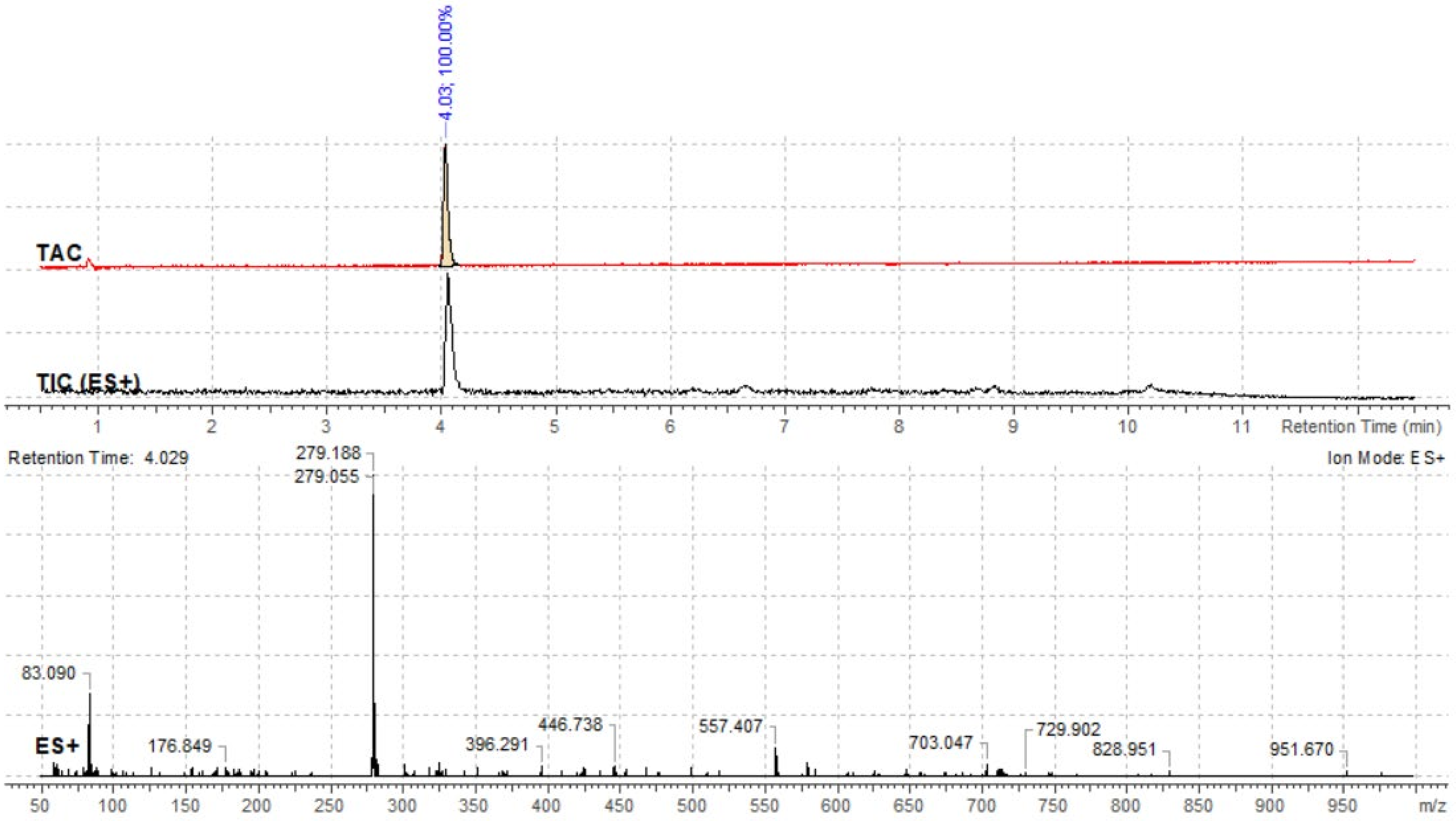

HMRS traces for final compounds

(*R*)-*N*-benzyl-2-(2,5-dioxopyrrolidin-1-yl)propanamide **(*R*)-7** ESI+

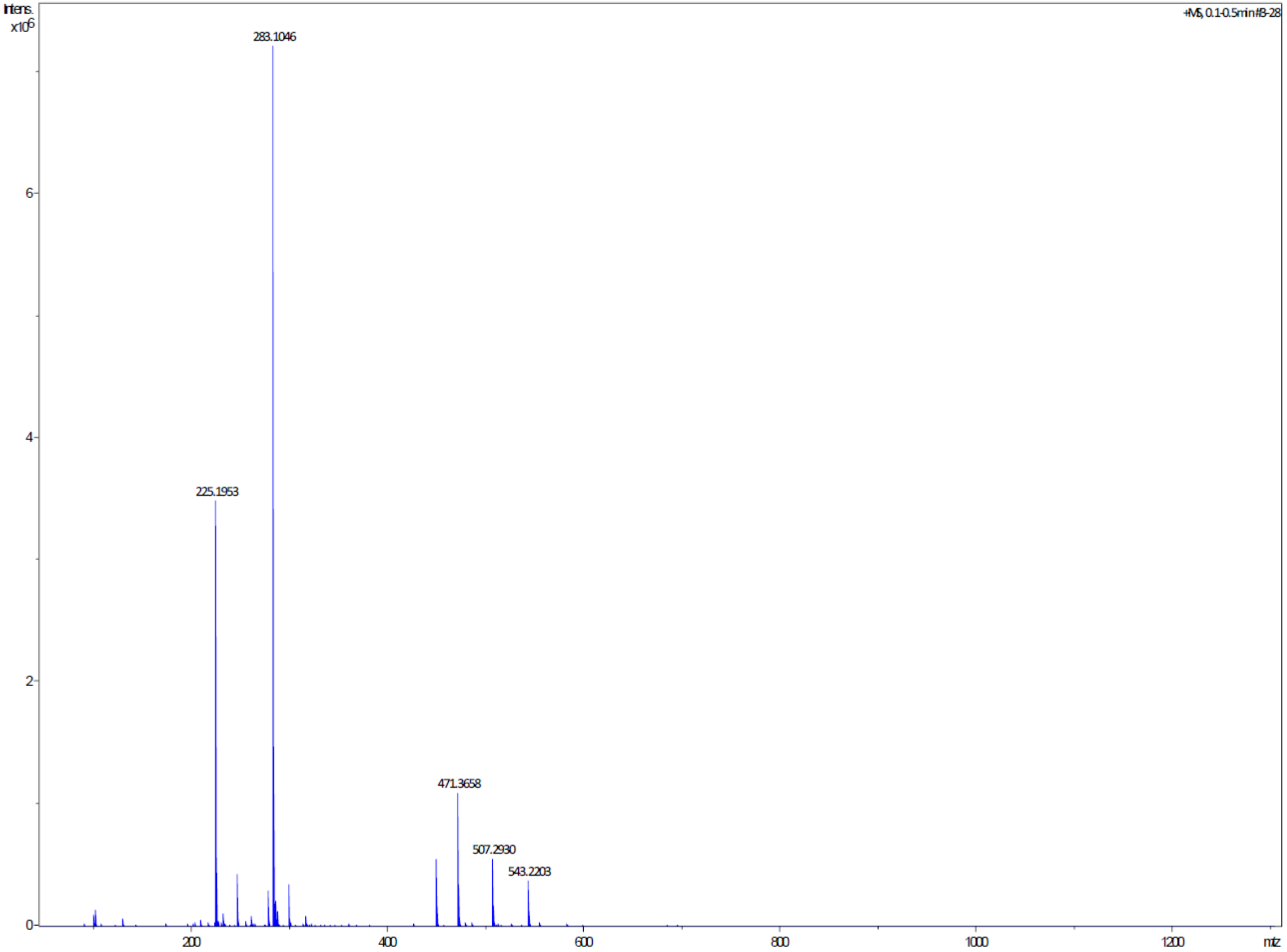

(*R*)-*N*-benzyl-2-(2,5-dioxopyrrolidin-1-yl)propanamide **(*R*)-7** ESI-

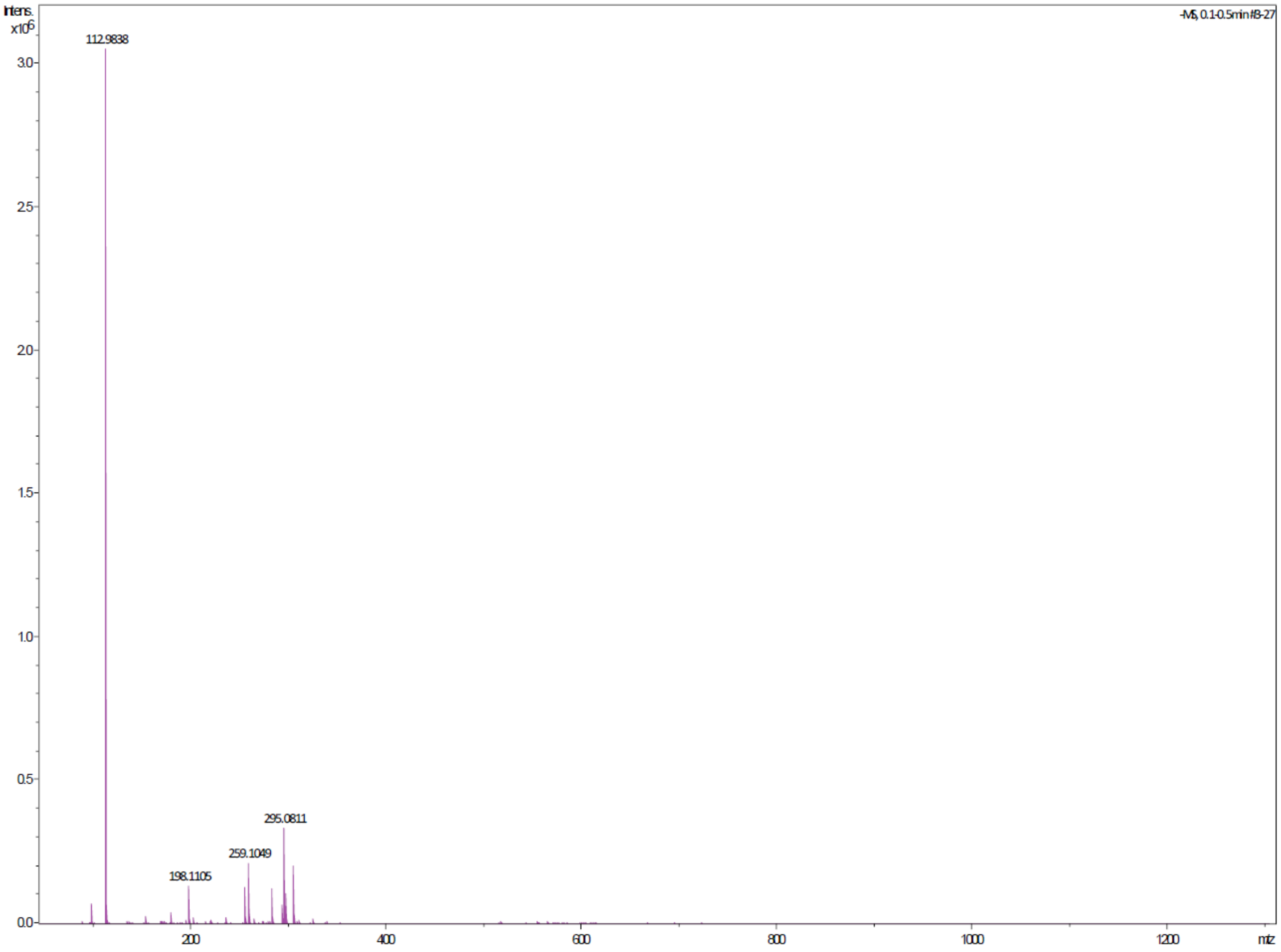

(*S*)-*N*-benzyl-2-(2,5-dioxopyrrolidin-1-yl)propanamide **(*S*)-7** ESI+

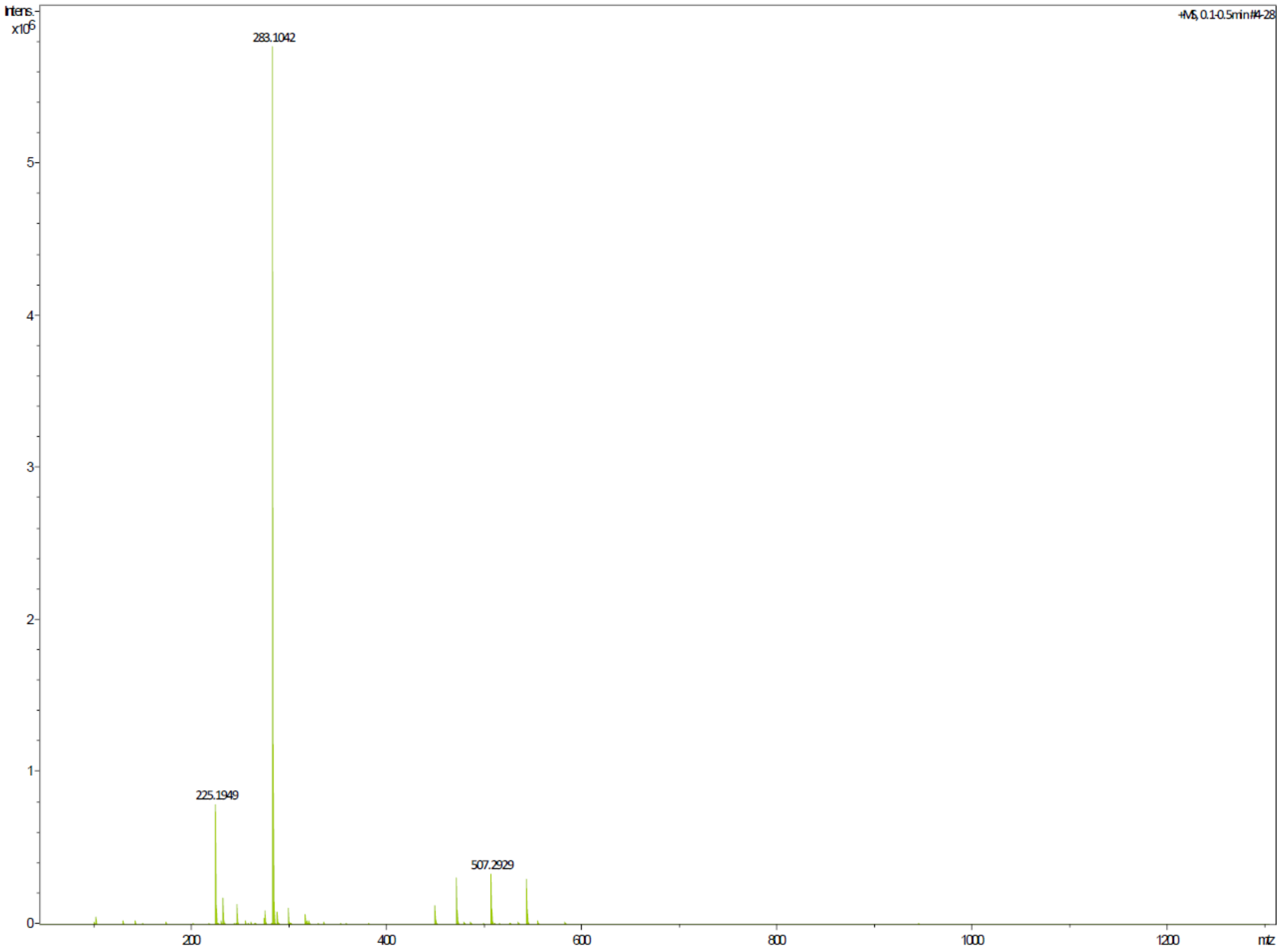

(*S*)-*N*-benzyl-2-(2,5-dioxopyrrolidin-1-yl)propanamide **(*S*)-7** ESI-

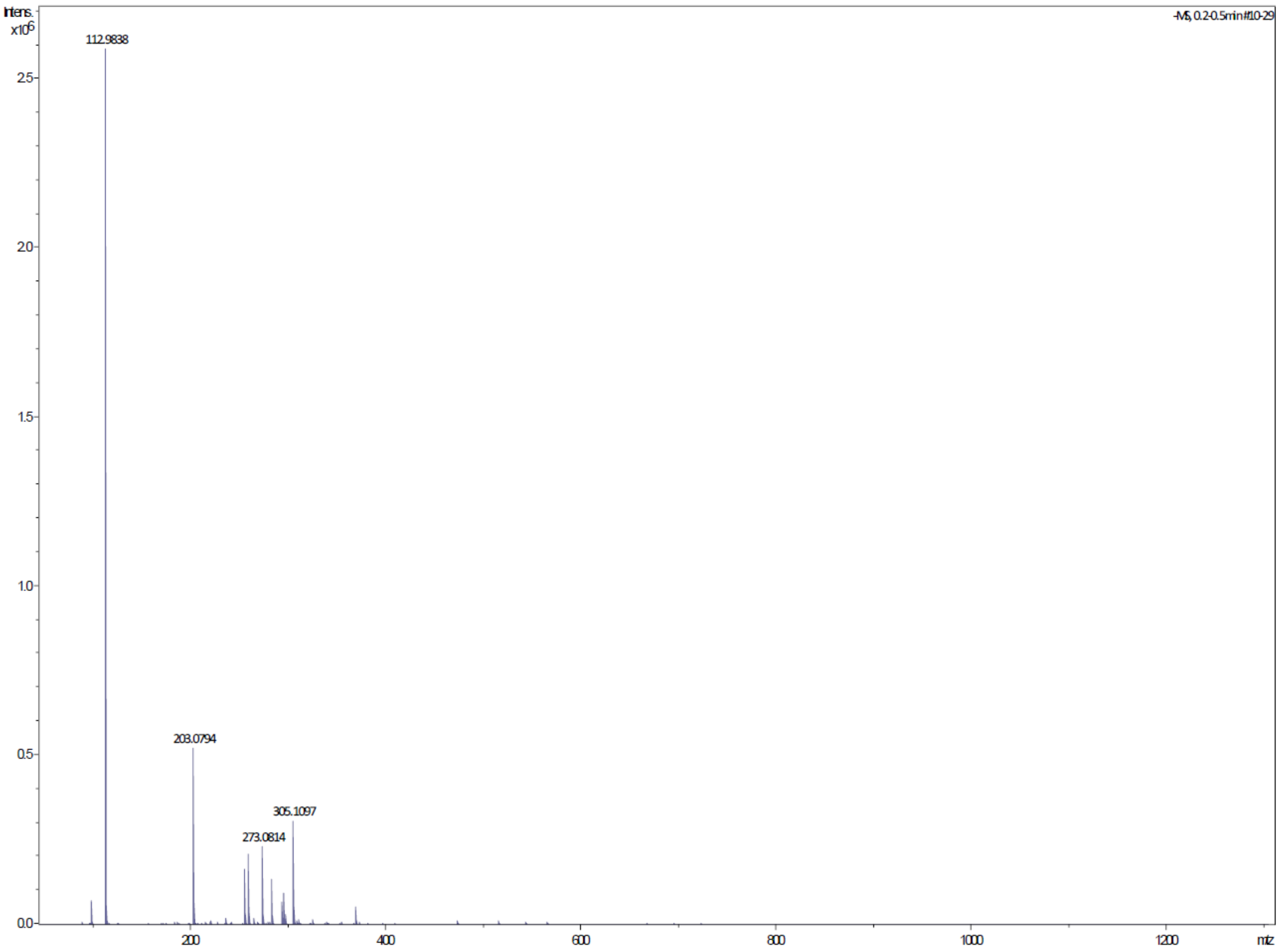

(*R*)-2-(2,5-dioxopyrrolidin-1-yl)-*N*-(2-fluorobenzyl)propanamide ***(R)*-8** ESI+

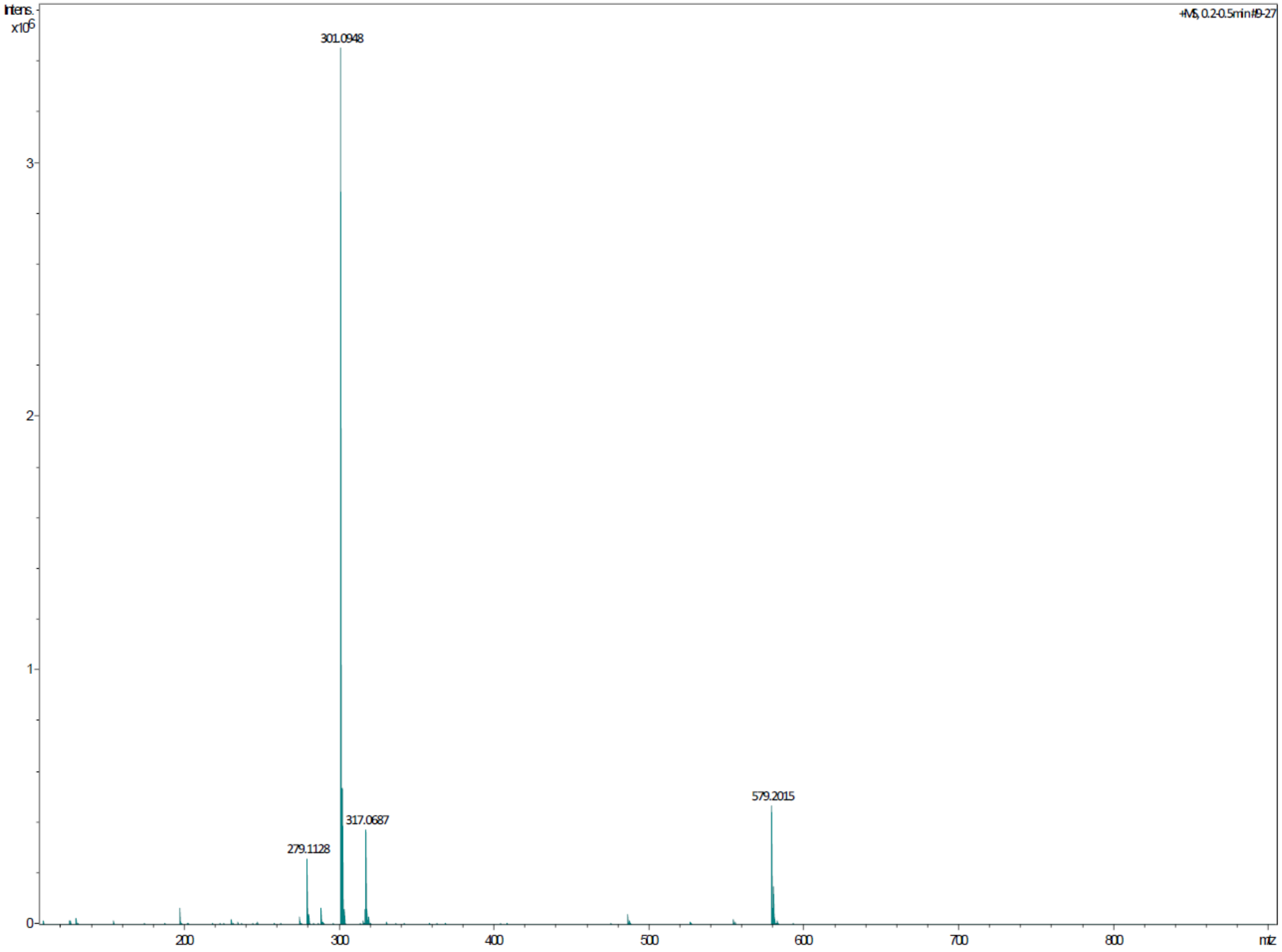

(*R*)-2-(2,5-dioxopyrrolidin-1-yl)-*N*-(2-fluorobenzyl)propanamide **(*R*)-8** ESI-

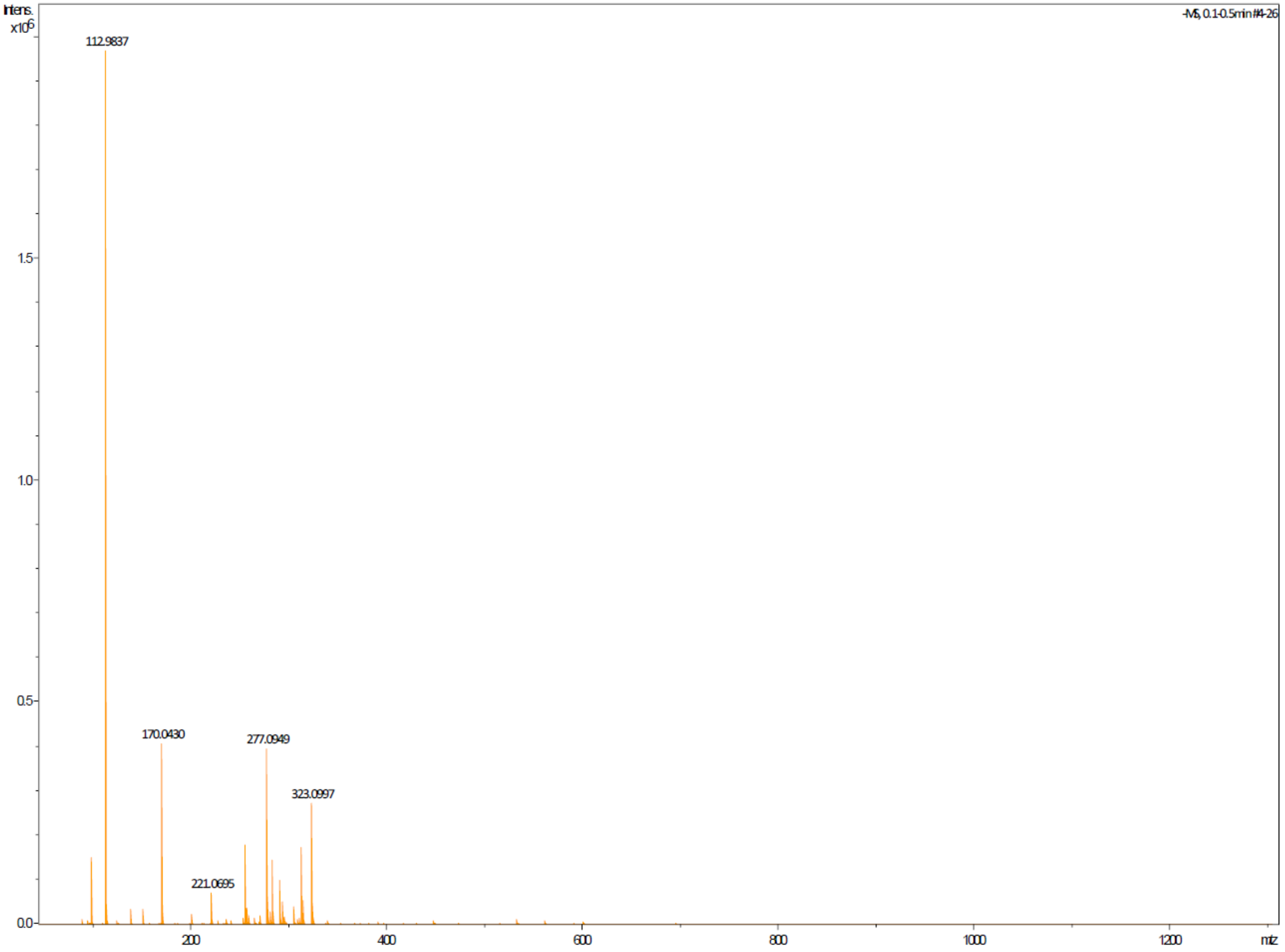

(*S*)-2-(2,5-dioxopyrrolidin-1-yl)-*N*-(2-fluorobenzyl)propanamide **(*S*)-8** ESI+

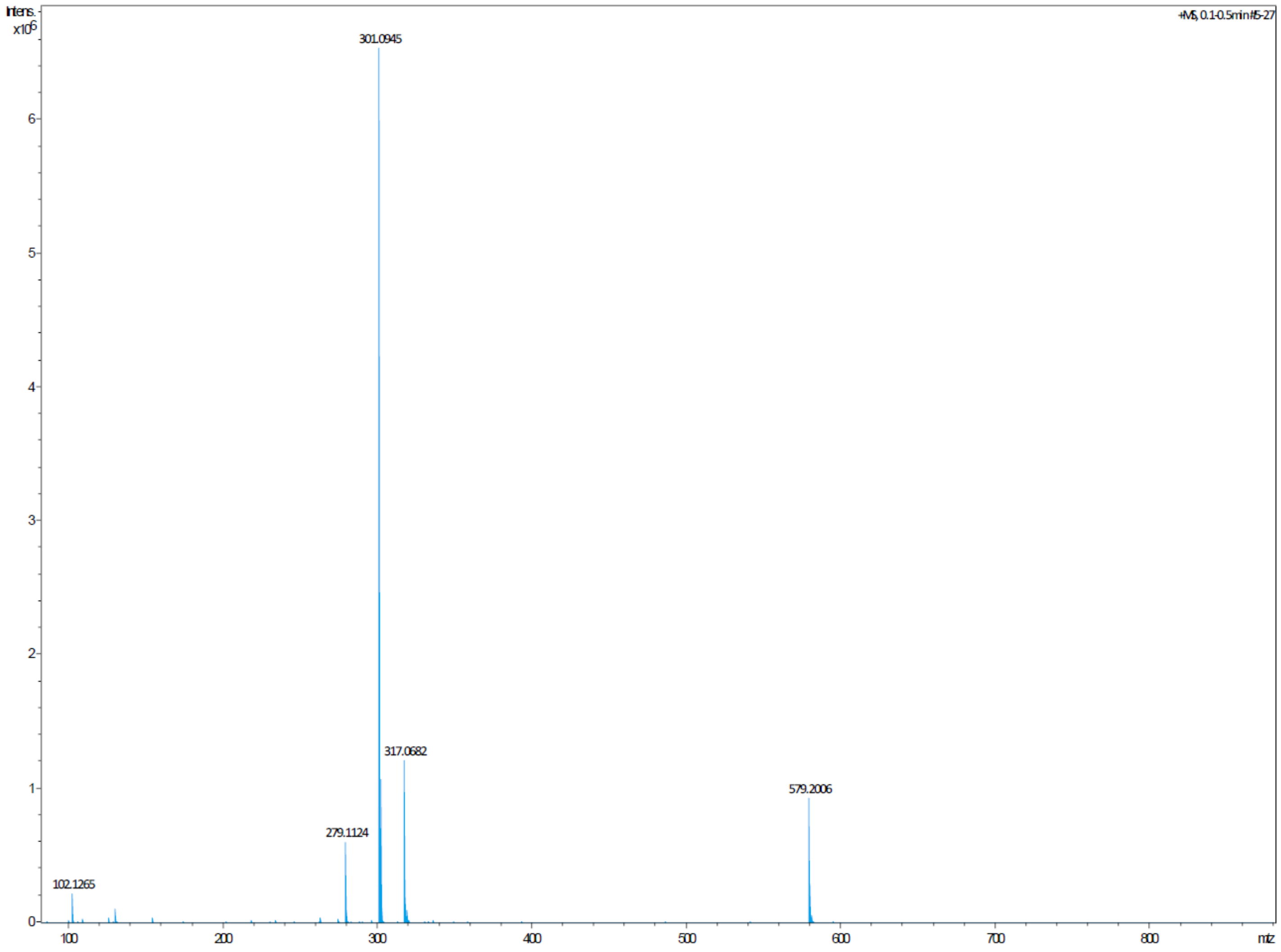

(*S*)-2-(2,5-dioxopyrrolidin-1-yl)-*N*-(2-fluorobenzyl)propanamide **(*S*)-8** ESI-

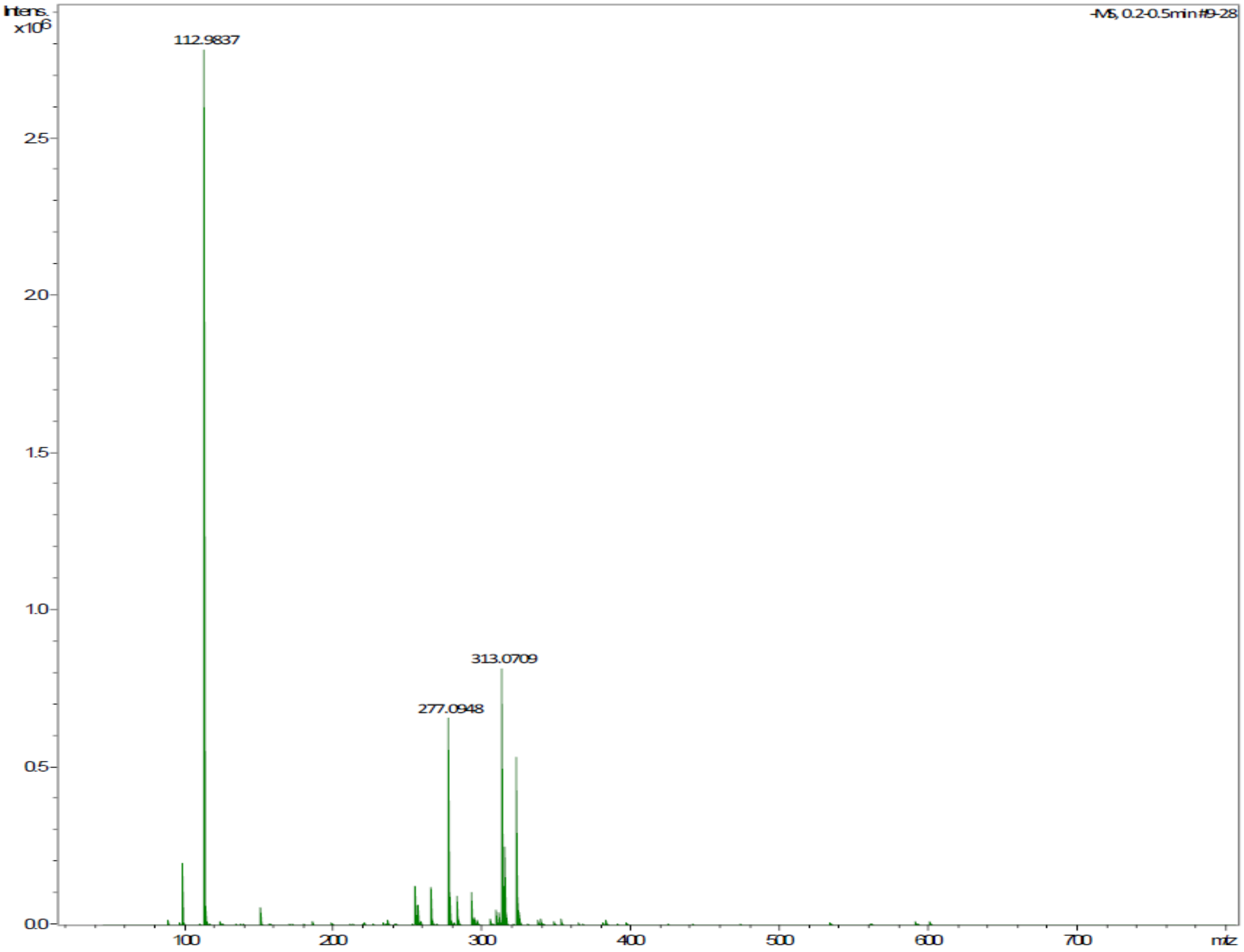

^{1}H NMR, ^{13}C NMR spectra for intermediates and final compounds

(*R*)-*tert*-butyl-(1-(benzylamino)-1-oxopropan-2-yl)carbamate **(*R*)-1** ^{1}HNMR

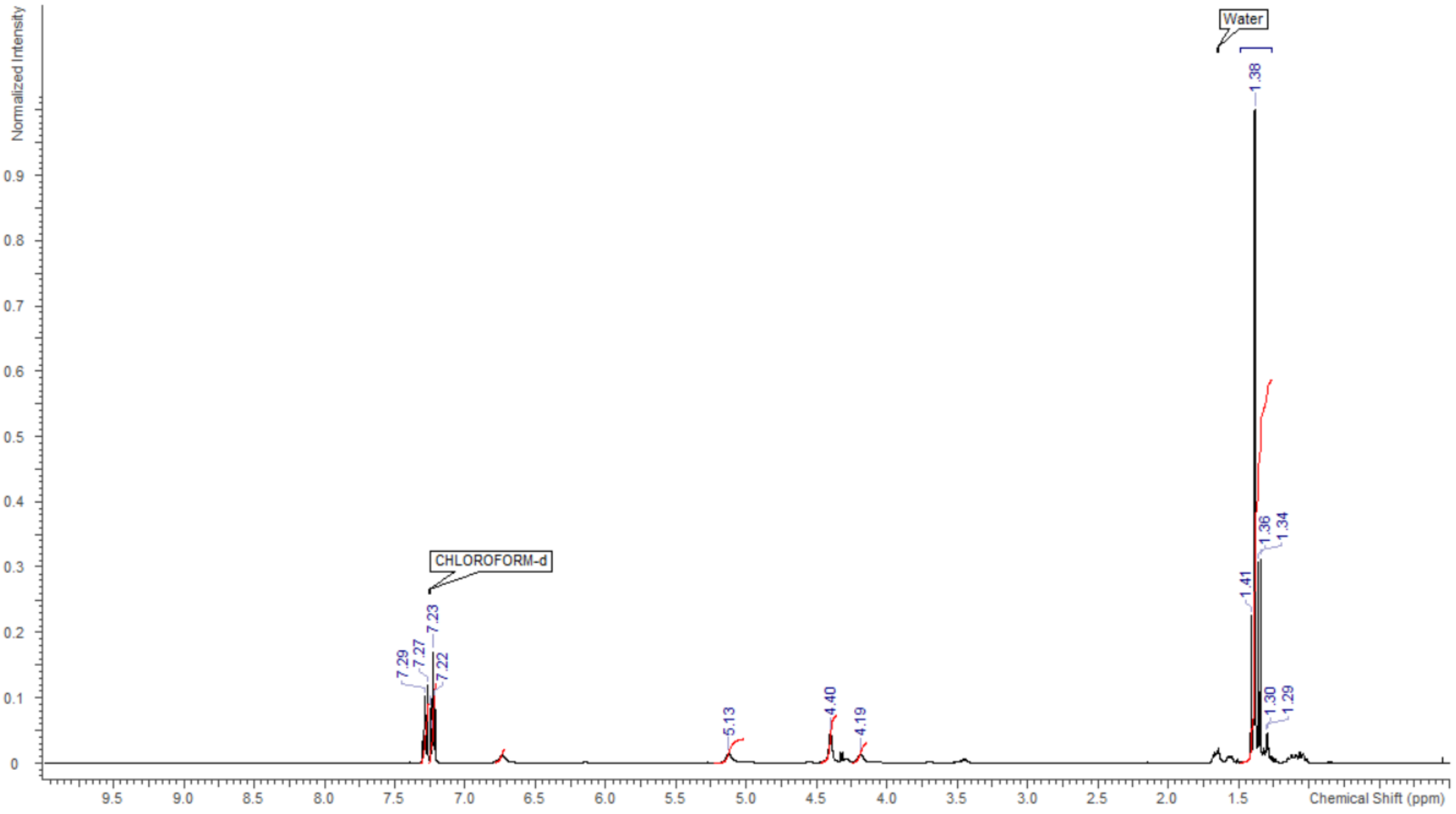

(*R*)-*tert*-butyl-(1-(benzylamino)-1-oxopropan-2-yl)carbamate ***(R)*-1** ^{13}CNMR

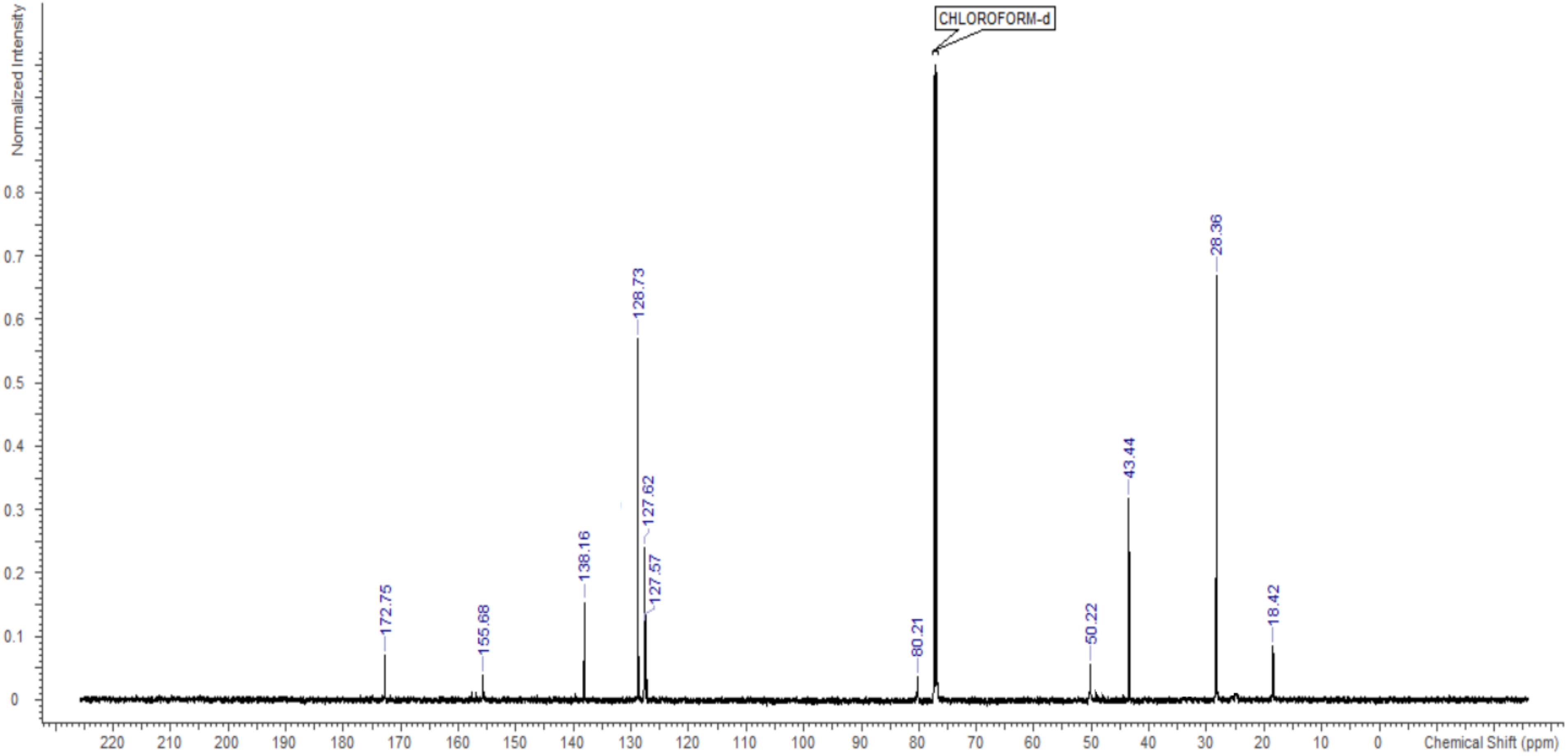

(*R*)-tert-butyl-(1-((2-fluorobenzyl)amino)-1-oxopropan-2-yl)carbamate **(*R*)-2** ^{1}HNMR

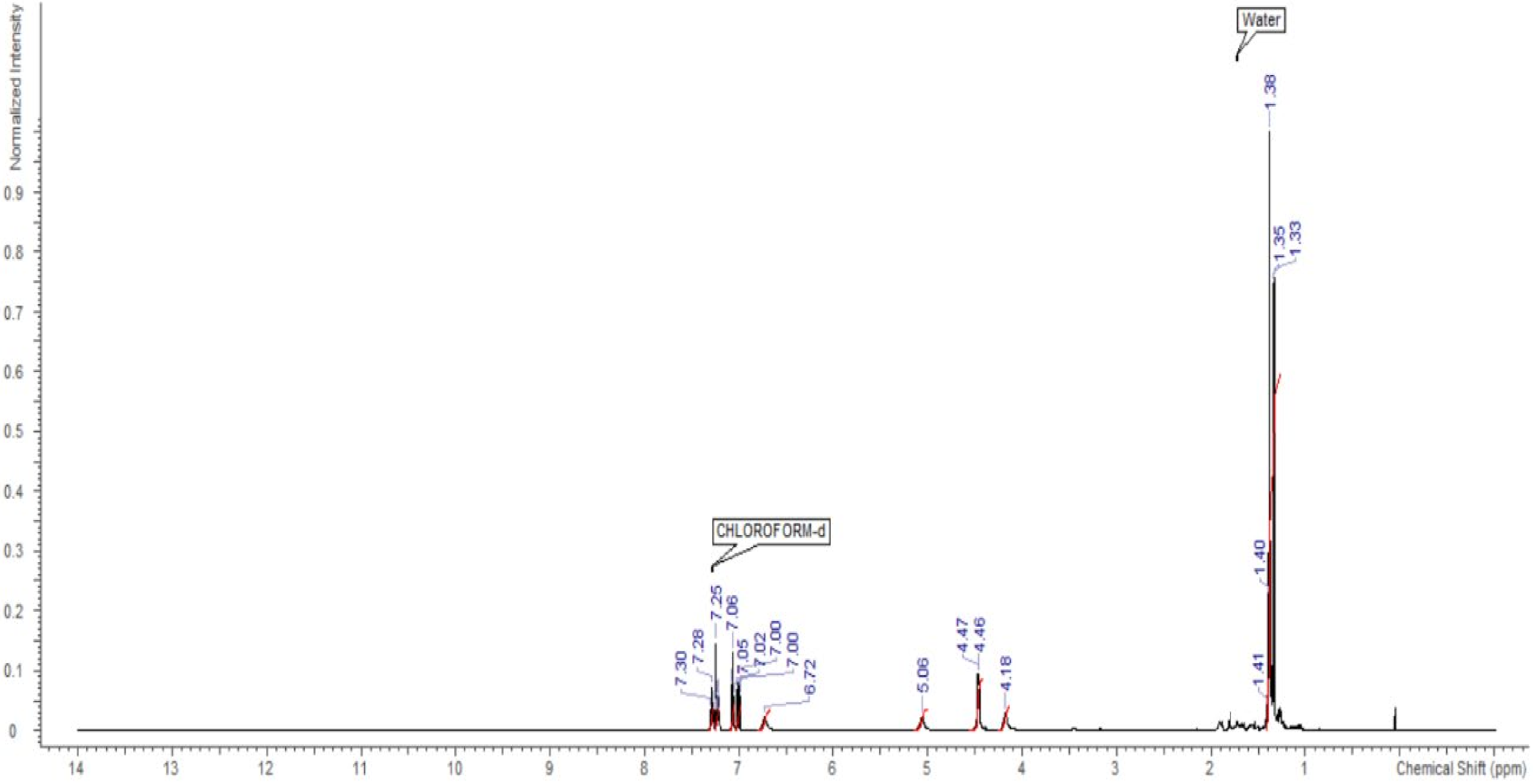

(*R*)-tert-butyl-(1-((2-fluorobenzyl)amino)-1-oxopropan-2-yl)carbamate ***(R)*-2** ^{13}CNMR

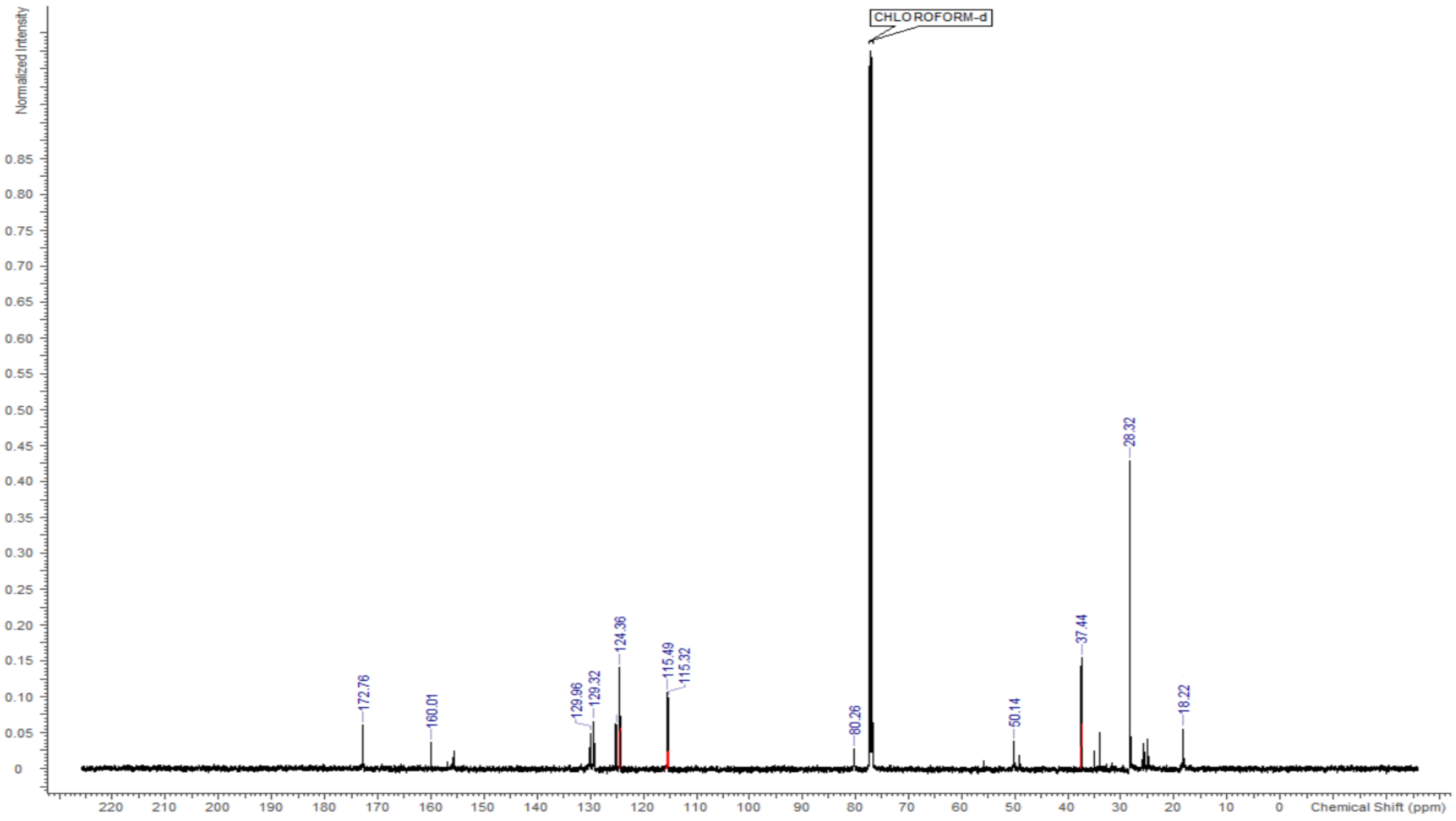

(*S*)-*tert*-butyl-(1-(benzylamino)-1-oxopropan-2-yl)carbamate **(*S*)-1** ^{1}HNMR

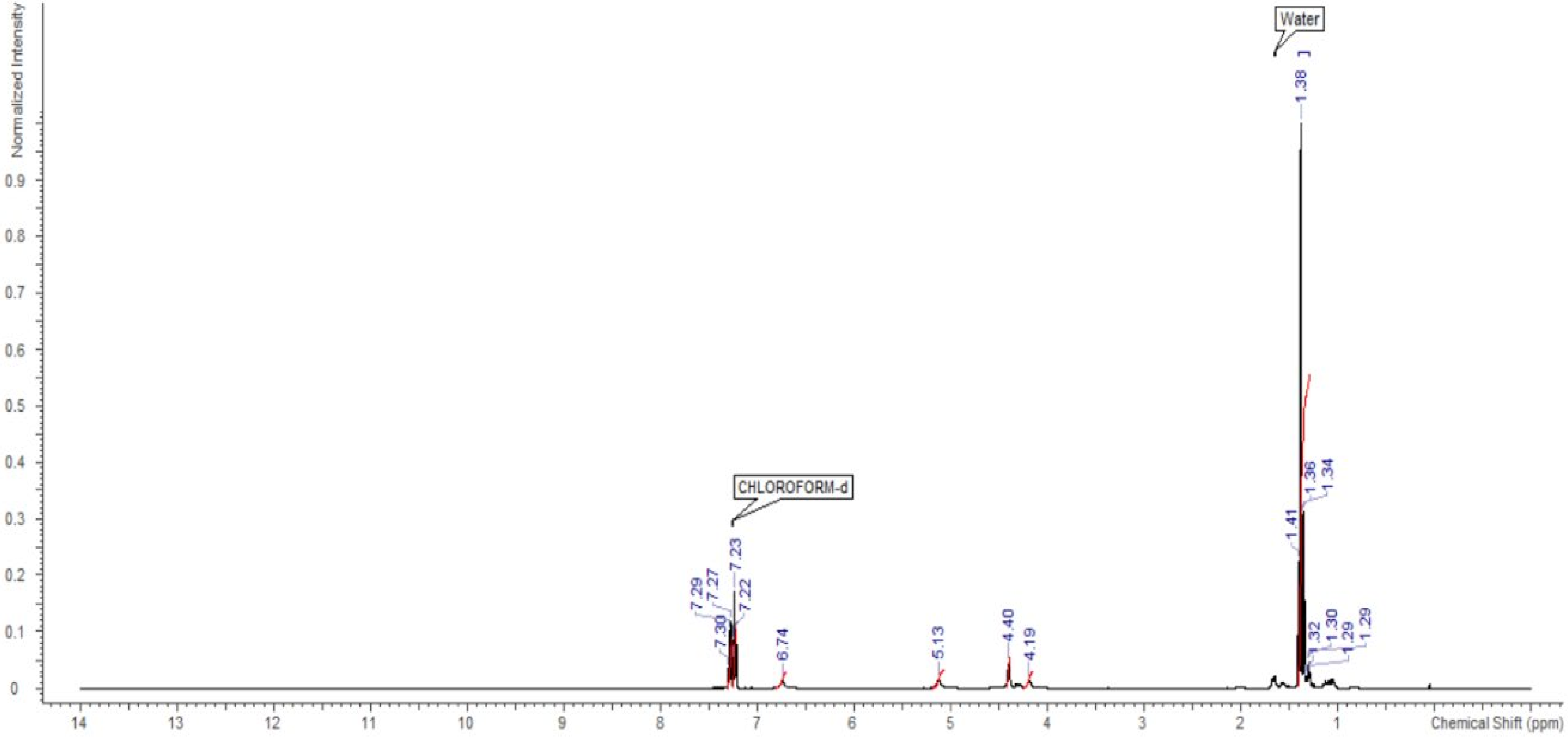

(*S*)-*tert*-butyl-(1-(benzylamino)-1-oxopropan-2-yl)carbamate **(*S*)-1** ^{13}CNMR

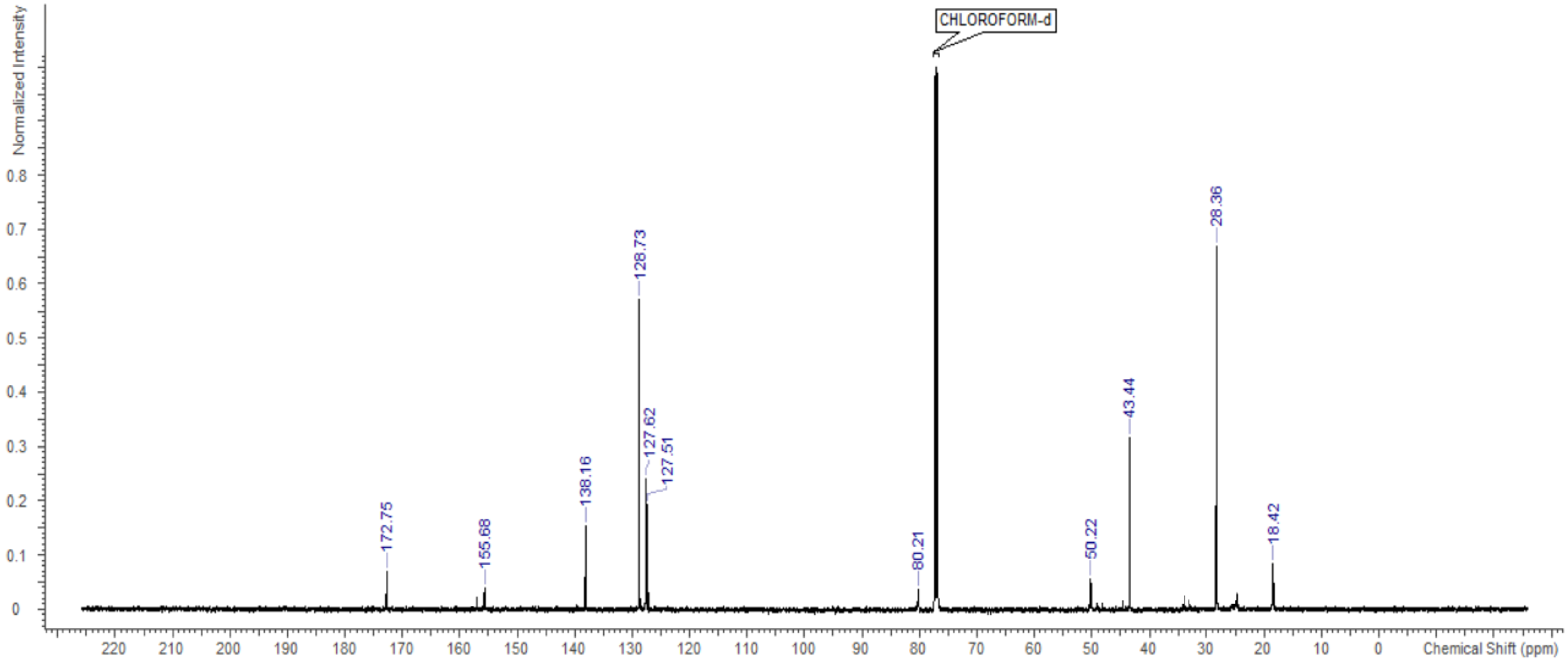

(*S*)-tert-butyl-(1-((2-fluorobenzyl)amino)-1-oxopropan-2-yl)carbamate **(*S*)-2** ^{1}HNMR

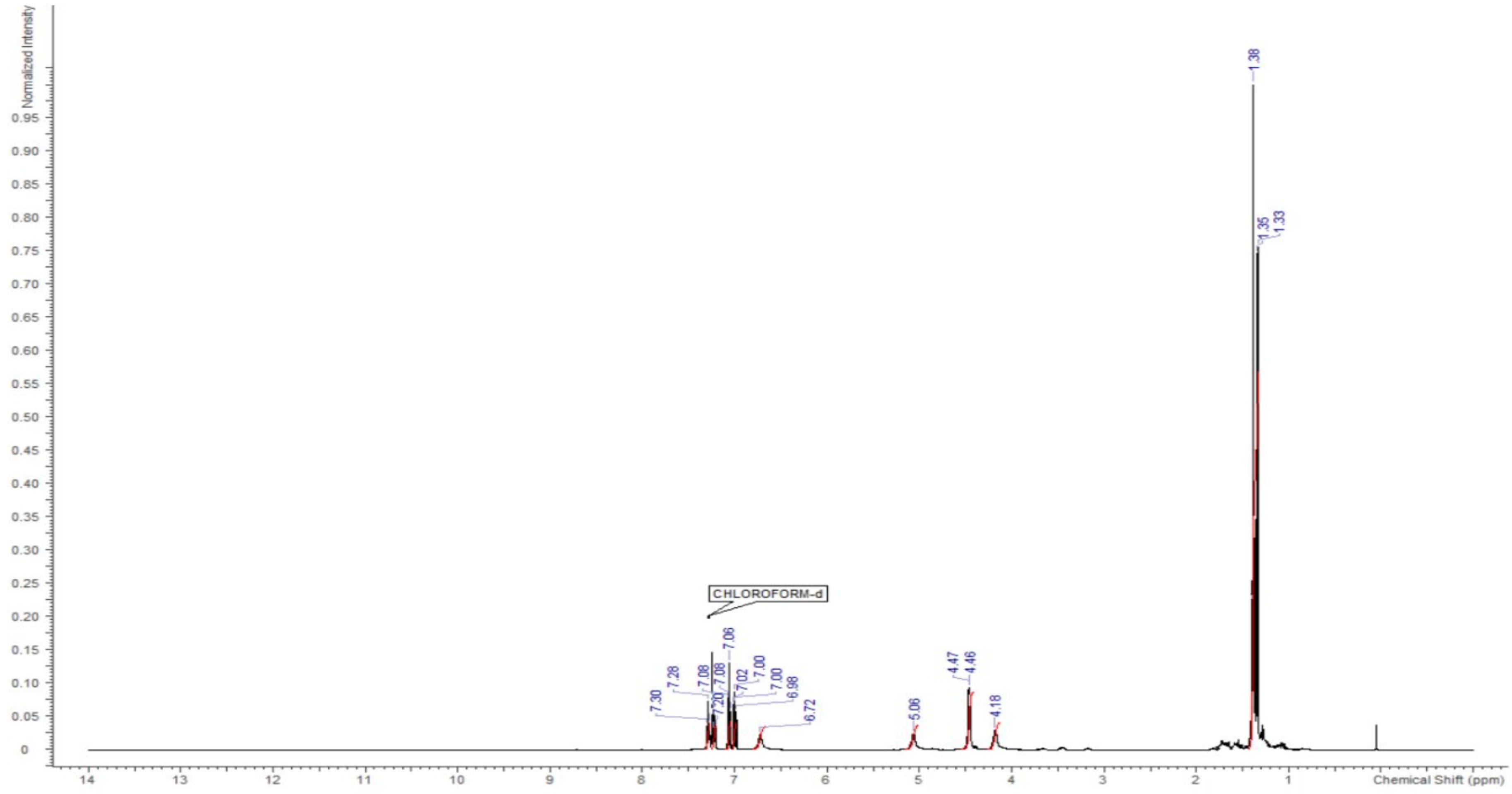

(*S*)-tert-butyl-(1-((2-fluorobenzyl)amino)-1-oxopropan-2-yl)carbamate **(*S*)-2** ^{13}CNMR

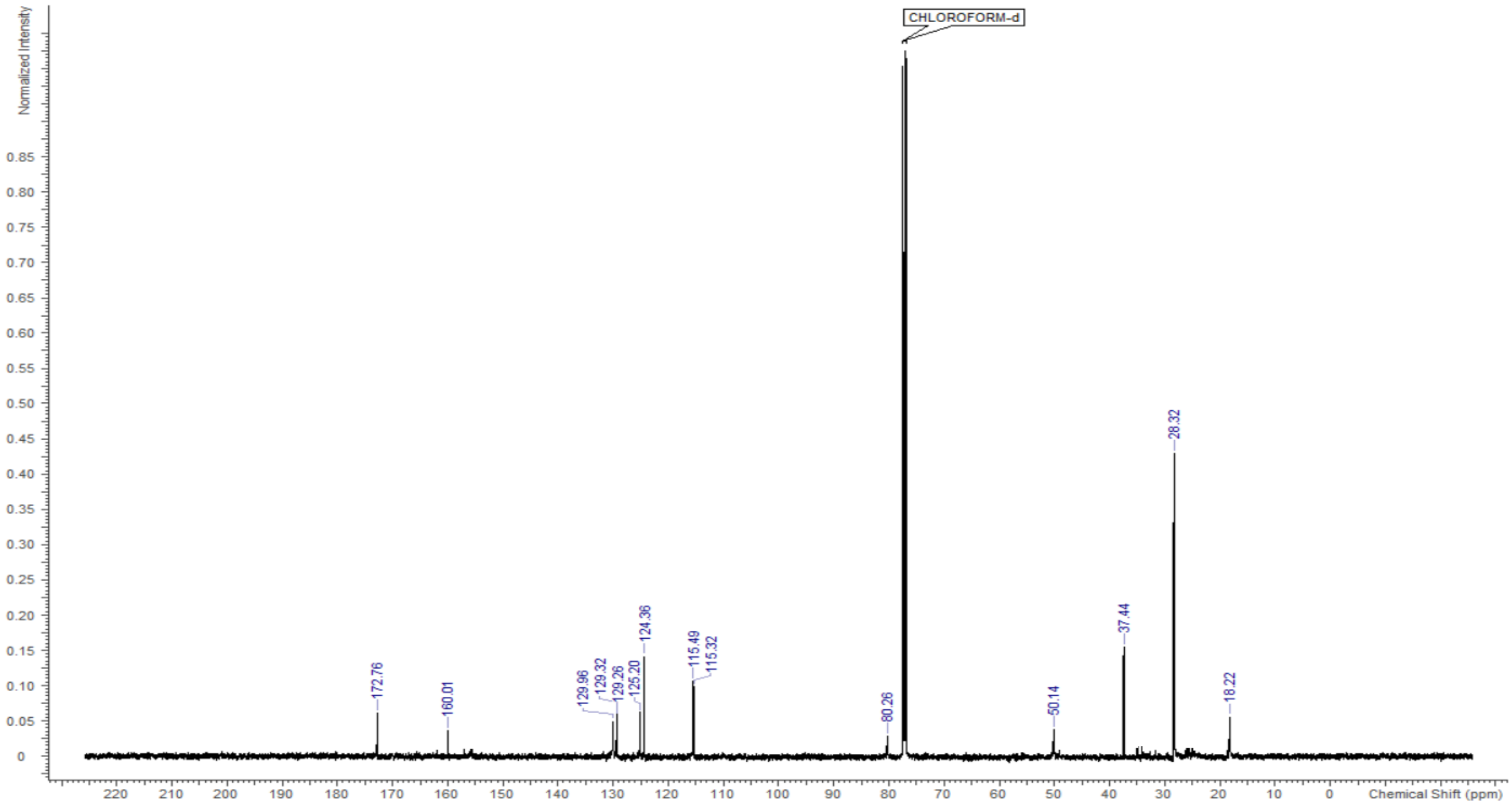

(R)-2-amino-*N*-benzylpropanamide **(R)-3** ^{1}HNMR

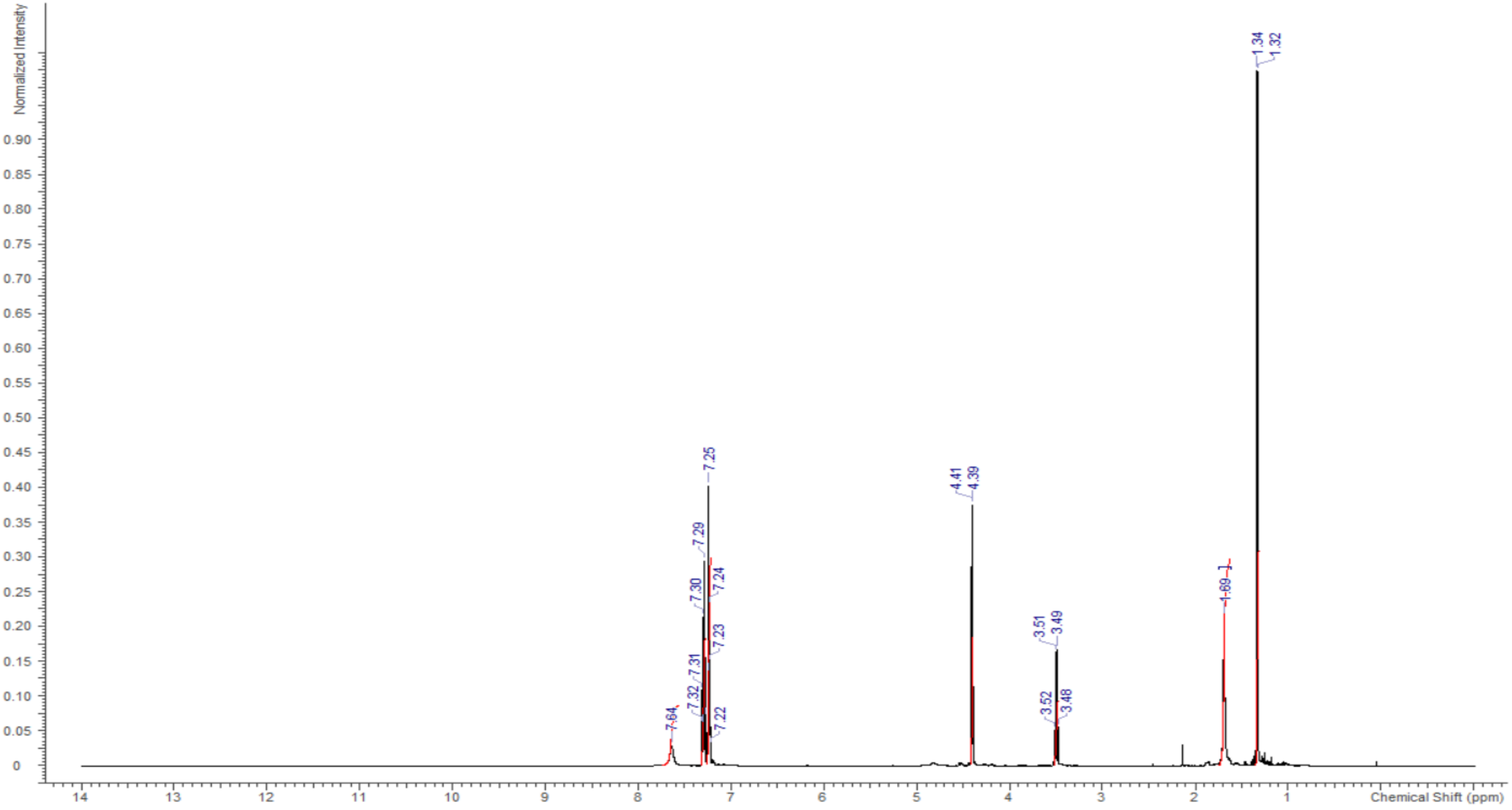

(*R*)-2-amino-*N*-benzylpropanamide ***(R)*-3** ^{13}CNMR

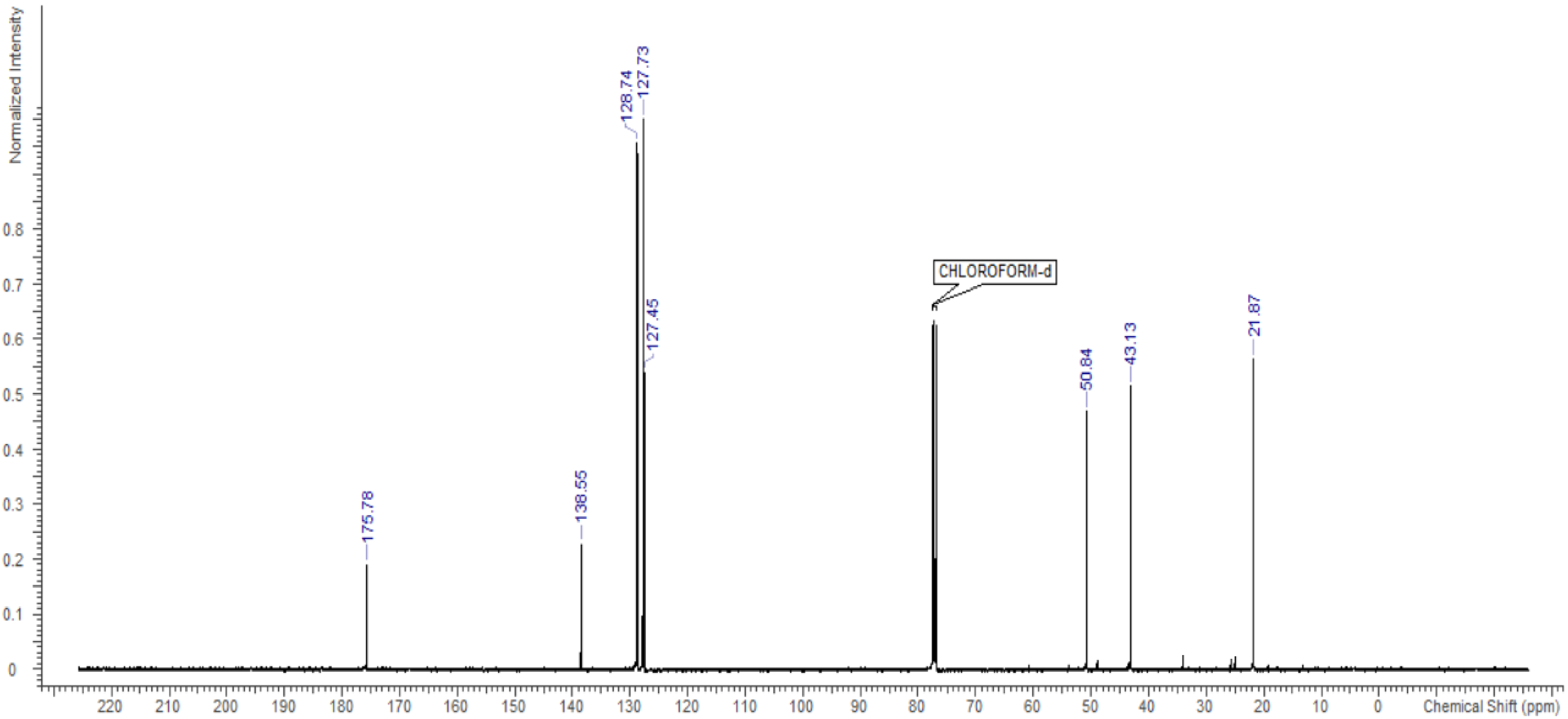

(*R*)-2-amino-*N*-(2-fluorobenzyl)propanamide **(*R*)-4** ^{1}HNMR

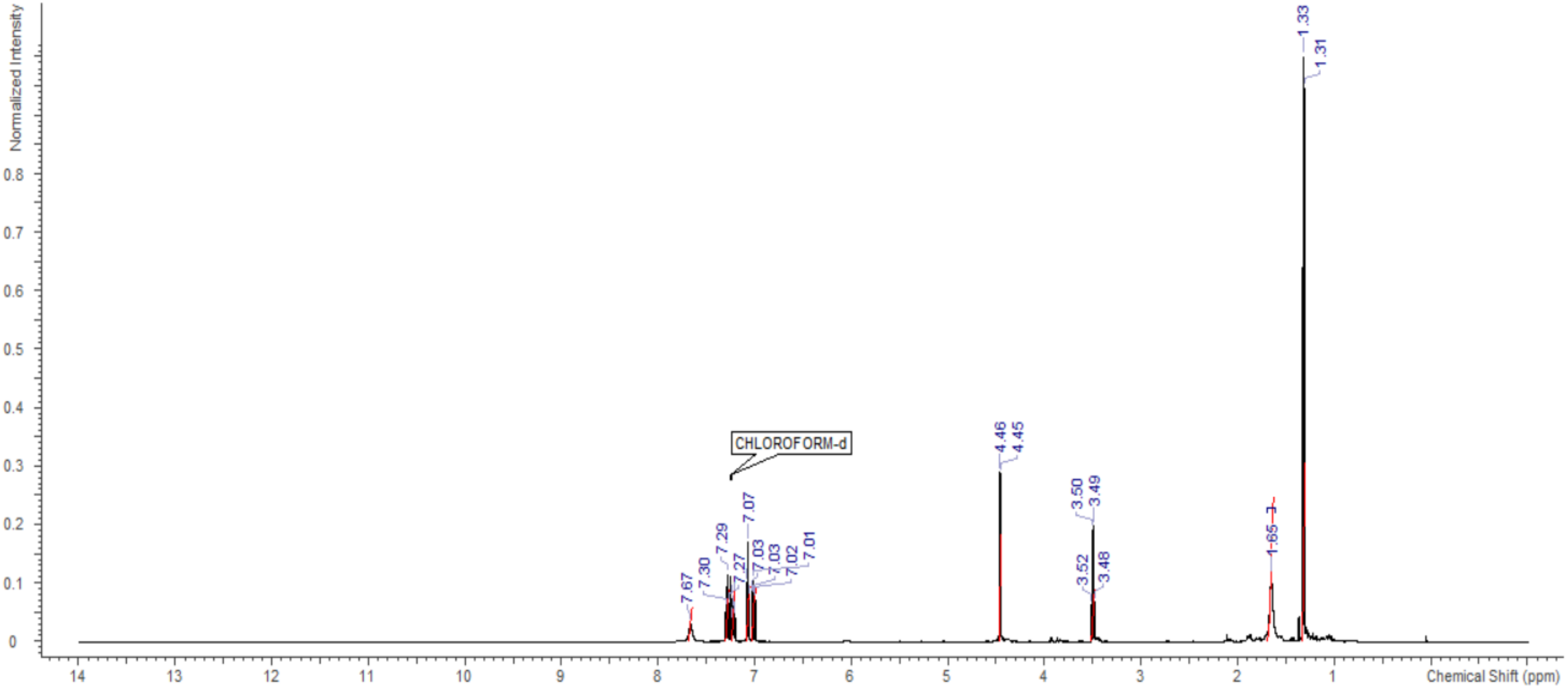

(*R*)-2-amino-*N*-(2-fluorobenzyl)propanamide **(*R*)-4** ^{13}CNMR

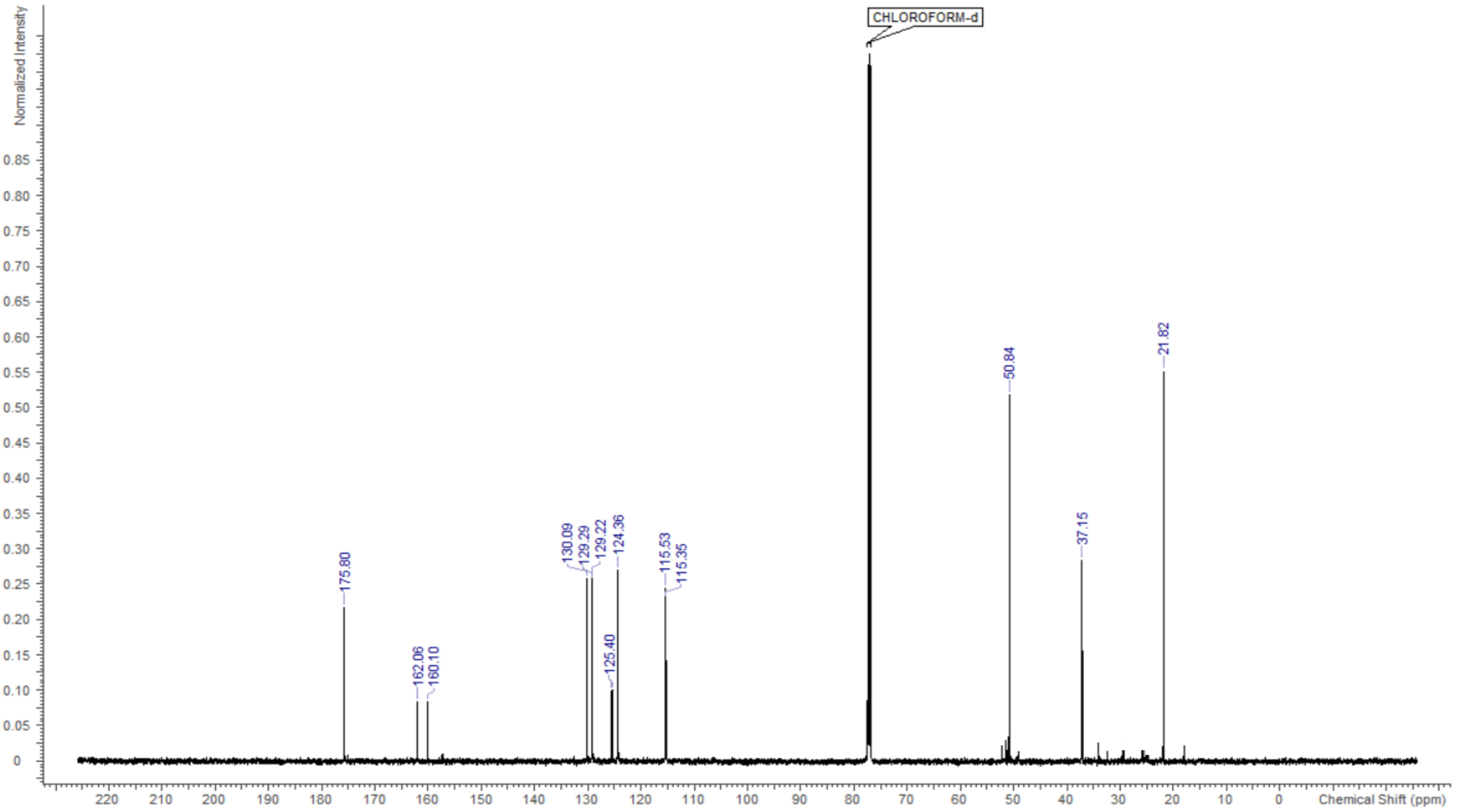

(*S*)-2-amino-*N*-benzylpropanamide **(*S*)-3** ^{1}HNMR

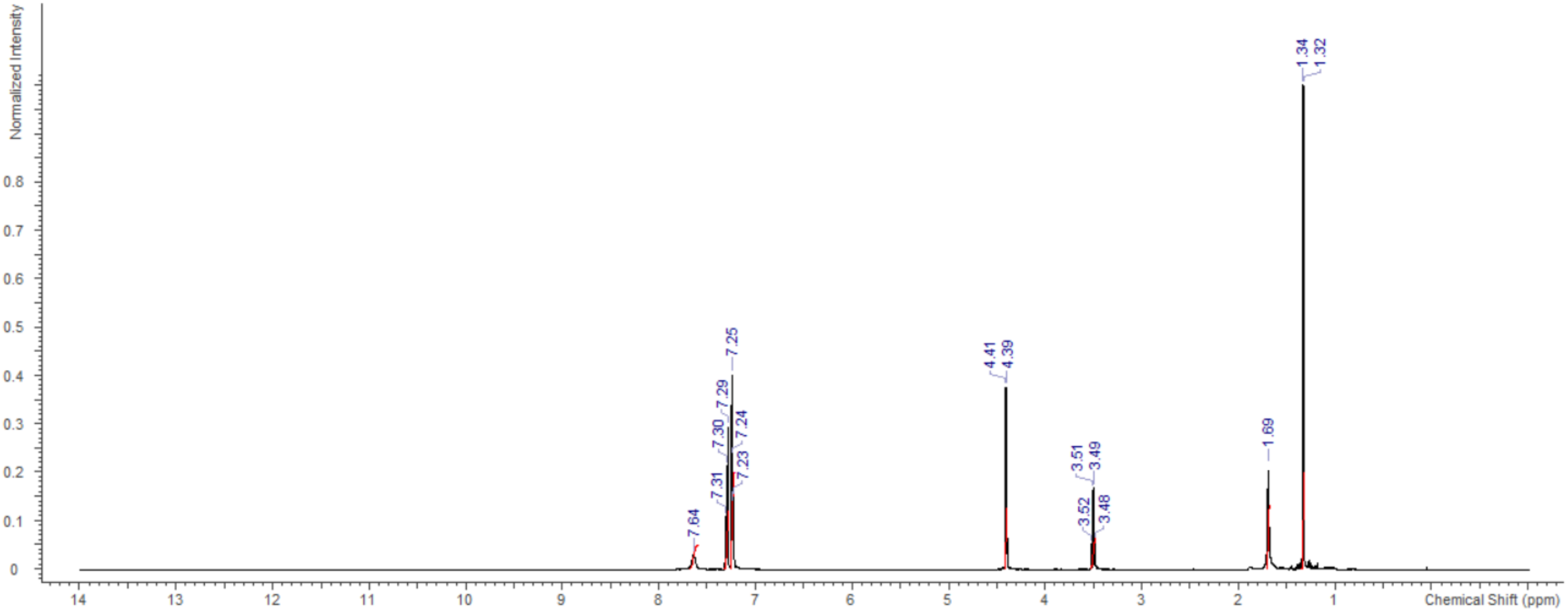

(*S*)-2-amino-*N*-benzylpropanamide **(*S*)-3** ^{13}CNMR

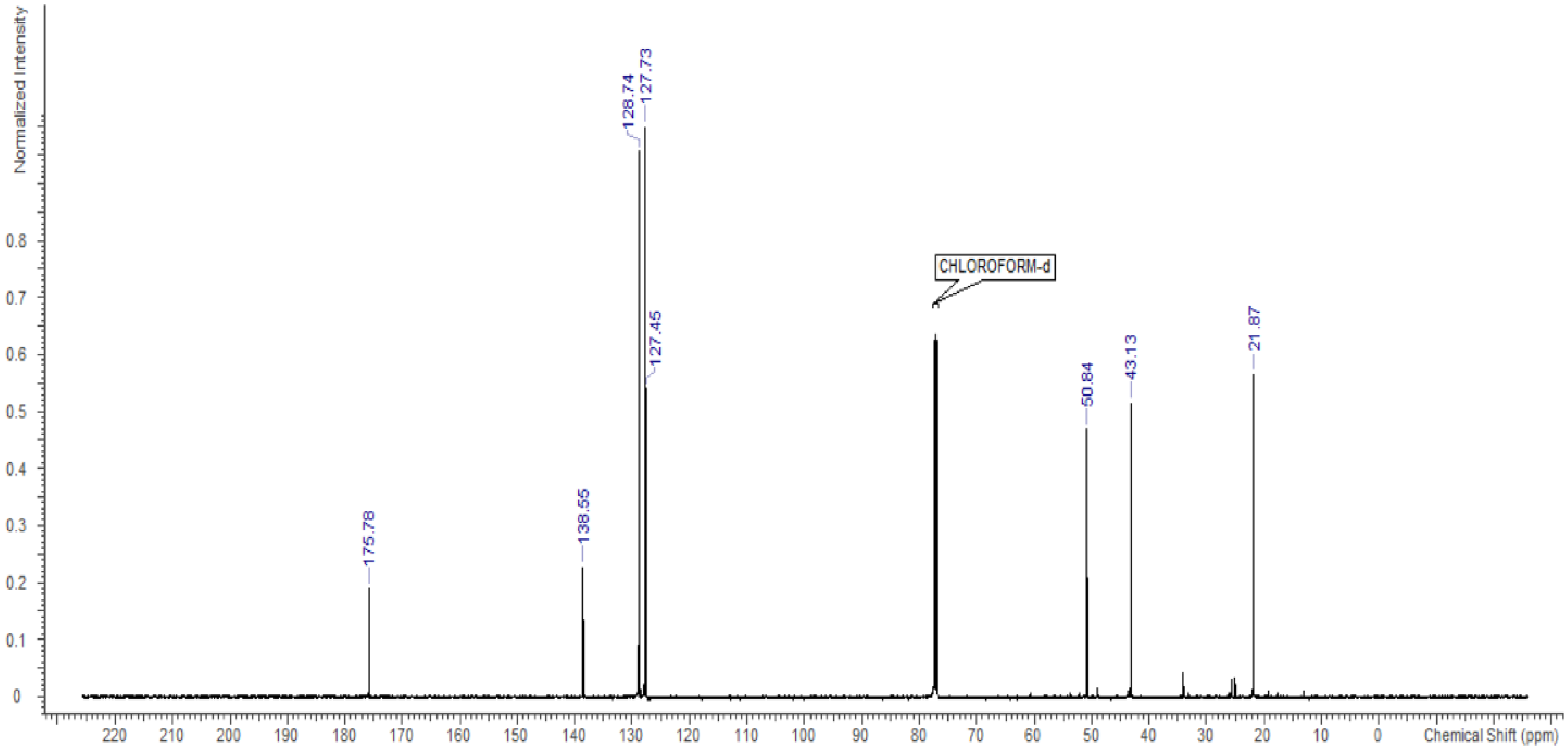

(*S*)-2-amino-*N*-(2-fluorobenzyl)propanamide **(*S*)-4** ^{1}HNMR

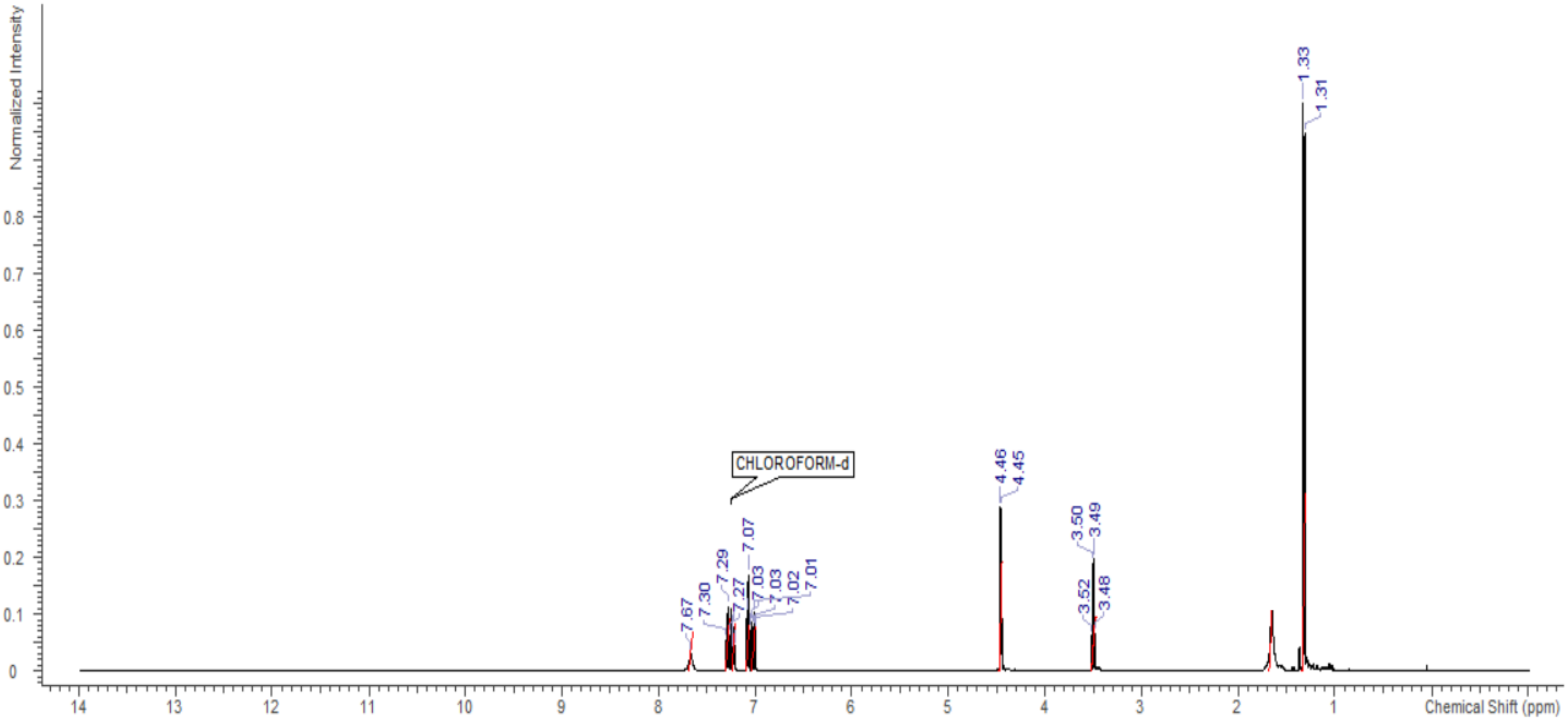

(*S*)-2-amino-*N*-(2-fluorobenzyl)propanamide **(*S*)-4** ^{13}CNMR

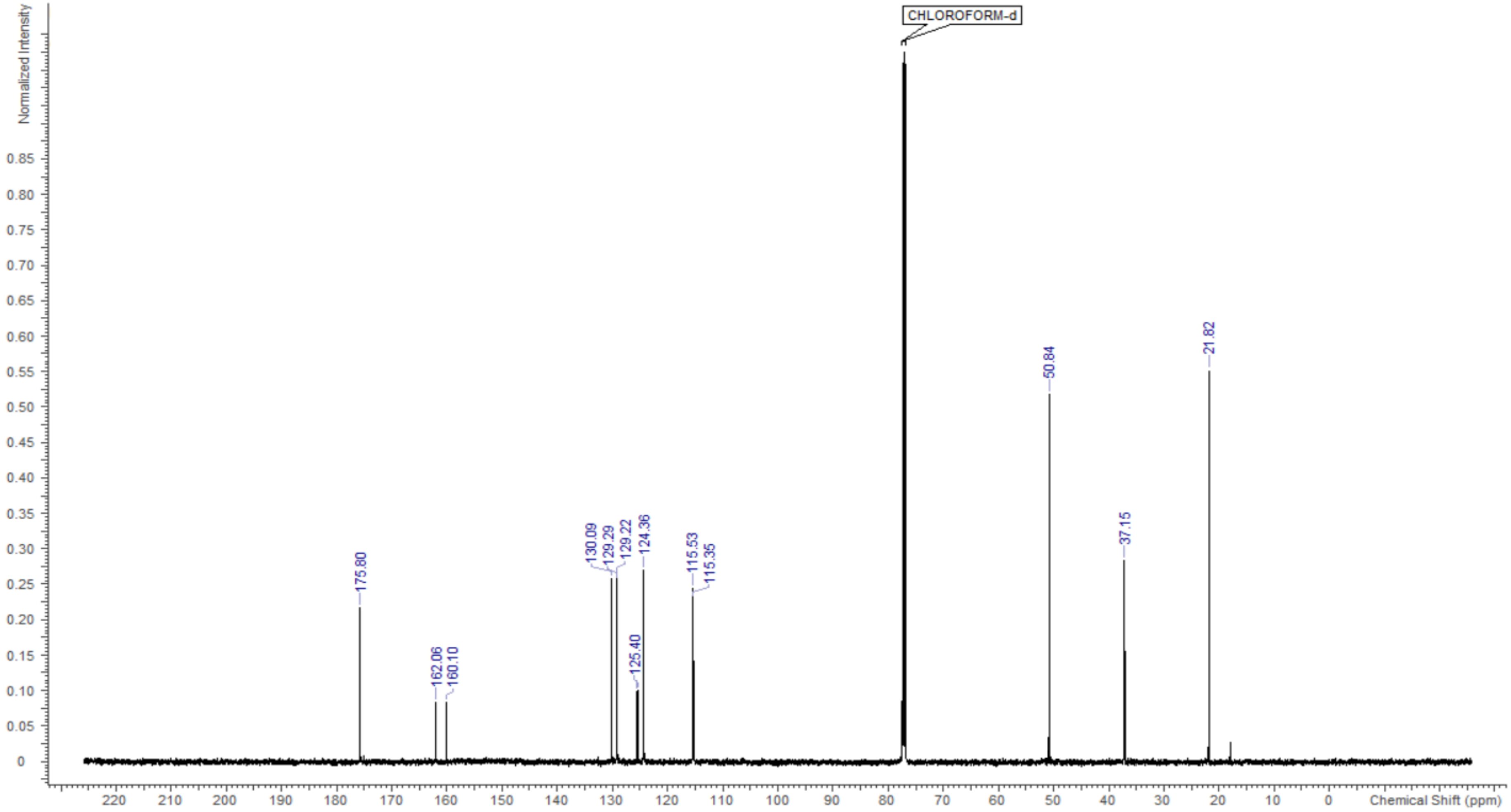

(*R*)-4-((1-(benzylamino)-1-oxopropan-2-yl)amino)-4-oxobutanoic acid **(*R*)-5** ^{1}HNMR

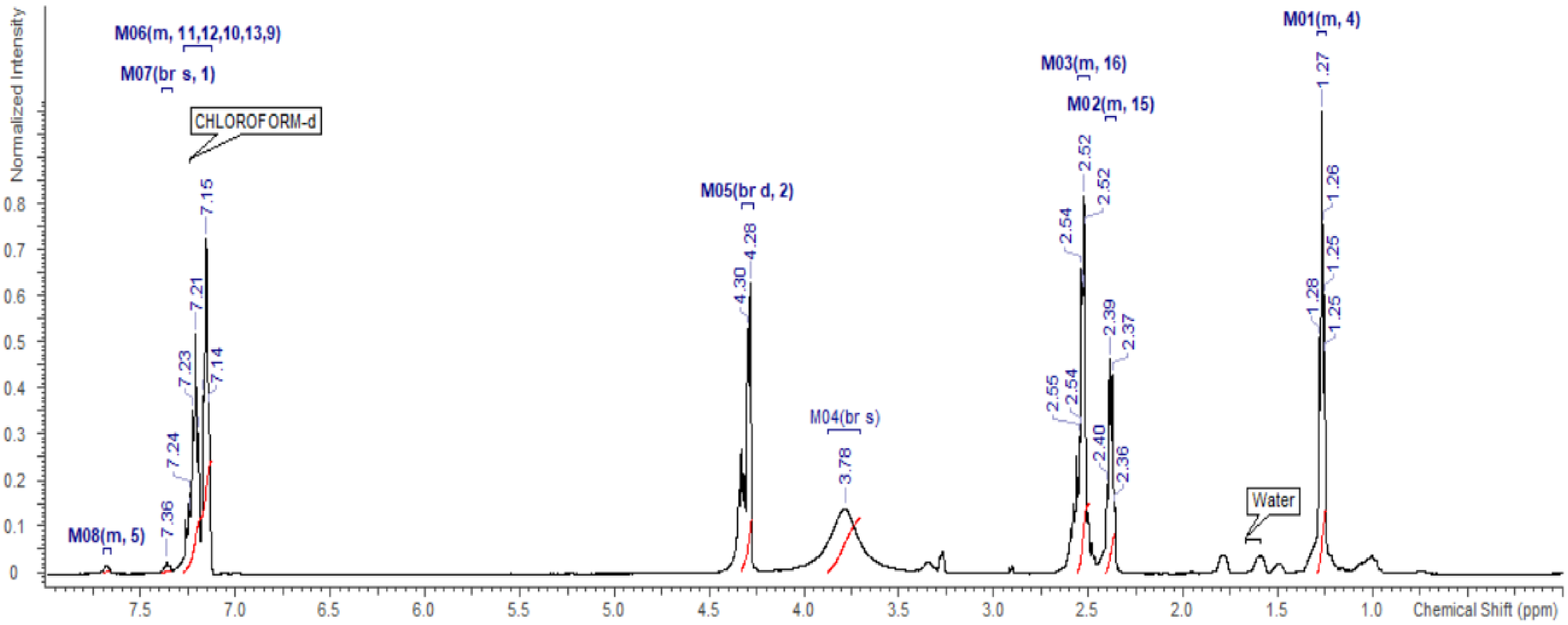

(*R*)-4-((1-(benzylamino)-1-oxopropan-2-yl)amino)-4-oxobutanoic acid ***(R)*-5** ^{13}CNMR

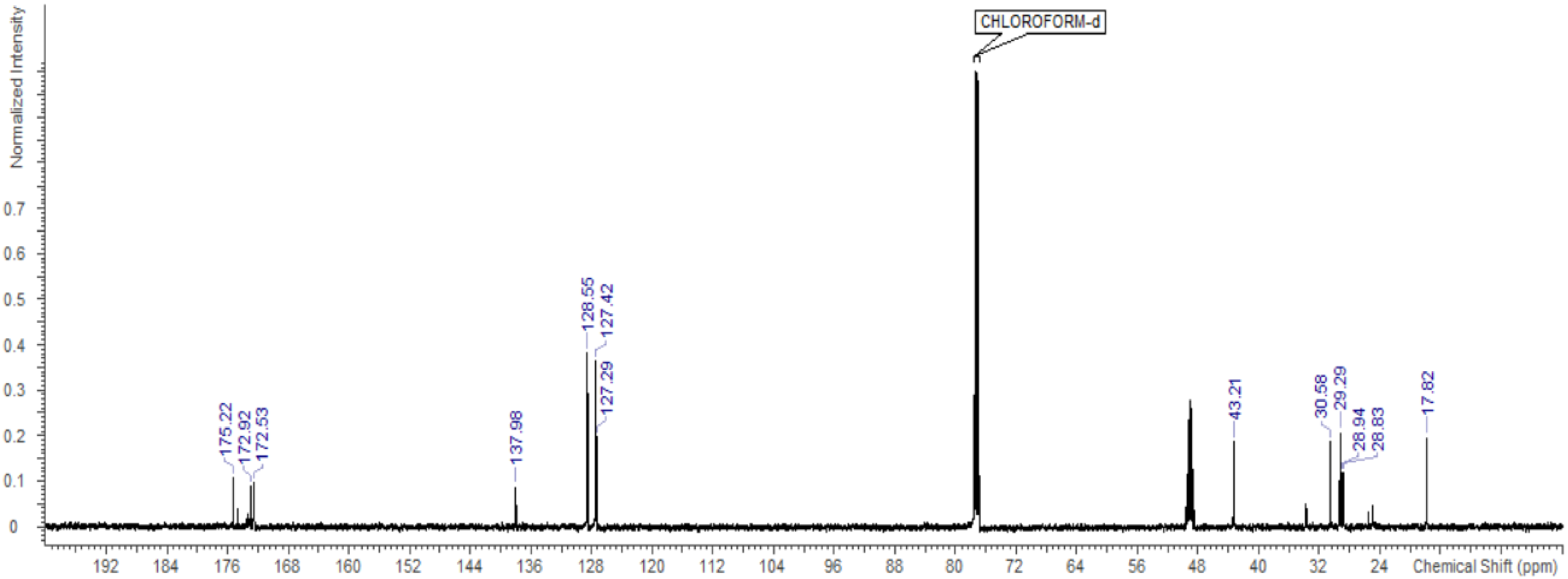

(*R*)-4-((1-((2-fluorobenzyl)amino)-1-oxopropan-2-yl)amino)-4-oxobutanoic acid ***(R)*-6** ^{1}HNMR

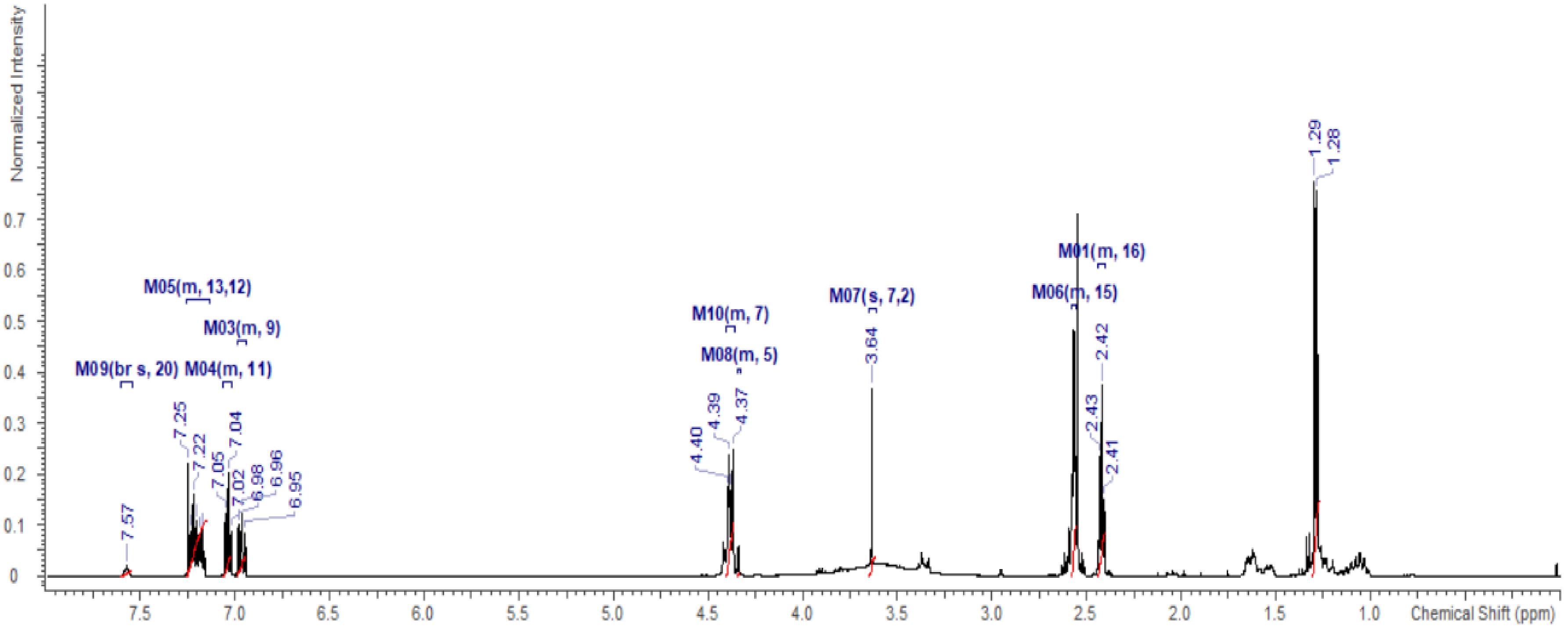

(*R*)-4-((1-((2-fluorobenzyl)amino)-1-oxopropan-2-yl)amino)-4-oxobutanoic acid ***(R)*-6** ^{13}CNMR

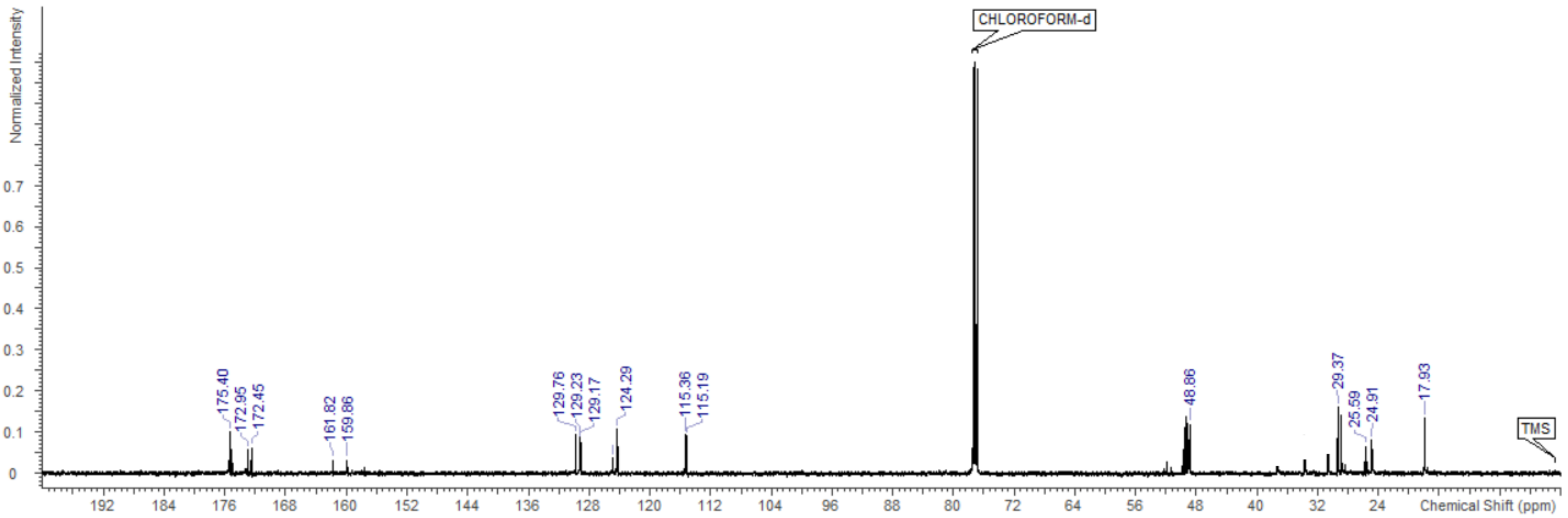

(*S*)-4-((1-(benzylamino)-1-oxopropan-2-yl)amino)-4-oxobutanoic acid **(*S*)-5** ^{1}HNMR

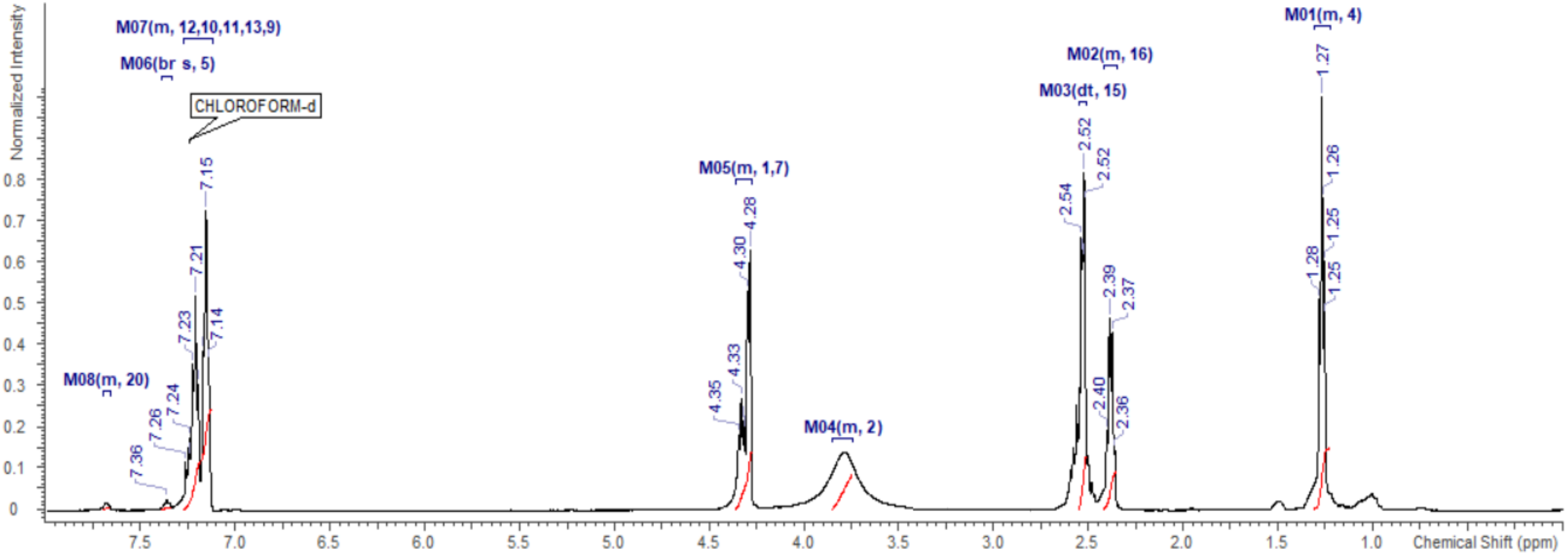

(*S*)-4-((1-(benzylamino)-1-oxopropan-2-yl)amino)-4-oxobutanoic acid **(*S*)-5** 13CNMR

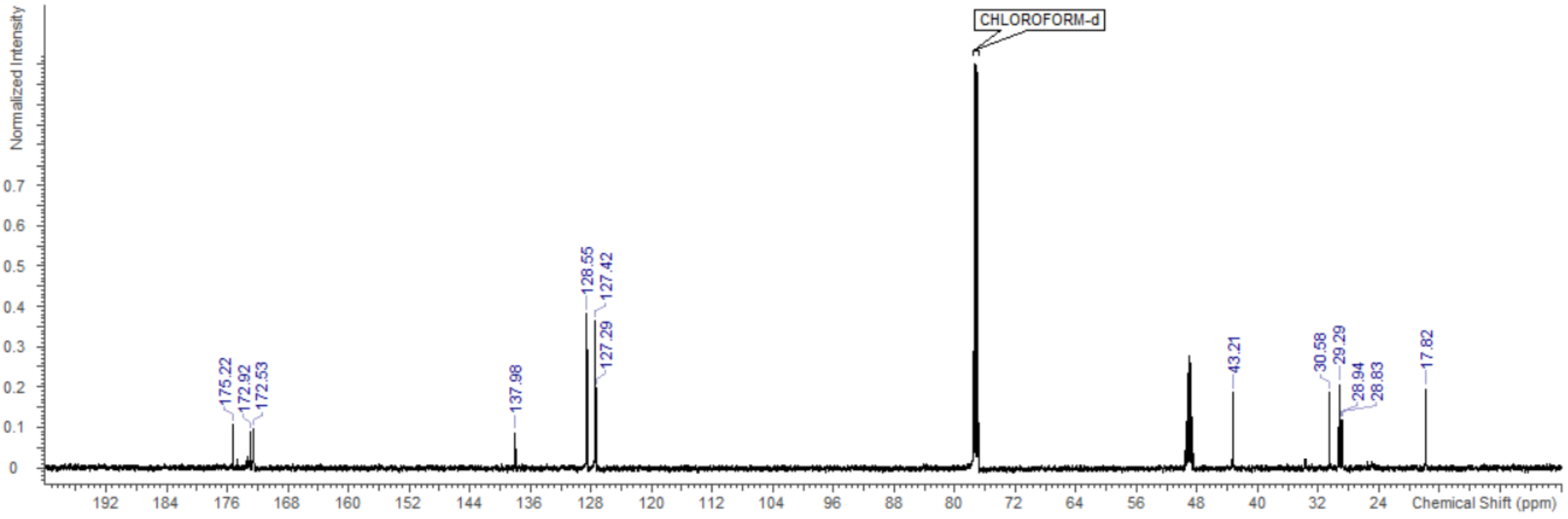

(*S*)-4-((1-((2-fluorobenzyl)amino)-1-oxopropan-2-yl)amino)-4-oxobutanoic acid **(*S*)-6** ^{1}HNMR

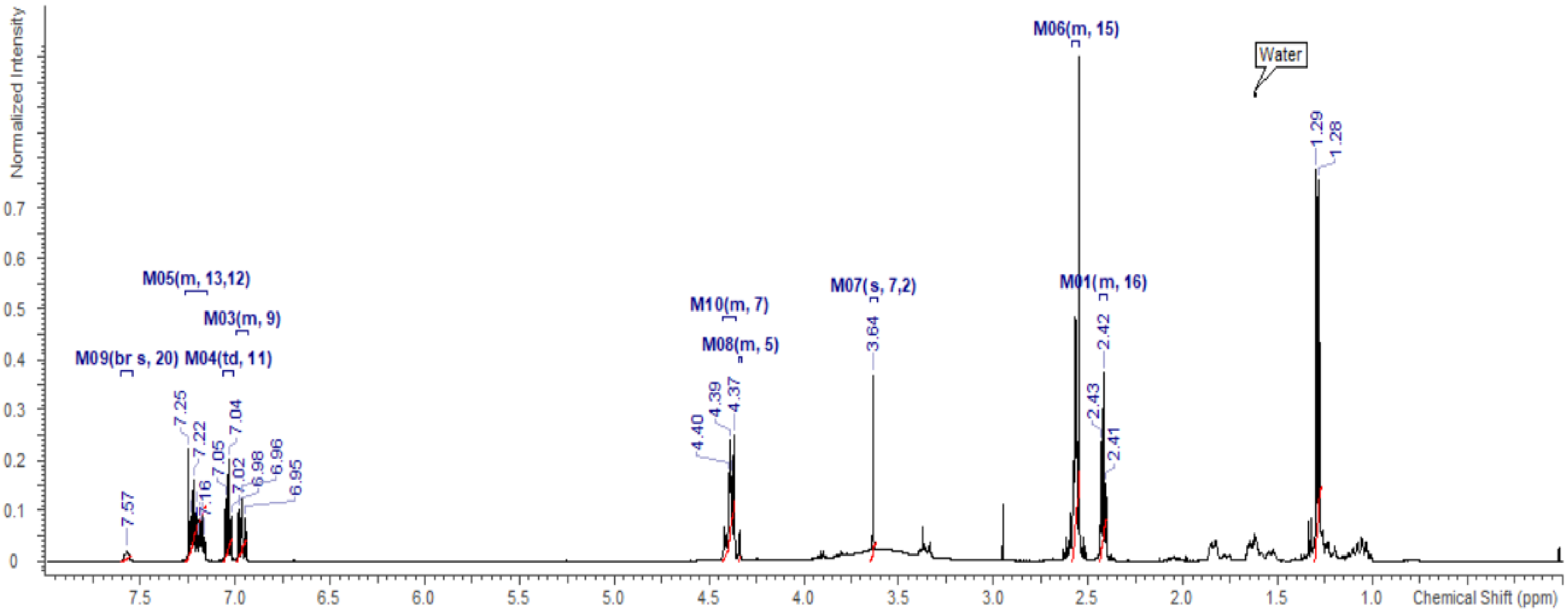

(*S*)-4-((1-((2-fluorobenzyl)amino)-1-oxopropan-2-yl)amino)-4-oxobutanoic acid ***(S)*-6** ^{13}CNMR

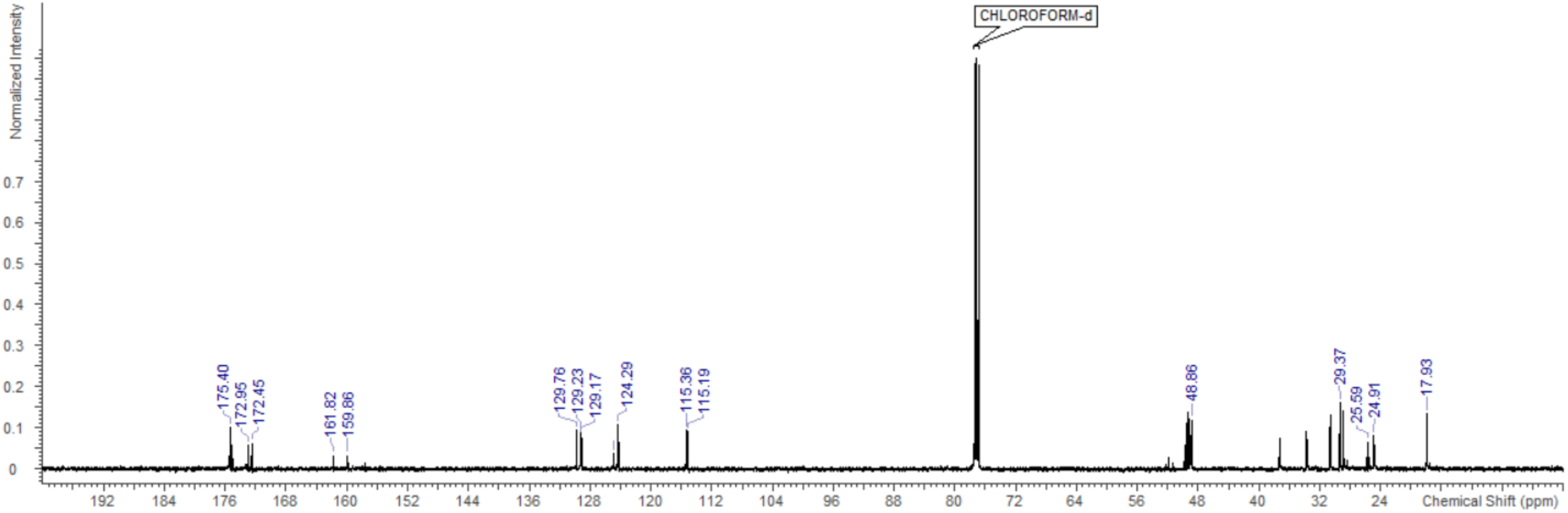

(R)-N-benzyl-2-(2,5-dioxopyrrolidin-1-yl)propanamide **(*R*)-7** ^{1}HNMR

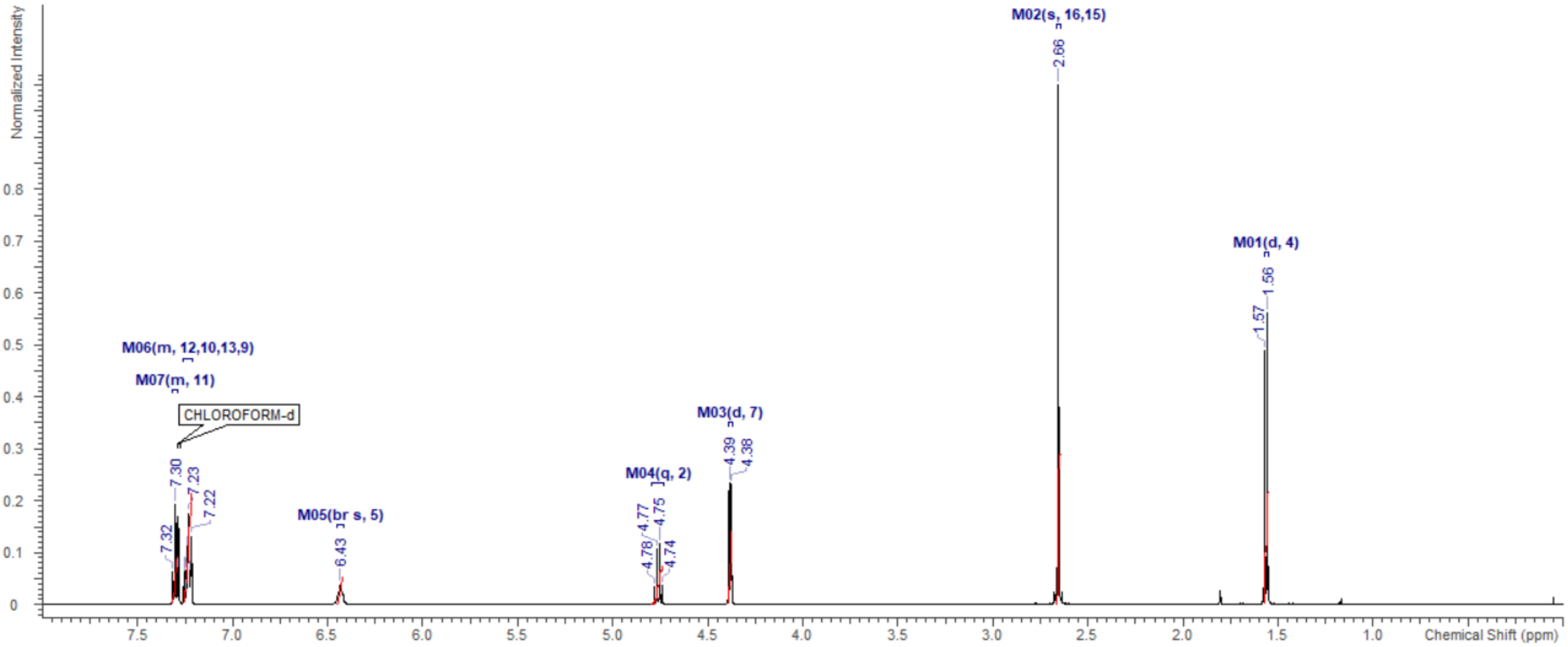

(*R*)-*N*-benzyl-2-(2,5-dioxopyrrolidin-1-yl)propanamide ***(R)*-7** ^{13}CNMR

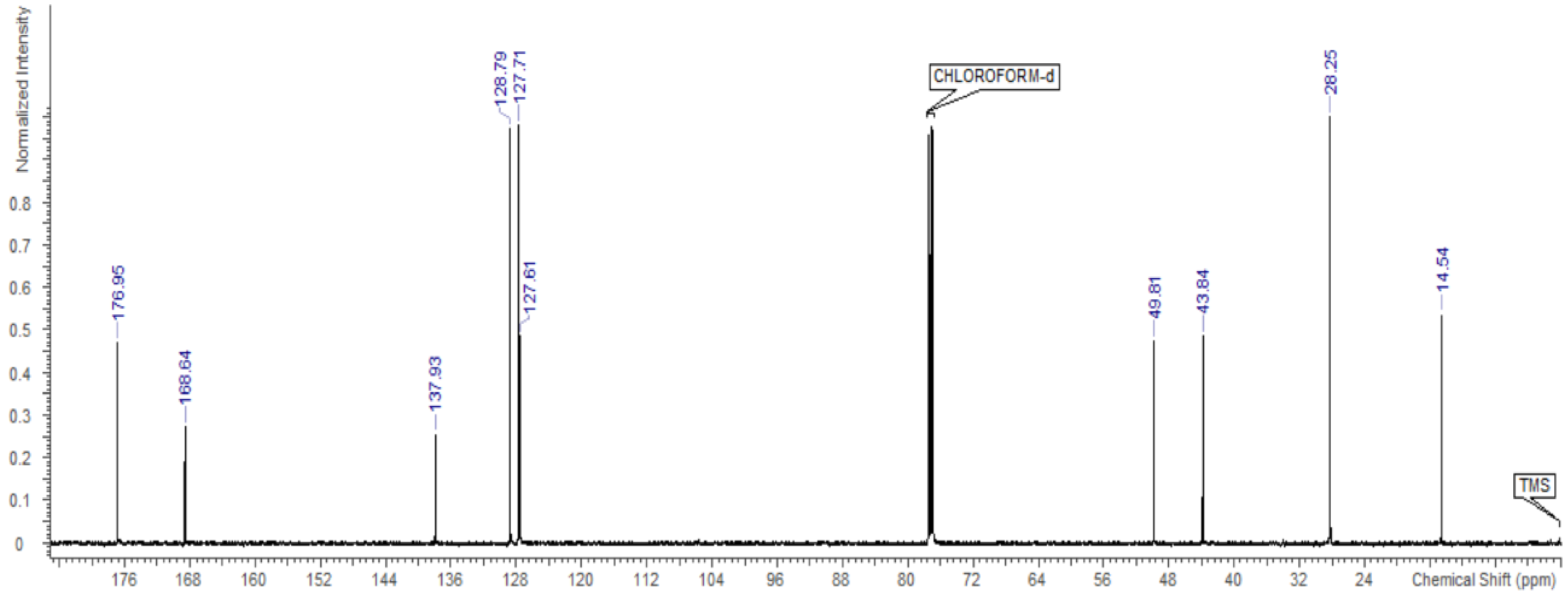

(*R*)-2-(2,5-dioxopyrrolidin-1-yl)-*N*-(2-fluorobenzyl)propanamide ***(R*)-8** ^{1}HNMR

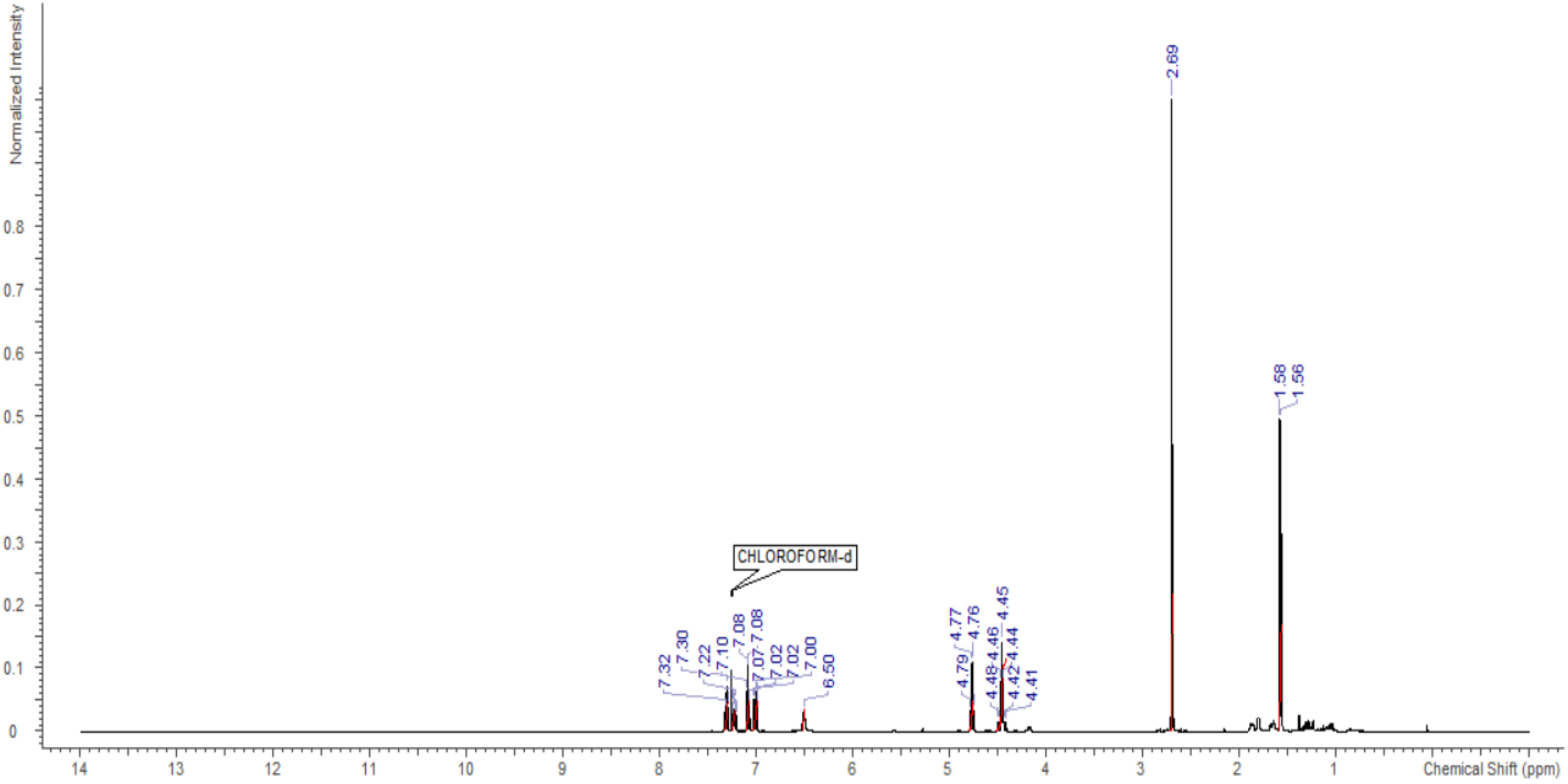

(*R*)-2-(2,5-dioxopyrrolidin-1-yl)-*N*-(2-fluorobenzyl)propanamide ***(R)*-8** ^{13}CNMR

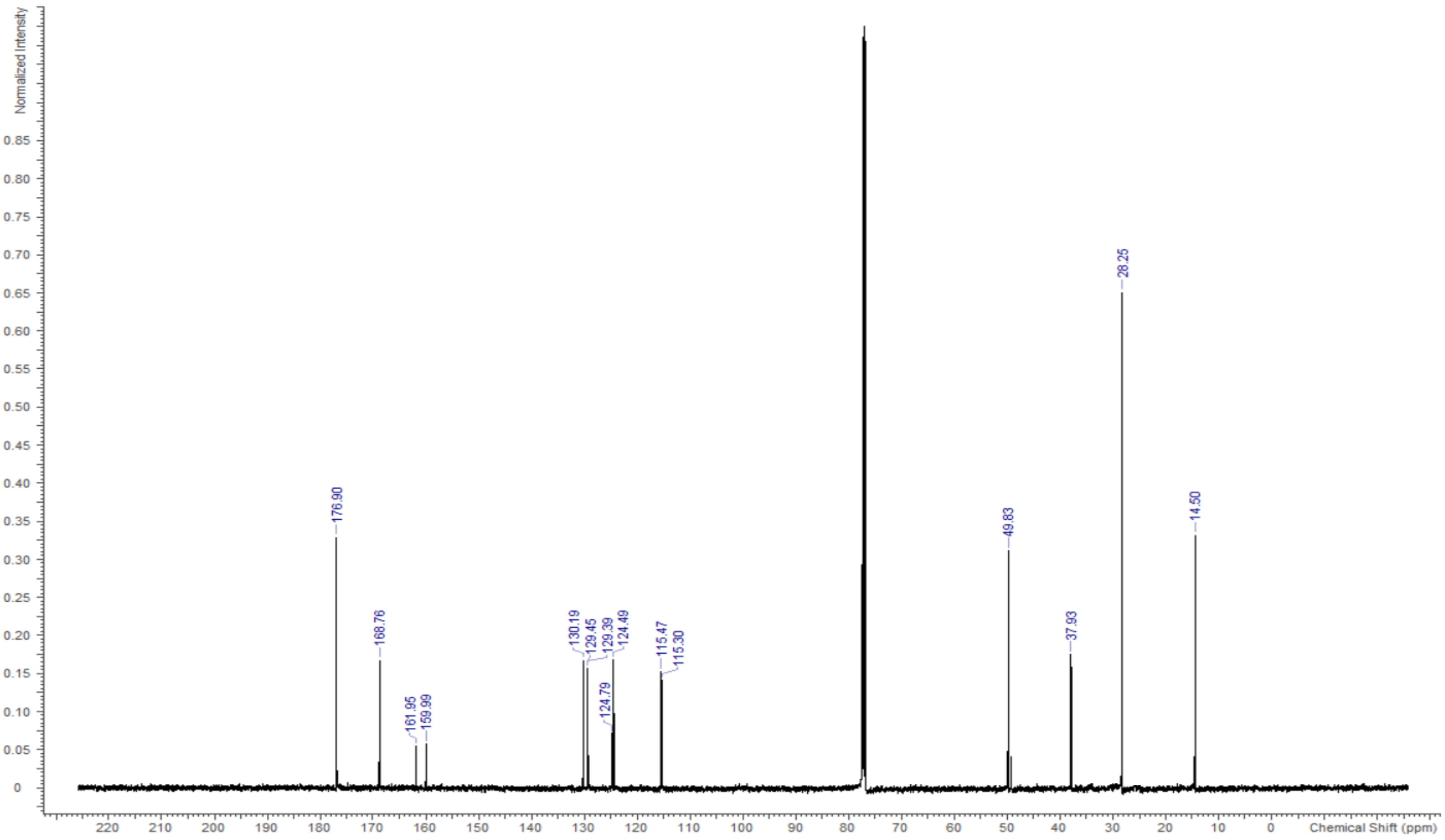

(*S*)-*N*-benzyl-2-(2,5-dioxopyrrolidin-1-yl)propanamide **(*S*)-7** ^{1}HNMR

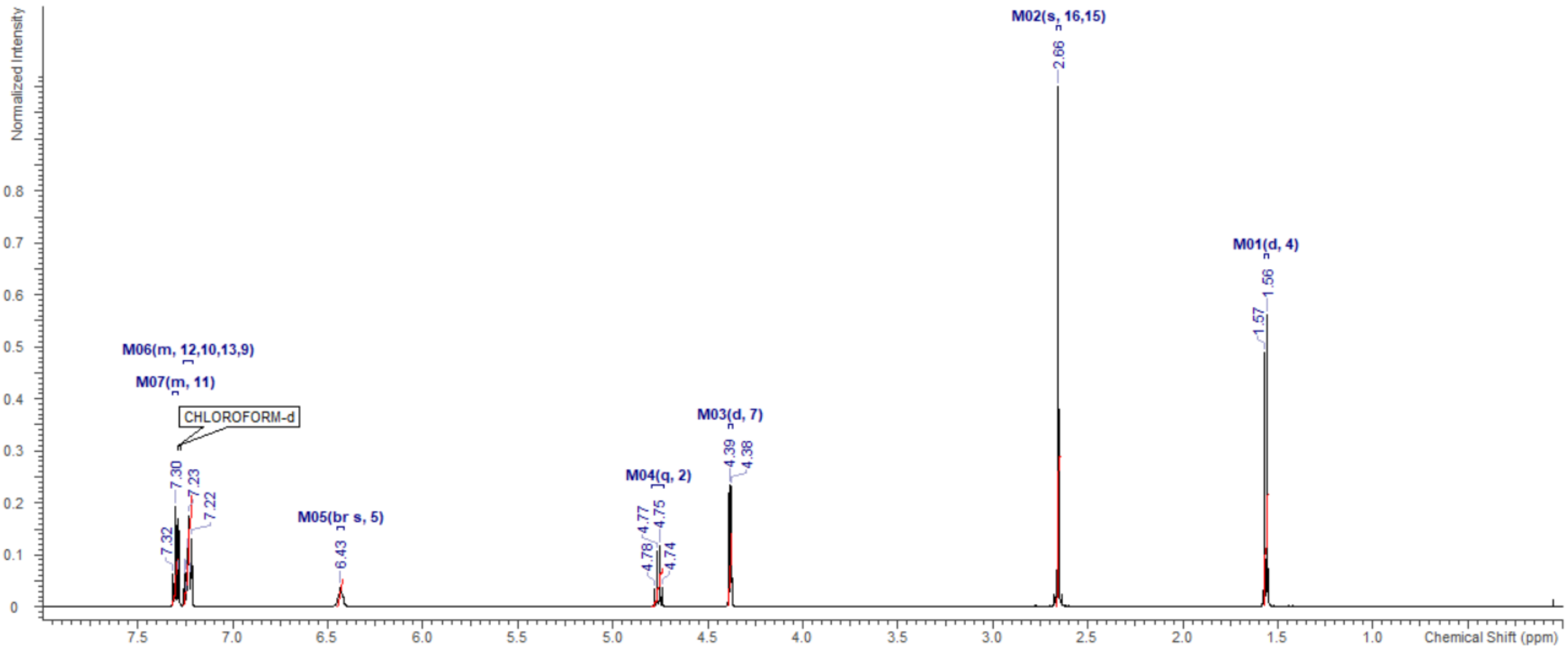

(*S*)-*N*-benzyl-2-(2,5-dioxopyrrolidin-1-yl)propanamide **(*S*)-7** ^{13}CNMR

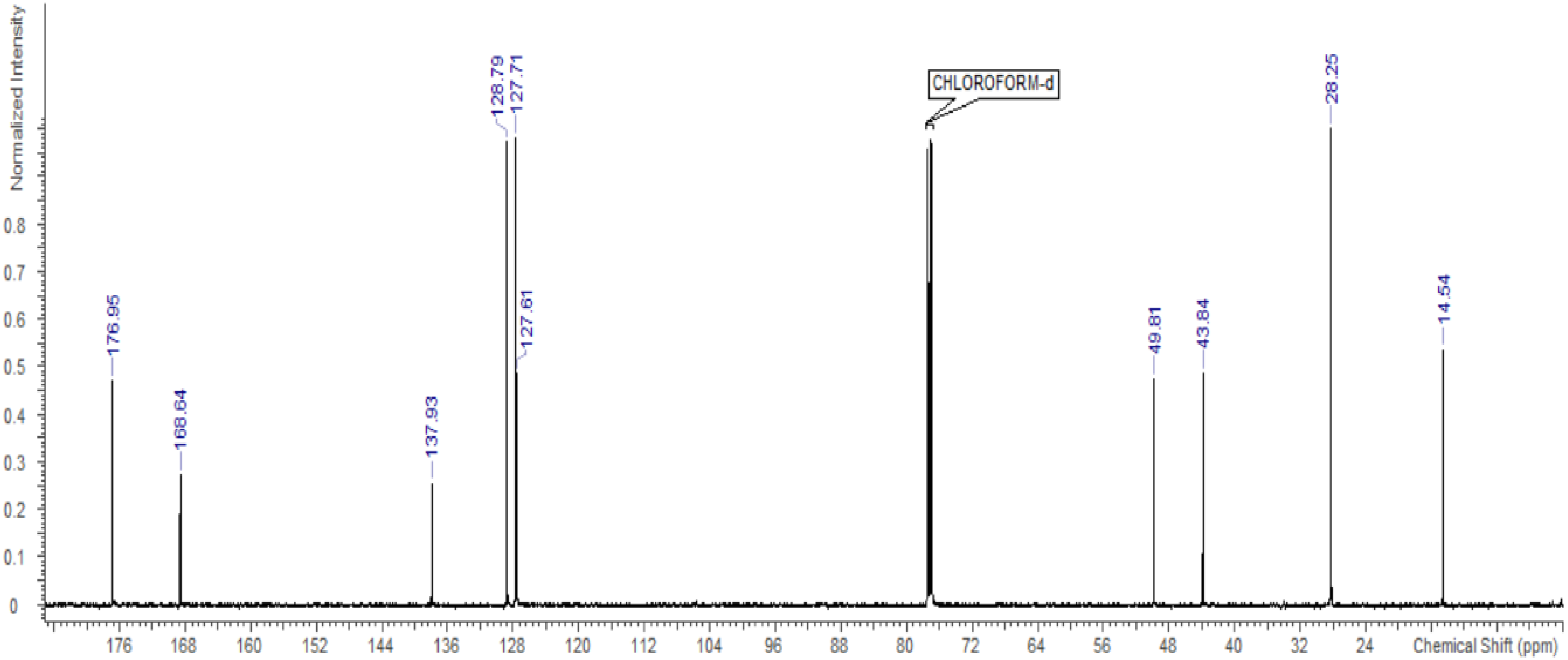

(*S*)-2-(2,5-dioxopyrrolidin-1-yl)-*N*-(2-fluorobenzyl)propanamide **(*S*)-8** ^{1}HNMR

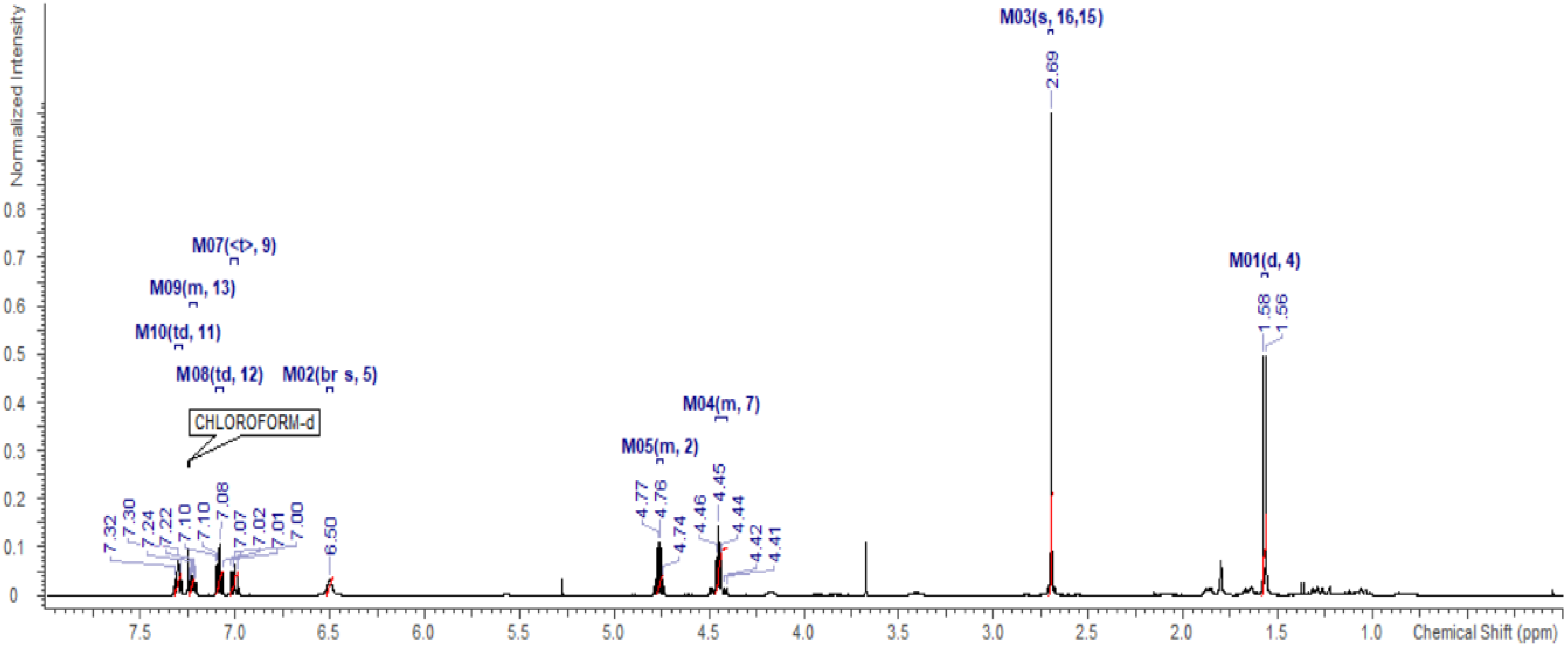

(*S*)-2-(2,5-dioxopyrrolidin-1-yl)-*N*-(2-fluorobenzyl)propanamide **(*S*)-8** ^{13}CNMR

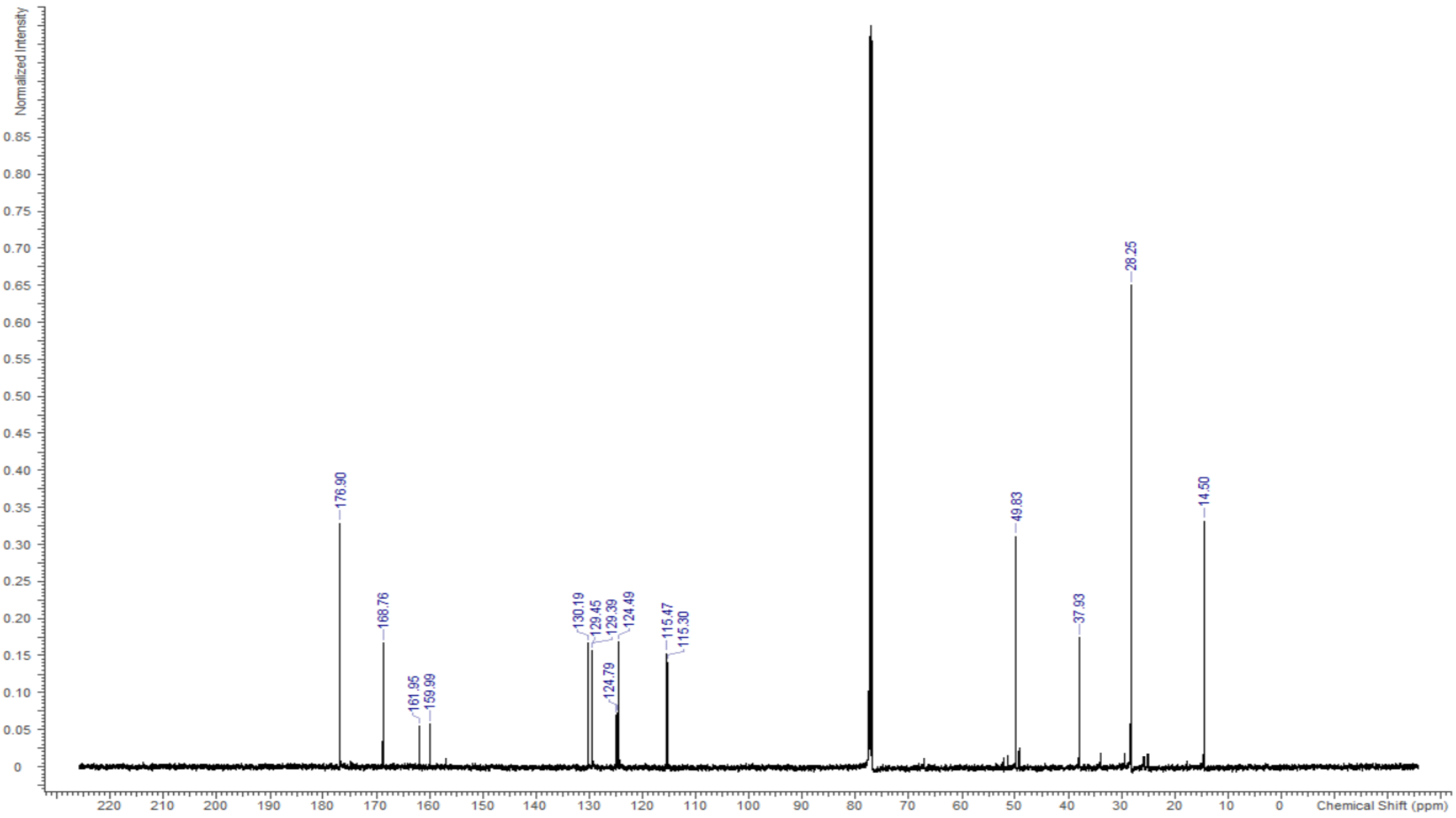

Chiral HPLC chromatograms

(*R*)-*N*-benzyl-2-(2,5-dioxopyrrolidin-1-yl)propanamide **(*R*)-7**

Analysis Report

<Sample Information>

Sample Name	: R-AS-1		
Sample ID	:		
Data Filename	: R-AS-1.lcd		
Method Filename	: chiralne.lcm		
Batch Filename	:		
Vial #	: 1-43	Sample Type	: Unknown
Injection Volume	: 10 uL		
Date Acquired	: 02.03.2020 15:24:25	Acquired by	: Michal Abram
Date Processed	: 02.03.2020 17:05:46	Processed by	: Michal Abram

<Chromatogram>

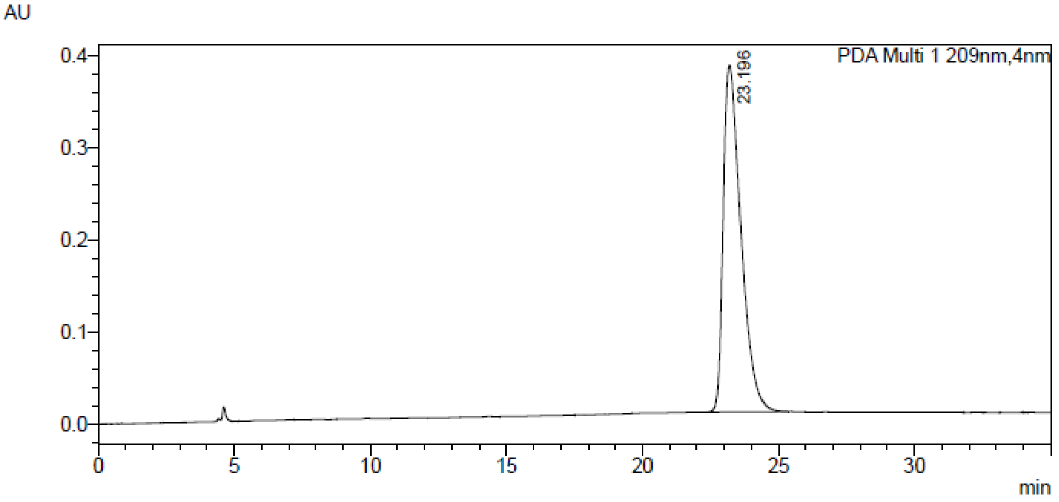

<Peak Table>

PDA Ch1 209nm

Peak#	Ret. Time	Height	Area%
1	23.196	375665	100.000
Total		375665	100.000

(*S*)-*N*-benzyl-2-(2,5-dioxopyrrolidin-1-yl)propanamide **(*S*)-7**

Analysis Report

<Sample Information>

Sample Name	: S-AS-1
Sample ID	:
Data Filename	: S-AS-1.lcd
Method Filename	: chiralne.lcm
Batch Filename	:
Vial #	: 1-41
Injection Volume	: 10 uL
Date Acquired	: 02.03.2020 16:58:32
Date Processed	: 02.03.2020 17:33:35
Sample Type	: Unknown
Acquired by	: Michal Abram
Processed by	: Michal Abram

<Chromatogram>

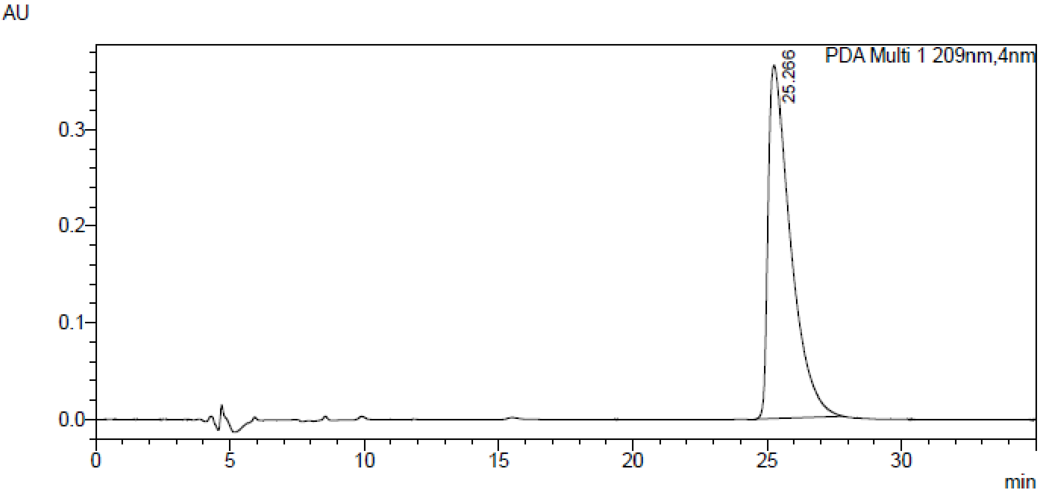

<Peak Table>

PDA Ch1 209nm

Peak#	Ret. Time	Height	Area%
1	25.266	365599	100.000
Total		365599	100.000

(*R*,*S*)-*N*-benzyl-2-(2,5-dioxopyrrolidin-1-yl)propanamide **(*R*,*S*)-7**

Analysis Report

<Sample Information>

Sample Name : R,S-AS-1
Sample ID :
Data Filename : R,S-AS-1.lcd
Method Filename : chiralne.lcm
Batch Filename :
Vial # : 1-42
Sample Type : Unknown
Injection Volume : 10 uL
Date Acquired : 02.03.2020 16:06:58
Acquired by : Michal Abram
Date Processed : 02.03.2020 16:42:01
Processed by : Michal Abram

<Chromatogram>

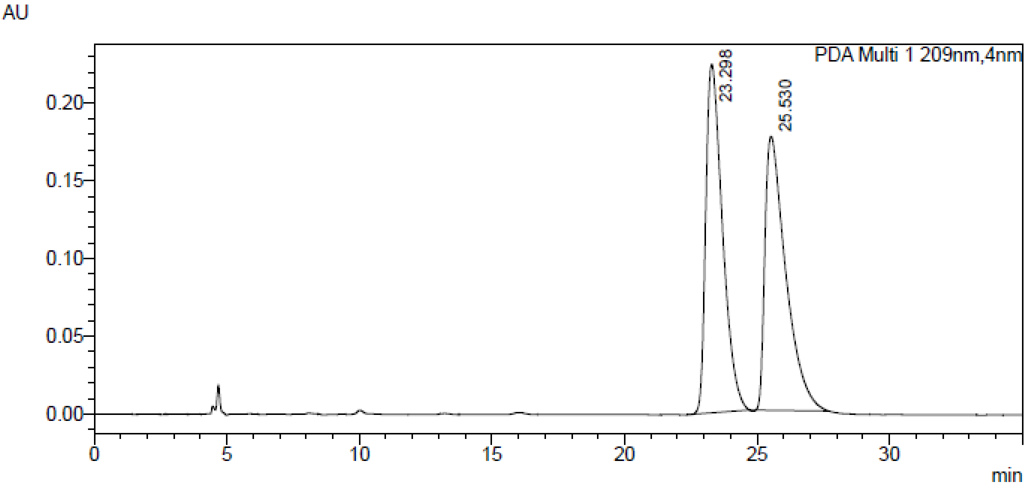

<Peak Table>

PDA Ch1 209nm

Peak#	Ret. Time	Height	Area%
1	23.298	224496	50.524
2	25.530	176123	49.476
Total			100.000

(*R*)-2-(2,5-dioxopyrrolidin-1-yl)-*N*-(2-fluorobenzyl)propanamide **(*R*)-8**

Analysis Report

<Sample Information>

Sample Name : R-AS-7
Sample ID :
Data Filename : R-AS-7.lcd
Method Filename : chiralneKA.lcm
Batch Filename :
Vial # : 1-2
Sample Type : Unknown
Injection Volume : 10 uL
Date Acquired : 24.06.2021 16:38:56
Acquired by : Chemia leków1
Date Processed : 24.06.2021 17:04:17
Processed by : Chemia leków1

<Chromatogram>

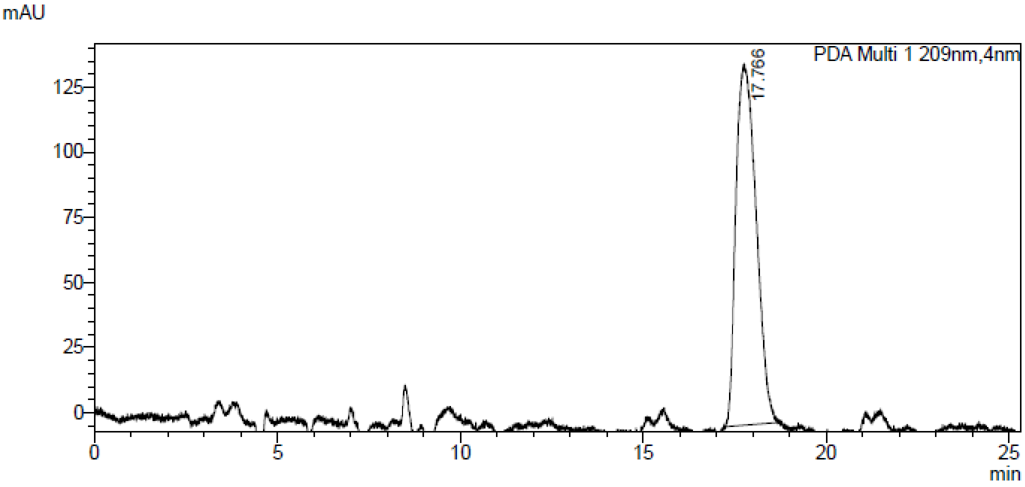

<Peak Table>

PDA Ch1 209nm

Peak#	Ret. Time	Area	Area%
1	17.766	5322466	100.000
Total		5322466	100.000

(*S*)-2-(2,5-dioxopyrrolidin-1-yl)-*N*-(2-fluorobenzyl)propanamide **(*S*)-8**

Analysis Report

<Sample Information>

Sample Name	: S-AS-7		
Sample ID	:		
Data Filename	: S-AS-7.lcd		
Method Filename	: chiralneKA.lcm		
Batch Filename	:		
Vial #	: 1-3	Sample Type	: Unknown
Injection Volume	: 10 uL		
Date Acquired	: 05.07.2021 16:12:54	Acquired by	: Chemia leków1
Date Processed	: 05.07.2021 16:49:38	Processed by	: Chemia leków1

<Chromatogram>

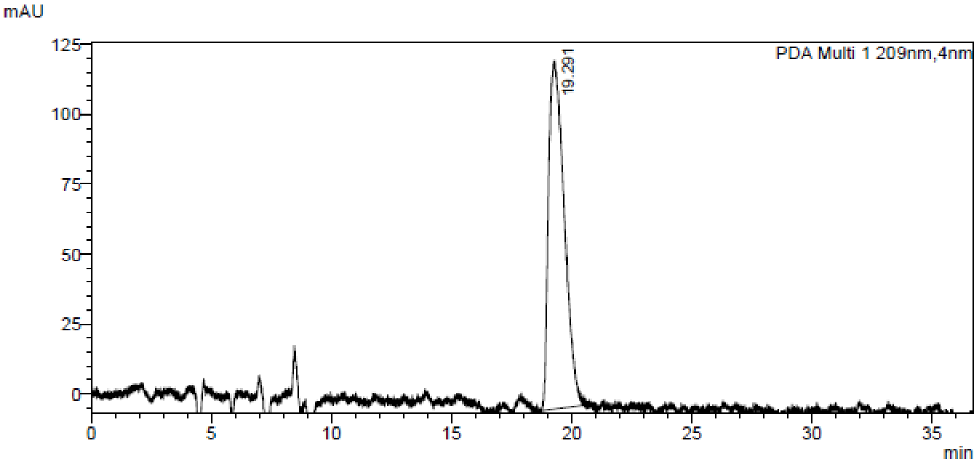

<Peak Table>

PDA Ch1 209nm

Peak#	Ret. Time	Area	Area%
1	19.291	5651044	100.000
Total		5651044	100.000

(*R*,*S*)-2-(2,5-dioxopyrrolidin-1-yl)-*N*-(2-fluorobenzyl)propanamide **(*R*,*S*)-8**

Analysis Report

<Sample Information>

Sample Name : R,S-AS-7
Sample ID :
Data Filename : R,S-AS-7.lcd
Method Filename : chiralneKA.lcm
Batch Filename :
Vial # : 1-1
Injection Volume : 10 uL
Date Acquired : 24.06.2021 16:09:04
Date Processed : 24.06.2021 16:36:56

Sample Type : Unknown
Acquired by : Chemia leków1
Processed by : Chemia leków1

<Chromatogram>

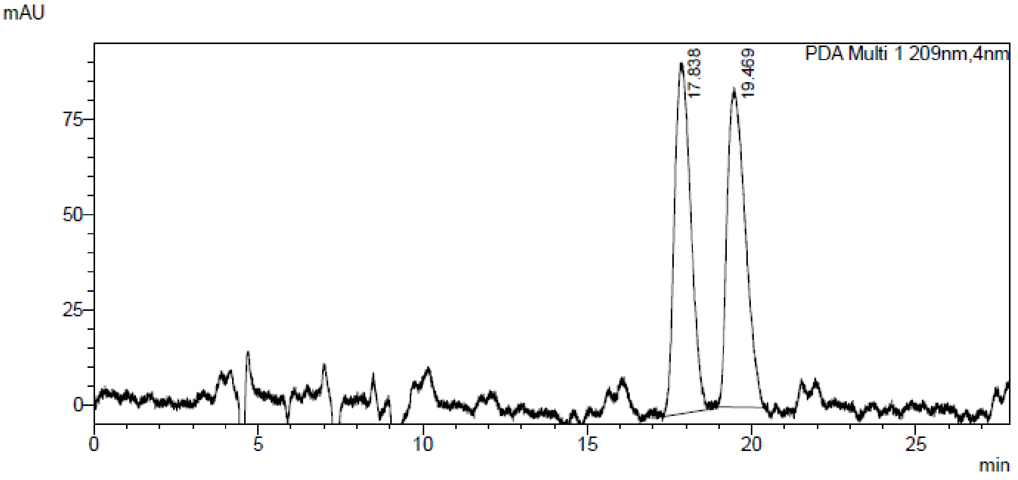

<Peak Table>

PDA Ch1 209nm

Peak#	Ret. Time	Area	Area%
1	17.838	3161557	49.799
2	19.469	3187130	50.201
Total		6348687	100.000
